# Molecular Evolution of GDP-D-Mannose Epimerase (*GME*), a Key Gene in Plant Ascorbic Acid Biosynthesis

**DOI:** 10.3389/fpls.2018.01293

**Published:** 2018-09-04

**Authors:** Junjie Tao, Han Wu, Zhangyun Li, Chunhui Huang, Xiaobiao Xu

**Affiliations:** ^1^College of Agronomy, Jiangxi Agricultural University, Nanchang, China; ^2^Institute of Kiwifruit, Jiangxi Agricultural University, Nanchang, China

**Keywords:** ascorbic acid, *GME*, molecular evolution, L-galactose pathway, Viridiplantae

## Abstract

The widespread ascorbic acid (AsA) plays a vital role in plant development and abiotic stress tolerance, but AsA concentration varies greatly among different plants. GDP-D-mannose epimerase (GME), which catalyzes GDP-D-mannose to GDP-L-galactose or GDP-L-gulose, is a key enzyme in plant AsA biosynthesis pathway. Functions and expression patterns of *GME* have been well studied in previous works, however, little information is known about the evolutionary patterns of the gene. In this study, *GME* gene structure, corresponding conserved protein motifs and evolutionary relationships were systematically analyzed. A total of 111 *GME* gene sequences were retrieved from 59 plant genomes, which representing almost all the major lineages of Viridiplantae: dicotyledons, monocotyledons, gymnosperms, pteridophytes, bryophytes, and chlorophytes. Results showed that homologs of *GME* were widely present in Viridiplantae. *GME* gene structures were conservative in higher plants, while varied greatly in the basal subgroups of the phylogeny including lycophytes, bryophytes, and chlorophytes, suggesting *GME* gene structure might have undergone severe differentiation at lower plant and then gradually fixed as plant evolution. The basic motifs of GME were strongly conserved throughout Viridiplantae, suggesting the conserved function of the protein. Molecular evolution analysis showed that strong purifying selection was the predominant force in the evolution of *GME*. A few branches and sites under episodic diversifying selection were identified and most of the branches located in the subgroup of chlorphytes, indicating episodic diversifying selection at a few branches and sites may play a role in the evolution of *GME* and diversifying selection may have occurred at the early stage of Viridiplantae. Our results provide novel insights into functional conservation and the evolution of *GME*.

## Introduction

L-ascorbic acid (AsA), also well known as vitamin C or ascorbate, is an essential water-soluble micronutrient for both animals and plants. As a general antioxidant and a cofactor for enzymes, AsA plays important roles in the process of sustaining biological activities in living organisms. Earlier studies revealed that AsA is a versatile reducing agent with anticancer properties (Ma et al., 2014; Yun et al., 2015; Doskey et al., 2016). In plant tissues, AsA is a multifunctional metabolite and has been implicated in numerous cellular processes of growth and development, including photosynthesis (Talla et al., 2011), seed set and germination (Liu et al., 2010; Ye et al., 2012), flowering (Barth et al., 2006; Kotchoni et al., 2009), senescence (Wang et al., 2011; Zhang et al., 2013) and so on. Moreover, AsA also plays a role in promoting plant tolerance to various environmental stresses (Gallie, 2012; Akram et al., 2017).

Although AsA distribute widely over Viridiplantae, different organisms have varying amounts of AsA concentration. In lower photosynthetic organism of cyanobacteria, AsA level is about 250-fold lower than that in higher plants (Tel-or et al., 1986; Obinger et al., 1998). In green algae, *Ulva compressa* and *Ulva fasciata* have very similar levels of AsA and the concentration is about 0.5 μmol g^−1^ fresh weight (FW) (Shiu and Lee, 2005; Mellado et al., 2012). In mosses, AsA concentrations are relatively closer with algae, such as the levels of AsA in *Hypnum plumaeforme*, *Brachythecium velutinum*, and *Marchantia polymorpha* are 0.1–0.6 μmol g^−1^ FW, 0.25–0.5 μmol g^−1^ FW, and 0.3 μmol g^−1^ FW, respectively (Paciolla and Tommasi, 2003; Sun et al., 2010). However, the ascorbate concentrations are still about several-fold lower in algae and mosses than in higher plants, which generally have a wide range of variability of AsA contents from 2 to 138 μmol g^−1^ FW (Gest et al., 2013). Furthermore, the amount of ascorbate has been shown to have considerable variation even within a genus or species. For example, in the genus *Actinidia*, *A. chinensis* var. *chinensis* has concentrations about 2.84–23.86 μmol g^−1^ FW, and *A. chinensis* var. *deliciosa* contains between 2.84 and 14.2 μmol g^−1^ FW, while *A. eriantha* can have concentrations over 28.4 μmol g^−1^ FW (Huang et al., 2004). The AsA concentrations in different plants are largely affected by external environmental factors, such as light intensity, altitude, and temperature (Gest et al., 2013). In addition, internal molecular mechanisms, such as biosynthesis, oxidation and recycling, also play important roles in maintaining optimal levels of ascorbate in plant growth and development.

**FIGURE 1:**
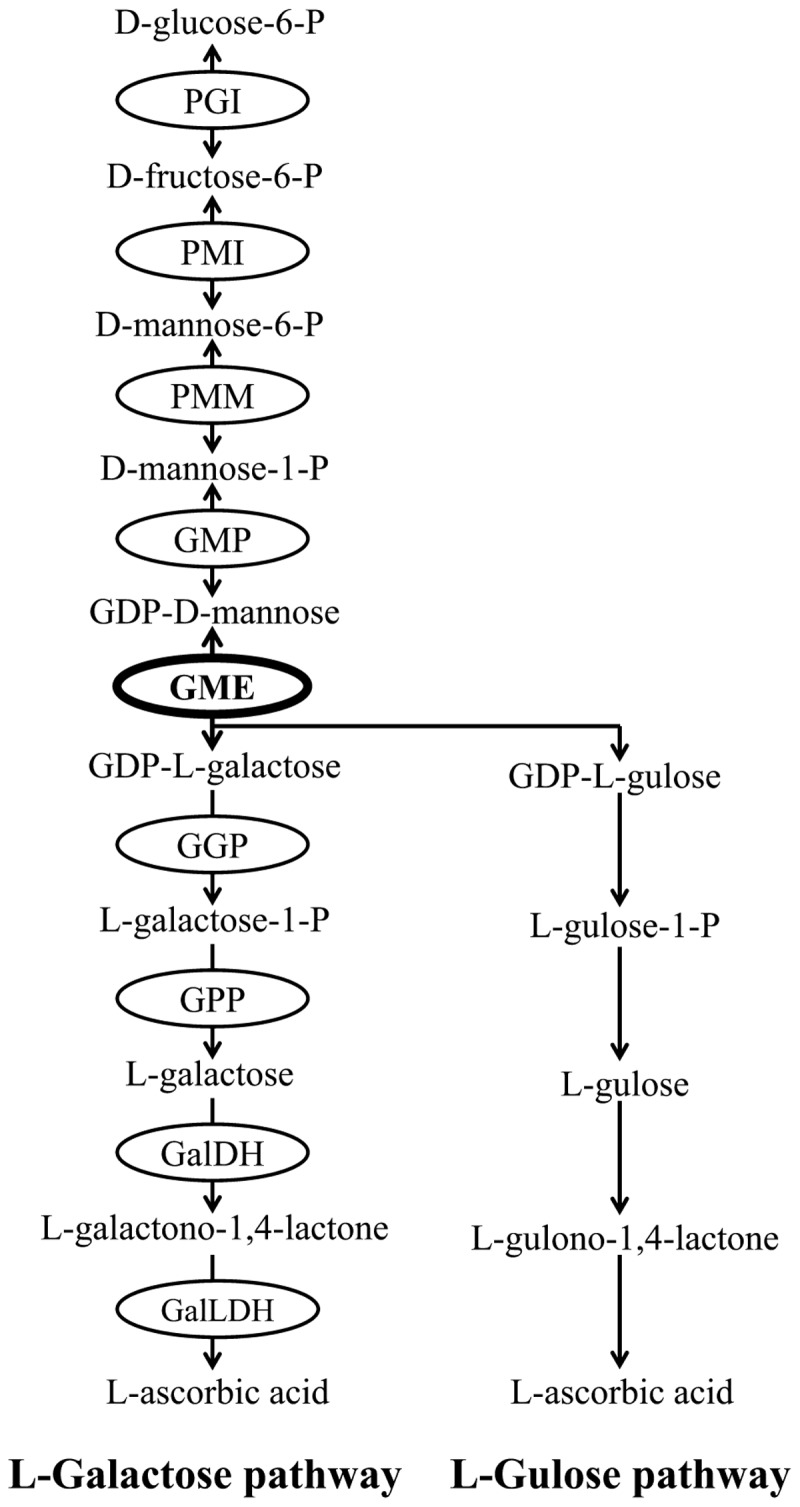
L-galactose pathway and L-gulose pathway of plant AsA biosynthesis. Enzymes (circled) in L-galactose pathway include PGI (glucose-6-phosphate isomerase), PMI (mannose-6-phosphate isomerase), PMM (phosphomannomutase), GMP (GDP-mannose pyrophosphorylase), GME (GDP-mannose-3′,5′-epimerase), GGP (GDP-L-galactose phosphorylase), GPP (L-Galactose-1-phosphate phosphatase), GalDH (L-Galactose dehydrogenase), GalLDH (L-Galactono-1,4-lactone dehydrogenase).

Ascorbic acid biosynthetic pathways have been well documented in plants. At least four biosynthetic pathways have been identified in plants, including L-galactose pathway (Wheeler et al., 1998), L-gulose pathway (Wolucka and Van Montagu, 2003), D-Galacturonate pathway (Agius et al., 2003) and myo-Inositol pathway (Lorence et al., 2004). Among these four pathways, the L-galactose pathway has been fully elucidated and is regarded as the primary route of ascorbate biosynthesis in most plant species (Bulley and Laing, 2016). Nine structural genes are involved in L-galactose pathway and all of them have been identified and cloned from various plants (**Figure 1**).

GDP-D-mannose 3′,5′-epimerase (GME) is considered to be a key regulator of ascorbate accumulation and cell wall biosynthesis. Plant GME release two epimerization products from GDP-D-mannose, one is GDP-L-galactose in L-galactose pathway and another is GDP-L-gulose in an alternative pathway of L-gulose (**Figure 1**; Wheeler et al., 1998; Wolucka and Van Montagu, 2003). GME is the most conserved protein in ascorbate biosynthesis pathway (Wolucka and Montagu, 2007), and its function has been demonstrated in many higher plants. For example, the AsA content is decreased significantly and plant growth and development is impaired severely in GME-silenced tomato (Gilbert et al., 2009; Voxeur et al., 2011). Meanwhile, AsA accumulation is increased and abiotic stresses tolerance is enhanced obviously in GME-overexpression tomato (Zhang et al., 2011). Furthermore, GME also regulates plant vegetative growth and reproductive development in *Arabidopsis* (Qi et al., 2017). However, earlier studies in some plants, such as kiwifruit (Bulley et al., 2009; Li et al., 2013) and peach (Imai et al., 2009), showed that GME alone may not determine AsA levels. As a result, further studies from more-broadly sampled taxa are needed to improve our understandings of the exact function of GME in regulating plant AsA biosynthesis.

Previous studies of *GME* mainly focused on gene expression patterns, regulatory mechanisms and functional analysis, while the evolutionary patterns of *GME* in plants are still unclear till now. In the present study, *GME* sequences were retrieved from 59 plant species which covering the main lineages of Viridiplantae and the molecular evolution of *GME* was investigated by phylogenetic analyses and molecular evolutionary approaches. Our results provide novel insights into the evolution and the function of *GME*, particularly in the context of AsA biosynthesis.

## Materials and Methods

### Sequence Acquisition and Characterization

The genomic DNA sequences, coding sequences and corresponding protein sequences of plant *GME* genes used in this study were retrieved from the public databases Phytozome v12.1^1^ and National Center of Biotechnology Information (NCBI)^2^. To identify genes encoding GME proteins in Viridiplantae, the AtGME (AT5G28840) amino acid sequence of *Arabidopsis thaliana* and AeGME (MG383560) protein sequences of *A. eriantha* were used as queries to perform BLASTP searches with default algorithm parameters in Phytozome v12.1 database (Goodstein et al., 2012). Additionally, GME protein sequences of *A. deliciosa* and *A. rufa*, which were obtained from NCBI using AeGME sequence as a query, were also used in following analyses. The identical, redundant, and incomplete sequences were removed manually from the downloaded data set using BioEdit v7.1.3 (Hall, 1999), and only the full-length coding sequences were retained. Then, the potential plant GME protein sequences retrieved from Phytozome v12.1 and NCBI were checked and analyzed using HMMER website^3^ (Finn et al., 2015) and the CD-search tool in the Conserved Domain Database of NCBI^4^ (Marchler-Bauer et al., 2015) to confirm the presence of NAD-binding domain, which is a well conserved functional domain of plant GMEs (Watanabe et al., 2006; Siow et al., 2013).

### Multiple Sequence Alignment

Multiple sequence alignment for all the selected plant GME amino acid sequences was firstly generated using MAFFT v7.158 (Katoh and Standley, 2013) by the auto option, after which they were manually adjusted using BioEdit v7.1.3 (Hall, 1999). Then, the alignment of the amino acid sequences was converted into the alignment of nucleotide sequences in fixed codon positions utilizing PAL2NAL^5^ (Suyama et al., 2006). Finally, Gblocks v0.91b (Castresana, 2000) was used to identify conserved blocks and regions that had gaps in more than 50% of the sequences in the multiple alignment of nucleotide sequence were removed.

### Recombination Detection

Phylogenetic reconstruction and molecular evolutionary analysis can be profoundly influenced by recombination (Posada and Crandall, 2002; Anisimova et al., 2003; Shriner et al., 2003). To avoid the impact of recombination, Genetic Algorithm Recombination Detection (GARD) (Kosakovsky Pond et al., 2006) method implemented in Datamonkey website^6^ (Delport et al., 2010) was used to test whether there was any evidence of recombination in plant *GME* nucleotide sequences.

### Phylogenetic Tree Reconstruction

The phylogenetic tree reconstruction was performed using Bayesian inference method implemented in MrBayes v3.1.2 software (Ronquist and Huelsenbeck, 2003). Prior to the phylogenetic analysis, the most suitable nucleotide substitution model was firstly determined using the AIC criterion in MrModeltest v2.3 (Nylander, 2008). The Bayesian phylogenetic estimation was conducted by running four independent Markov Chain Monte Carlo (MCMC) algorithms (three heated chains and one cold chain) for 10,000,000 generations. Trees were sampled every 100 generations and the first 25% of the samples were discarded as burn-in. Finally, the resulting phylogenetic tree was edited and visualized in iTOL^7^.

### Gene Structure and Conserved Motifs Analyses

To analysis gene structure of *GME*, the Gene Structure Display Server v2.0 (GSDS v2.0^8^) was used to display the exon-intron organizations of *GME* by uploading *GME* genomic DNA sequences and their corresponding cDNA sequences to the website. Conserved motifs of GME were identified using the Multiple Expectation Maximization for Motif Elicitation (MEME) v4.12.0^9^ with the maximum number of motifs = 8 (optimized based on an iterative analysis), motif width between 15 and 50 aa, and other parameters at default setting.

### Molecular Evolution Analyses

The ratio of non-synonymous (*d*_N_) over synonymous substitutions (*d*_S_) (ω = *d*_N_/*d*_S_) is an effective indicator to evaluate selective pressures acting on protein-coding genes, where ω > 1, ω < 1, and ω = 1 indicates diversifying positive selection, negative selection and neutral evolution, respectively. To investigate the influence of selective pressures acting on *GME* genes in Viridiplantae, codon-based maximum likelihood methods implemented in codeml program from PAML package version 4.7 were utilized (Yang, 2007). The reconstructed Bayesian phylogenetic tree and the alignment of codon-based nucleotide sequences were input into codeml program to perform molecular evolution analyses. The site-specific models were firstly employed to examine the selective pressure at amino acid sites and identify potential sites under diversifying selection (Yang et al., 2000). Three pairs of log-likelihood ratio tests (LRTs) of M0 (one-ratio model) vs. M3 (discrete model), M1a (neutral model) vs. M2a (selection model), M7 (β) vs. M8 (β and ω) were employed to detect the variation of ω among sites. For one LRT, the difference in the number of parameters between nested models was used as degree of freedom, and twice the difference of the log likelihood values between the two models was compared with chi-square (χ^2^) distribution. The comparison of M0 with M3 was used to evaluate the heterogeneity of ω among sites, while the other two comparisons (M2a versus M1a and M8 versus M7) were used to detect presence of sites evolving under positive selection. Amino acids under positive selection were identified using the Bayes empirical Bayes method (BEB) (Yang et al., 2005).

To test for possible rate heterogeneity of ω ratios across different branches, branch models of free-ratio model (Mf) and one-ratio model (M0) were compared using LRT method. Mf model allows each branch to have an independent ω, while the M0 allows all branches and sites to have a single ω ratio. Branch-site model was further performed to detect the role of positive selection acting on a specific branch (Zhang et al., 2005). For this analysis, the alternative model of branch-site model A (unfixedω) and the corresponding null model (ω fixed to 1) were also compared using LRT. The branches of interest, such as within a lineage or a particular species, were used as foreground branches, and the remaining lineages or branches were used as background branches. Sites under positive selection in the foreground branch were identified using BEB method if the LRT is significant.

In addition, other four most likelihood methods implemented in the Datamonkey web server (see footnote 6) (Delport et al., 2010), including Fixed Effects Likelihood (FEL), Single Likelihood Ancestral Counting (SLAC), Fast Unconstrained Bayesian AppRoximation (FUBAR), and Mixed Effects Model of Evolution (MEME), were also employed to test the impact of positive selection on *GME* and confirm the results of PAML.

## Results

### Identification of *GME* Genes in Viridiplantae

BLAST results showed that *GME* genes are widely present in various plant species. A total of 111 *GME* homologous from 59 Viridiplantae species were obtained in the final data (**Supplementary Table 1** and **Supplementary Data 1**, **2**). These plant species covered a broad range of the major lineages of Viridiplantae, including dicotyledons, monocotyledons, gymnosperms, pteridophytes, bryophytes, and unicellular green algae. *GME* sequences of *A. deliciosa*, *A. rufa*, *A. erantina*, and *Picea sitchensis* were downloaded from NCBI database, while the rest of *GME* sequences were retrieved from Phytozome v12.1 database. The detailed information of the obtained plant *GME* sequences was provided in **Supplementary Table 1**.

All obtained GME candidates were checked to confirm the presence of conserved domains in their proteins sequences. The results of the NCBI conserved domain database showed that all the predicted GME proteins used in this study containing the NDA-binding domain. In addition to this, the conserved epimerase domain was also detected in all the predicted GME proteins by both the NCBI conserved domain search and the HMMER search. These results indicated that the obtained GME proteins have similar epimerase function.

Most Viridiplantae species have different *GME* gene copy numbers (**Supplementary Table 1**). In some species, such as *A. thaliana*, *Boechera stricta*, *Spirodela polyrhiza*, *M. polymorpha*, and *Volvox carteri*, only one *GME* copy was identified. However, in some other plant species, such as *Brassica rapa*, *Zea mays*, *P. sitchensis*, *Selaginella moellendorffii*, and *Physcomitrella patens*, more than two copies of *GME* were identified. Most of the plants used in this study contained only one or two GME copies, while the eudicot plant *Glycine max*, the monocot plant *Panicum virgatum*, the gymnosperm plant *P. sitchensis*, the lycophyte plant *S. moellendorffii*, and the bryophyte plant *P. patens* possessed 5, 5, 3, 9, 3 *GME* gene copies, respectively.

### Recombination Test and Phylogenetic Analyses

**FIGURE 2:**
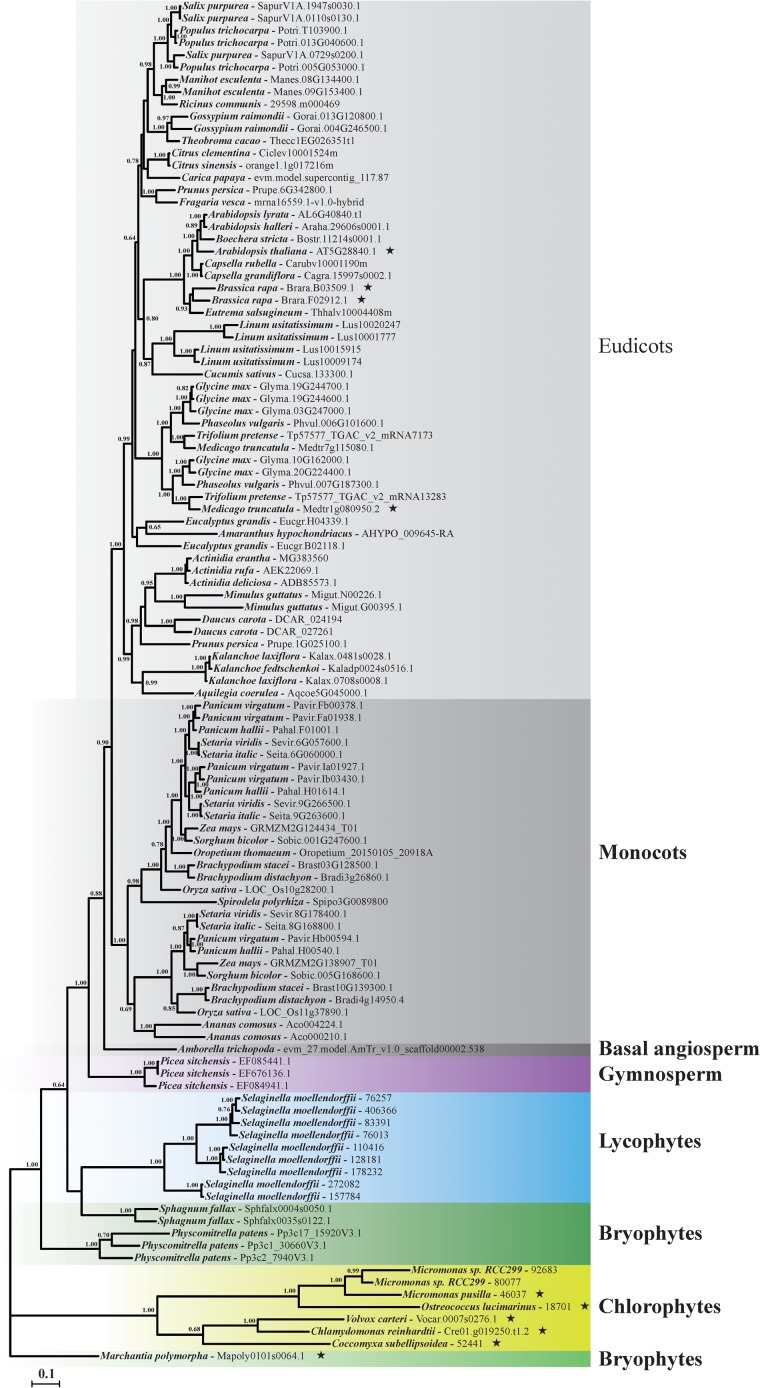
Phylogenetic relationships of *GME* gene in Viridiplantae. The phylogenetic tree of plant *GMEs* was constructed by the Bayesian analyses method based on the best fitted nucleotide substitution model of GTR+I+G. Posterior probabilities above 0.6 were labeled near the nodes. Accession number of each GME sequence was listed behind the species name. Branches identified under episodic diversifying selection by branch-site model were labeled using asterisk marks (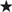).

Gaps in more than 50% of the sequences in the nucleotide sequence alignment were removed. No evidence of recombination was found by GARD method implemented in Datamonkey website, suggesting the sequence alignment of the collected data could be used in subsequent phylogenetic reconstruction and molecular evolutionary analyses. The best-fit model of GTR+I+G was selected by AIC standard in MrModeltest and used in phylogenetic reconstruction analysis. Results showed that the Bayesian phylogenetic tree of plant *GME* was basically consistent with the evolutionary relationship of Viridiplantae (**Figure 2**). All of the angiosperm plant *GME* genes (including eudicots, monocots, and basal angiosperm) formed a single clade with high bootstrap support as showed in *GME* gene tree (**Figure 2**). Plant *GME* genes in gymnosperm, lycophytes, and chlorophytes also formed a single clade, respectively. However, *GME* genes of bryophytes did not form a monophyletic clade, and were positioned in three different clades (**Figure 2**).

### Genomic Structure of *GME* Genes and Sequence Feature of GME Proteins

**FIGURE 3:**
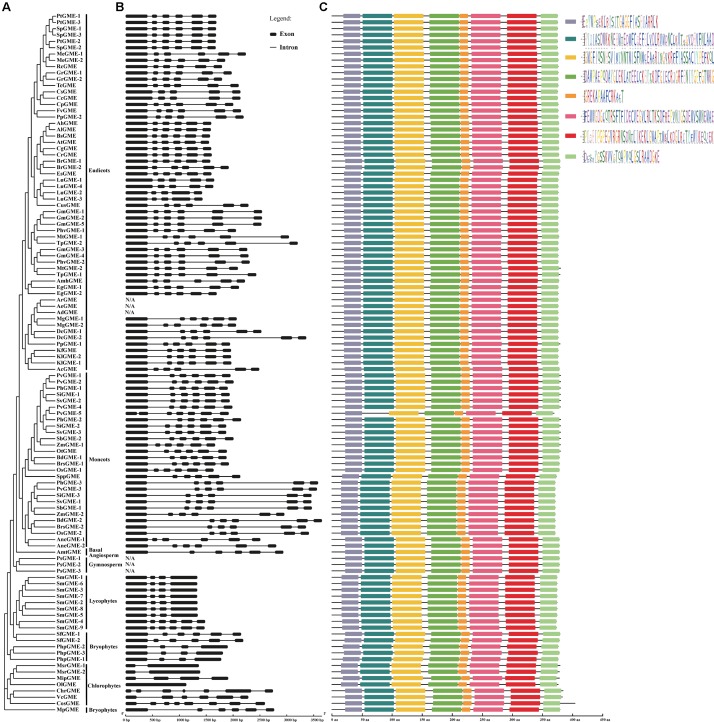
*GME* gene structure and conserved motifs in Viridiplantae. **(A)** Phylogenetic tree of plant *GME* genes. **(B)** Exon-intron structures of plant *GME* genes. **(C)** Schematic diagram of conserved motifs of GME proteins. Different motifs were shown by different-colored boxes.

The exon-intron organization was examined to understand the structural diversity and the evolutionary relationship of plant *GME* genes. As the *GME* genomic DNA sequences of *A. deliciosa*, *A. rufa*, *A. erantina*, and *P. sitchensis* were unavailable right now, their exon-intron structures were not displayed. The exon-intron structures of plant *GME* genes were relatively conserved and most of the neighboring sequences in the phylogenetic tree tend to have similar gene structures according to the results (**Figure 3**). Except a few exceptions, the *GME* gene structures in the subgroup of angiosperms (including eudicots, monocots, and basal angiosperm) were highly conserved and almost all contained six exons in their coding regions. The *CsGME* (*Citrus sinensis*), *CcGME* (*Citrus clementina*), and *LuGME* (*Linum usitatissimum*) in eudicots subgroup contained five exons, while *PvGME-5* (*P. virgatum*) in monocots contained seven exons. It could be concluded that one intron of *CsGM*, *CcGME*, and *LuGME* may be lost during the evolution of these sequences by comparing their structure with others. For *PvGME-5*, the first exon appears truncated and joined with another; this sequence (369 aa) with seven exons is the shortest in this study (**Figure 3**). Two main types of exon-intron structures (four and six exons) were identified in the subgroups of lycophytes and bryophytes. In the chlorophytes subgroup, the *GME* gene structures were not conserved, and various types of gene structures can be observed including one, two, four, six, seven, and eight exons. Except for *OlGME* (*Ostreococcus lucimarinus*) where no intron structure was observed, most Viridiplantae species contained at least one intron, and the intron number or length was variable among most species.

The full-length of most GME amino acid sequences was around 377 aa (**Supplementary Table 1**) and shared 62.6–100% pairwise protein sequence identity. The length of GME varied from 369 to 447 aa (**Supplementary Table 1**), suggesting certain degree of insertion or deletion events may have occurred during the evolution of plant GME. To better understand the protein evolution, the conserved motifs of GME were investigated using MEME v4.12.0. Unlike the variation of exon-intron structures, the basic motifs of GME were strongly conserved throughout Viridiplantae (**Figure 3**). Except PvGME-5, which lost one motif and contained only seven motifs, all remaining protein sequences contained eight conserved motifs.

### Molecular Evolution Analyses

Maximum-likelihood methods implemented in PAML, including specific site model, branch model, and branch-site model, were used to detect the selective pressures acting on plant *GME* genes. Three pairs of site-specific models were conducted to test positively selected sites on the evolution of plant *GME*. The maximum likelihood estimates of the ω values under the one-ratio model M0 for *GME* genes was 0.0287 (**Supplementary Table 2**), which approximates zero, indicating that the evolution of Viridiplantae GME genes was under strong purifying selection. The LRT test for model M0 versus M3 was statistically significant (−2ΔlnL = 1861.68, *p* < 0.001) (**Supplementary Table 2**), indicating that ω ratio was variable among amino acid sites along GME sequences. Then to determine the selective constraint among sites and identify potential sites under positive selection, the designated models were compared against their corresponding null models (i.e., M2a versus M1a and M8 versus M7). Both LRTs showed that the alternative models of M2a and M8 were not significantly better than null models of M1a and M7, respectively, and no amino acid site was detected to be under positive selection (**Supplementary Table 2**).

As positive selection may only influence some specific branches, branch-specific models of free-ratio model (Mf) and one-ratio model (M0) were compared to detect selective constraints among different branches. The LRT test showed that the Mf was significantly better than the M0 (−2ΔlnL = 5197.214, *p* < 0.001), suggesting that selective constraint level was heterogeneous among branches (**Supplementary Table 3**). The modified branch-site models were further used to detect selective pressure on different clades or branches and also identify sites influenced by positive selection. Firstly, the main lineages of eudicots, monocots, gymnosperm, lycophytes, and chlorophytes were used as foreground branch, respectively, while rest branches in the phylogenetic tree were used as background branches. According to the LRTs results, no significant differences were identified between the foreground branches and the background branches (**Supplementary Table 4**). Secondly, to test whether a particular branch was under positive selection during evolution process, each branch in the phylogenetic tree was labeled as foreground branch and the rest of the branches were treated as background branch. The LRTs identified 10 branches, including *A. thaliana*, *B. rapa* (*BrGME-1*), *B. rapa* (*BrGME-2*), *Chlamydomonas reinhardtii*, *Coccomyxa subellipsoidea*, *Medicago truncatula* (*MtGME-2*), *Micromonas pusilla*, *O. lucimarinus*, *V. carteri*, *M. polymorpha*, were significantly better fit to the data than their corresponding null models (**Supplementary Table 4**). And amino acid sites under episodic positive selection were identified in each foreground branch with BEB posterior probabilities criterion of 95% (**Supplementary Table 4**). It was worth noting that five branches of *C. reinhardtii*, *C. subellipsoidea*, *M. pusilla*, *O. lucimarinus*, *V. carteri* belonged to chlorophytes subgroup, one branch of *M. polymorpha* belonged to bryophytes subgroup and four branches of *A. thaliana*, *B. rapa* (*BrGME-1*), *B. rapa* (*BrGME-2*), *M. truncatula* (*MtGME-2*) belonged to eudicots subgroup (**Figure 2**).

In addition, the FEL, SLAC, FUBAR, and MEME methods implemented in Datamonkey web server were also used to detect evidence of positive selection and verify the results of PAML. Results showed that no specific codons under positive selection were identified by methods of FEL, SLAC, and FUBAR. However, MEME identified 11 sites with evidence of episodic diversifying selection with significance level of *p* < 0.1 (**Supplementary Table 5**).

## Discussion

The reaction catalyzed by GME involves two pathways of plant AsA biosynthesis and is regarded to be a key link in plant AsA biosynthesis. Previous works about *GME* mainly focused on model plants and some cash crops, and found two copies of *GME* in tomato (Zhang et al., 2011) and only one copy of *GME* in most of other plants such as *Arabidopsis* (Wolucka et al., 2001), rice (Watanabe et al., 2006), kiwifruit (Li et al., 2013), and tea (Li et al., 2017). In this study, 111 *GME* homologous were obtained from 59 representative Viridiplantae species, which covered all the main lineages of plants including angiosperms, gymnosperms, lycophytes, bryophytes, and green algae. Except for a few plants, most Viridiplantae contained one or two *GME* homologous in the collected data set. Some plants contained more than three copies of *GME*, such as *S. moellendorffii* which contained as much as nine *GME* homologous (**Supplementary Table 1**). However, no signal of ancient whole genome duplication or polyploidy event was identified in *Selaginella* genome (Banks et al., 2011), and the mechanism of the formation of multi-copy of *GME* in these plants is still unclear. Rice genome encodes three homologs of GMPase (GDP-mannose pyrophosphorylase) which catalyzes the synthesis of GDP-D-mannose in L-galactose pathway. However, only two of the homologs involved in rice AsA biosynthesis and played different roles in the process (Qin et al., 2016). Two GME homologs in tomato participated in the biosynthesis of AsA and cell wall. However, they were specifically expressed in different organs or tissues, and had diversified roles in cell wall biosynthesis and development (Mounet-Gilbert et al., 2016). For the plants containing multiple homologs of *GME*, whether these homologs all participate in AsA biosynthesis and have same functions are still uncertain and needed to be verified in future works.

Most of the plant *GME* genes have similar structure. *GME* exon-intron structure was much conserved in eudicots and monocots, and most of the sequences contained six exon structures (**Figure 3**). The length and the arrangement of the exons in higher plants were also highly conserved. However, in the basal subgroups of the phylogenetic tree, such as lycophytes, bryophytes, and chlorophytes, the structural types of *GME* gene varied greatly (**Figure 3**). For example, six types of exon-intron structures (one, two, four, six, seven, and eight exons) were identified in the subgroup of chlorophytes. These diverse gene structures indicated that *GME* gene structure may have undergone severe differentiation at the early diverging lineages of Viridiplantae, and the structure such as exon number was gradually fixed as plant evolution. Protein is the ultimate executor of the function of the gene. GME protein motif analyses showed that the protein structure was much conserved even in the basal subgroups of the phylogenetic tree where the gene structure was relatively diversified (**Figure 3**). The conserved motif of GME protein throughout the phylogenetic tree reflects the conserved and important function of the protein.

Stressful environments, such as high/low temperature, drought and salinity, can seriously affect normal growth and development of plants by inducing the over-production of reactive oxygen species (ROS). To adapt to these abiotic stresses, plants have evolved antioxidant systems consisting of enzymatic and non-enzymatic antioxidants to neutralize ROS and protect cells from oxidative damage. AsA is an efficient and major non-enzymatic antioxidant molecule and can improve plant tolerance to abiotic stresses by over-expressing AsA biosynthesis related genes or exogenously applying AsA (Akram et al., 2017). Therefore, increasing AsA level in plants is an effective way to enhance plant resistance to stresses. Different plants contain different concentrations of AsA, and understanding the molecular evolution mechanisms that causes the concentration variation will be helpful to increase AsA levels in plants. *GME* was regarded as a key gene in plant AsA biosynthesis pathway. However, *GME* alone had small effect on AsA pool in some plants, and it usually needed to cooperate with GDP-L-galactose phosphorylase (*GGP*) to control AsA biosynthesis (Mellidou and Kanellis, 2017). In this study, strong purify selection was found to be the predominant force in the evolution of *GME*, which suggesting functional importance of the gene. Nevertheless, a small subset of branches and sites under positive selection were identified by two episodic diversifying selection methods of branch-site model and MEME. Most interesting, 5 out of 10 branches under positive selection were located in the subgroup of green algae, which indicated that diversifying selection mainly occurred in the early diverging lineages of Viridiplantae.

Plant biosynthetic pathways usually involve many genes, which are in the network of protein–protein interactions or the interaction between products and substrates. Previous studies showed that the evolutionary relationships of genes along the metabolic pathway were different, and the evolutionary rate of genes has a certain relationship with the position of genes in the pathway (Ramsay et al., 2009; Clotault et al., 2012; Nougué et al., 2014). L-galactose pathway is the major route for AsA biosynthesis in plants and genes involved in this pathway had been well documented (Akram et al., 2017). This study was the first attempt to illustrate the evolutionary pattern of the key gene *GME* which involved in L-galactose pathway. Molecular evolution analysis of other genes along the L-galactose pathway in future studies will be further improve our understanding of the evolutionary patterns of the pathway and the molecular mechanisms of AsA concentration difference among different plants.

## Conclusion

The evolutionary pattern of plant AsA biosynthesis pathway gene *GME* was analyzed in large-scale for the first time in this study. Molecular evolutionary analyses showed that *GME* was under strong purifying selection, and only a few branches and sites under episodic diversifying selection were identified, suggesting episodic diversifying selection at a few branches and sites may play a role in the evolution of *GME*. The branches under positive selection mainly presented in the lineage of chlorphytes, where the *GME* gene structures were also the most diverse, and these results suggested that *GME* had undergone severe differentiation and diversifying selection in the early stage of Viridiplantae evolution.

## Author Contributions

JT and XX conceived and designed the project. JT, HW, and ZL collected the data and performed the experiments. JT and CH analyzed the data. JT wrote the paper.

## Conflict of Interest Statement

The authors declare that the research was conducted in the absence of any commercial or financial relationships that could be construed as a potential conflict of interest.

## Footnotes

1 https://phytozome.jgi.doe.gov/pz/portal.html

2 https://www.ncbi.nlm.nih.gov/

3 https://www.ebi.ac.uk/Tools/hmmer/

4 https://www.ncbi.nlm.nih.gov/Structure/cdd/wrpsb.cgi

5 http://www.bork.embl.de/pal2nal/

6 http://www.datamonkey.org/

7 https://itol.embl.de/

8 http://gsds.cbi.pku.edu.cn/

9 http://alternate.meme-suite.org/tools/meme
