# Supplementary material for: Molecular Evolution of GDP-D-Mannose Epimerase (GME), a Key Gene in Plant Ascorbic Acid Biosynthesis
Source: Front Plant Sci. 2018 Sep 4;9:1293. doi: 10.3389/fpls.2018.01293 (PMC6132023; doi:10.3389/fpls.2018.01293)
Supplement: Supplementary file 1 [file Table_1.DOCX]

**Supplemental Table 1. List of *GME* genes in 59 representative Viridiplantae genomes**

| **Lineage** | **Species** | **Gene** | **Locus** | **Length (aa)** | **Location** |
| --- | --- | --- | --- | --- | --- |
| Eudicots | Aquilegia coerulea | AcGME | Aqcoe5G045000.1 | 378 | Chr_05:2374757..2378363 forward |
|  | Actinidia deliciosa | AdGME | ADB85573.1 | 376 | N/A |
|  | Actinidia erantina | AeGME | MG383560 | 376 | N/A |
|  | Arabidopsis halleri | AhGME | Araha.29606s0001.1 | 377 | Scaffold29606:5..2157 forward |
|  | Arabidopsis lyrata | AlGME | AL6G40840.t1 | 377 | scaffold_6:14248117..14250361 reverse |
|  | Amaranthus hypochondriacus | AmhGME | AHYPO_009645-RA | 377 | scaffold_92:655411..659157 reverse |
|  | Actinidia rufa | ArGME | AEK22069.1 | 376 | N/A |
|  | Arabidopsis thaliana | AtGME | AT5G28840.1 | 377 | Chr5:10862173..10864903 reverse |
|  | Boechera stricta | BsGME | Bostr.11214s0001.1 | 377 | Scaffold11214:56473..58719 forward |
|  | Brassica rapa | BrGME-1 | Brara.F02912.1 | 379 | A06:23602270..23604838 reverse |
|  |  | BrGME-2 | Brara.B03509.1 | 379 | A02:29252778..29255916 forward |
|  | Capsella grandiflora | CgGME | Cagra.15997s0002.1 | 377 | Scaffold15997:3997..5803 reverse |
|  | Capsella rubella | CrGME | Carubv10001190m | 377 | scaffold_6:10664200..10666963 reverse |
|  | Carica papaya | CpGME | evm.model.supercontig_117.87 | 376 | supercontig_117:885787..887790 reverse |
|  | Citrus clementina | CcGME | Ciclev10001524m | 375 | scaffold_5:29481817..29485113 forward |
|  | Citrus sinensis | CsGME | orange1.1g017216m | 375 | scaffold00255:117357..120593 forward |
|  | Cucumis sativus | CusGME | Cucsa.133300.1 | 384 | scaffold01037:1027361..1031022 forward |
|  | Daucus carota | DcGME-1 | DCAR_027261 | 376 | DCARv2_Chr8:24639540..24642067 forward |
|  |  | DcGME-2 | DCAR_024194 | 376 | DCARv2_Chr7:10573859..10577221 reverse |
|  | Eucalyptus grandis | EgGME-1 | Eucgr.H04339.1 | 376 | Chr08:59009011..59011121 reverse |
|  |  | EgGME-2 | Eucgr.B02118.1 | 376 | Chr02:40193389..40196915 forward |
|  | Eutrema salsugineum | EsGME | Thhalv10004408m | 377 | scaffold_6:4460665..4463316 reverse |
|  | Fragaria vesca | FvGME | mrna16559.1-v1.0-hybrid | 376 | LG1:19856430..19858578 reverse |
|  | Glycine max | GmGME-1 | Glyma.19G244600.1 | 376 | Chr19:49179855..49183396 reverse |
|  |  | GmGME-2 | Glyma.19G244700.1 | 376 | Chr19:49190234..49193448 reverse |
|  |  | GmGME-3 | Glyma.20G224400.1 | 376 | Chr20:45903633..45907319 reverse |
|  |  | GmGME-4 | Glyma.10G162000.1 | 376 | Chr10:39625525..39628332 reverse |
|  |  | GmGME-5 | Glyma.03G247000.1 | 379 | Chr03:44384142..44387168 reverse |
|  | Gossypium raimondii | GrGME-1 | Gorai.004G246500.1 | 376 | Chr04:58349574..58352755 reverse |
|  |  | GrGME-2 | Gorai.013G120800.1 | 376 | Chr13:30631595..30634875 forward |
|  | Kalanchoe fedtschenkoi | KfGME | Kaladp0024s0516.1 | 376 | Scaffold_24:4344945..4348134 forward |
|  | Kalanchoe laxiflora | KlGME-1 | Kalax.0708s0008.1 | 376 | scaffold_708:44510..47720 reverse |
|  |  | KlGME-2 | Kalax.0481s0028.1 | 376 | scaffold_481:178986..182336 forward |
|  | Linum usitatissimum | LuGME-1 | Lus10009174 | 376 | scaffold471:26673..28321 forward |
|  |  | LuGME-2 | Lus10001777 | 376 | scaffold1230:27722..29145 reverse |
|  |  | LuGME-3 | Lus10020247 | 376 | scaffold641:58541..59972 forward |
|  |  | LuGME-4 | Lus10015915 | 376 | scaffold201:183945..185577 forward |
|  | Manihot esculenta | MeGME-1 | Manes.09G153400.1 | 376 | Chromosome09:26955144..26958583 forward |
|  |  | MeGME-2 | Manes.08G134400.1 | 376 | Chromosome08:30057259..30060677 reverse |
|  | Medicago truncatula | MtGME-1 | Medtr7g115080.1 | 411 | chr7:47515136..47519250 reverse |
|  |  | MtGME-2 | Medtr1g080950.2 | 416 | chr1:36020421..36023967 forward |
|  | Mimulus guttatus | MgGME-1 | Migut.G00395.1 | 376 | scaffold_7:2513057..2516269 reverse |
|  |  | MgGME-2 | Migut.N00226.1 | 376 | scaffold_14:1056860..1060539 forward |
|  | Phaseolus vulgaris | PhvGME-1 | Phvul.006G101600.1 | 376 | Chr06:21165142..21168097 reverse |
|  |  | PhvGME-2 | Phvul.007G187300.1 | 376 | Chr07:30717747..30721259 reverse |
|  | Populus trichocarpa | PtGME-1 | Potri.013G040600.1 | 375 | Chr13:2821496..2825163 reverse |
|  |  | PtGME-2 | Potri.005G053000.1 | 375 | Chr05:3780098..3783416 reverse |
|  |  | PtGME-3 | Potri.T103900.1 | 375 | scaffold_150:63629..66859 reverse |
|  | Prunus persica | PpGME-1 | Prupe.1G025100.1 | 379 | Pp01:1735713..1739528 reverse |
|  |  | PpGME-2 | Prupe.6G342800.1 | 376 | Pp06:29531300..29535130 reverse |
|  | Ricinus communis | RcGME | 29598.m000469 | 376 | 29598:138318..141290 forward |
|  | Salix purpurea | SpGME-1 | SapurV1A.0110s0130.1 | 375 | chr13:3755832..3759201 reverse |
|  |  | SpGME-2 | SapurV1A.0729s0200.1 | 375 | chr05:2660188..2663062 reverse |
|  |  | SpGME-3 | SapurV1A.1947s0030.1 | 375 | Scaffold1947:25921..29267 reverse |
|  | Theobroma cacao | TcGME | Thecc1EG026351t1 | 426 | scaffold_5:37832297..37836614 forward |
|  | Trifolium pratense | TpGME-1 | Tp57577_TGAC_v2_mRNA13283 | 379 | Tp57577_TGAC_v2_LG3:2009201..2013046 forward |
|  |  | TpGME-2 | Tp57577_TGAC_v2_mRNA7173 | 376 | Tp57577_TGAC_v2_LG6:3787517..3791505 reverse |
| Monocots | Ananas comosus | AncGME-1 | Aco000210.1 | 378 | LG12:3684241..3688461 reverse |
|  |  | AncGME-2 | Aco004224.1 | 378 | LG15:576590..581328 reverse |
|  | Brachypodium distachyon | BdGME-1 | Bradi3g26860.1 | 416 | Bd3:27332429..27335855 forward |
|  |  | BdGME-2 | Bradi4g14950.4 | 371 | Bd4:15616678..15622198 forward |
|  | Brachypodium stacei | BrsGME-1 | Brast03G128500.1 | 378 | Chr03:12393877..12397683 forward |
|  |  | BrsGME-2 | Brast10G139300.1 | 371 | Chr10:14248954..14253644 reverse |
|  | Oropetium thomaeum | OtGME | Oropetium_20150105_20918A | 380 | Oropetium_genomic_20141112_045:210309..214229 reverse |
|  | Oryza sativa | OsGME-1 | LOC_Os10g28200.1 | 378 | Chr10:14640026..14643350 forward |
|  |  | OsGME-2 | LOC_Os11g37890.1 | 371 | Chr11:22459800..22464681 reverse |
|  | Panicum hallii | PhGME-1 | Pahal.H01614.1 | 380 | Chr09:17685910..17689896 reverse |
|  |  | PhGME-2 | Pahal.F01001.1 | 380 | Chr06:3373099..3376999 reverse |
|  |  | PhGME-3 | Pahal.H00540.1 | 371 | Chr08:42243295..42248129 reverse |
|  | Panicum virgatum | PvGME-1 | Pavir.Ib03430.1 | 380 | Chr09b:55036378..55039420 forward |
|  |  | PvGME-2 | Pavir.Ia01927.1 | 447 | Chr09a:26327563..26332840 reverse |
|  |  | PvGME-3 | Pavir.Hb00594.1 | 371 | Chr08b:13503712..13508534 forward |
|  |  | PvGME-4 | Pavir.Fa01938.1 | 380 | Chr06a:44924396..44928156 forward |
|  |  | PvGME-5 | Pavir.Fb00378.1 | 369 | Chr06b:8269421..8271340 reverse |
|  | Setaria italica | SiGME-1 | Seita.9G263600.1 | 380 | scaffold_9:21847034..21850853 forward |
|  |  | SiGME-2 | Seita.6G060000.1 | 380 | scaffold_6:4874581..4877924 forward |
|  |  | SiGME-3 | Seita.8G168800.1 | 371 | scaffold_8:31180672..31185345 reverse |
|  | Setaria viridis | SvGME-1 | Sevir.8G178400.1 | 371 | Chr_08:30918217..30922957 reverse |
|  |  | SvGME-2 | Sevir.9G266500.1 | 380 | Chr_09:22173604..22177545 forward |
|  |  | SvGME-3 | Sevir.6G057600.1 | 380 | Chr_06:4874111..4877453 forward |
|  | Sorghum bicolor | SbGME-1 | Sobic.005G168600.1 | 371 | Chr05:64736442..64740782 reverse |
|  |  | SbGME-2 | Sobic.001G247600.1 | 380 | Chr01:26301630..26305587 reverse |
|  | Spirodela polyrhiza | SppGME | Spipo3G0089800 | 373 | pseudo3:6999685..7001819 forward |
|  | Zea mays | ZmGME-1 | GRMZM2G124434_T01 | 380 | 1:240922200..240925273 forward |
|  |  | ZmGME-2 | GRMZM2G138907_T01 | 371 | 4:9763231..9767123 forward |
| Basal angiosperms | Amborella trichopoda | AmtGME | evm_27.model.AmTr_v1.0_scaffold00002.538 | 378 | AmTr_v1.0_scaffold00002:9178765..9181701 reverse |
| Gymnosperms | Picea sitchensis | PsGME-1 | EF676136.1 | 378 | N/A |
|  |  | PsGME-2 | EF085441.1 | 378 | N/A |
|  |  | PsGME-3 | EF084941.1 | 378 | N/A |
| Lycophytes | Selaginella moellendorffii | SmGME-1 | 406366 | 374 | scaffold_5:1650434..1651768 forward |
|  |  | SmGME-2 | 128181 | 374 | scaffold_89:765149..766487 forward |
|  |  | SmGME-3 | 83391 | 374 | scaffold_5:1614585..1615917 reverse |
|  |  | SmGME-4 | 157784 | 373 | scaffold_71:710728..713495 forward |
|  |  | SmGME-5 | 178232 | 374 | scaffold_44:1553140..1554545 forward374 |
|  |  | SmGME-6 | 76257 | 374 | scaffold_1:5430253..5431586 forward |
|  |  | SmGME-7 | 76013 | 374 | scaffold_1:5395170..5396501 reverse |
|  |  | SmGME-8 | 110416 | 374 | scaffold_40:902195..903534 forward |
|  |  | SmGME-9 | 272082 | 373 | scaffold_99:498712..501468 forward |
| Bryophytes | Marchantia polymorpha | MpGME | Mapoly0101s0064.1 | 379 | scaffold_101:665457..668951 forward |
|  | Physcomitrella patens | PhpGME-1 | Pp3c2_7940V3.1 | 376 | Chr02:5395298..5398478 forward |
|  |  | PhpGME-2 | Pp3c1_30660V3.1 | 376 | Chr01:21789695..21792471 forward |
|  |  | PhpGME-3 | Pp3c17_15920V3.1 | 380 | Chr17:10791520..10794382 forward |
|  | Sphagnum fallax | SfGME-1 | Sphfalx0035s0122.1 | 379 | super_35:1905299..1907812 forward |
|  |  | SfGME-2 | Sphfalx0004s0050.1 | 378 | super_4:790371..793199 forward |
| Chlorophytes | Chlamydomonas reinhardtii | ChrGME | Cre01.g019250.t1.2 | 384 | chromosome_1:3095306..3098524 forward |
|  | Coccomyxa subellipsoidea | CosGME | 52441 | 404 | scaffold_2:2477947..2481512 reverse |
|  | Micromonas pusilla | MipGME | 46037 | 378 | scaffold_17:242301..244210 reverse |
|  | Micromonas sp. RCC299 | MsrGME-1 | 80077 | 379 | Chr_03:1382679..1384042 reverse |
|  |  | MsrGME-2 | 92683 | 378 | Chr_17:80762..82297 forward |
|  | Ostreococcus lucimarinus | OlGME | 18701 | 376 | Chr_17:203570..204939 reverse |
|  | Volvox carteri | VcGME | Vocar.0007s0276.1 | 383 | scaffold_7:2243833..2246722 reverse |
