# Supplementary material for: Molecular Evolution of GDP-D-Mannose Epimerase (GME), a Key Gene in Plant Ascorbic Acid Biosynthesis
Source: Front Plant Sci. 2018 Sep 4;9:1293. doi: 10.3389/fpls.2018.01293 (PMC6132023; doi:10.3389/fpls.2018.01293)
Supplement: Supplementary file 2 [file Table_2.DOCX]

**Supplemental Table 2. Results of positive selection tests in plant GME using site models**

| **Model** | **Np** | **lnL** | **Parameters** | **Models compared** | **d.f.** | **-2ΔlnL** | ***p-*value** | **Positively selected sites (posterior probability)** |
| --- | --- | --- | --- | --- | --- | --- | --- | --- |
| M0 | 221 | -30862.364 | ω=0.02872 |  |  |  |  | none |
| M3 | 225 | -29931.523 | p0=0.562, ω0=0.00108  P1=0.34778, ω1=0.03669  P2=0.09122, ω2=0.18514 | M0 vs. M3 | 4 | 1861.68^***^ | 0.000 | none |
| M1a | 222 | -30665.647 | p_0_=0.96962, ω_0_=0.02349 p_1_=0.03038, ω_1_=1.0000 |  |  |  |  | Not allowed |
| M2a | 224 | -30665.647 | p_0_=0.96962, ω_0_=0.02349; p_1_=0.03038 ω_1_=1.000;  p_2_=0.0, ω_2_=13.419148 | M1a vs. M2a | 2 | 0 | 1.000 | none |
| M7 | 222 | -29899.719 | p=0.20828, q=4.39061 |  |  |  |  | Not allowed |
| M8 | 224 | -29899.723 | p0=0.99999, p=0.20827  q=4.39061  p1=0.00001, ω=2.71684 | M7 vs. M8 | 2 | 0.008 | 0.996 | none |

Np: number of estimated parameters; lnL: log likelihood score; df : degrees of freedom,; -2ΔlnL: twice the log-likelihood difference of the model compared. ^***^Significant at *p* < 0.0001;
