# Supplementary material for: Molecular Evolution of GDP-D-Mannose Epimerase (GME), a Key Gene in Plant Ascorbic Acid Biosynthesis
Source: Front Plant Sci. 2018 Sep 4;9:1293. doi: 10.3389/fpls.2018.01293 (PMC6132023; doi:10.3389/fpls.2018.01293)
Supplement: Supplementary file 3 [file Table_3.DOCX]

**Supplemental Table 3. PAML branch model analysis to test the variable selective pressure among branches**

| Model | Np | lnL | LRT pair | d.f. | -2ΔlnL | *p-*value |
| --- | --- | --- | --- | --- | --- | --- |
| M0 | 221 | -30862.364 |  |  |  |  |
| Mf | 439 | -28263.757 | M0 vs. Mf | 218 | 5197.214^***^ | 0.000 |

Np: number of estimated parameters; lnL: log likelihood score; ΔlnL: log-likelihood difference of the model compared ^; ***^Significant at *p* < 0.0001
