# Supplementary material for: Molecular Evolution of GDP-D-Mannose Epimerase (GME), a Key Gene in Plant Ascorbic Acid Biosynthesis
Source: Front Plant Sci. 2018 Sep 4;9:1293. doi: 10.3389/fpls.2018.01293 (PMC6132023; doi:10.3389/fpls.2018.01293)
Supplement: Supplementary file 4 [file Table_4.DOCX]

**Supplemental Table 4. Branch-site models used to identify branches and sites under episodic positive selection in plant *GME***

| **Foreground branches** | **Model** | **lnL** | **Parameter estimates** | **-2ΔlnL** | **d.f** | ***p-*value** | **Positively Selected sites** |
| --- | --- | --- | --- | --- | --- | --- | --- |
| Arabidopsis thaliana | Model A | -30669.449 | p0 = 0.968, p1 = 0.027, p2a = 0.004, p2b = 0.00012  Background: ω0 = 0.025, ω1 = 1.000, ω2a = 0.025, ω2b = 1.000  Foreground: ω0 = 0.025, ω1 = 1.0000 ω2a =13.991, ω2b = 13.991 | 4.676^*^ | 1 | 0.0306 | 101S (0.981) |
|  | Null model | -30671.787 | p0 = 0.9581, p1 = 0.027, p2a = 0.014, p2b = 0.00041  Background: ω0 = 0.025, ω1 = 1.000, ω2a = 0.025, ω2b = 1.000 Foreground: ω0 = 0.025, ω1 = 1.000, ω2a =1, ω2b = 1 |  |  |  |  |
| Brassica rapa  (*BrGME-1*) | Model A | -30667.853 | p0 = 0.970, p1 = 0.028, p2a = 0.003, p2b = 0.00007  Background: ω0 = 0.025 ω1 = 1.000, ω2a = 0.025, 9ω2b = 1.000  Foreground: ω0 = 0.025, ω1 = 1.000, ω2a =16.747, ω2b = 16.747 | 6.034^*^ | 1 | 0.0140 | 82D (0.997) |
|  | Null model | -30670.870 | p0 = 0.964, p1 = 0.027, p2a = 0.009, p2b = 0.00024  Background: ω0 = 0.025 ω1 = 1.000, ω2a = 0.025, 9ω2b = 1.000  Foreground: ω0 = 0.025, ω1 = 1.000, ω2a =1.000, ω2b = 1.000 |  |  |  |  |
| Brassica rapa  (*BrGME-2*) | Model A | -30665.037 | p0 = 0.967, p1 = 0.027, p2a = 0.005, p2b = 0.00016  Background: ω0 = 0.025, ω1 = 1.000, ω2a = 0.025, ω2b = 1.000  Foreground: ω0 = 0.025, ω1 = 1.000, ω2a =13.90, ω2b = 13.90 | 5.698^*^ | 1 | 0.0170 | 9D (0.996); 17K (0.969) |
|  | Null model | -30667.886 | p0 = 0.945, p1 = 0.026, p2a = 0.027, p2b = 0.00076  Background: ω0 = 0.025, ω1 = 1.000, ω2a = 0.025, ω2b = 1.000  Foreground: ω0 = 0.025, ω1 = 1.000, ω2a =1, ω2b = 1 |  |  |  |  |
| Medicago truncatula  (*MtGME-2*) | Model A | -30667.502 | p0 = 0.970, p1 = 0.026, p2a = 0.003, p2b = 0.00009  Background: ω0 = 0.025, ω1 = 1.000, ω2a = 0.025 ω2b = 1.000  Foreground: ω0 = 0.025, ω1 = 1.000, ω2a = 26.993., ω2b = 36.993 | 6.906^**^ | 1 | 0.0086 | 344G (0.990) |
|  | Null model | -30670.955 | p0 = 0.942, p1 = 0.026, p2a = 0.032, p2b = 0.001  Background: ω0 = 0.025, ω1 = 1.000, ω2a = 0.025 ω2b = 1.000  Foreground: ω0 = 0.025, ω1 = 1.000, ω2a =1., ω2b = 1 |  |  |  |  |
| Marchantia polymorhpa | Modle A | -30662.509 | p0 = 0.946, p1 = 0.028, p2a = 0.026, p2b = 0.00075  Background: ω0 = 0.025, ω1 = 1.000, ω2a = 0.025, ω2b = 1.000  Foreground: ω0 = 0.025, ω1 = 1.000, ω2a = 7.958, ω2b = 7.958 | 5.956^*^ | 1 | 0.0147 | 252S (0.960); 325L (0.983) |
|  | Null model | -30665.487 | p0 = 0.935, p1 = 0.027, p2a = 0.036, p2b = 0.001  Background: ω0 = 0.025, ω1 = 1.000, ω2a = 0.025, ω2b = 1.000  Foreground: ω0 = 0.025, ω1 = 1.000, ω2a =1, ω2b = 1 |  |  |  |  |
| Micromonas pusilla | Model A | -30659.334 | p0 = 0.952, p1 = 0.027, p2a = 0.020, p2b = 0.00057  Background: ω0 = 0.025, ω1 = 1.000, ω2a = 0.025 ω2b = 1.000  Foreground: ω0 = 0.025, ω1 = 1.000, ω2a =6.142., ω2b = 6.142 | 8.714^**^ | 1 | 0.0032 | 101S (0.981); 228K (0.997);  252S (0.980); 312R (0.969) |
|  | Null model | -30663.691 | p0 = 0.937, p1 = 0.027, p2a = 0.035, p2b = 0.001  Background: ω0 = 0.025, ω1 = 1.000, ω2a = 0.025, ω2b = 1.000  Foreground: ω0 = 0.025, ω1 = 1.000, ω2a =1., ω2b = 1 |  |  |  |  |
| Ostreococcus lucimarinus | Model A | -30642.075 | p0 = 0.904, p1 = 0.027, p2a = 0.067 p2b = 0.002  Background: ω0 = 0.025, ω1 = 1.000, ω2a = 0.025 ω2b = 1.000  Foreground: ω0 = 0.025, ω1 = 1.000, ω2a =11.848., ω2b = 11.848 | 19.104^***^ | 1 | 0.0000 | 57T (0.964); 121A(0.967);  218 T (0.986);249 S (0.980);  264 S (0.973);274 S( 0.952);  325 L (0.982) |
|  | Null model | -30651.627 | p0 = 0.901, p1 = 0.027, p2a = 0.070, p2b = 0.002  Background: ω0 = 0.025, ω1 = 1.000, ω2a = 0.025; ω2b = 1.000  Foreground: ω0 = 0.025, ω1 = 1.000, ω2a =1., ω2b = 1 |  |  |  |  |
| Volvox carteri | Model A | -30644.191 | p0 = 0.92046, p1 = 0.028, p2a = 0.050, p2b = 0.002  Background: ω0 = 0.025, ω1 = 1.000, ω2a = 0.025 ω2b = 1.000  Foreground: ω0 = 0.025, ω1 = 1.000, ω2a = 12.367, ω2b = 12.367 | 19.816^***^ | 1 | 0.0000 | 124N (0.974); 184C (0.985);  232S (0.951); 314K (0.986);  322Y (0.992) |
|  | Null model | -30654.099 | p0 = 0.908, p1 = 0.027, p2a = 0.063, p2b = 0.002  Background: ω0 = 0.025, ω1 = 1.000, ω2a = 0.025; ω2b = 1.000  Foreground: ω0 = 0.025, ω1 = 1.000, ω2a =1., ω2b = 1 |  |  |  |  |
| Chlamydomonas reinhardt | Model A | -30637.566 | p0 = 0.918, p1 = 0.028, p2a = 0.052, p2b = 0.0016  Background: ω0 = 0.025, ω1 = 1.000, ω2a = 0.025, ω2b = 1.000  Foreground: ω0 = 0.025, ω1 = 1.000, ω2a =35.374, ω2b = 35.374 | 31.582^***^ | 1 | 0.0000 | 17K (0.998); 80T (0.987);  146V (0.993); 296S (0.977);  319I (0.991) |
|  | Null model | -30653.357 | p0 = 0.904, p1 = 0.027, p2a = 0.067, p2b = 0.002  Background: ω0 = 0.025, ω1 = 1.000, ω2a = 0.025, ω2b = 1.000  Foreground: ω0 = 0.025, ω1 = 1.000, ω2a =1, ω2b = 1 |  |  |  |  |
| Coccomyxa subellipsoidea | Model A | -30614.617 | p0 = 0.819, p1 = 0.030, p2a = 0.145, p2b = 0.005  Background: ω0 = 0.024, ω1 = 1.000, ω2a = 0.024, ω2b = 1.000  Foreground: ω0 = 0.024, ω1 = 1.000, ω2a = 999.0, ω2b = 999.0 | 51.482^***^ | 1 | 0.0000 | 1G (0.985); 47A (0.995); 48S (0.952);  54E(0.999);55H(0.999);120A(0.953); 152D(0.994);173C(0.989); 218T(0.970);  228K(0.998) 251Y(0.960);271I(0.952);  272V(0.956); 273L(0.973) ;305L (0.976) |
|  | Null model | -30640.358 | p0 = 0.857, p1 = 0.028, p2a = 0.112, p2b = 0.004  Background: ω0 = 0.024, ω1 = 1.000, ω2a = 0.024 ω2b = 1.000  Foreground: ω0 = 0.024, ω1 = 1.000, ω2a =1, ω2b = 1 |  |  |  |  |

lnL: log likelihood score; df : degrees of freedom,; -2ΔlnL: twice the log-likelihood difference of the model compared.

^*^significant at p <0.05; ^**^significant at p<0.01; ^***^Significant at *p* < 0.0001;
