# Supplementary material for: Molecular Evolution of GDP-D-Mannose Epimerase (GME), a Key Gene in Plant Ascorbic Acid Biosynthesis
Source: Front Plant Sci. 2018 Sep 4;9:1293. doi: 10.3389/fpls.2018.01293 (PMC6132023; doi:10.3389/fpls.2018.01293)
Supplement: Supplementary file 5 [file Table_5.DOCX]

**Supplemental Table 5. Results of positive selection tests using methods of SLAC, FEL, FUBAR, and MEME**

| **Mean *d*_N_/*d*_S_** | **Positive selection sites** | | | |
| --- | --- | --- | --- | --- |
|  | **SLAC** | **FEL** | **FUBAR** | **MEME^a^ (*p*-value)** |
| 0.069 | None | None | None | 56 (0.023); 60 (0.014)  101 (0.022); 228 (0.089)  262 (0.006); 264 (0.023)  307 (0.021); 338 (0.064)  343 (0.042); 344 (0.000)  360 (0.060) |

^a^ Codons with p-values < 0.1
