# Supplementary material for: Molecular Evolution of GDP-D-Mannose Epimerase (GME), a Key Gene in Plant Ascorbic Acid Biosynthesis
Source: Front Plant Sci. 2018 Sep 4;9:1293. doi: 10.3389/fpls.2018.01293 (PMC6132023; doi:10.3389/fpls.2018.01293)
Supplement: Supplementary file 6 [file Table_6.DOCX]

**Supplemental Data 1. Alignment of plant GME coding sequences used in this study**

>AcGME

ATGGGG---AGTACT------GATGCCGCCACC---AAGTAT------------------GGGGAGTACACTTAC---GAGCAATTGGAGAGGGAGCTTTACTGGCCATCTGAGAAGCTCCGCATCTCTATTACTGGTGCTGGTGGGTTCATAGCATCCCACATTGCTCGAAGACTGAAGACCGAAGGACACTACATCATTGCTTCTGACTGGAAGAAGAATGAGCATATGCCCGAGGACATGTTTTGTCATGAGTTTCACCTTGCAGATCTTAGGGTCATGGATAACTGTATGAAGGTCACCTCTGGGGTGGATCATGTTTTTAACCTTGCTGCTGATATGGGTGGAATGGGGTTTATCCAATCGAACCACTCAGTCATCATGTATAACAACACTATGATCAGTTTCAACATGCTTGAGGCTGCTAGGATTAATGGTGTCAAGAGGTTTTTCTATGCATCCAGTGCGTGTATCTACCCTGAATTCAAGCAATTGGAAACCTCTAAT------GTT---------AGTTTGAAGGAGGCTGACGCTTGGCCAGCAGAGCCTCAAGATGCATATGGTCTCGAAAAGCTTGCAACAGAGGAATTATGTAAGCACTACACCAAAGACTTTGGAATTGAGTGTCGTATTGGACGATTCCACAACATTTATGGCCCCTTTGGGACATGGAAAGGTGGAAGGGAGAAAGCCCCTGCTGCTTTTTGTAGGAAGGCCCTCACTGCCATTGACAAGTTTGAGATGTGGGGGGATGGACTGCAGACCAGGTCCTTCACTTTTATTGATGAATGCGTTGAAGGTGTCCTGAGATTAACAAAGTCAGACTTCCGGGAGCCTGTGAATATTGGAAGTGATGAGATGGTCAGTATGAATGAGATGGCTGAGATTGTGCTCGGCTTTGAGGACAAG---AAGCTTCCTATCCAACATATTCCTGGTCCCGAGGGTGTGCGAGGTCGCAACTCTGACAACACTTTGATAAAAGAGAAGCTTGGTTGGGCCCCAACTATGAGATTGAAGGATGGACTGAGGATCACATACTTTTGGATCAAGGAACAAATTGAAAAAGAAAAGACTCGTGGCATT---GATCTCTCA---GTTTATGGATCTTCAAAAGTTGTTGGAACGCAGGCACCTGTCCAGCTGGGCTCACTTCGTGCGGCTGATGGCAAAGAG---------------------------------------------------------------------------

>AdGME

ATGGGA---AGCACC------AGT---GAATCT---AACTAC------------------GGATCGTACACCTAT---GAGAATCTCGAGAGGGAACCCTACTGGCCGGAGGCGAAGCTCCGCATCTCCATTACTGGAGCCGGTGGGTTCATTGCCTCGCACATTGCAAGGCGACTGAAGGGCGAGGGGCATTACATCATTGCTTCTGACTGGAAGAAAAACGAGCACATGACCGAGGACATGTTTTGTCACGAGTTCCATCTCGTTGATCTTAGGGTGATGGACAACTGCTTGAAAGTCACGACCGGAGTCGATCATGTGTTCAATCTTGCTGCTGATATGGGTGGTATGGGATTCATTCAGTCCAACCACTCGGTCATTATGTATAACAACACAATGATCAGCTTCAACATGCTTGAAGCAGCTAGGGTCAATGGTGTTAAGAGGTTCTTTTATGCTTCTAGCGCTTGTATTTATCCTGAATTTAAGCAATTGGACACT---AAT------GTG---------AGCTTGAAGGAGTCTGATGCTTGGCCCGCTGAGCCTCAAGATGCTTATGGTTTAGAGAAGCTTGCAACCGAGGAATTATGCAAGCACTACACCAAGGACTTTGGCATTGAATGTAGGATTGGAAGATTTCATAACATTTATGGACCTTTTGGCACATGGAAAGGTGGAAGGGAGAAAGCCCCTGCTGCATTCTGCAGAAAGACCCTTACCTCCACTGATAGGTTTGAGATGTGGGGAGACGGTCTGCAAACCCGATCTTTCACCTTCATTGATGAATGTGTCGAAGGTGTCCTAAGATTGACGAAGTCAGACTTCAGAGAACCAGTGAATATCGGAAGTGATGAGATGGTCAGCATGAATGAGATGGCCGAGATCGTTCTCAGCTTCGAGAACAAG---AAGCTGCCCATCCATCACATTCCAGGCCCAGAGGGCGTCCGTGGACGAAACTCGGACAACACCCTGATTAAGGAGAAGCTTGGGTGGGCCCCAACTATGAAACTGAAGGATGGGCTGAGATTCACATACTTCTGGATAAAGGAGCAACTTGAGAAAGAGAAGGCTCAGGGCATC---GATCTGTCA---ACTTATGGATCGTCAAAAGTTGTGGGAACGCAAGCCCCGGTTCAGTTGGGCTCTCTTCGTGCTGCTGATGGCAAAGAA---------------------------------------------------------------------------

>AeGME

ATGGGA---AGCACC------AGT---GAATCT---AACTAC------------------GGATCGTACACCTAT---GAGAACCCCGAGAGGGAACCCTACTGGCCGGAGGCGAAGCTCCGCATCTCCATTACTGGAGCCGGTGGGTTCATTGCCTCGCACATTGCAAGGCGACTGAAGGGCGAGGGGCATTACATCATTGCTTCTGACTGGAAGAAAAACGAGCACATGACCGAGGACATGTTTTGTCACGAGTTCCATCTCGTTGATCTCAGGGTGATGGACAACTGCTTGAAAGTCACGACCGGAGTCGATCATGTGTTCAATCTTGCTGCTGACATGGGTGGCATGGGATTCATTCAGTCCAACCACTCGGTCATTATGTATAACAACACAATGATCAGCTTCAACATGCTTGAAGCAGCTAGGGTCAATGGTGTTAAGAGGTTCTTTTACGCTTCTAGCGCTTGTATTTATCCTGAATTTAAGCAGTTGGACACT---AAT------GTG---------AGCTTGAAGGAGTCTGATGCTTGGCCCGCTGAGCCTCAAGATGCTTATGGTTTAGAGAAGCTTGCAACCGAGGAATTATGCAAGCACTACACCAAGGACTTTGGCATTGAATGTAGGATTGGAAGGTTTCATAACATTTATGGACCTTTTGGCACATGGAAAGGTGGAAGGGAGAAAGCCCCTGCTGCATTCTGCAGAAAGACCCTTACCTCCACTGATAGGTTTGAGATGTGGGGAGACGGTCTGCAAACCCGATCTTTCACCTTCATTGATGAATGTGTCGAAGGTGTCCTAAGATTGACAAAGTCAGACTTTAGAGAACCGGTGAATATCGGAAGCGATGAGATGGTCAGCATGAACGAGATGGCCGAGATCGTTCTCAGCTTCGAGAACAAG---AAGCTGCCCATCCATCACATTCCTGGCCCAGAGGGGGTCCGTGGTCGAAACTCGGACAACCCCCTGATTAAGGAGAAGCTTGGGTGGGCCCCAACTATGAAACTGAAGGATGGGCTGAGATTCACATACTTCTGGATCAAGGAGCAACTTGAGAAAGAGAAGGCTCAGGGCATC---GATCTGTCA---ACTTATGGATCGTCAAAAGTTGTGGGAACGCAAGCCCCGGTTCAGTTGGGCTCTCTTCGTGCTGCTGATGGCAAAGAA---------------------------------------------------------------------------

>AhGME

ATGGGA---ACTACC------AAT---GGAAGT---GATTAT------------------GGTGCTTACACATAC---AAGGAGCTAGAAAGAGAGCCTTATTGGCCATCTGAGAAGCTCAAGATATCGATTACCGGCGCTGGAGGTTTCATTGCGTCTCACATTGCTCGTCGTTTGAAGCACGAAGGTCATTACGTGATTGCTTCTGACTGGAAGAAGAATGAGCATATGACTGAAGACATGTTCTGTGATGAATTCCATCTTGTTGATCTTAGGGTTATGGAGAATTGTCTCAAGGTTACTAAAGGAGTTGATCATGTTTTTAACTTAGCTGCTGATATGGGTGGTATGGGTTTCATTCAGTCTAACCATTCGGTGATCATGTATAACAATACTATGATTAGTTTCAATATGATCGAGGCGGCTAGGATCAATGGGATTAAGAGGTTCTTCTATGCTTCTAGTGCTTGTATTTACCCGGAGTTCAAGCAGTTGGAGACTACTAAT------GTG---------AGCCTGAAGGAGTCAGATGCTTGGCCTGCAGAGCCTCAAGATGCTTATGGTTTGGAGAAACTTGCAACGGAGGAGTTGTGCAAGCATTACAACAAAGATTTTGGAATTGAGTGCCGCATTGGAAGGTTCCATAACATTTATGGTCCATTTGGAACATGGAAAGGTGGAAGGGAGAAGGCTCCAGCTGCTTTCTGTAGAAAGGCTCTCACCTCCACAGATAGGTTTGAGATGTGGGGAGACGGGCTACAAACCCGTTCTTTTACCTTCATCGATGAGTGCGTTGAAGGTGTACTCAGGTTGACAAAATCTGATTTCCGTGAGCCGGTGAACATTGGAAGCGATGAGATGGTGAGCATGAATGAGATGGCTGAGATGGTTCTCAGCTTTGAGGAGAAG---AAGCTTCCAATTCACCACATTCCAGGCCCAGAAGGTGTTCGTGGTCGTAACTCAGACAACAATCTGATCAAAGAAAAGCTTGGTTGGGCTCCAAATATGAGATTGAAGGAGGGGCTTAGAATAACCTACTTCTGGATAAAGGAACAGATCGAGAAAGAGAAAGCAAAAGGAAGC---GATGTGTCC---CTTTACGGGTCATCAAAGGTGGTTGGAACTCAAGCGCCGGTTCAGCTAGGCTCACTCCGCGCAGCTGATGGAAAAGAG---------------------------------------------------------------------------

>AlGME

ATGGGA---ACTACA------AAT---GGAAGT---GATTAT------------------GGTGCTTACACTTAC---AAGGAGCTAGAAAGAGAGCCTTATTGGCCATCTGAGAAGCTCAAGATATCGATTACCGGCGCTGGAGGTTTCATTGCTTCGCACATTGCTCGTCGTCTGAAGCACGAAGGTCATTACGTGATTGCTTCTGACTGGAAGAAGAATGAGCATATGACTGAAGACATGTTCTGTGATGAATTCCACCTTGTTGATCTTAGGGTTATGGAGAATTGTCTCAAGGTTACTAAAGGAGTTGATCATGTTTTTAACTTAGCTGCTGATATGGGTGGTATGGGTTTCATTCAGTCTAACCATTCGGTGATCATGTATAACAATACTATGATTAGTTTCAATATGATCGAGGCGGCTAGGATCAATGGGATTAAGAGGTTCTTCTATGCTTCTAGTGCTTGTATTTACCCGGAGTTCAAGCAGTTGGAGACTACTAAT------GTG---------AGCTTGAAGGAGTCAGATGCTTGGCCTGCAGAGCCTCAAGATGCTTATGGTTTGGAGAAACTTGCAACAGAGGAGTTGTGCAAGCATTACAACAAAGATTTTGGAATTGAGTGCCGCATTGGAAGGTTCCATAACATTTATGGTCCTTTTGGAACATGGAAAGGTGGAAGGGAGAAGGCTCCAGCTGCTTTCTGTAGAAAGGCTCTCACCTCCACAGATAGGTTTGAGATGTGGGGAGACGGGCTTCAAACCCGCTCTTTTACCTTCATTGATGAGTGCGTTGAAGGTGTACTCAGGTTGACAAAATCTGATTTCCGTGAGCCGGTGAACATTGGAAGCGATGAGATGGTGAGCATGAATGAGATGGCTGAGATGGTTCTCAGCTTTGAGGAGAAG---AAGCTTCCAATTCACCACATTCCAGGCCCAGAAGGTGTTCGTGGTCGTAACTCAGACAACAATCTGATCAAAGAAAAGCTTGGTTGGGCTCCAAATATGAGATTGAAGGAGGGGCTTAGAATAACCTACTTCTGGATAAAGGAACAGATCGAGAAAGAGAAAGCAAAAGGAAGT---GATGTATCC---CTTTACGGGTCATCAAAGGTGGTTGGAACTCAAGCACCGGTTCAGCTAGGCTCACTCCGCGCGGCTGATGGAAAAGAG---------------------------------------------------------------------------

>AmhGME

ATGGGT---AGTAAT------GAT---GGATTT---GACTAT------------------GGTGCTTACACCTAT---GACAAGCTCGAGAGGGAGCCATACTGGCCATCGGAAAAATTGAGGATATCAATCACTGGAGCGGGTGGTTTCATTGGATCTCACATTGCTCGGCGTTTGAAAAGTGAAGGGCACTATGTTATAGCTTCAGATTGGAAGAAGAATGAGCACATGACTGAGGATATGTTTTGTAATGAATTCCATCTTGTTGATCTTAGGGTGATGGACAATTGTTTGACAGTGACTAAAGACGTCGATCATGTGTTTAACCTTGCTGCCGATATGGGTGGCATGGGTTTCATTCAATCTAATCACTCTGTCATCATGTATAACAATACAATGATCAGTTTCAATATGCTCGAGGCAGCCCGGATTAACGGTGTCAAAAGGTTCTTCTATGCCTCGAGTGCGTGCATTTACCCTGAATTCAAGCAACTGGAAACTGCCAAT------GTT---------AGTCTTAAGGAGGCTGATGCTTGGCCAGCAGAGCCACAAGATGCTTATGGCTTAGAAAAGCTAGCTACTGAGGAGCTTTGCAAACATTACACCAAAGATTTTGGAATTGAATGCAGAATTGGAAGGTTCCACAACATTTATGGTCCTTTCGGAACCTGGAAAGGTGGTCGGGAGAAGGCTCCGGCTGCCTTCTGCAGGAAGGCGATCACTTCTACTGATAAGTTTGAGATGTGGGGGGATGGGTTGCAGACTCGATCTTTCACATTCATTGATGAATGTGTGGAAGGTGTACTTCGATTGACCAAGTCAGACTTCCGGGAGCCGGTGAACATTGGGAGTGATGAAATGGTTAGCATGAACGAAATGGCTGAGATTGTTTTAAGCTTCGAGGATCGT---AAGCTTCCGATCCATCATATTCCCGGTCCAGAGGGAGTTCGTGGCCGTAATTCAGATAACACATTGATTAAGGAGAAACTTGGCTGGGCTCCAACCATGAGGCTAAAGGATGGGCTAAGGATTACATACTTTTGGATCAAAGAGCAGATCGAAAAGGAAAAGTCTCAAGGTGTG---GACCTGTCG---GTTTATGGATCGTCTAAGGTGGTCACCACCCAAGCTCCGGTTCAGCTTGGTTCTTTACGCGCTGCCGATGGCAAGGAA---------------------------------------------------------------------------

>AmtGME

ATGGGG---AGTGCTGGAAAGGAA---GCCACC---AGCTAT------------------GGCGAATACACTTAC---GCGAATTTAGAGAGAGAATCATACTGGCCCTCTGAGAAACTGAGGATCTCCATCACTGGAGCTGGAGGATTCATAGCCTCTCACATAGCCCGAAGGCTTAAGAGTGAGGGCCATTACATCATAGCTTCAGATTGGAAGAAGAATGAGCACATGCCCGAGGACATGTTTTGCCATGAGTTTCACTTGGTAGACCTAAGGGTCATGGATAATTGCCTTAAGGTCACTACTGGTGTGGACCATGTCTTCAATTTGGCTGCTGACATGGGAGGAATGGGTTTTATACAGTCGAACCATTCTGTTATTATGTACAATAATACCATGATCAGTTTCAATATGCTCGAGGCAGCGAGGATTAATGGAGTTAAAAGGTTTTTCTATGCTTCTAGTGCTTGCATCTACCCTGAGTTCAAGCAGCTGGAGACA---AAT------GTT---------AGCTTGAAGGAATCTGATGCTTGGCCTGCTGAGCCTCAAGATGCATATGGGCTGGAGAAGCTTGCAACTGAGGAGCTGTGCAAACACTACACGAAGGACTTTGGAATCGAGTGCCGTGTTGGACGTTTCCACAACATTTATGGTCCATTTGGAACATGGAAAGGAGGGAGAGAAAAGGCACCAGCTGCCTTTTGTAGGAAGGCAATCACTTCCACCGATAAGTTTGAGATGTGGGGCGATGGGCTTCAAACTCGTTCTTTCACATTCATTGATGAATGTGTTGAAGGCGTCCTAAGGTTGACAAAGTCTGATTTTAGGGAGCCAGTGAACATTGGGAGCGATGAGATGGTGAGCATGAATGAGATGGCTGAAATTGTGCTTAGTTTTGAGGACAAG---AAGCTCCCCATCCACCACATTCCAGGTCCCGAGGGTGTGAGGGGACGCAACTCTGATAATACTCTTATCAAGGAGAAGCTGGGATGGGCACCCACCATGAAATTGAAGGATGGTTTGAGGTTCACATACTTTTGGATAAAGGATCAGATTGAGAAAGAGAAAGCTCAGGGGATT---GACCTTTCA---GTTTATGGGTCTTCGAAAGTGGTCGGGACTCAGGCCCCAGTTCAACTTGGATCACTGAGAGCGGCTGATGGGAAGGAA---------------------------------------------------------------------------

>AncGME-1

ATGGGG---GGCGCGGAAAATAAC---GGAACC---AACTAC------------------GGCGAGTACACCTAC---GCAGAGCTAGAGAGGGAGCCCTACTGGCCATCAGAGAAGCTGAGAATTTCCATAACAGGCGCGGGTGGATTCATCGCGTCCCACATTGCGAGGCGTTTGAAGAGTGAAGGGCACTACATCATCGCTTCCGACTGGAAGAAGAATGAGCATATGGATGAAGACATGTTCTGCCACGAATTCCACTTGGCGGATCTCAGGGTGATGGACAATTGCCTGAAGGTCACTGACGGTGTGGACCATGTTTTCAACCTCGCGGCCGATATGGGCGGGATGGGTTTTATCCAGTCAAACCATTCGGTGATTATGTACAACAACACCATGATCAGTTTCAACATGCTCGAAGCTGCGAGGATCAACGGCGTGAAGAGGCTTTTCTATGCCTCGAGTGCCTGTATTTACCCTGAATTCAAGCAATTGGAGACC---AAT------GTG---------AGCTTGAAGGAATCTGATGCTTGGCCCGCCGAGCCTCAAGATGCTTATGGCCTGGAGAAGCTCGCAACTGAGGAACTGTGCAAGCACTACAACAAGGACTTTGGCATCGAGTGCCGCGTTGGGCGTTTCCACAACATTTATGGCCCATTCGGAACATGGAAAGGTGGGAGGGAGAAAGCTCCTGCTGCTTTCTGCAGAAAAGCTCTCACTTCTACTGACAGGTTTGAGATGTGGGGTGACGGACTGCAGACTCGATCCTTCACTTTCATCGACGAGTGCGTCGAAGGCGTCCTGAGGTTGACGAAATCGGATTTCCGAGAGCCGGTGAACATCGGAAGCGACGAAATGGTGAGCATGAATGAGATGGCTGAAATAGTTCTCAGCTTCGAGAACAAG---CAGCTTCCCATCCATCACATCCCCGGCCCTGAGGGCGTCCGAGGCCGCAACTCCGACAACACCCTAATCAAAGAGAAACTTGGTTGGGCCCCGACGATGAAACTGAGAGATGGGCTAAGGTTCACATACTTCTGGATCAAAGAACAAATCGAGAAGGAGAAAGGTCAGGGGCTC---GACATCTCC---GTCTACGGCTCGTCCAAGGTGGTGCAAACCCAAGCGCCGGTCCAGCTGGGCTCGCTTCGCGCCGCAGATGGAAAAGAG---------------------------------------------------------------------------

>AncGME-2

ATGGGA---AGCACTGGAAACGAA---GGAACG---ATCTAT------------------GGCGAGTACACTTAT---GCGGAGTTGGAGAGGGAGCCTTACTGGCCCACTGAGAAGTTGCGGATCTCTGTGACGGGAGCCGGAGGCTTTATCGGGTCTCACATTGCGAGGCGGTTGAAGAGTGAGGGGCATTACATCATTGCCTCGGACTGGAAGAAGAACGAACACATGACCGAGGATATGTTCTGCCACGAGTTCCACCTCGTCGATCTCAGGGTGATGGACAACTGCTTGAAGGTGACCACAGGAGTAGACCATGTTTTCAATCTTGCGGCCGATATGGGTGGGATGGGATTTATTCAGTCCAATCACTCTGTGATCATGTACAACAATACTATGATCAGTTTCAACATGCTCGAAGCGGCAAGGATTAATAGCGTGAAAAGGTTCTTCTATGCATCGAGTGCCTGTATCTACCCTGAATTCAAGCAATTGGACACT---AAT------GTG---------AGCTTGAAGGAATCCGATGCTTGGCCTGCGGAGCCCCAAGATGCGTATGGCTTGGAGAAGCTCGCGACGGAGGAATTGTGCAAGCACTACACCAAGGACTTTGGGATTGAGTGTCGGATTGGGCGCTTCCATAACATTTATGGCCCTTTTGGAACATGGAAAGGTGGTAGGGAGAAGGCGCCTGCTGCCTTCTGTAGAAAGACTCTCACTTCCACTGATAGGTTTGAAATGTGGGGAGATGGGCTGCAGACTCGATCCTTTACTTTCATTGATGAGTGCGTTGAAGGTGTCCTGAGATTGACGAAGTCGGATTTCCGAGAGCCAGTAAACATTGGAAGTGATGAAATGGTCAGCATGAATGAGATGGCTGAAATCGTTCTCAGCTTTGAGGACAAG---AAGCTGCCCATCCACCACATTCCTGGTCCAGAGGGTGTTCGTGGCCGCAACTCTGACAACACCCTTATAAAAGAGAAACTCGGGTGGGCCCCATCAATGAGACTCAGGGACGGCCTGAGGATCACATACTTCTGGATCAAGGAACAACTTGAGAAGGAGAAAGTCGCGGGAGTT---GATCTGTCC---TTATATGGCTCATCCAAGGTGGTGCAGACACAAGCACCGGTTCAGTTGGGCTCACTCCGTGCGGCAGATGGAAACGAA---------------------------------------------------------------------------

>ArGME

ATGGGA---AGCACC------AGT---GAATCT---AACTAC------------------GGATCGTACACCTAT---GAGAACCTCGAGAGGGAACCCTACTGGCCGGAGGCGAAGCTCCGCATCTCCATTACCGGAGCCGGTGGGTTCATTGCCTCGCACATTGCAAGGCGACTGAAGGGAGAGGGGCATTACATCATTGCTTCTGACTGGAAGAAAAACGAGCACATGACCGAGGACATGTTTTGTCACGAGTTCCATCTCGTTGATCTCAGGGTGATGGACAACTGCTTGAAAGTCACTACCGGAGTCGATCATGTGTTCAATCTTGCTGCTGATATGGGTGGTATGGGATTCATTCAGTCCAACCACTCGGTCATAATGTATAACAACACAATGATCAGCTTCAACATGCTTGAAGCAGCTAGGGTCAATGGTGTTAAGAGATTCTTTTATGCTTCTAGCGCTTGTATTTATCCTGAATTTAAGCAGTTGGACACT---AAT------GTG---------AGCTTGAAGGAGTCTGATGCTTGGCCCGCTGAGCCTCAAGATGCTTATGGTTTAGAGAAGCTTGCAACCGAGGAATTATGCAAGCACTACACCAAGGACTTTGGCATTGAATGTAGGATTGGAAGGTTTCATAACATTTATGGACCTTTTGGCACATGGAAAGGTGGAAGGGAGAAAGCCCCTGCTGCATTCTGCAGAAAGACCCTTACCTCCACTGATAGGTTTGAGATGTGGGGAGACGGTCTGCAAACCCGATCTTTCACCTTCATTGATGAATGTGTCGAAGGTGTCCTAAGATTGACGAAGTCAGACTTCAGAGAACCAGTGAATATCGGAAGTGATGAGATGGTCAGCATGAATGAGATGGCCGAGATCGTTCTCAGCTTCGAGAACAAG---AAGCTGCCCATCCATCACATTCCTGGCCCAGAGGGGGTCCGTGGTCGAAACTCGGACAACACCCTGATTAAGGAGAAGCTTGGGTGGGCCCCAACTATGAAACTGAAGAATGGGCTGAGATTCACATACTTCTGGATCAAGGAGCAACTTGAGAAAGAGAAGGCTCAGGGCATC---GATCTGTCG---ACTTATGGGTCATCAAAAGTTGTGGGAACGCAAGCCCCGGTTCAGTTGGGCTCTCTTCGTGCTGCTGATGGCAAAGAA---------------------------------------------------------------------------

>AtGME

ATGGGA---ACTACC------AAT---GGAACA---GACTAT------------------GGAGCATACACATAC---AAGGAGCTAGAAAGAGAGCAATATTGGCCATCTGAGAATCTCAAGATATCAATAACAGGAGCTGGAGGTTTCATTGCATCTCACATTGCTCGTCGTTTGAAGCACGAAGGTCATTACGTGATTGCTTCTGACTGGAAAAAGAATGAACACATGACTGAAGACATGTTCTGTGATGAGTTCCATCTTGTTGATCTTAGGGTTATGGAGAATTGTCTCAAAGTTACTGAAGGAGTTGATCATGTTTTTAACTTAGCTGCTGATATGGGTGGTATGGGTTTTATCCAGAGTAATCACTCTGTGATTATGTATAATAATACTATGATTAGTTTCAATATGATTGAGGCTGCTAGGATCAATGGGATTAAGAGGTTCTTTTATGCTTCGAGTGCTTGTATCTATCCAGAGTTTAAGCAGTTGGAGACTACTAAT------GTG---------AGCTTGAAGGAGTCAGATGCTTGGCCTGCAGAGCCTCAAGATGCTTATGGTTTGGAGAAGCTTGCTACGGAGGAGTTGTGTAAGCATTACAACAAAGATTTTGGTATTGAGTGTCGAATTGGAAGGTTCCATAACATTTATGGTCCTTTTGGAACATGGAAAGGTGGAAGGGAGAAGGCTCCAGCTGCTTTCTGTAGGAAGGCTCAGACTTCCACTGATAGGTTTGAGATGTGGGGAGATGGGCTTCAGACCCGTTCTTTTACCTTTATCGATGAGTGTGTTGAAGGTGTACTCAGGTTGACAAAATCAGATTTCCGTGAGCCGGTGAACATCGGAAGCGATGAGATGGTGAGCATGAATGAGATGGCTGAGATGGTTCTCAGCTTTGAGGAAAAG---AAGCTTCCAATTCACCACATTCCTGGCCCGGAAGGTGTTCGTGGTCGTAACTCAGACAACAATCTGATCAAAGAAAAGCTTGGTTGGGCTCCTAATATGAGATTGAAGGAGGGGCTTAGAATAACCTACTTCTGGATAAAGGAACAGATCGAGAAAGAGAAAGCAAAGGGAAGC---GATGTGTCG---CTTTACGGGTCATCAAAGGTGGTTGGAACTCAAGCACCGGTTCAGCTAGGCTCACTCCGCGCGGCTGATGGAAAAGAG---------------------------------------------------------------------------

>BdGME-1

ATGGGG---AGCACCGACAAGACC---GGAACT---CCTTAT------------------GGTGAGTACACATAT---GCTGAGCTGGAGAGGGAACTATACTGGCCATCTGAGAAGCTGAGGATTTCGATCACTGGAGCTGGTGGTTTCATTGGATCCCATATTGCTCGTCGTCTGAAGAGTGAGGGACACTACATCATTGCCTCTGACTGGAAGAAGAACGAGCATATGACTGAGGACATGTTCTGCCATGAGTTCCACCTTGTTGACCTCAGGGTCATGGATAACTGCCTTAAGGTCACCAATGGTGTGGACCATGTCTTCAATCTTGCTGCTGATATGGGTGGCATGGGTTTCATTCAGTCCAACCACTCCGTTATCATGTACAACAACACCATGATCAGTTTTAACATTCTTGAGGCTGGGCGTATCAATGGTGTGAAGAGGTTCTTCTATGCCTCGAGCGCATGCATTTACCCTGAATTCAAGCAGCTTGAGACA---AAT------GTG---------AGCTTGAAGGAAGCTGATGCCTGGCCTGCTGAGCCACAAGATGCCTATGGCTTGGAGAAGCTGGCGACTGAGGAGCTGTGCAAGCACTACACCAAGGACTTTGCCATTGAGTGCCGTGTTGGCCGTTTCCACAATATTTACGGTCCCTTTGGAACATGGAAAGGTGGTCGCGAGAAGGCACCTGCTGCCTTCTGCAGGAAGGCCCAGACCTCCACAGAGCGGTTTGAGATGTGGGGTGACGGTCTCCAGACCCGATCCTTCACTTTTATCGACGAGTGTGTCGAGGGTGTTCTGAGGTTGACAAAGTCGGACTTCCGCGAGCCAGTGAACATTGGGAGCGATGAAATGGTGAGCATGAACGAGATGGCAGAGATTGTTCTGGGCTTTGAGGACAAG---AAGCTGCCCATCCACCACATCCCTGGTCCCGAGGGTGTCCGTGGACGCAACTCCGACAATACCCTTATCAAGGAGAAGCTTGGCTGGGCTCCCACGATGAGACTCAAGGATGGCCTGAGGTTCACCTACTTCTGGATCAAGGAGCAGATCGAGAAGGAGAGGACGGAGGGGATG---GACGTGGCC---AGGTACGGGTCATCCAAGGTGGTGTCTACCCAAGCGCCGGTTCAGCTGGGCTCTCTCCGCGCCGCTGACGGCAAGGAG---------------------------------------------------------------------------

>BdGME-2

ATGGCA------------------------------CTCAAC------------------AAGGAGTATACGTAT---GCGGATCTGGAGAAGGAACCATACTGGCCATTTGAGAAGCTGCGGATCTCGATTACGGGAGCTGGCGGGTTCATAGCCTCCCACATCGCGAGGCGCCTGAAGGGCGAGGGGCACTACATTATCGCCTCTGACTGGAAGAAGAATGAGCACATGGAGGAGGATATGTTCTGTCATGAGTTCCATCTTGCTGATCTGAGGGTGATGGACAACTGCCTCAAGGTGACCACTGGGGTCGATCATGTTTTCAACCTTGCAGCTGATATGGGAGGGATGGGCTTCATCCAGTCTAACCACTCCGTGATCATGTACAACAACACTATGATCAGCTTTAACATGCTTGAGGCTGCTAGAATCAATGGCATCAAAAGGTTCTTTTATGCCTCAAGTGCTTGCATCTACCCTGAATTTAAGCAGCTGGAAACT---GTA------GTT---------AGCTTGAAGGAGGCAGATGCTTGGCCTGCTGAGCCTCAAGACGCTTATGGCTTGGAGAAACTTGCTACTGAGGAACTGTGCAAGCATTACACAAAGGACTTTGGCATTGAGTGCAGGGTTGGTCGCTTTCACAATATATATGGTCCATATGGAACATGGAAGGGTGGAAGGGAGAAGGCACCTGCTGCTTTCTGCAGAAAGGCTCAGACCTCCACTGATCGCTTTGAGATGTGGGGAGATGGTCTGCAGACTAGATCCTTCACATTTATTGATGAATGTGTAGAGGGTGTCCTCAGGCTAACGAAGTCTGATTTCCGTGAGCCTGTAAACATCGGAAGCGATGAAATGGTGAGCATGAATGAGATGGCTGAGATAGTCCTCGGCTTTGAGAACAAG---CAGCTGCCCATCCACCACATCCCAGGCCCTGAGGGTGTTCGTGGCCGGAACTCCGACAACACTCTCATCAAGGAGAAGCTTGGCTGGGCTCCAACCATGAGGCTGAAGGATGGGCTGAGGATCACATACTTCTGGATCAAGGAGCAGCTAGAGAAGGAAAGGGCGGAAGGCGGC---GACGTTTCA---GCCTATGGATCATCCAAGGTCTGCACCACGCAGGCGCCAGTCCAGCTCGGCTCCCTCCGCGCTGCAGATGGCAAGGAG---------------------------------------------------------------------------

>BrGME-1

ATGGCTGCAGCTACC------AAT---GGAAGTACTGACTAC------------------GGTGCCTACACATAC---AAGGAGCTTGAGAGGGAGCTCTACTGGCCTTCCGAGAAGCTGAGGATCTCCATCACCGGCGCTGGAGGTTTCATCGCTTCCCACATTGCTCGCCGTCTGAAGCACGAGGGCCACTACGTCATTGCTTCTGACTGGAAGAAGAATGAGCACATGACTGAGGACATGTTCTGCGACGAGTTCCACCTTGTTGACCTCAGGGTCATGGAGAATTGCCTCAAGGTCACCGATAAAGTTGATCATGTTTTTAACTTGGCTGCTGATATGGGTGGGATGGGTTTCATCCAGTCCAACCACTCGGTGATTATGTATAACAACACCATGATTAGCTTCAACATGATTGAGGCTGCTAGGATCAATGGGATCAAGAGGTTCTTCTATGCTTCAAGTGCATGTATCTATCCGGAGTTCAAGCAGCTGGAAACTACCAAT------GTG---------AGCCTCAAGGAGTCAGATGCTTGGCCTGCTGAGCCTCAAGATGCTTATGGTCTGGAGAAACTTGCAACGGAGGAGCTCTGCAAGCATTACAACAAAGATTTTGGCATTGAGTGCCGCATTGGAAGGTTCCATAACATCTATGGTCCCTTTGGAACATGGAAAGGTGGAAGAGAGAAGGCTCCAGCTGCTTTCTGCAGGAAGGCTCTGACCTCCACAGATAGGTTTGAGATGTGGGGAGACGGGCTTCAGACCCGTTCATTTACCTTCATTGATGAGTGTGTTGAAGGTGTACTCAGGTTGACTAAGTCAGACTTCCGTGAGCCGGTGAACATTGGAAGCGATGAGATGGTGAGCATGAATGAGATGGCTGAGATGGTTCTCAGCTTTGAGGAAAAG---AAGCTTCCAATTCACCACATTCCAGGCCCTGAAGGTGTTCGTGGCCGTAACTCAGACAACAATCTGATCAAAGAGAAGCTTGGTTGGGCTCCAACTATGAGATTGAAGGAGGGGCTTAGAATAACCTACTTCTGGATAAAGGAACAGATTGAGAAAGAGAAGGCTAAAGGCAGT---GATGTGACG---CTTTATGGTTCATCTAAGGTGGTTGGGACTCAAGCACCGGTTCAGCTTGGCTCACTCAGAGCTGCTGATGGAAAAGAG---------------------------------------------------------------------------

>BrGME-2

ATGGCGTCAACCACC------AAT---GGAAGTACTGACTAT------------------GGTGCCTACACATAC---AAGGACCTCTCTCGAGAGCTTTACTGGCCTTCTCACAAGCTCAAGATATCAATCACAGGAGCCGGAGGTTTCATAGCCTCCCACATCGCTCGCCGTCTAAAGCACGAGGGCCATTACGTCATTGCATCCGACTGGAAGAAGAATGAGCACATGACTGAAGACATGTTCTGCGACGAGTTCCACCTCGTTGATCTCAGGGTCATGGAGAATTGCCTCAAGGTTACTGATGGTGTTGATCACGTCTTTAACTTGGCTGCTGATATGGGCGGTATGGGTTTCATCCAGTCCAACCACTCTGTTATCATGTATAACAATACTATGATCAGCTTTAATATGATCGAGGCTGCTAGGATCAATGGGATTAAGAGGTTCTTTTATGCTTCTAGTGCATGTATCTATCCAGAGTTCAAGCAGCTGGAAACTAGTAAT------GTG---------AGCCTCAAGGAGTCAGATGCTTGGCCTGCAGAGCCACAAGATGCTTATGGTTTGGAGAAACTAGCAACGGAGGAGCTGTGCAAGCATTACAACAAAGATTTTGGGATTGAGTGCCGCATTGGAAGGTTCCATAACATTTACGGTCCTTTCGGAACATGGAAAGGTGGAAGAGAGAAGGCACCAGCTGCTTTCTGTAGAAAGGCTCTCACCTCCACAGATAGGTTTGAGATGTGGGGAGATGGGCTTCAAACTCGCTCTTTCACCTTCATTGATGAGTGTGTTGAAGGTGTACTCAGGTTGACAAAATCAGATTTCCGTGAGCCGGTGAACATAGGAAGCGATGAGATGGTGAGCATGAACGAGATGGCTGAGATGGTTCTGAGCTTTGAGGAAAAG---AAGCTACGGATTCACCACATTCCAGGACCAGAAGGTGTTCGTGGCCGTAACTCAGACAACAAGCTGATCAAAGAGAAGCTTGGCTGGGCTCCAACAATGAGACTGAAGGAGGGGCTTAGAATAACTTACTTCTGGATAAAGGAACAGATTGAGAAAAAGAAAGCTAAAGGCAGT---GATGTGTCG---CTTTATGGGTCTTCAAAAGTGGTTGGGACTCAAGCACCGGTTCAGCTTGGTTCACTCCGAGCTGCTGATGGAAAAGAG---------------------------------------------------------------------------

>BrsGME-1

ATGGGG---AGCACCGACAAGACC---GGAACT---CCTTAT------------------GGTGAGTACACATAT---GCTGAGCTGGAGAGGGAACTGTACTGGCCATCTGAGAAGCTGAGGATTTCGATCACTGGAGCTGGTGGTTTCATTGGATCCCATATTGCTCGTCGTCTGAAGAGTGAGGGACACTACATCATCGCCTCTGACTGGAAGAAGAATGAGCATATGACCGAGGACATGTTCTGCCATGAGTTCCACCTTGTTGACCTCAGGGTCATGGATAACTGCCTTAAGGTCACCAGTGGTGTGGACCATGTCTTCAACCTTGCTGCTGATATGGGTGGCATGGGGTTTATTCAGTCCAACCACTCCGTTATCATGTACAACAACACCATGATCAGTTTTAACATGCTTGAGGCTGGGCGTATCAATGGTGTGAAGAGGTTCTTCTATGCCTCAAGCGCATGCATTTACCCTGAATTCAAGCAGCTTGAGACA---AAT------GTG---------AGCTTGAAGGAAGCTGATGCCTGGCCTGCTGAGCCTCAAGATGCTTATGGCCTGGAGAAGCTGGCGACTGAGGAGCTGTGCAAGCACTACACCAAGGACTTTGCCATTGAGTGTCGTGTTGGCCGTTTCCACAATATTTACGGTCCCTTTGGAACATGGAAAGGTGGTCGCGAGAAGGCACCTGCTGCCTTCTGCAGGAAGGCGCAGACTTCCACCGAGCGGTTTGAGATGTGGGGTGATGGTCTCCAGACCCGATCCTTCACTTTTATCGACGAGTGTGTCGAGGGTGTTCTGAGGTTGACAAAGTCGGACTTCCGCGAGCCTGTGAACATTGGGAGCGATGAAATGGTGAGCATGAACGAGATGGCAGAGATTGTTCTGAGCTTCGAGGATAAG---AAGCTGCCTATCCACCACATCCCTGGCCCTGAGGGTGTCCGTGGACGCAACTCCGACAATACCCTTATCAAGGAGAAGCTTGGCTGGGCTCCCACAATGAAACTCAAGGATGGCCTGAGGTTCACCTATTTCTGGATCAAGGAGCAGATCGAGAAGGAGAGGACGGAGGGGATG---GACGTGGCG---AGGTACGGGTCATCCAAGGTGGTGTCAACCCAAGCGCCGGTTCAGTTGGGCTCCCTCCGCGCCGCTGACGGCAAGGAG---------------------------------------------------------------------------

>BrsGME-2

ATGGCA------------------------------CTCAAC------------------AAGGAGTATACGTAT---GCGGATCTGGAGAAGGAACCATACTGGCCATTTGAGAAGCTGCGGATCTCGATTACGGGAGCTGGCGGGTTCATAGCCTCCCACATTGCGAGGCGCCTGAAGGGCGAGGGGCACTACATTATCGCTTCTGACTGGAAGAAGAATGAGCACATGGATGAGGATATGTTCTGTCATGAGTTCCATCTTGCTGATCTGAGGGTGATGGACAACTGCCTCAAGGTGACCACTGGGGTTGATCATGTTTTCAACCTTGCAGCTGATATGGGAGGGATGGGCTTCATCCAGTCTAACCACTCCGTGATCATGTACAACAACACTATGATCAGCTTTAACATGCTTGAGGCTGCTAGAATCAATGGCATCAAAAGGTTCTTTTATGCCTCGAGTGCTTGCATCTACCCTGAATTTAAGCAGCTGGAAACT---GTA------GTT---------AGCTTGAAGGAGGCAGATGCTTGGCCTGCTGAGCCTCAAGACGCCTATGGCTTGGAGAAACTTGCTACTGAGGAACTGTGCAAGCACTACACAAAGGACTTTGGCATTGAGTGCAGGGTTGGTCGCTTTCACAATATATATGGTCCATACGGAACATGGAAGGGTGGAAGGGAGAAGGCACCTGCTGCTTTCTGCAGAAAGGCTCAGACCTCCACTGACCGCTTTGAGATGTGGGGAGATGGTCTGCAGACTAGATCCTTCACATTTATTGATGAATGTGTAGAGGGTGTCCTCAGGCTAACGAAGTCTGATTTCCGTGAGCCTGTAAACATCGGAAGCGATGAAATGGTGAGCATGAATGAGATGGCTGAGATAGTCCTCGGCTTTGAGAACAAG---CAGCTGCCCATCCACCACATCCCAGGCCCTGAGGGTGTCCGTGGCCGGAACTCTGACAACACGCTCATCAAGGAGAAGCTTGGCTGGGCTCCAACCATGAGGCTGAAGGATGGGCTGAGGATCACATACTTCTGGATCAAGGAGCAGCTGGAGAAGGAAAGGGCAGAAGGTGGT---GATGTTTCG---GCCTATGGATCATCCAAGGTCTGCACCACGCAGGCGCCAGTCCAGCTCGGCTCCCTCCGCGCCGCAGATGGCAAGGAG---------------------------------------------------------------------------

>BsGME

ATGGGA---ACTACC------AAT---GGAAGT---GATTAT------------------GGTGCTTACACATAC---AAGGAGCTAGAGAGAGAGCCTTATTGGCCATCTGAGAAGCTGAAGATATCAATAACTGGTGCTGGAGGTTTCATTGCGTCTCACATTGCTCGGCGTTTGAAACACGAAGGGCATTACGTGATTGCTTCTGACTGGAAGAAGAATGAACATATGACTGAAGATATGTTCTGTGATGAATTTCACCTTGTTGATCTCAGGGTTATGGAGAATTGTCTCAAGGTTACAAAAGGAGTTGATCATGTCTTTAATTTAGCTGCTGATATGGGTGGTATGGGTTACATTCAGTCTAACCACTCGGTGATCATGTATAATAATACTATGATCAGTTTCAATATGATTGAGGCGGCTAGGATCAATGGGATTAAGAGGTTCTTCTATGCTTCTAGTGCGTGTATCTATCCGGAGTTCAAGCAGTTGGAAACTACTAAC------GTG---------AGCCTGAAGGAGTCAGATGCTTGGCCTGCAGAGCCTCAAGATGCTTATGGTTTGGAGAAACTAGCAACGGAGGAGTTGTGTAAGCATTACAACAAAGATTTTGGAATTGAGTGCCGCATTGGAAGGTTTCATAACATTTATGGTCCTTTTGGAACATGGAAAGGTGGAAGGGAGAAGGCTCCAGCTGCTTTCTGCAGAAAGGCTCTCACTTCCACAGATAGATTCGAGATGTGGGGAGACGGGCTTCAAACCCGCTCATTTACCTTCATCGATGAGTGCGTTGAAGGTGTACTTAGGCTGACAAAATCAGATTTCCGTGAGCCAGTGAACATCGGAAGCGATGAGATGGTGAGCATGAATGAGATGGCTGAGATGGTTCTCAGTTTTGAGGTGAAG---AAGCTTCCAATTCACCACATTCCGGGCCCAGAAGGTGTTCGTGGCCGTAACTCAGACAACAATCTCATCAAAGAAAAGCTTGGTTGGGCTCCAAATATGAGATTGAAGGAGGGGCTTAGAATAACCTACTTCTGGATAAAGGAACAGATCGAGAAAGAGAAAGAAAAAGGCAGT---GACGTGTCG---CTTTACGGGTCATCAAAGGTGGTTGGAACTCAAGCACCGGTTCAGCTGGGCTCACTCCGTGCGGCTGATGGAAAAGAG---------------------------------------------------------------------------

>CcGME

ATGGGA---AGTACT------GAA---GGAACC------TAT------------------GGTGCATACACCTAT---GAGGAGCTTGAGAGGGAACCTTACTGGCCTTCCGAGAAGCTCCGAATTTCTGTCACTGGGGCAGGTGGTTTTATTGCCTCCCACATTGCTCGGCGTCTGAAGAGTGAAGGCCATTACATTATTGCTTCTGACTGGAAGAAGAATGAGCACATGACTGAGGATATGTTCTGTCATGAATTCCACCTTGTTGACCTTAGGGTCATGGACAATTGCTTGAAAGTTACTAAGGGGGTTGACCATGTGTTTAACCTTGCTGCTGATATGGGTGGTATGGGCTTTATTCAGTCCAACCACTCTGTCATCATGTATAACAATACCATGATCAGCTTCAACATGCTTGAGGCTTCTAGGATCAGTGGAGTAAAGAGGTTTTTCTATGCCTCTAGTGCCTGCATCTATCCCGAATTTAAGCAGCTGGAAACT---AAC------GTA---------AGCTTGAAGGAGTCTGATGCCTGGCCCGCTGAGCCTCAAGATGCTTATGGCTTGGAGAAGCTTGCAAGTGAGGAGTTGTGTAAGCACTACACGAAAGACTTTGGAATTGAGTGTCGTGTTGGAAGATTCCACAATATTTATGGGCCTTTTGGAACATGGAAAGGTGGAAGGGAAAAAGCTCCTGCTGCTTTTTGCCGAAAGGCTCTTACTTCAACTGACAAGTTTGAGATGTGGGGAGATGGACTTCAAACTCGATCTTTCACCTTCATTGATGAATGTGTTGAAGGTGTGCTCAGATTGACGAAGTCAGACTTCCGTGAGCCAGTGAACATTGGTAGTGATGAGATGGTTAGCATGAACGAGATGGCTGAGATTGTTCTTAGCTTTGAGGACAAG---AAGCTACCTATCCACCACATTCCTGGCCCTGAGGGTGTCCGTGGTCGTAATTCAGACAACACACTGATCAAAGAGAAGCTTGGTTGGGCACCATCCATGAAGTTGAAGGATGGGCTGAGAATTACGTATTTCTGGATCAAGGAACAGATTGAGAAGGAGAAGACTCAAGGTATT---GACCTATCT---GTTTATGGATCATCGAAGGTGGTAGGAACTCAAGCACCAGTTCAGTTAGGCTCACTTCGTGCTGCTGATGGCAAAGAA---------------------------------------------------------------------------

>CgGME

ATGGGA---ACTACC------AAT---GGAACT---GATTAT------------------GGTGCTTACACATAC---AAGGAGCTTGAGAGGGAGCCTTATTGGCCATCTGAGAAGCTTAAGATATCAATCACTGGTGCTGGTGGTTTCATTGCTTCTCACATTGCTCGTCGTTTGAAGCATGAAGGGCATTACGTGATTGCTTCTGATTGGAAGAAGAATGAGCATATGACTGAAGATATGTTTTGTGATGAGTTCCACCTTGTTGATCTTAGGGTTATGGAGAATTGTCTCAAGGTTACTAAAGGTGTTGATCATGTGTTTAACTTGGCTGCTGATATGGGTGGTATGGGTTTTATCCAGTCCAACCATTCTGTGATTATGTATAACAATACTATGATTAGTTTCAACATGATTGAGGCTGCTAGGATCAATGGGATTAAGAGGTTCTTCTATGCTTCTAGTGCGTGTATCTATCCGGAGTTTAAGCAGCTGGAGACTACTAAT------GTG---------AGCCTTAAGGAGTCAGATGCTTGGCCTGCAGAGCCTCAAGATGCTTATGGTTTGGAGAAACTTGCAACGGAGGAGTTGTGCAAGCATTACAACAAAGATTTTGGAATTGAGTGCCGCATTGGAAGGTTCCATAACATTTATGGTCCTTTTGGAACGTGGAAAGGTGGAAGGGAGAAGGCTCCAGCTGCTTTCTGCAGAAAGGCTCTCACCTCCACAGATAAGTTTGAGATGTGGGGAGATGGTCTTCAAACCCGCTCATTTACCTTCATTGATGAGTGTGTTGAAGGTGTACTCAGGTTGACAAAATCTGATTTCCGTGAGCCAGTGAACATCGGAAGCGATGAGATGGTGAGCATGAATGAGATGGCTGAGATGGTTCTCAGTTTTGAGGAGAAG---AAGCTTCCAATTCAGCACATTCCAGGCCCAGAAGGTGTTCGTGGCCGTAACTCAGACAACAATCTCATCAAAGAAAAGCTTGGTTGGGCTCCAAATATGAGATTGAAGGAGGGGCTAAGAATAACCTACTTCTGGATAAAGGAACAGATCGAGAAAGAGAAAGCAAAAGGAAGC---GATGTTACG---CTTTACGGTTCATCAAAGGTGGTTGGAACTCAAGCACCGGTGCAGCTAGGCTCACTCCGCGCTGCCGATGGAAAAGAG---------------------------------------------------------------------------

>ChrGME

ATGGCC---ACCGCC------GCAGTTCACGAG---GACTAT------------------GCTTCCGTCTCGAAACTCGCCAAGTACCCGTTTGAGCCCTACTGGCCCAGTGCCAAGCTCAGGATTTGCGTTACAGGCGCGGGCGGCTTCATTGCCAGCCACCTGGCCAAGCGTCTCAAGTCTGAGGGTCACTACATCGTGGCTTGCGACTGGAAGCGCAATGAGCACTTCGCGGAGGAGGAGTTCTGCCACGAGTTCCACCTGGTGGACCTGCGCGTGTACGACAATTGCAAGAAGGTGTGCGAGGGCTGTGAGCACGTGTTCAACCTGGCCGCCGACATGGGCGGCATGGGCTTCATCCAGTCCAACCACTCCGTCATCATGTACAACAACACGATGGTGTCTTTCAACATGATGGAGGCGGCGCGCGTCACCGGCATCAAGCGCTTCTTCTACGCCTCGTCCGCCTGCATCTACCCCGAGTACAAGCAGCTGGATGTG---GAG------GTGGAGGGCGGCGGCCTCAAGGAGGACGATGCCTGGCCCGCCCAGCCCCAAGATGCGTACGGCCTGGAGAAGCTGGCCACCGAGGAGCTGTGCAAGCACTACAACAAGGACTTCGGCATTGAGTGCCGCATCGCGCGCTTCCACAACATTTACGGCCCGCACGGCACCTGGAAGGGCGGCCGCGAGAAGGCACCCGCGGCCTTCTGTCGCAAGGTGCTCACCTCCACCTCGGAGATCGAGATGTGGGGCGACGGCAAGCAGACGCGCTCCTTCACCTTCATTGACGACTGCGTGGAGGGCATTCTGCGCATCACCAAGTCGGACTTCCGCGACCCCCTGAACCTGGGCTCCACCGAGATGGTGTCCATGAACGGCATGATGGAGCTGGCCATGTCCTTCGACGACAAG---AAGCTGCCCATCAAGCACATCCCCGGCCCCGAGGGCGTGCGCGGCCGCAACAGCGACAACAAGCTCATCCTCGAGAAGCTGGGCTGGGAGCCCACCGTGACGCTCGCGGACGGCCTCAAGCGCACCTACGAGTGGATCAAGGGGCAGCTGGATGCGGAGAAGGAGAAGGGCGTG---GACGCAACC---AAGTACAGCCACAGCACCATCGTGCAGACCAGCGCGCCCATCGAGCTGGGGTCGCTGCGCAAGGCTGACGGCGAGGAGGGCTTCGAG------------------------------------------------------------------

>CosGME

ATGGCC---ACCAAC------ACACTCAACTCT---GAGTAC------------------TATGAGTCCAGCAAGCTCGCCAAGTTCCCATTTGAGCCGTACTGGCCCGAGCAGAAGCTCAAGATTTGCGTGACAGGCGCTGGCGGGTTCATCGCATCCCACCTGGCAAAGCGTCTGAAGTCCGAAGGACACTACCTGGTCTGTGCAGACTGGAAGCGCAACAGCTTCATGCCGGAGGAGGCGTTTTGCGATGAGTTCCACCTGGTGGATCTCCGCGTCTACGACAACTGCAAGAAGGTTGTGAAAGGCTGCGACCACGTCTTCAACCTTGCAGCTGACATGGGAGGTATGGGCTTCATCCAGTCCAACCACTCCGTCATCATGTACAACAACACCATGATCAGCTTCAACATGATGGAGGTGGCTCGCATTGAGGGCATCAAGAGGTTCTTCTATGCCTCCAGCGCCTGCATCTACCCCGAGAACAGGCAGCTGGAGACA---GAG------ATCGAAGGCGGTGGCCTGAAGGAGGACACTGCCTGGCCTGCACAGCCTCAAGACGCTTACGGTCTGGAGAAGCTTGCATCGGAGGAGCTCGCCATGCACTACGACAAGGACTTCGGCATCGAGTGCAGAATCGCCCGCTTCCACAACATCTACGGACCTTATGGCACCTGGAAGGGAGGCCGCGAGAAGGCCCCGGCTGCTTTCTGCAGAAAGGTGCTGACCTCCCCGAAGGACATTGAGATGTGGGGCGACGGCCTCCAGACGCGCAGCTTCACCTTCATCGATGACTGCGTGGAGGGTATCCTGCGCATCACCAAGTCCGACTACAAGGAGCCCCTCAACCTGGGCAGCTCCGAGATGGTGAGCATGAACGAGATGATGGAGACGATCAAGGGCTTTGAGGCGAAG---GATCTGCCGATCCGCCACATCCCGGGCCCCGAGGGTGTGCGCGGCCGCAACAGCGACAACGCCCTCATCCTGGAGAAGATCGGCTGGGAGCCCACCATCAAGCTGGCAGACGGCCTCCGCGTCACCTACACCTGGATCAAGTCCCAGCTCGAGGAGGAGGCG------GGCACA---GATGCGTCG---GTGTACGCCTCGTCCACCATCGTGCAGACCAGCGCCCCCAAGGAGCTCGGCACCTTGAGGCAGGCCGACGGCGACGAGGGCTTTGCCAGCAAGGCAGCCGCCAAGGTGGCCAAGCTTGCCAACGGCGTTGCCAACGGCCTGAAGACCTCTGCT

>CpGME

ATGGGA---AGCAAT------GAT---GGAACT---GATTAT------------------GGTGCATACACCTAC---AAGGAACTCGAAAGGGAGCCCTATTGGCCGTCTGAGAAGCTTCGAATTTCGATTACTGGGGCAGGTGGTTTCATTGCCTCCCATATTGCTCGTCGTTTGAAAAGCGAGGGCCATTACATAATTGCTTCAGACTGGAAGAAGAATGAGCATATGACTGAGGATATGTTCTGTCATGAATTCCACCTTGTTGATCTCAGGGTTATGGACAATTGCTTGAAGGTTACTAAGGGAGTTGATCATGTGTTTAACCTTGCTGCTGACATGGGTGGGATGGGATTCATCCAGTCCAACCACTCGGTCATTATGTATAACAATACCATGATCAGCTTCAACATGCTTGAGGCTTCCAGGATTAATGGAGTCAAGAGATTTTTCTATGCATCCAGCGCGTGTATCTATCCTGAATTTAAGCAGCTAGAAACT---AAT------GTG---------AGCTTGAAGGAGTCTGATGCTTGGCCTGCAGAGCCTCAAGATGCATATGGTTTGGAAAAGCTTGCGACTGAGGAATTATGCAAGCACTATACAAAAGATTTCGGAATTGAGTGTCGTATTGGAAGATTCCATAACATTTATGGTCCTTTTGGGACGTGGAAAGGTGGAAGGGAGAAGGCACCGGCTGCTTTTTGTAGAAAGGCTATCACTTCCACTGACAAGTTTGAGATGTGGGGAGATGGAATTCAAACTCGCTCTTTCACCTTCATCGATGAATGTGTTGAAGGTGTCCTCAGATTAACAAAGTCAGATTTCCGTGAGCCGGTAAACATTGGAAGTGATGAGATGGTTAGCATGAATGAGATGGCTGAGATTGTTCTCAGTTTTGAGAACAAA---AAGCTTCCCATCCAACACATTCCTGGCCCAGAGGGAGTGCGAGGTCGTAATTCGGACAATACACTGATAAAAGAGAAGCTTGGCTGGGCTCCTACAATGAAGTTGAAGGATGGGCTAAGAATTACATACTTCTGGATCAAGGAACAAATTGAGAAAGAGAAATCTCAGGGTATT---GATCTGTCT---ATTTATGGATCATCTAAGGTGGTGGGAACTCAAGCACCGGTTCAGTTGGGCTCACTTCGTGCTGCTGATGGCAAAGAG---------------------------------------------------------------------------

>CrGME

ATGGGA---ACTACC------AAT---GGAACT---GATTAT------------------GGTGCTTACACATAC---AAGGAGCTTGAGAGGGAGCCTTATTGGCCATCTGAGAAGCTTAAGATATCAATCACTGGTGCTGGTGGTTTCATTGCTTCTCACATTGCTCGTCGTTTGAAGCATGAAGGGCATTACGTGATTGCTTCTGATTGGAAGAAGAATGAGCATATGACTGAAGATATGTTTTGTGATGAGTTCCACCTTGTTGATCTTAGGGTTATGGAGAATTGTCTCAAGGTCACTAAAGGTGTTGATCATGTGTTTAACTTGGCTGCTGATATGGGTGGTATGGGTTTTATCCAGTCCAACCATTCTGTGATTATGTATAACAATACTATGATTAGTTTCAACATGATTGAGGCTGCTAGGATCAATGGGATTAAGAGGTTCTTCTATGCTTCTAGTGCGTGTATCTATCCGGAGTTTAAGCAGCTGGAAACTACTAAT------GTG---------AGCCTTAAGGAGTCAGATGCTTGGCCTGCAGAGCCTCAAGATGCTTATGGTTTGGAGAAACTTGCAACGGAGGAGTTGTGCAAGCATTACAACAAAGATTTCGGAATTGAGTGCCGCATTGGAAGGTTCCATAACATTTATGGTCCTTTTGGAACGTGGAAAGGTGGAAGGGAGAAGGCTCCAGCTGCTTTCTGCAGAAAGGCTCTCACCTCCACAGATAAGTTTGAGATGTGGGGAGATGGTCTTCAAACCCGCTCATTTACCTTTATTGATGAGTGTGTTGAAGGTGTACTCAGGTTGACAAAATCTGATTTCCGTGAGCCAGTGAACATCGGAAGCGATGAGATGGTGAGCATGAATGAGATGGCTGAGATGGTTCTCAGTTTTGAGGAAAAG---AAGCTTCCAATTCAGCACATTCCAGGCCCAGAAGGTGTTCGTGGCCGTAACTCAGACAACAATCTCATCAAAGAAAAGCTTGGTTGGGCTCCAAATATGAGATTGAAGGAGGGGCTTAGAATAACCTACTTCTGGATAAAGGAACAGATCGAGAAAGAGAAAGCAAAAGGAAGC---GATGTTACG---CTTTACGGTTCATCAAAGGTGGTTGGAACTCAAGCACCGGTGCAGCTAGGCTCACTCCGCGCTGCCGATGGAAAAGAG---------------------------------------------------------------------------

>CsGME

ATGGGA---AGTACT------GAA---GGAACC------TAT------------------GGTGCATACACCTAT---GAGGAGCTTGAGAGGGAACCTTACTGGCCTTCCGAGAAGCTCCGAATTTCTGTCACTGGGGCAGGTGGTTTTATTGCCTCCCACATTGCTCGGCGTCTGAAGAGTGAAGGCCATTACATTATTGCTTCTGACTGGAAGAAGAATGAGCACATGACTGAGGATATGTTCTGTCATGAATTCCACCTTGTTGACCTTAGGGTCATGGACAATTGCTTGAAAGTTACTAAGGGGGTTGACCATGTGTTTAACCTTGCTGCTGATATGGGTGGTATGGGCTTTATTCAGTCCAACCACTCTGTCATCATGTATAACAATACCATGATCAGCTTCAACATGCTTGAGGCTTCTAGGATCAGTGGAGTAAAGAGGTTTTTCTATGCCTCCAGTGCCTGCATCTATCCCGAATTTAAGCAGCTGGAAACT---AAC------GTA---------AGCTTGAAGGAGTCTGATGCCTGGCCCGCTGAGCCTCAAGATGCTTATGGCTTGGAGAAGCTTGCAAGTGAGGAGTTGTGTAAGCACTACACGAAAGACTTTGGAATTGAGTGTCGTGTTGGAAGATTCCACAATATTTATGGGCCTTTTGGAACATGGAAAGGTGGAAGGGAAAAAGCTCCTGCTGCTTTTTGCCGAAAGGCTCTTACTTCAACTGACAAGTTTGAGATGTGGGGAGATGGACTTCAAACTCGATCTTTCACCTTCATTGATGAATGTGTTGAAGGTGTGCTCAGATTGACGAAGTCAGACTTCCGTGAGCCAGTGAACATTGGTAGTGATGAGATGGTTAGCATGAATGAGATGGCTGAGATTGTTCTTAGCTTTGAGGACAAG---AAGCTACCTATCCACCACATTCCTGGCCCTGAGGGTGTCCGCGGTCGTAATTCAGACAACACACTGATCAAAGAGAAGCTTGGTTGGGCACCATCAATGAAGTTGAAGGATGGGCTGAGAATTACGTATTTCTGGATCAAGGAACAGATTGAGAAGGAGAAGACTCAAGGTATT---GACCTATCT---GTTTATGGATCATCGAAGGTGGTAGGAACTCAAGCTCCAGTTCAGTTAGGCTCACTTCGTGCTGCTGATGGCAAAGAA---------------------------------------------------------------------------

>CusGME

ATGGGG---AGCGCA------GGG---GAAACC---ACTTAT------------------GGGTCGTACACTTAT---CAGGAGCTTGAAAGAGAGGCTTATTGGCCATCGGAGAAACTGAGAATCTCCATCACTGGAGCTGGTGGGTTTATTGCTTCTCACATTGCCAGGCGTTTGAAGAGCGAAGGGCACTACATCATTGCTTCTGACTGGAAGAAGAATGAACACATGACAGAGGATATGTTTTGCCATGAATTTCATCTTGTTGATCTCAGAGTGATGGATAATTGTATGAAGGTGACTGAGAATGTTGACCATGTCTTCAATCTGGCTGCTGATATGGGTGGTATGGGCTTCATCCAGTCCAACCACTCAGTTATTATGTATAACAACACCATGATCAGCTTCAATATGCTTGAAGCTGCAAGAATCAATGGGGTTAAGAGGTTTTTCTATGCTTCCAGTGCTTGCATCTATCCTGAATTTAAGCAATTGGAAACT---AAC------GTG---------AGCTTGAAGGAGTCAGATGCCTGGCCTGCTGAGCCTCAAGATGCCTACGGTCTGGAGAAGCTTGCTACAGAGGAGTTGTGCAAACATTATACTAAGGATTTTGGTATTGAGTGCCGAATTGGGAGGTTCCATAACATTTATGGCCCATTTGGAACATGGAAAGGTGGAAGGGAAAAGGCTCCTGCTGCATTTTGCAGAAAGGCCCTCACTTCTGTAGACAAGTTTGAGATGTGGGGAGATGGTCTTCAGACAAGATCTTTCACTTTCATTGATGAATGTGTAGAAGGTGTCCTGAGATTGACTAAGTCAGACTTCCGTGAGCCAGTGAATATTGGAAGCGATGAGATGGTTAGCATGAATGAAATGGCAGAAATCGTGCTTAGCTTCGACGACAAA---AAACTTCCAATTCATCACATCCCTGGTCCCGAGGGTGTCCGTGGTCGTAACTCAGACAACACACTCATTAAAGAGAAACTTGGGTGGGCTCCCACGATGAAGTTGAAGGATGGATTGAGAATTACCTACATGTGGATCAAGGAGCAGATTGAAAAAGAGAAGTCTAAAGGCATT---GACCTGACA---GTTTATGGATCATCAAAGGTTGTTGGTACCCAAGCTCCGGTTCAACTCGGGTCACTCCGTGCCGCTGATGGTAAAGAG---------------------------------------------------------------------------

>DcGME-1

ATGGGC---AGCACT------GAG---CAAAAC---CTCTAC------------------GGAGCATACACCTAC---GAGAGCCTTGAGAGGGAACCTTACTGGCCTGCTGAAAATCTCAGAATTTCCATCACTGGAGCAGGTGGATTCATTGCCTCCCATATTGCGAGGCGTTTGAAGAGCGAAGGACATTACATCATTGCTTCTGACTGGAAGAAAAATGAGCACATGCCAGAAGACATGTTTTGTCATGAGTTTCATCTTGCTGATCTTAGAGTCATGGATAACTGTTTAAAAGTTACACAGAATGTTGACCATGTGTTTAATCTTGCTGCTGATATGGGAGGTATGGGCTTCATTCAGTCTAATCACTCAGTGATAATGTACAACAACACGATGATTAGTTTCAACATGCTTGAAGCAGGAAGGATCAATGGCGTCAAGAGGTTGTTTTATGCGTCTAGCGCTTGTATCTACCCTGAGTTTAAGCAATTGGAAACT---AAT------GTG---------AGCTTGAAGGAGTCTGATGCGTGGCCTGCAGAGCCTCAAGATGCATATGGCTTAGAAAAGCTAGCAACAGAAGAATTGTGCAAGCACTACACCAAGGATTTCGGTATTGAATGCCGAATTGGACGCTTCCACAACATTTATGGACCATTTGGAACATGGAAAGGTGGAAGAGAAAAGGCCCCTGCTGCTTTTTGTAGAAAAGCACTAACTTCTACTGATAAGTTTGAGATGTGGGGTGATGGTCTGCAAACTAGATCATTCACTTTCATAGATGAATGTGTTGAAGGTGTCCTGAGGTTGACAAAGTCTGATTTCCGAGAGCCAGTAAATATTGGAAGTGATGAGATGGTAAGCATGAATGAAATGGCGGAGATCGTCCTCAGCTTTGAGGATAGG---AAACTTCCTATCCAGCACATACCTGGCCCTGAGGGTGTCCGTGGACGGAACTCGGATAACACTCTGATTAAGGAGAAGCTTGGTTGGGCCCCTACCATGAAGCTAAAGGACGGATTGAGGATCACATACTTCTGGATAAAGGAGCAAATTGAGAAAGAGAAAGCTAAGGGTGGT---GACATGTCA---GTTTATGGGTCATCAAAAGTTGTGGGGACGCAGGCTCCAGTCCAGTTGGGTTCTCTTCGTGCAGCTGATGGCAAAGAA---------------------------------------------------------------------------

>DcGME-2

ATGGGC---AGCACT------GAG---CAAAAC---CTTTAT------------------ATAGCGTACACCTAT---GAGAGCCTTGAGAGGGAACCTTACTGGCCATCTGAGAAACTCCGAATTTCTATTACCGGAGCAGGTGGGTTTATTGCCTCACACATTGCAAGGCGTTTGAAGAGCGAAGGGCATTACATCATTGCTTCTGACTGGAAGAGAAATGAGCACATGCCAGAAGAAATGTTTTGTCATGAATTTCATCTTGTTGACCTTAGAGTCATGGATAACTGTCTGAAAGTTTCTGAGAATGTTGATCATGTGTTCAATCTTGCTGCTGACATGGGTGGTATGGGCTTCATTCAGTCCAATCACTCAGTGATTATGTATAACAACACTATGATCAGTTTTAACATGCTTGAAGCTGCAAGGATCAATGGCATCAAGAGGTTCTTTTATGCATCTAGCGCTTGTATCTACCCTGAATTTAAGCAATTGGAAACT---AAT------GTC---------AGCTTAAAGGAGGCTGATGCTTGGCCTGCAGAGCCTCAAGATGCATATGGCTTAGAAAAGCTGGCAACAGAAGAGTTATGCAAGCACTATACCAAGGATTTCGGAATTGAGTGCCGAATTGGTCGCTTCCACAACATCTACGGACCATTTGGAACTTGGAAAGGTGGGAGAGAAAAGGCACCTGCTGCTTTCTGTAGGAAAGCCCTTACCTCTACTGATAAATTTGAGATGTGGGGAGATGGTCTGCAAACTAGATCATTCACATTCATTGACGAATGTGTCGAAGGTGTTCTTAGATTGACCAAGTCTGATTTCAGAGAGCCAGTAAATATTGGAAGTGATGAAATGGTAAGCATGAACGAAATGGCGGAGATCATCCTCAGCTTTGAGGACAAG---AAACTTCCTATCCAACACATACCAGGTCCTGAGGGTGTTCGTGGACGGAATTCTGACAACACTTTAATCAAAGAGAAGCTTGATTGGGCCCCTACCATGAAGCTGAAGGATGGATTGAACATCACATACTTCTGGATAAAGGAGCAAATTGAGAAAGAGAAAGCCAAGGGTGGC---GATCTGTCA---GTTTATGGTTCATCAAAAGTTGTTGGCACACAGGCTCCTGTCCAGCTAGGTTCTCTTCGTGCAGCTGATGGCAAAGAA---------------------------------------------------------------------------

>EgGME-1

ATGGGG---AGCATT------GAT---GGGACT---GACTAT------------------GGCGCGTTCACATAT---GAAAACCTCGAAAGGGAGCCTTACTGGCCATCCGAGAAACTCAGAATCTCCATCACTGGTGCAGGTGGTTTCATTGCCTCTCACATTGCCCGGCGCCTGAAAAGTGAGGGCCACTACATTATCGCTTCAGACTGGAAGAAGAACGAGCACATGACTGAAGACATGTTCTGCCATGAATTCCATCTTGTCGACCTTAGAGTCATGGATAACTGCTTAAAGGTTACTAAGGGGGTCGACCATGTCTTTAACCTCGCTGCCGATATGGGTGGCATGGGCTTCATTCAGTCCAATCATTCCGTCATTATGTATAACAACACAATGATTAGCTTTAACATGCTCGAGGCTGCTAGGATAAATGGAGTAAAGAGGTTTTTCTATGCTTCTAGTGCTTGCATTTATCCTGAATTTAAGCAACTGGAGACC---AAT------GTG---------AGCTTGAAAGAGTCTGATGCATGGCCTGCTGAGCCTCAAGATGCTTATGGCTTGGAGAAGCTCGCTACTGAGGAATTGTGCAAGCATTACACCAAAGATTTTGGAATTGAGTGCCGCGTTGGAAGGTTCCACAACATTTATGGTCCTTTCGGGACATGGAAAGGGGGAAGGGAGAAGGCTCCGGCTGCTTTTTGCAGGAAGACCATCACTTCCACTGACAAGTTCGAAATGTGGGGAGATGGGCTTCAGACTCGATCTTTTACTTTCATTGACGAGTGTGTTGAAGGTGTTCTTAGATTGACAAAGTCAGATTTCCGTGAGCCAGTGAACATTGGAAGTGATGAGATGGTTAGCATGAATGAAATGGCTGAGATCGTTCTTAGCTTTGAGAATAAG---AAGCTTCCCATTCATCACATTCCTGGTCCTGAGGGTGTCCGTGGACGCAACTCGGACAACACGCTTATCAAGGAGAAGCTTGGTTGGGCTCCTACCATGAAATTGAGGGATGGCTTGAGAATCACATACTTCTGGATCAAAGAACAGATTGAGAAAGAAAAGGCTCAAGGGATG---GACCTCTCC---ATTTATGGTTCATCTAAGGTTGTAGGAACTCAAGCCCCTGTTCAGCTCGGTTCACTCCGAGCTGCCGATGGCAAGGAA---------------------------------------------------------------------------

>EgGME-2

ATGGGA---AGCATC------GAT---AGAAGC---AGTTAT------------------GGTGCGTACACCTAC---GAGTCCCTCGAGAGGGAGCCCTACTGGCCATCTCAGAAACTCAGGATTTCCATCACTGGGGCTGGGGGTTTCATCGCCTCCCACATTGCTCGGCGTTTGAAGAGCGAGGGTCACTACATCATTGCTTCTGACTGGAAGAAGAATGAGCACATGACCGAGGATATGTTCTGTAATGAATTCCACCTCGTTGACCTTAGGGTCATGGACAACTGCTTGAAGGTCACTCAGGGGGTTGACCATGTCTTCAACCTCGCTGCCGATATGGGCGGCATGGGTTTCATTCAGTCCAACCACTCGGTCATCATGTACAACAACACCATGATCAGCTTCAACATGCTTGAGGCGTCAAGAATTAACAGTGTGAAGAGGTTCTTTTATGCCTCCAGTGCTTGTATTTATCCAGAATTTAAGCAGCTGGAGACC---AAC------GTG---------AGCCTCAAGGAATCCGATGCCTGGCCTGCCGAGCCTCAAGATGCGTATGGCTTGGAAAAGCTTGCGACAGAGGAGTTGTGCAAGCACTACACCAAGGATTTTGCAATTGAATGCCGAATTGGAAGGTTCCATAACATTTATGGTCCTTTTGGAACATGGAAAGGTGGAAGGGAGAAAGCTCCGGCTGCTTTTTGCAGGAAGGCACTCACTTCCACTGACAAGTTTGAGATGTGGGGAGATGGACTGCAAACTCGCTCCTTCACATTCATCGATGAATGTGTTGAAGGTGTGCTGAGATTGACAAAGTCAGACTTCCGTGAGCCTGTGAACATCGGAAGTGATGAGATGGTGAGCATGAATGAGATGGCAGAGATGGTTCTCAGTTTTGAGGACAAA---AAGCTCCCAATTCACCACATTCCTGGGCCAGAGGGTGTACGTGGACGCAACTCAGAGAACACGCTGATTAAGGAGAAACTCGGATGGGCTCCTACCATGAGGTTGAAGGATGGGTTGAGAATTACATACTTCTGGATCAAGGAACAGATTGAGAAGGAGAAAGCGCAGGGCACG---GATCTGTCA---ATCTACGGGTCATCAAAAGTGGTGGGCACTCAAGCCCCTGTTCAGCTCGGTTCACTTCGAGCTGCCGATGGCAAAGAG---------------------------------------------------------------------------

>EsGME

ATGACA---ACAACC------AAT---GGAGCT---GACTAT------------------GGTGCCTACACATAC---AAGGAGCTTGAGAGAGAGCTTTACTGGCCTTCTGAGAAGCTGAGGATATCGATCACCGGTGCCGGAGGTTTCATCGCTTCCCACATTGCTCGCCGTTTGAAGCACGAAGGTCATTACGTGATTGCTTCTGACTGGAAGAAGAATGAGCACATGACTGAAGACATGTTCTGCAATGAGTTCCATCTCGTTGATCTCAGGGTCATGGAGAATTGCCTCAAGGTCACTAATGGTGTTGATCACGTCTTTAACTTAGCCGCCGACATGGGTGGTATGGGTTTCATCCAGTCCAACCACTCGGTCATCATGTACAACAACACCATGATCAGCTTCAACATGATCGAGGCTGCTAGGATCAATGGGATTAAGAGGTTCTTCTATGCCTCTAGTGCATGTATCTATCCCGAGTTCAAGCAGCTGGAAACGACTAAT------GTG---------AGCCTCAAGGAGTCAGATGCTTGGCCTGCAGAGCCTCAAGATGCTTATGGTTTGGAGAAACTTGCAACGGAGGAGTTGTGCAAGCATTACAACAAAGATTTTGGAATTGAGTGCCGCATTGGGAGGTTCCATAACATTTACGGTCCTTTTGGAACGTGGAAAGGTGGAAGAGAGAAGGCTCCAGCTGCGTTCTGCAGAAAGGCTCTCACCTCCACGGATAGGTTTGAGATGTGGGGAGACGGGCTTCAAACCCGCTCTTTCACCTTCATTGATGAGTGTGTTGAAGGTGTGCTTAGGTTGACAAAATCAGATTTCCGTGAGCCGGTGAACATTGGAAGCGATGAGATGGTGAGCATGAATGAGATGGCTGAGATGGTTCTCAGCTTTGAGGAGAAG---AATCTTCCGATTCACCACATTCCAGGCCCAGAAGGTGTTCGTGGCCGTAACTCAGACAACAATCTGATAAAAGAAAAGCTTGGTTGGGCTCCAACAATGAGATTGAAGGAGGGGCTTCGAATAACCTACTTCTGGATAAAGGAACAAATTGAGAAGGAGAAGGCTAAAGGCAGT---GATGTTACG---CTTTACGGTTCATCAAAGGTGGTTGGGACTCAAGCACCGGTTCAGCTTGGATCACTCCGCGCGGCTGATGGAAAAGAG---------------------------------------------------------------------------

>FvGME

ATGGGA---AGTGCT------GGT---GAGAGT---GGCTAT------------------GGCGCATACACCTAT---GAGGCCCTTGAGAGGGAGCCTTATTGGCCCTCGGAAAAACTCCGAATTTCAATTACTGGGGCTGGTGGCTTTATCGCCTCCCACATTGCCCGCCGATTGAAGAATGAGGGTCACTACATTATTGCTTCTGACTGGAAGAAGAATGAGCACATGACTGAAGACATGTTCTGCGATGAATTCCATCTTGTGGATCTCAGGGTCATGGATAACTGCTTGAAGGTTACCAAGGACGTTGATCATGTGTTCAACCTTGCTGCTGATATGGGCGGCATGGGATTCATTCAGTCCAATCACTCTGTCATTATGTACAACAATACCATGATCAGCTTTAACATGCTGGAAGCTGCTAGGATCACTGGAGTGAAAAGGTTTTTCTATGCTTCCAGTGCTTGTATTTATCCTGAGTTTAAGCAGCTGGAAACT---AAT------GTG---------AGCTTGAAGGAGGCGGATGCCTGGCCGGCAGAGCCTCAAGATGCTTATGGCTTAGAGAAACTTGCAACTGAGGAGTTGTGCAAGCACTACACCAAAGACTTTGGAATCGAATGTCGTATTGGAAGGTTCCACAATATTTATGGTCCTTTTGGAACCTGGAAAGGGGGCAGGGAAAAGGCACCTGCTGCTTTCTGCAGAAAGGCTCTCACATCCACTGATAAGTTTGAGATGTGGGGAGATGGACTTCAGACCCGATCCTTCACCTTTATTGATGAATGTGTTGAAGGTGTACTCCGGTTGACAAAGTCCGACTTCCGTGAGCCAGTGAATATTGGAAGTGACGAGATGGTCAGCATGAATGAGATGGCTGAGATTGTTCTTAGCTTTGAGAACAAG---AAGCTGCCTATTCAGCACATTCCTGGACCAGAGGGTGTCCGTGGTCGTAACTCTGACAACACTCTGATCAAAGAGAAACTTGGCTGGGCTCCTACCATGAGGCTGAAGGATGGACTGAGGTTTACATACTTCTGGATCAAGGAACAGATTGAGAAAGAGAAAGCACAAGGTACT---GACCTGTCG---GTTTATGGGTCATCTAAGGTGGTGGGAACTCAAGCCCCAGTTCAACTTGGTTCACTTCGTGCCGCTGATGGCAAAGAG---------------------------------------------------------------------------

>GmGME-1

ATGGGA---AGTTCT------GGA---ACAACC---GACTAT------------------GGAGCATACACATAC---CAAAACCTTGAGAGGGAACCTTACTGGCCCTCTGAAAAGCTCAGAATTTCCATCACTGGGGCTGGTGGTTTCATTGCCTCACACATTGCTCGCCGCCTCAAGACCGAGGGACATTACATCATTGCTTCTGATTGGAAGAAGAATGAGCACATGACTGAGGACATGTTCTGCCATGAATTCCATCTTGTTGACCTTAGGGTCATGGATAACTGCTTGACAGTTACCAAGGGTGTGGACCATGTTTTCAATCTTGCTGCTGATATGGGTGGGATGGGTTTTATCCAGTCCAACCACTCAGTCATTATGTACAACAACACCATGATTAGCTTCAACATGATTGAGGCTGCCAGGATCAATGGTGTTAAGAGGTTTTTTTATGCCTCTAGTGCTTGTATCTATCCTGAATTCAAACAGTTGGAGACA---AAT------GTG---------AGTTTGAAGGAGTCTGATGCCTGGCCTGCTGAGCCACAAGATGCATATGGGCTGGAGAAGCTTGCAACAGAAGAGTTATGCAAGCATTATAACAAGGATTTTGGAATTGAGTGCCGGATTGGGAGATTCCATAACATATATGGTCCTTATGGGACATGGAAGGGTGGAAGGGAGAAGGCTCCTGCTGCTTTTTGTCGCAAGACACTTACTTCCAAAGATCGATTTGAGATGTGGGGAGATGGATTGCAAACAAGATCCTTCACCTTCATTGATGAGTGTGTTGAAGGTGTACTGAGATTGACTAAATCAGACTTCCGGGAGCCAGTGAATATTGGAAGTGATGAAATGGTCAGTATGAATGAGATGGCAGAGATTGTTCTTAGCTTTGAGGATAAG---AATATACCAATATACCATATTCCTGGCCCAGAAGGTGTTCGGGGCCGTAATTCAGACAATACATTAATCAAAGAGAAACTTGGCTGGGCTCCAACTATGAAGTTGAAGGATGGGCTGAGAATTACATACTTTTGGATCAAAGAGCAGCTTGAGAAAGAGAAGGCAGAAGGTGTT---GATTTATCA---GGCTATGGATCATCCAAAGTGGTTCAGACTCAAGCCCCAGTTCAACTTGGCTCGCTACGTGCTGCAGATGGCAAAGAA---------------------------------------------------------------------------

>GmGME-2

ATGGGA---AGTTCT------GGA---ACAACC---GACTAT------------------GGAGCATACACATAC---CAAAACCTTGAGAGGGAACCTTACTGGCCCTCTGAAAAGCTCAGAATTTCCATCACTGGGGCTGGTGGTTTCATTGCCTCGCACATTGCTCGCCGCCTCAAGACCGAGGGACATTACATCATTGCTTCTGATTGGAAGAAGAATGAGCACATGACTGAGGACATGTTCTGCCATGAATTCCATCTTGTTGACCTTAGGGTCATGGATAACTGCTTGACAGTTACCAAGGGTGTGGACCATGTTTTCAATCTTGCTGCTGATATGGGTGGGATGGGTTTTATCCAGTCCAACCACTCAGTCATTATGTACAACAACACCATGATTAGCTTCAACATGATTGAGGCTGCCAGGATCAATGGTGTTAAGAGGTTTTTTTATGCCTCTAGTGCTTGTATCTATCCGGAATTCAAACAGTTGGAGACA---AAT------GTG---------AGTTTAAAGGAGTCTGATGCCTGGCCTGCTGAGCCACAAGATGCATATGGGCTGGAGAAGCTTGCAACAGAAGAGTTATGCAAGCATTATAACAAGGATTTTGGAATTGAGTGCCGGATTGGGAGATTCCATAACATATATGGTCCTTATGGGACATGGAAGGGTGGAAGGGAGAAGGCTCCTGCTGCTTTTTGTCGCAAGACACTTACTTCCAAAGATCGATTTGAGATGTGGGGAGATGGATTGCAAACAAGATCCTTCACCTTCATTGATGAGTGTGTTGAAGGTGTACTGAGATTGACTAAATCAGACTTCCGGGAGCCGGTGAATATTGGAAGTGATGAAATGGTCAGCATGAATGAGATGGCTGAGATTGTTCTTAGCTTTGAGGATAAG---AATATACCAATATACCATATTCCTGGCCCAGAAGGTGTTCGGGGCCGTAATTCAGACAATACATTAATCAAAGAGAAACTTGGCTGGGCTCCAACTATGAAGTTGAAGGATGGGCTGAGAATTACATACTTTTGGATCAAAGAGCAGCTTGAGAAAGAGAAGGCAGAAGGTGTT---GATTTATCA---GGCTATGGATCATCCAAAGTGGTTCAGACTCAAGCCCCAGTTCAACTCGGCTCGCTTCGGGCTGCAGATGGCAAAGAA---------------------------------------------------------------------------

>GmGME-3

ATGGGA---AGTGCT------GGA---GGAACT---GACTAT------------------GGTGCATACACTTAT---GAGAATCTTGAGAGAGAGCCTTACTGGCCATCAGAGAAGCTTAAGATTTCCATCACTGGTGCTGGGGGTTTTATCGCGTCACACATAGCTCGGCGCCTCAAGACAGAGGGGCATTACATTATTGCTTCTGATTGGAAGAAAAATGAGCACATGACTGAGGACATGTTCTGTGATGAATTCCATCTTGTTGATCTCAGGGTCATGAATAACTGCCTCAAGGTTACAGAGGGGGTTGATCATGTTTTCAATCTTGCCGCAGACATGGGTGGGATGGGTTTTATTCAGTCTAACCACTCTGTCATTATGTACAACAACACAATGATTAGCTTCAACATGATTGAGGCTGCCAGGATTAACGGCATTAAGAGGTTTTTTTATGCCTCTAGTGCTTGTATCTACCCTGAATTCAAACAGTTGGAAACT---AAT------GTT---------AGCTTGAAGGAGTCTGATGCATGGCCAGCTGAGCCACAAGATGCATATGGGCTAGAGAAGCTTGCAACAGAGGAATTATGCAAGCACTATAACAAGGATTTTGGAATTGAGTGCCGCATTGGGAGGTTCCACAACATATACGGTCCTTTTGGGACATGGAAAGGTGGAAGGGAGAAGGCTCCTGCTGCTTTTTGTCGTAAAGTTATCACTTCCTCTGATAGATTTGAGATGTGGGGGGATGGATTGCAAACACGATCATTTACCTTCATTGATGAATGTGTTGAAGGGGTGCTCAGATTGACTAAATCTGATTTCCGAGAGCCAGTAAATATTGGAAGCGATGAGATGGTCAGCATGAATGAGATGGCTGAGATCATTCTTGGCTTTGAGAACAAG---AATATTCCTATTCACCACATTCCTGGCCCCGAGGGTGTCCGAGGTCGTAATTCAGACAATACACTTATAAAAGAAAAACTTGGTTGGGCTCCAACTATGAGGTTGAAGGATGGACTGAGGATCACATACTTCTGGATTAAGGAGCAGATCGAGAAGGAGAAGGCTCAAGGTATT---GATATATCA---GTGTATGGGTCTTCCAAAGTGGTGCAGACTCAAGCCCCAGTTCAACTAGGCTCACTTCGAGCAGCAGATGGCAAAGAA---------------------------------------------------------------------------

>GmGME-4

ATGGGA---AGTGCT------GGA---AGAACT---GATTAT------------------GGTGCATACACCTAT---GAGAATCTTGAGAGAGAGCCTTACTGGCCATCAGAGAAGCTTAAGATTTCCATCACCGGTGCTGGGGGTTTTATCGCATCACACATAGCTCGGCGCCTCAAGACAGAGGGGCATTACGTTATTGCTTCGGATTGGAAGAAAAATGAGCACATGACTGAGAACATGTTCTGTGATGAATTCCATCTTGTTGATCTCAGGGTCATGGATAACTGCCTCAAGGTTACAAAGGGGGTTGATCATGTTTTCAATCTTGCTGCAGACATGGGTGGGATGGGTTTTATTCAGTCTAACCACTCTGTCATTATGTACAACAACACAATGATTAGCTTCAACATGATTGAGGCTGCCAGGATTAACGGCATTAAGAGGTTTTTTTATGCCTCTAGTGCTTGTATCTACCCTGAATTTAAACAGTTGGAAACT---AAT------GTG---------AGCTTGAAGGAGTCCGATGCATGGCCAGCTGAGCCTCAAGATGCATATGGGCTAGAGAAACTTGCAACAGAGGAATTATGCAAGCACTATAACAAGGATTTTGGAATTGAGTGCCGCATTGGGAGGTTCCACAACATATACGGTCCTTTTGGGACATGGAAAGGTGGAAGGGAGAAGGCTCCTGCTGCTTTTTGTCGTAAAGTAATCACTTCCACTGATAGATTTGAGATGTGGGGAGATGGATTGCAAACACGATCATTTACCTTCATTGATGAGTGTGTTGAAGGTGTGCTTAGATTGACTAAATCTGACTTCCGAGAGCCAGTAAATATTGGAAGCGATGAGATGGTTAGCATGAATGAGATGGCTGAGATTATTCTTGGCTTTGAGAACAAG---AATATTCCTATTCACCACATTCCTGGCCCTGAGGGTGTTCGAGGTCGTAATTCAGACAATACACTGATAAAAGAAAAACTTGGTTGGGCTCCAACTATGAGGTTGAAGGATGGGTTGAGAATCACATACTTCTGGATTAAGGAGCAGATCGAGAAGGAGAAGGCTCAAGGTATT---GATATATCT---GTGTATGGGTCTTCCAAAGTGGTGCAGACTCAAGCCCCAGTTCAACTAGGCTCACTTCGAGCTGCAGATGGCAAAGAA---------------------------------------------------------------------------

>GmGME-5

ATGGGA---ATTTCT------GGA---ACAACA---GACTAT------------------GGATCATTCACATAC---CAAAACCTTGAGAGGGAACCCTACTGGCCCTCAGAAAAGCTCAGAATTTCCATCACTGGGGCTGGTGGTTTCATTGCCTCACACATTGCTCGCCGCCTCAAGACGGAGGGACATTACATCATTGCTTCTGATTGGAAGAAGAATGAGCACATGACTGAGGGCATGTTCTGCCATGAATTCCATCTTGTTGACCTTAGGGTCATGGATAACTGCTTGACAGTTACCAAGGGTGTGGACCATGTTTTCAATCTTGCTGCTGATATGGGTGGGATGGGTTTCATCCAGTCCAACCACTCGGTCATTATGTACAACAACACCATGATTAGCTTCAACATGATTGAGGCTGCCAGGATCAATGGTGTTAAGAGGTTTTTTTATGCCTCTAGTGCTTGTATCTATCCTGAATTCAAGCAGTTGGAGACA---AAT------GTG---------AGTTTGAAGGAGTCTGATGCCTGGCCTGCTGAGCCACAAGATGCATATGGGCTGGAGAAGCTTGCAACAGAAGAGTTATGCAAGCATTATAACAAGGATTTTGGAATTGAGTGCCGCATTGGGAGATTCCATAACATATATGGTCCTTATGGGACATGGAAGGGTGGGAGGGAGAAGGCTCCTGCTGCTTTTTGTCGCAAGACACTTACTTCCAAAGACCGATTTGAGATGTGGGGAGATGGATTGCAAACAAGATCCTTCACCTTCATTGATGAGTGTGTTGAAGGTGTACTGAGATTGACTAAATCAGACTTCCGGGAGCCGGTGAATATTGGAAGTGATGAAATGGTCAGCATGAATGAGATGGCTGAGATTGTTCTTAGCTTTGAGGATAAG---AATATACCAATATACCATATTCCTGGCCCTGAAGGTGTTCGGGGTCGTAATTCAGACAATACATTAATCAAAGAGAAACTTGGCTGGGCTCCAACTATGAAGTTGAAGGATGGGCTGAGAATTACATACTTTTGGATCAAAGAGCAGCTTGAGAAAGAGAAGGCAGAAGGTGTT---GATTTATCA---GGCTATGGATCATCCAAAGTGGTTCAGACTCAAGCCCCAGTTCAACTTGGTTCCCTTCGGGCTGCAGATGGCAAAGAA---------------------------------------------------------------------------

>GrGME-1

ATGGGA---AGTACC------GAT---GGGACC---AGCTAT------------------GGTGCTTACACCTAT---GATGCCCTGGAGAGGGAGCCTTACTGGCCATCCGAGAAACTTCGAATTTCCATCACCGGTGCTGGTGGCTTCATCGCTTCGCACATCGCTCGACGTCTGAAGAGTGAAGGACATTACATCATTGCTTCTGATTGGAAGAAGAATGAGCACATGACAGAAGATATGTTCTGTCACGAATTCCATCTTGTCGATCTTCGAGTGATGGAAAATTGCTTGAAGGTTACCAATGGAGTGGATCATGTTTTCAACCTCGCCGCTGATATGGGTGGCATGGGCTTCATCCAGTCCAACCACTCGGTCATTATGTACAACAACACAATGATCAGTTTCAACATGCTCGAGGCTGCTCGAATCAGTGGAGTTAAGAGGTTTTTTTACGCCTCCAGTGCTTGTATCTATCCTGAATTTAAGCAATTGGAAACT---AAT------GTG---------AGCTTGAAAGAATCCGATGCCTGGCCTGCTGAGCCACAAGATGCTTACGGCTTGGAGAAGCTTGCAACGGAGGAGTTGTGCAAGCACTACACCAAAGACTTTGGAATTGAATGTCGCATTGGAAGGTTCCACAACATTTACGGCCCTTTTGGAACTTGGAAAGGTGGAAGGGAGAAGGCTCCAGCTGCCTTTTGCAGAAAGGCTATTACTTCCATTGACAAGTTTGAGATGTGGGGAGATGGTCTTCAGACCCGATCTTTCACTTTCATTGATGAATGTGTTGAAGGTGTACTTAGATTGACAAAGTCTGATTTCCGTGAGCCTGTGAACATCGGAAGTGATGAGATGGTTAGCATGAACGAGATGGCGGAGATTGTGCTTAGCTTTGAAGATAAA---AAGCTTCCAATCCACCATATCCCCGGTCCAGAGGGTGTCCGTGGTCGTAATTCAGACAATACATTGATCAAAGAAAAACTTGGTTGGGCCCCTACAATGAGGTTGAAGGATGGGCTGAGAATTACATACTTCTGGATCAAGGAACAGATTGAGAAAGAGAAGGTTCAAGGCATT---GATCTATCT---GTTTACGGATCATCTAAGGTGGTAGGAACCCAAGCACCGGTCCAGCTCGGGTCACTTCGTGCTGCTGATGGCAAAGAA---------------------------------------------------------------------------

>GrGME-2

ATGGGC---AGTACT------GAT---GGAACC---AGCTAT------------------GGTGCTTACACCTAT---GATGCTCTGGAGAGGGAGCCTTACTGGCCATCTCAGAAACTCCGGATTTCCATCACTGGTGCCGGTGGGTTCATTGCTTCCCATATTGCTCGACGTCTGAAGAGTGAAGGCCATTACATCATTGCTTCTGACTGGAAGAAGAACGAGCACATGACAGAAGATATGTTTTGTAATGAATTCCACCTCGTTGATCTTCGAGTCATGGAGAATTGCTTGAAAGTTTCCAAAGGAGTGGACCATGTCTTCAACCTCGCTGCCGATATGGGTGGGATGGGTTTCATTCAGTCAAACCACTCTGTCATTATGTACAACAACACTATGATCAGTTTCAACATGCTTGAGGCTGCTAGGATTAGTGGAGTTAAGAGGTTTTTCTATGCCTCGAGTGCTTGTATCTACCCTGAATTTAAGCAGTTGGACACT---AAT------GTG---------AGCCTAAAAGAATCCGATGCCTGGCCTGCTGAGCCTCAAGATGCTTATGGCTTGGAAAAGCTTGCAACTGAGGAGTTGTGCAAGCACTACACCAAAGATTTTGGAATCGAGTGTCGTATTGGAAGGTTTCACAACATTTATGGTCCTTTTGGAACATGGAAAGGTGGAAGGGAGAAGGCTCCAGCTGCCTTTTGCAGAAAAGCTATTACTTCCATTGACAAGTTTGAGATGTGGGGAGACGGTCTTCAGACCCGATCTTTCACCTTCATTGATGAATGTGTCGAAGGTGTACTTAGATTGACAAAGTCAGATTTCCGCGAACCTGTGAACATTGGAAGTGATGAGATGGTCAGCATGAATGAGATGGCAGAAATTGTTCTTAGCTTTGAGGATAAA---AATCTTCCAATCCATCATATTCCTGGCCCAGAGGGTGTCCGTGGTCGTAATTCAGACAATACGTTGATCAAAGAAAAACTTGGTTGGGCTCCTACAATGAGGTTGAAGGATGGGCTGAGAATTACATACTTCTGGATCAAGGAACAAATTGAGAAAGAGAAGGCTCAAGGCATA---GAGCTATCT---GTCTACGGGTCATCCAAGGTCGTGGGAACTCAAGCACCAGTCCAGCTGGGCTCACTTCGTGCAGCCGATGGCAAAGAA---------------------------------------------------------------------------

>KfGME

ATGGGA---ACTACC------GAT---GGAGGC---AAATAC------------------GGGGCATTCACATAC---GAGAACCTCGAGAGGGAGCCCTACTGGCCATCCGAGAAGCTTCGGATATCCATAACAGGTGCTGGTGGATTCATTGCCTCACACATTGCTCGGAGACTGAAGAGTGAAGGGCACTATATCATTGCATCCGACTGGAAGAAGAACGAGCACATGACTGAGGACATGTTTTGCCATGAGTTCCATCTTGCTGATCTGAGAGTCATGGAGAATTGCTTGAAGGTGACGTCTGGGGTAGATCATGTGTTCAACCTTGCGGCTGATATGGGAGGCATGGGGTTTATCCAGTCAAACCACTCGGTCATCATGTACAATAACACCATGATCAGCTTTAATATGATGGAGGCCGCCAGAATCAATGGGGTCAAGAGGTTCTTTTACGCATCTAGTGCGTGTATTTACCCTGAGTTCAAGCAATTGGAAACT---AAT------GTT---------AGCTTGAAGGAGTCAGATGCATGGCCTGCAGAGCCGCAAGACGCCTATGGTCTTGAGAAGCTTGCAACTGAGGAGCTGTGCAAACATTACAACAAGGATTTCGGGATCGAATGCCGCATTGGGCGGTTCCACAATATCTACGGCCCCTTTGGAACATGGAAAGGAGGGCGTGAGAAGGCCCCTGCAGCCTTCTGTAGGAAGGCTCTCACTGCGACCGACAAGTTTGAGATGTGGGGGGATGGGCTTCAAACCCGTTCTTTCACCTTCATTGATGAGTGTGTCGAGGGGGTTCTTAGGCTGACAAAGTCAGACTTCCGAGAACCAGTCAACATTGGAAGTGATGAGATGGTCAGCATGAATGAGATGGCCGAGATTGTCCTCAGCTTTGAGGACAGG---AAGCTCCCTATTGAGCACATACCCGGTCCGGAAGGTGTCCGAGGCCGGAACTCAGACAACACCCTGATCAAGGAAAAGCTCGGCTGGGCTCCTTCCATGAAATTAAAGGATGGCCTCAGGATCACTTACTTCTGGATCAAAGAGCAGATTGAGAAAGAGAAGGCCGAAGGGAAG---GATTTATCA---GTCTATGGTTCGTCCAAGGTTGTGGGAACTCAAGCTCCGGTTCAGCTGGGTTCCCTTCGTGCCGCTGACGGCAAGGAA---------------------------------------------------------------------------

>KlGME-1

ATGGGA---ACTACC------GAT---GGAGGC---AAATAC------------------GGGGCATTCACATAC---GAGAACCTCGAGAGGGAGCCCTACTGGCCATCCGAGAAGCTTCGGATATCCATAACCGGTGCTGGTGGATTCATTGCTTCTCACATTGCTCGGCGATTGAAGAGTGAAGGGCACTACATCATTGCTTCCGACTGGAAGAAGAACGAGCACATGACTGAGGACATGTTTTGCCATGAGTTCCATCTTGCTGATCTGAGGGTCATGGAGAATTGCTTGAAGGTGACGTCTGGGGTCGATCATGTGTTCAACCTTGCGGCTGATATGGGAGGCATGGGGTTTATCCAGTCAAACCACTCGGTCATCATGTACAATAACACCATGATCAGCTTTAATATGATGGAGGCCGCCAGAATCAATGGGGTCAAGAGGTTCTTTTACGCATCTAGTGCGTGCATTTACCCTGAGTTCAAGCAATTGGAAACT---AAT------GTT---------AGCTTGAAGGAGTCAGATGCGTGGCCTGCAGAGCCGCAAGACGCCTATGGTCTCGAGAAGCTTGCAACTGAGGAGCTGTGCAAGCATTACAACAAGGATTTCGGGATCGAATGCCGCATTGGGCGGTTCCACAATATCTACGGCCCCTTTGGAACATGGAAAGGAGGGCGTGAGAAGGCCCCTGCAGCCTTCTGTAGGAAGGCTCTCACTGCGACCGACAAGTTTGAGATGTGGGGGGATGGGCTTCAAACCCGTTCTTTCACCTTCATTGATGAGTGTGTCGAGGGGGTTCTTAGGCTGACAAAGTCAGACTTCCGAGAACCAGTCAACATTGGAAGTGATGAGATGGTCAGCATGAATGAGATGGCCGAGATTGTCCTCAGCTTTGAGGACAGG---AAGCTCCCTATCGAGCACATACCCGGTCCGGAAGGTGTCCGAGGCCGGAACTCAGACAACACTCTGATCAAGGAAAAGCTCGGCTGGGCTCCTTCCATGAAACTAAAGGATGGCCTCAGGATCACTTACTTCTGGATCAAAGAGCAGATTGAGAAAGAGAAGGCCGAAGGGAAG---GATTTGTCA---GTCTATGGTTCGTCCAAGGTTGTGGGAACTCAAGCTCCGGTTCAGCTGGGTTCCCTTCGTGCCGCTGACGGCAAGGAA---------------------------------------------------------------------------

>KlGME-2

ATGGGA---ACTACC------GAT---GGAGGC---AAATAC------------------GGGGCATTCACATAC---GAGAACCTCGAGAGGGAGCCCTACTGGCCATCCGAGAAGCTTCGGATATCCATAACTGGTGCTGGTGGATTCATTGCCTCACACATTGCTCGGAGACTGAAGAGTGAAGGGCACTATATCATTGCATCCGACTGGAAGAAGAACGAGCACATGACTGAGGACATGTTTTGCCATGAGTTCCATCTTGCTGATCTGAGAGTCATGGAGAATTGCTTGAAGGTGACGTCTGGGGTAGATCATGTGTTCAACCTTGCGGCTGATATGGGAGGCATGGGGTTTATCCAGTCAAACCACTCGGTCATCATGTACAATAACACCATGATCAGCTTTAATATGATGGAGGCCGCCAGAATCAATGGGGTCAAGAGGTTCTTTTACGCATCTAGTGCGTGCATTTACCCTGAGTTCAAGCAATTGGAAACT---AAT------GTT---------AGCTTGAAGGAGTCAGATGCGTGGCCTGCAGAGCCGCAAGACGCCTATGGTCTCGAGAAGCTTGCAACTGAGGAGCTGTGCAAGCATTACAACAAGGATTTCGGGATCGAATGCCGCATTGGGCGGTTCCACAATATCTACGGCCCCTTTGGAACATGGAAAGGAGGGCGTGAGAAGGCCCCTGCAGCCTTCTGTAGGAAGGCTCTCACTGCGACCGACAAGTTTGAGATGTGGGGGGATGGGCTTCAAACCCGTTCTTTCACCTTCATTGATGAGTGTGTCGAGGGGGTTCTTAGGCTGACAAAGTCAGACTTCCGAGAACCAGTCAACATTGGAAGTGATGAGATGGTCAGCATGAATGAGATGGCCGAGATTGTCCTCAGCTTTGAGGACAGG---AAGCTCCCTATTGAGCACATACCCGGTCCGGAAGGTGTCCGAGGCCGGAACTCAGACAACACCCTGATCAAGGAAAAGCTCGGCTGGGCTCCTTCCATGAAATTAAAGGATGGCCTCAGGATCACTTACTTCTGGATCAAAGAGCAGATTGAGAAAGAGAAGGCCGAAGGGAAG---GATTTATCA---GTCTATGGTTCGTCCAAGGTTGTGGGAACTCAAGCTCCGGTTCAGCTGGGTTCCCTTCGTGCCGCTGACGGCAAGGAA---------------------------------------------------------------------------

>LuGME-1

ATGGGG---AGTAAC------GAT---GGAAGC---AACTAC------------------GGTGCTTATACCTAT---GAGGAGCTAGAGAGAGAGGCTTACTGGCCATCTGAGAAGCTGAGGATCTCAATAACTGGAGCTGGTGGGTTTATTGCCTCTCACATAGCTAGGCGTTTGAAAAGTGAAGGTCACTACATTATTGCTTCTGACTGGAAGAAGAATGAGCACATGCCAGAAGACATGTTCTGTCATGAATTCCATCTTGTGGATCTGAGGGTTATGGATAATTGTTTGAAGGTTACACAAGCTGTTGACCATGTGTTCAATCTGGCTGCTGACATGGGTGGGATGGGCTTTATTCAGTCCAATCATTCTGTCATCATGTACAACAATACCATGATCAGCTTCAACATGCTTGAAGCGTCTAGGATTAATGGTGTTAAGAGGTTCTTTTATGCCTCTAGTGCTTGCATATATCCTGAATTTAAGCAGCTCGAGACA---AAT------GTG---------AGCTTGAAGGAAGCTGATGCCTGGCCTGCAGAGCCTCAAGATGCTTATGGCCTGGAGAAGCTTGCAACTGAGGAGTTGTGCAAGCACTATACCAAAGATTTCGGTATTGAATGCCGTGTTGGAAGGTTCCACAACATTTATGGTCCTTTCGGAACATGGAAAGGTGGGAGGGAAAAGGCACCTGCTGCATTTTGCAGAAAGGCTCTTACCTCTGCTGATAAGTTTGAGATGTGGGGAGATGGACTTCAGACTCGTTCCTTCACCTTTATTGATGAATGTGTCGAAGGTGTTCTCAGGTTGACTAAGTCTGATTTCCGTGAGCCGGTAAACATTGGAAGCGATGAAATGGTTAGCATGAATGAAATGGCTGAGATTGTACTGAGCTTTGAGGAGAGG---AAACTCCCCATCCAGCACATTCCAGGTCCGGAAGGTGTTCGTGGCCGTAACTCTGACAACACTTTGATCAAGGAAAAGCTTGGTTGGGCTCCAACGATGATGCTCAAGGATGGGCTGAAAATCACATACTTCTGGATAAAGGAACAGATTGAGAAAGAGAAGGCTAAAGGAGTA---GACTTGGCA---GTGTACGGGTCATCGAAAGTTGTTGGAACTCAAGCTCCAGTTCAGTTGGGTTCGCTTCGTGCTGCTGATGGCAAGGAA---------------------------------------------------------------------------

>LuGME-2

ATGGGA---AGCAAC------GAT---GAAGCC---AGCTAC------------------GGATCGTACACCTAC---GAGGAGCTCGAGAGGGAGCCATACTGGCCTTCAGAGAAGCTGAGGATCTCAATAACCGGAGCCGGAGGGTTCATTGCATCGCACATAGCTAGGCGTTTGAAGAGCGAAGGGCACTACATCATCGCTTCGGACTGGAAGAAGAACGAGCACATGACCGAGGACATGTTCTGTCACGAATTCCACCTCGTCGATCTTAGGGTCATGGACAATTGCTTGAAGGTCACGAACGGCGTAGACCATGTCTTCAACTTGGCTGCCGATATGGGCGGGATGGGGTTCATCCAGTCGAACCATTCGGTTATCATGTACAACAACACCATGATCAGCTTCAACATGCTCGAAGCCTCCCGGATCAATGGTGTCAAGAGATTCTTTTATGCCTCTAGTGCTTGCATATACCCTGAGTTTAAGCAGCTGGAGACA---AAC------GTG---------AGCTTGAAGGAAGCTGATGCTTGGCCTGCAGAGCCACAAGATGCTTATGGTCTGGAGAAGCTTGCAACAGAGGAGTTGTGCAAGCACTACACCAAGGACTTCGGGATCGAATGTCGAGTCGGAAGGTTCCACAACATCTACGGTCCTTTCGGAACATGGAAAGGTGGCAGGGAGAAGGCACCAGCTGCATTCTGCAGAAAGGCTCTCACTTCTGCAGACAAGTTCGAGATGTGGGGAGACGGTCTTCAAACTCGATCGTTCACCTTCATCGACGAGTGCGTCGAAGGAGTCCTGAGGCTGACCAAGTCCGACTTTCGTGAGCCGGTCAACATTGGGAGCGACGAGATGGTAAGCATGAACGAAATGGCCGAGATTGTTCTGAGCTTTGAAGACAGG---AAGCTCCCCATTCACCACATTCCGGGCCCGGAAGGTGTTCGTGGTCGAAACTCGGATAATACCCTGATCAAGGAGAAACTCGGTTGGGCTCCAACCATGAAACTGAAGGACGGGCTGAGGTTCACTTACTTCTGGATCAAGGAACAGATCGAGAAGGAGAAGGCGAAAGGTGTC---GACTTAGCG---GTGTATGGCTCGTCGAAAGTGGTCGGAACTCAGGCCCCGGTTCAGCTCGGATCCCTCCGTGCTGCTGATGGCAAGGAA---------------------------------------------------------------------------

>LuGME-3

ATGGGA---AGCAAC------GAT---GAAGCC---AGCTAT------------------GGATCGTACACCTAC---GAGGAGCTCCAGCGGGAGCTTTACTGGCCATCGGAGAAGCTGAGGATCTCCATAACCGGAGCCGGAGGGTTCATCGCATCGCACATAGCCAGGCGTCTAAAGAGCGAAGGGCACTACATCATCGCTTCGGACTGGAAGAAGAACGAGCACATGACCGAGGACATGTTCTGTCACGAATTCCACCTTGTGGATCTTAGGGTCATGGACAATTGCTTGAAGGTCACGAATGGTGTAGACCATGTCTTCAACTTGGCTGCCGATATGGGCGGGATGGGGTTCATCCAGTCAAACCATTCCGTCATCATGTACAACAATACCATGATCAGCTTCAACATGCTCGAAGCCTCCCGGATCAATGGTGTCAAGAGGTTCTTTTATGCCTCGAGTGCTTGCATATATCCCGAGTTTAAGCAACTGGAGACA---AAC------GTG---------AGCTTAAAGGAAGCTGATGCTTGGCCAGCAGAGCCACAAGATGCTTACGGTCTGGAGAAGCTTGCAACAGAGGAGTTGTGCAAGCACTACACCAAGGACTTCGGGATCGAATGTCGTGTCGGAAGGTTCCACAACATCTATGGTCCTTTCGGAACATGGAAAGGTGGCAGGGAGAAGGCACCAGCTGCATTCTGCAGAAAGGCTCTCACGTCTGCCGAAAAGTTTGAAATGTGGGGAGACGGTCTTCAAACTCGATCCTTCACCTTCATCGACGAGTGCGTCGAAGGAGTCCTGAGGCTGACCAAGTCCGACTTTCGCGAGCCAGTCAACATTGGGAGCGACGAGATGGTAAGCATGAACGAAATGGCCGAGATTGTTCTGAGCTTTGAAGACAGG---AAGCATCCCATCCACCACATTCCTGGCCCGGAAGGTGTTCGTGGTCGAAACTCGGATAATACTCTTATCAAGGAAAAACTCGGTTGGGCTCCGACCATGAAGCTGAAGGACGGGCTGAGGTTCACTTACTTCTGGATCAAGGAACAGATAGAGAAGGAGAAGGCGAAAGGTGTC---GACTTGGCG---GTTTATGGTTCGTCGAAAGTGGTAGGAACTCAGGCTCCGGTTCAGCTGGGATCGCTCCGTGCTGCCGATGGCAAGGAA---------------------------------------------------------------------------

>LuGME-4

ATGGGG---AGTAAC------GAT---GGCAGC---AGCTAC------------------GGTGCTTACACCTAT---GAGGAGCTAGAGAGAGAGGCTTACTGGCCATCTGAGAAGCTGAGGATCTCAATAACTGGAGCTGGTGGGTTTATTGCCTCTCACATAGCTAGGCGTTTGAAAAGTGAAGGTCACTACATTATTGCTTCCGACTGGAAGAAGAATGAGCACATGCCAGAAGACATGTTCTGTCATGAATTCCATCTTGTGGATCTGAGGGTTATGGATAATTGTTTGAAAGTTACACAAGCTGTTGACCATGTGTTCAACCTGGCTGCTGACATGGGTGGGATGGGCTTTATTCAGTCCAATCATTCTGTCATCATGTACAATAATACCATGATTAGCTTCAACATGCTTGAAGCCTCTAGGATTAATGGTGTTAAGAGGTTCTTTTATGCCTCTAGTGCTTGCATATATCCCGAATTTAAGCAGCTTGAGACT---AAT------GTG---------AGCTTGAAGGAAGCTGATGCCTGGCCTGCAGAGCCTCAAGATGCTTATGGCCTGGAGAAGCTTGCAACTGAGGAGTTGTGCAAGCACTATACCAAAGATTTCGGTATTGAATGTCGTGTTGGAAGGTTCCACAACATTTATGGTCCTTTCGGAACATGGAAAGGTGGGAGGGAAAAGGCACCTGCTGCATTTTGCAGAAAGGCTCTTACTTCTGCTGATAAGTTTGAGATGTGGGGAGATGGACTTCAGACTCGTTCCTTCACCTTTATTGATGAATGTGTCGAAGGTGTTCTCAGGTTGACTAAGTCTGATTTCCGTGAGCCGGTAAACATTGGAAGTGATGAAATGGTTAGCATGAATGAAATGGCTGAGATTGTACTGAGCTTTGAGGATAGG---AAACTACCCATCCAGCACATTCCAGGTCCGGAAGGTGTTCGTGGCCGCAACTCTGACAACACTTTGATAAAGGAAAAGCTTGGTTGGGCTCCAACGATGAGGCTCAAGGATGGGCTGAGAATCACATACTTCTGGATAAAGGAACAGATTGAGAAAGAGAAGGCTAAAGGAGTG---GACTTGGCA---GTGTACGGGTCATCAAAAGTGGTTGGAACTCAAGCTCCAGTTCAGTTGGGGTCGCTTCGTGCTGCTGATGGCAAGGAA---------------------------------------------------------------------------

>MeGME-1

ATGGGA---AGCACT------GAT---GGAACC---AGCTAT------------------GGGGCTTTCACCTAT---GAGAATCTGGAGAGGGAACCATACTGGTCATCTGAGAAGCTCCGAATTTCCATTACTGGGGCTGGTGGTTTTATTGCCTCCCACATTGCTCGTCGTTTGAAGAGTGAAGGCCATTACATTATTGCTTCTGACTGGAAGAAGAATGAGCACATGACAGAAGATATGTTCTGTCATGAGTTCCATCTTGTGGATCTAAGGGTCATGGATAATTGCTTGAAGGTAACAAAGGATGTAGACCATGTGTTTAACCTTGCTGCTGATATGGGTGGCATGGGCTTCATTCAGTCCAACCACTCTGTGATTATGTATAACAATACAATGATCAGCTTCAACATGCTTGAGGCTGCTAGGATTAATGGAGTTAAGAGGTTCTTCTATGCCTCTAGTGCTTGTATTTATCCTGAATTTAAGCAGCTGGACACT---AAT------GTG---------AGCTTGAAGGAATCTGATGCCTGGCCTGCAGAGCCTCAAGATGCATATGGCTTGGAGAAACTTGCTACAGAGGAGTTGTGCAAGCACTACACAAAGGACTTTGGAATTGAATGCCGTATTGGAAGGTTCCATAATATTTATGGTCCTTTTGGAACTTGGAAAGGTGGCAGGGAGAAGGCACCTGCTGCTTTTTGTAGAAAGGCTATCACTTCCACTGATAAGTTTGAGATGTGGGGAGATGGACTTCAAACCCGGTCCTTTACCTTTATTGATGAATGCGTGGAAGGCGTTCTTAGATTGACTAAGTCAGACTTTCGAGAGCCAGTGAACATTGGAAGTGATGAAATGGTCAGCATGAATGAGATGGCTGAGATTGTTCTCAGCTTTGAAAACAAG---AAGCTTCCAATCCATCACATTCCTGGTCCAGAAGGTGTCCGAGGCCGTAACTCAGACAACACTCTTATCAAAGAGAAGCTTGGTTGGGCTCCTACAATGAAACTGAAGGATGGGCTGAGAATTACGTACTTCTGGATCAAGGAACAAATAGAAAAAGAGAAAGCTCAAGGTATT---GACTTGTCC---ATTTATGGATCATCCAAAGTGGTGGGAACTCAAGCGCCAGTTCAGTTGGGTTCACTTCGCGCCGCTGATGGCAAAGAA---------------------------------------------------------------------------

>MeGME-2

ATGGGA---AGCACT------GAG---GGAACC---AACTAT------------------GGGGCTTTTACCTAT---GAGAATCTTGAGAGGGAACCTTACTGGCCATCTGAGAAGCTCCGAATTTCCATTACTGGGGCTGGTGGTTTTATTGCCTCCCACATTGCTCGCCGTTTGAAGAGTGAGGGCCATTACATTATTGCTTCTGATTGGAAGAAGAATGAGCACATGACAGAAGATATGTTCTGCCACGAATTCCATCTGGTGGATCTAAGGGTCATGGATAATTGCTTGAAGGTGACAAAGGATGTAGACCATGTGTTTAACCTTGCTGCTGATATGGGTGGGATGGGCTTCATCCAGTCCAACCACTCTGTCATTATGTATAACAACACAATGATCAGCTTCAACATGCTTGAGGCTGCTAGGATCAGTGGAGTTAAGAGGTTTTTCTATGCCTCTAGTGCTTGTATTTACCCTGAATTTAAGCAGCTGGATACA---AAT------GTG---------AGCTTGAAGGAATCTGATGCCTGGCCTGCAGAGCCTCAAGATGCATATGGCTTAGAGAAACTTGCTACAGAGGAGTTGTGCAAGCACTACACAAAGGACTTTGGAATCGAATGTCGTATTGGAAGGTTCCATAACATTTACGGTCCCTTTGGAACTTGGAAAGGTGGCAGAGAGAAGGCACCTGCTGCTTTTTGCAGAAAGGCTATCACTTCCATCGACAAGTTTGAGATGTGGGGAGATGGACTTCAAACTCGATCCTTCACCTTTATTGATGAATGTGTGGAAGGTGTGCTGAGATTGACCAAGTCGGATTTCCGAGAGCCAGTAAACATTGGAAGTGATGAAATGGTTAGCATGAATGAGATGGCTGATATTGTTCTCAGCTTTGAGAACAAG---AAGCTCCCTATCCATCACATTCCTGGCCCAGAAGGTGTACGGGGCCGTAACTCGGACAACACTCTGATCAAAGAGAAGCTTGGTTGGGCTCCCACAATGAAGCTGAAGGATGGCCTGAGAATTACATACTTCTGGATTAAGGAACAGATTGAGAAAGAGAAGGCTCAAGGTATA---GATTTGTCC---ATTTACGGATCATCTAAAGTGGTGGGAACTCAAGCACCAGTTCAACTGGGTTCACTTCGGGCTGCTGATGGCAAAGAA---------------------------------------------------------------------------

>MgGME-1

ATGGGA---AGCACC------------GGTGAAAACTTGTAC------------------AAGGCCTACACGTAC---GAGAATCTCGAGACGGAGCCCTATTGGCCCTCGGAAAAGCTCCGGATCTCCATAACTGGTGCTGGAGGATTCATCGCGTCTCACATTGCGAGGCGTTTGAAGAGCGAAGGTCATTACATCATCGCATCGGACTGGAAGAAAAACGAGCACATGCCCGAGGACATGTTTTGTCATGAATTCCACCTCGTTGATCTGAGGGTCATGGATAACTGTCTGAAAGTAACCGAAGGTGTGGATCATGTCTTTAATCTTGCTGCTGATATGGGAGGGATGGGCTTCATTCAGTCGAACCACTCCGTCATTATGTACAACAATACTATGATCAGTTTTAATATGATCGAGGCTGGCAGAATTAACGGAGTTAAGAGGTTTTTCTACGCCTCAAGTGCGTGTATTTATCCCGAATTTAAGCAACTGGAGACC---AAT------GTG---------AGCTTGAAAGAATCTGATGCATGGCCCGCCGAGCCTCAAGATGCTTACGGTCTAGAGAAGCTGGCGACAGAGGAATTATGTAAGCACTACAACAAGGACTTTGGAATCGAGTGCAGAATCGGAAGGTTCCATAATATCTACGGTCCCTTTGGAACATGGAAAGGTGGAAGGGAAAAAGCACCAGCTGCTTTCTGTAGGAAAACTCTCACTTCTACCGATAAATTCGAAATGTGGGGAGATGGCCTCCAAACTCGATCTTTCACATTCATCGATGAATGCGTTGAGGGTGTTTTAAGATTAACTAAATCGGACTTCCGAGAGCCTGTGAATATTGGAAGTGACGAGGAGGTGAGCATGAACGGGATGGCCGAAATTATCTCGAGCTTCGAGGATAAG---AAGCTTCCGATCCACCACATTCCGGGACCCGAGGGTGTACGTGGTCGAAATTCCGATAATACCCTAATTAAGGAAAAGCTCGGATGGGCCCCGTCTATGAAACTAAAGGATGGATTGAGAATCACGTATTTCTGGATTAAGGAACAACTCGAGAAAGAGAAAACCCTAGGGGTA---GATTTGTCA---AATTACGGCTCGTCGAAAGTCGTGGGAACTCAAGCCCCGGTTCAACTCGGCTCTCTTCGTGCTGCTGATGGCAAAGAA---------------------------------------------------------------------------

>MgGME-2

ATGGCA---AGCAGT------GAC---GAAACC---AACTAT------------------GGATCATACACATAC---GAAACCCTCGAAAGGGAACCCTACTGGCCATCTGAAAAGCTTCGGATTTCCATTACGGGAGCAGGTGGATTTATCGCTTCCCATATTGCCAGGCGTTTGAAGAGTGAGGGCCATTACATCATCGCTTCGGATTGGAAGAAAAACGAGCACATGACGGAGGATATGTTCTGTAATGAATTCCATCTCGTTGATCTGAGGGTTATGGACAACTGTCTGAAAGTAACCGACGGTGTGGATCACGTCTTCAATCTTGCTGCCGATATGGGAGGCATGGGCTTCATTCAATCCAACCACTCCGTCATTATGTACAACAATACAATGATCAGCTTTAACATGATTGAAGCTGCAAGAATCAACGGTGTTAAGAGGTTTTTTTATGCATCGAGTGCTTGTATCTATCCGGAATTTAAACAGTTGGAGACT---AAT------GTG---------AGCTTGAAGGAATCTGATGCATGGCCTGCTGAGCCTCAAGATGCTTATGGGTTAGAGAAGCTGGCAACCGAAGAATTATGTAAGCACTACAATAAGGACTTCGGGATTGAATGCCGGATTGGAAGGTTTCATAACATTTATGGTCCCTTTGGGACATGGAAAGGTGGCAGGGAGAAAGCACCAGCTGCCTTCTGTAGGAAATCACTTACTTCAACCGATAAGTTTGAAATGTGGGGTGACGGTTTGCAAACTCGATCTTTCACCTTTATTGATGAATGTGTTGAAGGTGTTCTCAGATTGACTAAGTCCGACTTCCGAGAGCCGGTGAATATTGGGAGTGATGAGATGGTGAGCATGAATGAAATGGCAGAGATTGTACTTGGCTTCGAGAACAAG---AAACTGCCCATTTATCACATTCCAGGGCCTGAAGGTGTTCGTGGCCGAAATTCAGACAATACTTTGATTAAGGAAAAGCTTGGGTGGGCCCCTTCTATGAAACTTAAGGACGGGCTGAGAATAACGTATTTCTGGATAAAGGAGCAACTTGAAAAGGAGAAAGCTCATGGAACG---GACTTGTCT---GCTTACGGCTCTTCGAAAGTTGTCGGAACACAGGCTCCCGTTCAACTCGGTTCTCTCCGAGCTGCTGATGGCAAGGAA---------------------------------------------------------------------------

>MipGME

ATGGCG---GCCGCT---------------------GGATACGATAACCTGCAGCTCCGAGGGAAGTACGGCACG------------------GAGAAGTACTGGCCGGAGAAGAAGCTGAAGATTTGCGTCACCGGCGCCGGCGGCTTCATCGCGTCGCACCTCGCCAAGAGGCTCAAGGAAGAGGGGCACCACATCGTCGGATGCGACTGGAAGAGGAACGAGCACATGCCGGAGGAAATGTTCTGCGACGAGTTTATACTCGCGGACCTGCGTCTGTTTGAAAACTGCCAGAAAGTCCTCAAAGGCTGCGACCACTGCTTCAACCTCGCCGCAGACATGGGCGGCATGGGCTTCATCCAGAGCAACCACTCCGTCATCTTCTACAACAACGTCATGATCTCTTTCAACGTCATGGAAGCGTGCCGCGTCGAGGGCGTCACGCGCGTCTTCTACGCCTCCTCCGCGTGCATCTATCCCGAGGGCGCGCAGCTCACGACC---GAGGCGCGTCTCTCCGCG---GGGCTGAAAGAGGCCGACGCGTGGCCGGCGCAGCCGCAGGACGCGTACGGCCTCGAGAAGCTCGCGTCCGAGGAGGTCTACAAGCACTACCAGAGCGACTTTGGGATCCAGACCAGGATCGCGAGGTTCCACAACATCTACGGCCCGTTCGGGACGTGGAAGGGGGGCCGCGAGAAAGCGCCCGCGGCGTTTTGCCGCAAAGCCGCGACGGCGACGACGGAGGTGGAGATGTGGGGCGACGGCCTCCAGACGCGGTCGTTCACGTACATCGACGACTGCGTCGAGGGGATCGTCCGCCTCACGAAGTCCGACTTCTGCGAGCCCGTCAACTTGGGCTCGGACGAGATG---------------------GCGCTCGCGCTCGGCTTCGCGGGTAAGCCGGACATGCCCATCAAGCACATCCCCGGTCCCGAGGGCGTCCGCGGCCGCAACTCCAACAACGACCTCATCAAGGAGAAGCTCGGGTACGCGCCGTCCGTGCCGCTCGCGGAAGGCCTGAAGGTGACGTTCGAGTGGATCAACGAGAAGATCGAGGAGGAGGTGAAGGGTGGCGCG---AACGCGGAGGAGGCGTTCAGCAAGTCGACCATCTGCGGGACGATGGCGCCGACGGAGCTCGGCGCGTTGCGCGCCGCGGACGGCCAGGAAGGCCTCAAGGCGAAG------------------------------------------------------------

>MpGME

ATGGCT---TCGAACGGAGTGAAT---GGAACC---ACCAAT------------------GGGCTGTACGAGGCC---ACAAACTTGGACAAGGAGCTGTACTGGCCCGAGAAGAAGTTGCGAATTTCTGTGACAGGTGCCGGGGGTTTCATCGCCTCGCACATTGCCAGGAGGCTCAAGAGCGAGGGGCATTACGTGATTGCTTCCGATTGGAAGAGGAACGAGCACATGACCGAGGATATGTTCTGTGATGAGTTCCATCTGGTCGACCTCAGGGTCATGGAAAATTGTTTGAAGGTCACACAGGATGTGGAACATGTGTTCAACTTGGCCGCCGACATGGGAGGCATGGGATTCATTCAGTCCAACCACTCGGTCATCATGTACAACAATACCATGATCAGCTTCAACATGCTCGAAGCCGCCCGCATCAACGGTGTTAAGAGATTCTTCTACGCATCAAGCGCATGCATTTACCCAGAGTACAAGCAGTTGGACGTC---GACTGT---GTC---------AGCCTGAAGGAAGCCGACGCCTGGCCCGCTGAGCCACAAGATGCCTACGGTTTGGAAAAGCTTGCGACCGAAGAGCTGTGCAAGCATTACACCAAGGACTTCGGAATGCAGTGTAGAATTGGACGATTCCACAACATCTACGGCCCCTACGGAACCTGGAAGGGAGGACGTGAGAAAGCCCCTGCTGCTTTCTGCAGAAAGGCGATCACCTCCACCGAGAAGTTCGAAATGTGGGGAGATGGCAAGCAGACCAGATCATTCACATTCATTGACGAATGTGTCGAGGGTGTCCTCCGATTGACCAAGTCAGAGTACTCTGAGCCAGTAAACATCGGAAGTGACGAGATGGTGAGCATGAACGAGATGGCAGAGATCGTGCTGAGCTTCGAGGGTAAG---GACTTGCCGATCGAACACATCCCCGGACCCGAGGGAGTGAGGGGAAGGAACTCCGACAACACTTTGATCAAGGAGAAATTAGGATGGTCTCCGACAATGAGGCTGAAGGACGGTCTTAGAATCACATACTACTGGATCTTAGAGCAGGTCGAGAAGGAGAAGGCTAAGGGTGTG---ACTATGGAC---ACGTACGCCACATCGAAGGTCGTGGGCACGCAGGCACCCGTAGCGTTGGGCTCCCTGCGCGCAGCCGACGGCAAGGAG---------------------------------------------------------------------------

>MsrGME-1

ATGTCC---GCTGCC---------------------GGGTAC---------GAGCTCCGCGGCGTCTACGGCACC------------------GAGCCCTACTGGCCGGAGAAGAAGCTGAAGATCTGCGTGACCGGCGCGGGCGGCTTCATCGCCTCGCACCTCGCCAAGAGGCTCAAGGAGGAGGGTCACTACGTCGTCGGATGCGACTGGAAGCGCAACGAGCACATGCCGGAGGAGATGTTCTGCGACGAGTTCATCCTCGCCGATCTCCGCCTCTTCGAGAACTGCCAGAAGGTTCTCAAGGGCTGCGACCACTGCTTCAACCTCGCCGCGGACATGGGCGGCATGGGCTTCATCCAGTCCAACCACTCCGTGATCTTCTACAACAACGTCATGATATCCTTCAACGTGATGGAGGCGTGCCGCGTCGAGGGCGTCACCCGCGTCTTCTACGCCTCCTCCGCGTGCATCTACCCCGAGGGTGCGCAGCTCACCACC---GAG------CTCTCCGCC---GGGCTCAAGGAATCCGACGCGTGGCCCGCGCAGCCCCAGGACGCGTACGGCCTCGAGAAGCTCGCGTCCGAGGAGGTGTACAAGCACTACCAGTCCGACTTTGGCATCCAGACCCGCATCGCGCGCTTCCACAACATCTACGGCCCGTTCGGCACCTGGAAGGGCGGCCGCGAGAAGGCGCCAGCCGCGTTTTGCCGCAAGGCGGCCACCGCCACCACCGAGGTGGAGATGTGGGGCGACGGCAAGCAGACGCGTTCGTTCACCTACATCGACGACTGCGTCGAGGGTATCATCCGTCTCACGAAGTCCGACTTCGCCGAGCCCGTCAACCTCGGGTCCGACGAGATGGTGAGCATGAACGAGATGCAGGCGCTGGCGCTGGGTTTCGCGGGCAAGCAGGACATGCCCATCAAGCACATCCCGGGCCCCGAGGGCGTGCGCGGCCGCAACTCCAACAACGACCTCATCAAGGAGAAGCTCGGGTACGCGCCTTCCGTCAAGCTCGCGGACGGCCTCAAGGTGACCTACGAGTGGATCGAGGGGAAGATCAAGGAGGAGGTGGCGGCGGGTGCC---AACGCCGAGGAGGCGTTCTCCAAGTCCACCATCTGCGGCACCATGGCGCCCACCGAGCTCGGCGCGCTCCGCGCCGCCGACGGGCAAGAGAACCTCGCA---AAG------------------------------------------------------------

>MsrGME-2

ATGTCT---GCAGCT---------------------GGTTTC---------AAGCTTCGCGGCATCTACGGAACC------------------GAGCCGTACTGGCCCAAGAAAAAGCTAAAAATATGTGTCACCGGCGCCGGCGGATTCATAGCTTCACATCTCGCGCAGAGGCTGAAAGAAGAGGGACACTTCGTCGTTGGCTGTGACTGGAAGCGAAACGAGCATATGCCGGAGGAGATGTTCTGCGACGAGTTCATCCTCGCGGACCTTCGCCTTTTCGAGAACTGCCAGAATGTTCTCAAGGGCTGCGACCACTGCTTCAACCTCGCCGCGGACATGGGCGGCATGGGCTTCATCCAGTCAAACCACTCCGTCATCTTCTACAACAACATCATGATATCTTTCAACATGATGGAGGCCTGCCGCGTCGAGGGCATCACCCGCGTCTTCTACGCCTCATCCGCGTGCATCTACCCCGAGGGTGCGCAGCTCACCACC---GAC------CTCTCCGCA---GGTCTCAAGGAGGCTGACGCCTGGCCCGCGCAGCCCCAGGACGCCTACGGACTCGAGAAACTCGCTTCCGAGGAGGTGTACAAGCACTACCAGTCTGACTTTGGCATCCAGACACGCATCGCGCGCTTCCACAACATCTACGGCCCCTTCGGTACCTGGAAAGGCGGCCGCGAGAAGGCGCCCGCTGCGTTTTGCAGAAAAGCCGCGACGGCGACGACCGAGGTTGAGATGTGGGGCGACGGAAAGCAGACCCGCTCTTTCACCTACATCGACGATTGCATCGAGGGTATCCTCCGCTTGACCAAGTCCGACTTCGCCGAGCCCGTCAACCTGGGTTCTGACGAGATGGTGAGCATGAACGAGATGCAGGCGCTGGCGCTCGGATTCGCGGGCAAGCCGAACATGCCCGTGAAGCACATCCCGGGGCCTGAGGGCGTGCGCGGTCGCAACTCCAACAACGACCTCATCATGGAGAAGCTCGGCTACGCGCCCTCCGTCAAGCTCGCGGACGGCCTCAAAGTGACCTACGAGTGGATCGAGGCGAAGATCAAGGAGGAGGTGGCAGACGGCGCC---GACGCGGAGGCTGCGTTCTCCAAGTCCACCATCTGTGGCACCATGGCGCCCACCGAGCTTGGCGCCCTCCGCGCCGCCGACGGGGCCGAGAATCTGAAG------------------------------------------------------------------

>MtGME-1

ATGGGA---AGTTCT------GGAATAAACAAC---GACTAT------------------GGTGCATTCACATAC---CAAAACCTTGAGAGAGAACCATATTGGCCAACAGAAAAGCTAAGAATTTCCATTACCGGTGCTGGTGGGTTTATTGCCTCCCACATTGCTCGCCGTCTTAAGACCGAGGGCCATTACATTATTGCCTCTGATTGGAAGAAGAATGAGCACATGACTGAGGACATGTTCTGTCATGAGTTCCATCTTGTTGATCTTAGGGTTATGGATAATTGCCTCAAAGTTACTAAGGATGTGGATCATGTTTTCAATCTTGCTGCTGATATGGGTGGGATGGGTTTTATCCAATCCAATCACTCTGTCATTATGTATAACAACACAATGATTAGCTTCAATATGATTGAAGCTGCTAGGATTAATGGTGTTAAGAGGTTTTTTTATGCCTCTAGTGCTTGTATCTACCCTGAATTCAAACAGTTGGAAACA---AAT------GTG---------AGCTTGAAGGAGGCTGATGCCTGGCCTGCTGAGCCACAAGATGCATATGGGCTGGAGAAGCTTGCAACAGAAGAGTTATGCAAGCATTATAACAAAGATTTTGGAATTGAGTGCCGCATTGGGCGGTTCCATAACATATACGGCCCTTTTGGTACATGGAAAGGTGGAAGGGAAAAGGCTCCTGCTGCTTTTTGCCGAAAGACACTTACTTCCACAGATAAATTTGAGATGTGGGGAGATGGATTACAGACACGATCCTTCACCTTCATTGACGAGTGTGTTGAAGGCGTGCTTAGATTGACTAAATCAGACTTTCGGGAGCCAGTGAATATTGGAAGTGACGAAATGGTTAGCATGAATGAGATGGCTGAGATTGTTCTTAGCTTTGAGAACAAA---AGCATACCCATCCAACACATTCCTGGTCCAGAGGGTGTTCGTGGCCGCAATTCAGACAATACACTTATCAAAGAGAAACTTGGCTGGGCTCCAACAATGAAGTTGAAGGATGGGCTGAGAATTACATACTTCTGGATTAAAGAGCAGCTTGAGAAAGAGAAGGCTGGAGGTGTT---GATGTAACA---TCCTATGGATCGTCTAAAGTGGTATCGACTCAAGCCCCTGTTCAACTAGGTTCACTTCGGGCTGCAGATGGCAATGAA---------------------------------------------------------------------------

>MtGME-2

ATGGGA---AGCACT------GAA---AAAACT---AACTAT------------------GGAGAATACACCTAT---GAGAATCTCGAGAGAGAGCCTTACTGGCCATCAGAAAAGCTTAAAATTTCCATCACTGGTGCTGGGGGTTTTATCGCGTCACACTTAGCGCGGCGTCTCAAGAAGGAGGGACATTACATTATTGCTTCTGACTGGAAGAAAAATGAGCACATGACTGAGGACATGTTCTGTGATGAATTCCATCTTGTTGATCTCAGGGTCATGGATAACTGCTTAACCGTTACTAAAGGGGTTGACCATGTTTTCAATCTTGCCGCTGATATGGGTGGAATGGGTTTTATTCAGTCCAATCATTCTGTTATTATGTATAACAACACAATGATTAGTTTCAATATGATTGAAGCTGCTAGGATTAATGGCATTAAGAGGTTCTTTTATGCCTCAAGTGCTTGTATCTACCCTGAATTTAAACAGTTAGAAACTACTAAT------GTG---------AGCTTGAAGGAGTCTGATGCATGGCCTGCTGAGCCACAAGATGCGTATGGGCTAGAGAAGCTTGCAACAGAGGAGATTTGCAAGCACTATAACAAAGATTTCGGAATTGAGTGCCGCATTGGGAGGTTCCATAACATATATGGTCCTTTTGGAACATGGAAAGGTGGAAGGGAGAAGGCTCCTGCTGCTTTTTGTCGGAAAGCAATCACATCCACAGACAAATTTGAGATGTGGGGAGATGGTTTGCAAACACGATCATTCACCTTCATCGATGAGTGTGTTGAAGGTGTACTTAGACTGACTAAATCCGATTTCCGCGAGCCGGTAAATATTGGAAGTGATGAGATGGTCAGCATGAATGAGATGGCTGAGATTGTTCTTGGTTTTGAGGACAAG---AAGACTCCTATACATCACATTCCTGGCCCAGAGGGTGTTCGTGGTCGTAACTCAGACAATACACTAATAAAAGAGAAACTTGGCTGGGCTCCAACAATGAAGTTGAAGGATGGGCTGAGGATTACATATGTCTGGATTAAGGAACAACTTGAAAAGGAGAAGGCTCAAGGTCTT---GATACATCA---GGGTACGGCTCATCAAAAGTGGTTTCAACCCAAGCACCAGTCCAATTAGGCTCACTTCGGGCAGCTGACGGCAAAGAGGGGAGTAGT------------------------------------------------------------------

>OlGME

ATGGCG---GCCGCC---------------------GGGTAC---------GAATTGCGCGGGATTTACGGCACG------------------GAGGAGTACTGGCCGGAGAAGAAGCTGAAGATTTGCGTGACGGGCGCGGGAGGTTTCATCGGGTCGCATCTCGCGAAACGATTGAAAGAGGAGGGACATCACGTCGTGGCGTGCGATTGGAAGCGCAATGAACACATGGAAGAGGCGATGTTCTGCGATGAGTTCATCTTGGCTGATTTGAGGCTGTACGAAAACTGTAAAAAGGTTCTCGAGGGGTGCGACCACTGCTTCAACCTCGCGGCGGACATGGGAGGGATGGGATTCATTCAGTCCAACCACTCCGTCATCTTCTACAACAACGTGATGATTTCCTTCAATATGATGGAAGCGATGCGGGTGCAGGGCGTGACGCGATGCTTTTACGCGTCGAGCGCGTGCATCTACCCGGAGGGCACGCAGTTGAGCACG---GAG------ATGCAAGAC---GGGTTGAAGGAAGCGAGCGCGTGGCCGGCGCAGCCGCAAGACGCGTATGGTCTCGAAAAGCTCGCGAGCGAGGAAGTGTACAAGCACTACCAGCAAGATTTTGGTATTCAGACGCGCATCGGTCGATTCCACAACATTTACGGTCCGTACGGCACGTGGAAGGGCGGTCGCGAAAAGGCGCCGGCGGCGTTCTGCCGTAAGGCTGCGACGGCTGAAAGCGAAGTCGAAATGTGGGGTGACGGTAAGCAAACGCGCTCTTTCACCTACATCGACGATTGCGTCGAGGGCATCTTGCGTCTCACCAAGAGCGACTTCGCCGAGCCGGTGAACATCGGTTCCGACGAAATGATCTCCATGAACGATATGCAAGCCATGACGTTGAAGTTCGCGGGCAAG---GACTTGCCAATCAAGCATATTCCGGGTCCGGAAGGTGTGCGCGGTCGCAACTCCAACAACGAACTCATCAAGGAAAAGCTCGGTTGGGCGCCGTCTGTCAAGCTCGCGGACGGCTTGAAGGTTACGTTTGAGTGGATCTCGAGCAAGATTGCCGAAGAGAAGGCCAAGGGTGTT---GACACCGCCGCCGCTTTCGGTAAGTCCACCATCTGTGGCACGCAAGCGCCGACCGAACTCGGTCAGTTGCGCGCTGCGGACGGCGACGAAAAGCTG---------------------------------------------------------------------

>OsGME-1

ATGGGG---AGCTCGGAGAAGAAC---GGAACT---GCTTAT------------------GGCGAGTACACCTAT---GCTGAACTGGAGAGGGAGCAGTACTGGCCGTCTGAGAAGCTGAGGATATCGATCACCGGAGCTGGTGGTTTCATTGGATCCCACATTGCTCGCCGTCTGAAGAGCGAGGGGCATTACATCATCGCCTCCGACTGGAAGAAGAATGAGCACATGACTGAGGACATGTTCTGCCATGAGTTCCACCTTGTTGACCTTAGGGTCATGGACAACTGCCTCAAGGTCACCAACGGCGTCGACCATGTGTTCAACCTTGCCGCTGATATGGGTGGTATGGGGTTCATTCAGTCCAACCACTCTGTGATCATGTACAACAACACCATGATCAGTTTCAACATGCTCGAGGCTGCACGTATCAATGGTGTGAAGAGGTTCTTCTATGCCTCAAGTGCATGCATTTACCCTGAATTCAAGCAGCTTGAAACT---AAC------GTT---------AGCCTGAAGGAATCTGATGCCTGGCCTGCTGAGCCTCAAGATGCCTATGGTTTGGAGAAGCTTGCAACTGAGGAGCTCTGCAAGCACTACACCAAGGACTTTGGCATTGAGTGCCGTGTTGGCCGCTTCCACAACATATATGGCCCCTTTGGAACATGGAAAGGTGGCCGTGAGAAGGCACCAGCTGCATTCTGCAGGAAGGCTCAGACTTCCACTGACAGGTTTGAGATGTGGGGTGATGGCCTCCAGACCCGGTCCTTCACATTCATAGATGAGTGTGTTGAGGGTGTTCTGAGGTTGACAAAGTCGGACTTCCGTGAGCCAGTGAACATTGGAAGCGATGAAATGGTAAGCATGAACGAGATGGCTGAAATCATTCTCAGCTTCGAGGATAGG---GAGCTGCCCATCCACCACATCCCTGGACCCGAGGGTGTCCGTGGCCGTAACTCCGACAACACCCTCATCAAGGAGAAGCTTGGCTGGGCACCCACAATGAAGCTCAAGGACGGGCTGAGGTTCACCTACTTCTGGATCAAGGAGCAGATAGAGAAGGAGAAGACCCAGGGCGTC---GACATCGCG---GGTTACGGCTCATCCAAGGTGGTGTCCACCCAGGCCCCGGTTCAGCTGGGCTCCCTCCGTGCTGCCGATGGCAAGGAG---------------------------------------------------------------------------

>OsGME-2

ATGGCG------------------------------CTCAAT------------------GAGGAGTACACATAC---GTGGAGCTGGAGAAGGAGCCTTACTGGCCATTTGAGAAGCTGCGGATCTCGATTACGGGAGCTGGCGGGTTCATCGCATCCCACATCGCGAGGCGCCTCAAGAGCGAGGGCCACTACATCATTGCTTCTGACTGGAAGAAAAATGAGCACATGACTGAGGATATGTTCTGCCATGAGTTCCATCTTGTTGATTTGAGGGTGATGGACAACTGCCTCAAGGTGACCACCGGGGTTGACCATGTTTTCAACCTTGCCGCTGATATGGGAGGGATGGGATTCATTCAATCCAATCACTCTGTTATCATGTACAACAACACCATGATCAGCTTTAACATGCTTGAGGCCGCTAGGATTAATGGTGTCAAAAGGTTCTTTTATGCTTCAAGTGCTTGTATCTACCCTGAATTTAAGCAGCTGGACACT---GTA------GTT---------AGCTTGAAGGAGTCAGATGCTTGGCCTGCAGAGCCTCAAGATGCCTATGGCTTGGAAAAACTTGCTACTGAGGAACTGTGCAAGCATTACACAAAGGATTTTGGCATTGAATGTCGGGTTGGCCGCTTTCATAATATCTATGGTCCCTTTGGAACATGGAAGGGTGGAAGGGAGAAGGCGCCTGCTGCTTTCTGCAGAAAAGCTCTAACCTCCACTGACCGTTTTGAGATGTGGGGAGATGGTCTTCAGACTAGATCCTTCACGTTTATTGATGAATGTGTGGAGGGTGTCCTCAGGTTGACAAAGTCCGATTTCCGTGAGCCTGTAAACATTGGAAGTGATGAAATGGTCAGCATGAATGAGATGGCTGAGATAGTCCTCAGCTTTGAGAACAAG---CAGCTGCCCATCCACCACATCCCCGGGCCAGAGGGTGTCCGTGGTCGCAACTCCGACAACACGCTCATCAAAGAGAAGCTTGGCTGGGCTCCAACCATGAGGCTGAAGGATGGTCTGAGGATCACATACTTCTGGATCAAGGAGCAGCTCGAGAAGGAGAAGGCCGAGGGCGTC---GACTTGTCG---GCCTACGGGTCATCCAAGGTAGTGCAGACTCAGGCGCCGGTGCAGCTTGGCTCCCTCCGTGCTGCTGATGGAAAGGAG---------------------------------------------------------------------------

>OtGME

ATGGGT---AGCAGCGAGAAGACC---GTTTCC---GCTTAT------------------GGCGAGTACACATAT---GCTGAGCTGGAGAGGGAGCCCTACTGGCCATCTGAGAAGCTGAGGATATCAATTACAGGGGCCGGTGGTTTCATTGGATCCCACATTGCCCGTCGTCTGAAAAGCGAGGGGCATTACATCATCGCCTCCGACTGGAAGAAGAATGAACACATGACTGAGGACATGTTCTGCCATGAGTTCCACCTGGTTGACCTCAGAGTCATGGACAACTGTTTGAAGGTTACCAACGGTGTTGATCATGTCTTCAATCTTGCTGCTGATATGGGTGGCATGGGGTTCATTCAGTCAAACCACTCCGTCATCATGTACAACAACACCATGATCAGTTTTAACATGCTTGAGGCTGCCCGTATCAACGGTGTGAAGAGGTTCTTCTATGCCTCCAGTGCATGTATTTACCCGGAATTCAAACAGCTTGACACA---AAT------GTG---------AGCTTGAAGGAATCTGATGCCTGGCCTGCTGAGCCTCAAGATGCTTATGGCTTGGAGAAGCTTGCAACTGAGGAGCTGTGCAAGCATTACACCAAGGACTTTGGCATTGAGTGCCGTGTTGGTCGCTTCCACAACATATATGGTCCCTTCGGAACATGGAAAGGTGGTCGTGAGAAGGCACCAGCTGCCTTCTGCAGAAAAGCTCAGACTTCCACTGATAGATTTGAGATGTGGGGTGACGGTCTGCAGACCCGGTCCTTCACTTTTATTGACGAGTGTGTTGAGGGTGTTCTGAGGTTGACAAAGTCAGACTTCCGTGAGCCAGTGAACATTGGAAGTGATGAAATGGTTAGCATGAATGAGATGGCTGAAATTGTTCTCAGCTTTGAGGATAGG---AAGCTGCCCATCCACCACATCCCTGGCCCAGAGGGGGTTCGTGGGCGTAACTCTGACAACACCCTTATCAAGGAGAAGCTTGGCTGGGCCCCGACAATGAAGCTCAAGGATGGGCTGAGGTTCACCTACTTCTGGATCAAGGAGCAGATTGAGAAGGAGAAGACGCAGGGGGTC---GACATCGCT---GCGTACGGGTCGTCCAAGGTGGTGTCCACCCAGGCTCCAGTGCAGCTGGGCTCTCTCCGTGCCGCCGACGGCAAGGAGGGCCTT---------------------------------------------------------------------

>PhGME-1

ATGGGG---AGCAGCGAGAAGACT---GTTACC---GCTTAT------------------GGCGAGTACACCTAT---GCTGAGCTGGAGAGGGAGCCCTACTGGCCAAGTGAGAAGTTGAGGATTTCGATTACTGGGGCTGGTGGTTTCATTGGATCCCACATTGCTCGCCGTCTGAAGAGCGAGGGCCATTATATCATTGCCTCTGACTGGAAGAAGAATGAGCACATGACAGAGGACATGTTCTGCCATGAGTTCCACCTGGTTGACCTTAGGGTCATGGACAACTGTTTGAAGGTTACCCAAGGTGTCGACCATGTATTCAATCTTGCTGCTGATATGGGTGGCATGGGGTTCATCCAGTCAAACCACTCTGTGATCATGTACAACAACACCATGATCAGTTTCAACATGCTCGAGGCTGCACGTATCAATGGTGTGAAGAGGTTCTTCTACGCCTCGAGTGCATGCATTTACCCTGAATTCAAGCAGCTTGACACA---AAT------GTG---------AGCCTGAAGGAGTCCGATGCCTGGCCTGCTGAGCCTCAAGATGCCTACGGCTTGGAGAAGCTTGCAACTGAGGAGTTGTGCAAGCACTACACCAAGGACTTTGGCATTGAGTGCCGCATTGGCCGTTTCCACAACATCTATGGCCCCTTCGGAACATGGAAAGGTGGTCGTGAGAAGGCACCTGCTGCCTTCTGCAGAAAGGCTCAGACATCCACTGAGAGGTTTGAGATGTGGGGCGATGGCCTCCAGACCCGATCCTTCACTTTCATCGATGAGTGCGTCGAGGGCGTTCTGAGATTGACCAAGTCCGACTTCCGCGAGCCGGTGAACATCGGGAGCGACGAGATGGTGAGCATGAACGAGATGGCTGAGATCGTGCTGAGCTTTGAGGATAGG---AAGCTGCCCATCCACCACATCCCCGGTCCAGAGGGGGTCCGTGGGCGCAACTCTGACAACACCCTTATCAAGGAGAAGCTCGGGTGGGCCCCGACAATGAGGCTCAAGGACGGGCTTCGGTTCACCTACTTCTGGATCAAGGAGCAGATCGAGAAGGAGAAGACCCAGGGCATC---GACGTGGCG---GCGTACGGGTCCTCCAAGGTGGTGTCGACCCAGGCGCCCGTGCAGCTGGGCTCCCTCCGCGCCGCCGACGGCAAGGAGGGCCTC---------------------------------------------------------------------

>PhGME-2

ATGGGG---AGCAGCGAGAAGACT---GTCACC---GCTTAT------------------GGTGAGTACACCTAT---GCTGAGCTGGAGAGGGAGCCCTACTGGCCAAGTGAGAAGCTGAGGATTTCGATTACTGGGGCTGGTGGTTTCATTGGATCCCACATTGCTCGCCGTCTCAAGAGCGAAGGCCATTATATCATCGCCTCTGACTGGAAGAAGAATGAGCACATGACTGAGGATATGTTCTGCCATGAGTTCCACCTTGTTGACCTCAGGGTCATGGACAACTGTCTCAAGGTTACCCAAGGCGTCGACCATGTATTCAATCTTGCTGCTGATATGGGTGGCATGGGGTTCATCCAGTCAAACCACTCTGTCATCATGTACAACAACACCATGATCAGTTTCAACATGCTTGAGGCTGCACGCATCAATGGTGTGAAGAGGTTCTTCTATGCTTCGAGTGCATGCATTTACCCTGAATTCAAGCAGCTTGACACA---AAT------GTG---------AGCTTGAAGGAATCTGATGCCTGGCCTGCTGAGCCTCAAGATGCCTATGGCTTGGAGAAGCTTGCAACTGAGGAGCTCTGCAAGCACTACACCAAGGACTTTGGCATCGAGTGCCGCGTTGGCCGTTTCCACAACATCTATGGCCCCTTTGGAACATGGAAAGGTGGTCGTGAGAAGGCACCTGCTGCCTTCTGCAGAAAGGCTCAGACATCTACCGAGAGGTTTGAGATGTGGGGCGATGGCCTCCAGACACGATCATTCACTTTCATCGACGAGTGCGTCGAGGGTGTCCTGAGGTTGACCAAGTCAGACTTCCGCGAGCCGGTGAACATCGGGAGCGACGAGATGGTGAGCATGAACGAGATGGCCGAGATCGTCCTGAGCTTTGAGGATAGG---AAGCTGCCCGTCCACCACATCCCTGGCCCGGAGGGGGTCCGCGGGCGCAACTCCGACAACACCCTCATCAAGGAGAAGCTTGGCTGGGCCCCGACCATGAAGCTCAAGGACGGGCTGAGGTTCACCTACTTCTGGATCAAGGAGCAGATCGAGAAGGAGAAGACGCAGGGGGTC---GACGTCGCG---GCGTACGGGTCCTCCAAGGTGGTGTCCACCCAGGCGCCCGTGCAGCTGGGCTCCCTCCGCGCCGCCGACGGCAAGGAGGGCCTC---------------------------------------------------------------------

>PhGME-3

ATGGCG------------------------------CTCAAC------------------AAGGAGTACACGTAC---GCGGAGCTGGAGAAGGAGCCCTACTGGCCATTTGAGAAGCTGCGGATCTCCATCACGGGCGCCGGCGGGTTCATCGCCTCCCACATTGCGAGGCGCCTCAAGAGCGAGGGCCACTACATCATCGCCTCTGACTGGAAGAAGAACGAGCACATGACTGAGGAGATGTTCTGCCACGAGTTCCATCTCGTTGACCTGAGGGTCATGGACAACTGCCTCAAGGTGACCACGGGGGTCGACCATGTGTTCAACCTTGCTGCTGATATGGGTGGGATGGGGTTCATCCAGTCCAACCACTCTGTGATCATGTACAACAACACCATGATCAGCTTTAACATGCTTGAGGCTGCTAGAATTAATGGCGTCAAGAGGTTCTTTTATGCATCAAGCGCTTGTATTTATCCTGAATTTAAGCAGTTGGAGACT---GTA------GTT---------AGCTTGAAGGAGTCAGATGCCTGGCCTGCAGAGCCTCAAGATGCCTATGGCTTGGAGAAACTTGCTACTGAGGAACTGTGCAAGCACTACACAAAGGATTTTGGCATTGAGTGCCGGATCGGTCGCTTTCACAACATATATGGTCCCTTTGGAACATGGAAAGGTGGAAGGGAGAAGGCACCTGCTGCTTTCTGCAGAAAGGCTTTAACCTCCACAGGGCGCTTTGAGATGTGGGGAGATGGTCTGCAAACCAGATCCTTCACATTTATTGATGAATGTGTCGAGGGTGTCCTTAGGTTAACAAAGTCTGATTTCCGTGAGCCTGTAAACATTGGAAGTGATGAGATGGTCAGTATGAATGAGATGGCCGAGATAGTCCTCAGCTTTGAGAACAAG---CAGCTGCCCATCCACCACATTCCTGGGCCAGAGGGTGTGCGTGGGCGCAACTCAGACAACACACTCATCAAGGAGAAGCTTGGCTGGGCTCCGACCATGAAGCTGAAGGACGGACTGAGGATAACATACTTCTGGATCAAGGAGCAGCTTGAGAAGGAGAAGGCTGAGGGCATG---GACCTATCG---GTCTACGGATCATCCAAGGTCGTGCAGACTCAGGCCCCTGTTCAGCTCGGTTCCCTCCGTGCTGCTGATGGCAAGGAG---------------------------------------------------------------------------

>PhpGME-1

ATGGCG---AGTAAT------GGC------------AGCTTC------------------GGTGACTACACGGCC---ACGAACTTGGACCGGGAAGAGTACTGGCCCTCCCAAAAGCTGAGAATTTCTATCACGGGAGCTGGTGGGTTTATTGCCTCGCACATCGCGCGTCGATTGAAGAGCGAGGGGCACTACATAATCGCCTCCGACTGGAAGAAGAATGAGCACATGAGTGAGGATATGTTCTGCGACGAGTTTCACCTCGTGGATCTGCGTGTCATGGACAATTGCATGAAGGTCACTAAGGGCGCTCATCATGTGTTCAACTTGGCTGCCGACATGGGTGGTATGGGCTTCATCCAGTCTAACCACGCCGTCATTATGTACAACAACACCATGATCAGTTTCAACATGCTCGAAGCCGCTCGCATCAACGGTGTTACCCGGTTTTTCTATGCCTCAAGCGCGTGCATTTACCCTGAATTCAAGCAACTCGAAACT---GAT------GTGAGC------AGTTTGAAGGAGTCTGATGCGTGGCCCGCTCTGCCCCAAGATGCTTACGGTCTTGAAAAGTTGGCCACCGAGGAGTTGTGCAAACATTACACGAAGGACTTTGGCATGGAGTGCCGCATTGGTCGCTTTCACAACATTTATGGCCCATACGGAACCTGGAAGGGTGGGCGTGAGAAGGCACCCGCCGCCTTCTGCAGAAAGGCCTTGACAGCCACCGAGCATTTCGAGATGTGGGGCGATGGCAAGCAAACTCGCTCCTTCACCTTCATCGACGAGTGTGTAGAGGGCGTCTTGCGCTTGACGAAGTCCGACTTCCAAGAGCCTGTAAATATCGGTAGCGATGAGATGGTGAGCATGAACGAGATGGCCGAGATTGTGCTCAGCTTCGACAACAAG---CAGCTCCCGATCAAGCACATTCCCGGACCCGAGGGAGTGCGGGGACGAAACTCTGATAACACTCTGATCAAGGAGAAGCTCGGCTGGGCTCCATCCATGCGACTCAGGGACGGACTCGCCATCACCTACAAGTGGATCAAGGAGCAGATTGAGAAGGAGAAGGAGTCCGGTGCA---GACCTTGCATCCAAGTATGGATCTTCCAAGGTGGTCGGTACCCAAGCTCCAGTGCAGCTGGGCTCTTTGAGAGCTGCTGATGGCAAGGAG---------------------------------------------------------------------------

>PhpGME-2

ATGGCG---AGCTAT------GGC------------CGCTTC------------------GGTGACTATACCGCC---ACAAACTTGGACCGGGAGGCATACAGGCCCGCGGAGAAGCTGCGAATCTCCATCACAGGGGCTGGTGGGTTCATCGCCTCGCACATCGCGCGCCGATTGAAGAGCGAAGGGCACTACATCATTGCCTCCGACTGGAAGAAGAATGAGCACATGAGCGAGGATATGTTCTGCGACGAGTTCCACCTCGTGGATCTGCGCGTCATGGACAATTGCTTGAAGGTCACCAAGGGCGCCAATCACGTCTTCAACCTGGCCGCCGACATGGGGGGCATGGGCTTCATCCAGTCCAATCACGCTGTCATTATGTACAACAATACCATGATCAGCTTCAACATGCTTGAAGCCTCACGCATCAATGGTGTCTCTAGGTTTTTCTATGCTTCAAGCGCGTGTATCTATCCTGAATTCAAACAGCTGGAGACT---GAC------GTGAGC------AGCCTGAAGGAGTCGGACGCTTGGCCCGCTCTGCCCCAGGATGCTTATGGCCTTGAGAAGTTAGCCACGGAGGAGCTGTGTAGGCACTATACAAAGGACTTCGGCATGGAGTGTCGCATTGGTCGTTTCCACAACATCTACGGTCCCTATGGAACCTGGAAGGGGGGACGTGAGAAGGCTCCCGCTGCCTTCTGCAGGAAGGCCTTGACAGCTACCGAGTACTTTGAGATGTGGGGCGATGGCAAGCAAACCCGTTCATTCACCTTCATCGACGAGTGCGTGGAGGGCGTCTTGCGCCTGACGAAGTCCGACTTCCAGGAACCAGTAAACATCGGCAGTGACGAGATGCTGAGCATGAACGAGATGGCCGAGATTGTGCTCAGCTTTGACAACAAG---AAGCTCCCAATCAAGCACATCCCCGGACCGGAGGGAGTGCGCGGGCGGAACTCAGACAACACTTTGATCAAAGAGAAGCTCGGCTGGGCTCCCTCCATGCGCCTCAGGGACGGGCTTGCCATCACATACAAATGGATCAAGGAGCAGATCGAGAAAGAGAAGGAATCGGGTGCC---GATCTTGCATCCAAATATGGATCCTCCAAGGTGGTCGGCACCCAGGCCCCAGTGCAGCTGGGCTCCTTGAGAGCCGCCGACGGCAAGGAG---------------------------------------------------------------------------

>PhpGME-3

ATGGCGGAGAGCAAT------------GGGACG---AGCTTC------------------GGCAACTACACGGCC---ACGAACTTGGACCGCGAGCTGTATTGGCCGAACCAGAAGCTGCGAATCTCCATCACGGGCGCCGGAGGGTTCATTGCGTCGCACATCGCGCGCCGATTGAAGAGCGAGGGCCACTACATCATCGCCTCTGACTGGAAGAAGAATGAACATATGAGTGAGGATGCGTTCTGCGATGAGTTCCACCTCGTTGACCTGCGCGTCATGGACAATTGCCTCAAGGTCACCCAGGGCGCGCACCACGTCTTCAACCTCGCGGCAGACATGGGCGGCATGGGTTTCATTCAGTCCAACCACGCCGTGATCATGTACAACAACACCATGATCAGCTTTAACATGCTCGAGGCCGCGCGCATCAATGGCGTCTCTCGGTTTTTCTATGCCTCAAGCGCGTGTATTTACCCTGAGTTCAAGCAGCTGGAGACT---GAT------GTGAGC------TCTTTGAAAGAATCGGATGCTTGGCCTGCTCTGCCCCAGGATGCCTACGGACTCGAGAAACTGGCCACAGAGGAGTTGTGCAAGCATTACACAAAGGACTTCGGCATGGAGTGCCGAATTGGGCGTTTCCACAACATCTACGGACCCTACGGAACATGGAAGGGGGGACGCGAGAAAGCGCCAGCTGCCTTCTGCCGAAAGGCCTTAACAGCTACGGAGTACTTCGAGATGTGGGGCGATGGCAAGCAAACCCGATCCTTCACCTTCATCGACGAATGCGTGGAGGGAGTCTTACGGTTGACGAAGTCCGATTTCCAGGAGCCAGTGAACATCGGAAGCGACGAGATGGTGAGCATGAACGAGATGGCTGAGATCGTGCTCAGCTTCGACAACAAG---AAACTTCCGATCAAGCACATTCCGGGGCCGGAAGGTGTGCGGGGGCGAAACTCGGACAACACACTGATTAAGGAGAAGCTGGGCTGGGCACCCTCGATGCGCCTGATGGACGGGCTGGCTATCACATACAAATGGATCAAGGAGCAGATCGACAAGGAGAAGGAATTGGGCACC---GAGCTCGCGTCCAAGTATGGCACCTCTATGGTGGTGGGCACGCAGGCTCCAGTGCAGCTCGGCTCCTTGCGAGCTGCGGACGGCAAGGAA---------------------------------------------------------------------------

>PhvGME-1

ATGGGA---AGTTCC------GGA---GCCAAC---GACTAT------------------GGAGCATACACTTAC---CAAAACCTTGAGAGGGAACCTTACTGGCCCTCTGAAAAGCTCAGAATTTCCATCACCGGGGCTGGTGGTTTCATTGCCTCACACATTGCACGCCGTCTCAAGACTGAGGGGCATTACATCATTGCTTCTGATTGGAAGAAAAATGAGCACATGACTGAAGACATGTTCTGCCATGAGTTCCATCTTGTTGATCTTAGGGTCATGGATAACTGCTTGGCAGTTACCAAGGGTGTGGATCATGTTTTCAATCTTGCTGCTGATATGGGTGGAATGGGTTTTATCCAGTCAAATCACTCAGTCATTATGTACAACAACACCATGATTAGCTTCAACATGATTGAGGCTGCTAGGATCAATGGTGTTAAGAGGTTTTTTTATGCTTCCAGTGCTTGTATCTACCCTGAGTTTAAACAGTTGGAAACA---AAT------GTG---------AGTTTGAAGGAGGCTGATGCCTGGCCTGCTGAGCCACAAGATGCATATGGGCTAGAGAAGCTTGCAACTGAAGAGTTATGCAAGCATTATAACAAGGACTTTGGAATTGAGTGCCGCATTGGGAGATTCCATAATATATATGGTCCTTATGGGACATGGAAAGGGGGAAGGGAAAAGGCTCCTGCTGCATTTTGCCGGAAGACTCTTACATCCAAAGACCAATTTGAGATGTGGGGAGATGGATTGCAAACAAGGTCCTTCACCTTCATTGATGAGTGTGTTGAAGGTGTACTCAGATTGACAAAATCAGATTTCCGGGAGCCAGTGAATATTGGAAGTGATGAAATGGTCAGCATGAATGAGATGGCTGAGATTGTTCTTAGCTTTGAGGACAAG---ACTATACCAATATACCACATTCCTGGTCCAGAAGGTGTTCGAGGACGTAATTCAGACAATACATTAATCAAAGAAAAACTTGGTTGGGCTCCAACTATGAAGTTGAAGGATGGACTGAGAATCACATACTTTTGGATCAAAGAGCAGCTTGAGAAAGAGAAGGCAGCTGGTGTT---GATTTATCA---GTGTATGGATCATCCAAAGTGGTGCAAACTCAGGCTCCTGTTCAACTGGGCTCCCTTAGGGCTGCAGATGGCAAAGAA---------------------------------------------------------------------------

>PhvGME-2

ATGGGA---AGTGCT------GGA---AAAACT---GACTAT------------------GGTGAGTACACTTAT---GAGAATCTTGAGAGAGAGCCTTACTGGCCATCAGAGAAGCTTAAGATTTCCATCACTGGGGCTGGGGGTTTTATTGCATCACACATAGCTCGGCGCCTCAAGAGGGAGGGACATTACATTATTGCTTCTGACTGGAAGAAAAATGAGCACATGACTGAGGACATGTTCTGTGATGAATTCCATCTTGTTGATCTCCGGGTCATGGATAACTGCCTGAAGGTTACCAAGGGGGTTGATCATGTTTTCAATCTTGCTGCAGACATGGGTGGGATGGGCTTTATTCAGTCTAATCACTCTGTCATTATGTACAACAACACGATGATTAGCTTCAACATGATTGAGGCTGCCAGGATTAACGGCATTAAGAGGTTTTTTTATGCCTCTAGTGCTTGTATCTACCCAGAGTTTAAACAGTTGGAAACC---AAT------GTG---------AGCTTGAAGGAGTCTGATGCATGGCCAGCTGAGCCACAAGATGCATATGGGCTGGAGAAGCTTGCAACTGAGGAGTTATGCAAGCACTATAACAAGGATTTTGGAATTGAGTGCCGTATTGGGAGGTTCCATAACATTTATGGCCCTTTTGGAACTTGGAAAGGTGGAAGGGAGAAGGCTCCTGCTGCTTTTTGTCGGAAGGTAATCACTTCCACTGATAGATTTGAGATGTGGGGAGATGGATTGCAAACACGATCATTTACCTTCATTGATGAGTGTGTTGAAGGTGTGCTAAGATTGACAAAATCTGACTTCCGTGAGCCGGTAAATATTGGAAGTGATGAGATGGTTAGCATGAATGAGATGGCTCAGATTATTCTTGGCTTTGAGAGCAAG---AATATACCTATTCATCACATTCCTGGTCCCGAGGGTGTTCGAGGTCGTAATTCAGAAAATACACTGATAAAAGAAAAACTTGGCTGGGCTCCAACTATGAAGTTGAAGGATGGGCTGAGGATCACATACTTCTGGATCAAGGAGCAGATTGAGAAGGAGAAGACTCAAGGTATT---GATATATCA---GTGTATGGGTCATCCAAAGTAGTGCAGACTCAGGCCCCAGTTCAACTAGGCTCACTTCGGGCAGCAGATGGCAAAGAA---------------------------------------------------------------------------

>PpGME-1

ATGGGA---ACTACT------GGG---GGAAGT---AAATAC------------------GGTGAATACACATAT---GAGAACCTGGAGAGGGAACAGTACTGGCCTTCCGAAAAGCTTCGGGTTTCCATAACGGGGGCTGGTGGTTTCATTGCTTCGCATATTGCCAGGCGACTGAAGAGTGAGGGTCACTACATTATTGCTTCTGACTGGAAGAAAAATGAACACATGACAGAGGACATGTTTTGTAATGAGTTCCATCTTGTTGATCTGAGGGTGATGGATAACTGTTTGAAGGTCACTTCTGGTGTTGACCATGTGTTCAACCTTGCTGCTGACATGGGAGGTATGGGTTTTATTCAGTCCAACCATTCCGTCATTATGTATAACAACACAATGATCAGCTTTAACATGCTCGAGGCAGCAAGGATCAATGGAGCTAAGAGGCTCTTTTATGCTTCTAGTGCTTGCATTTACCCTGAATTTAAACAGTTGGACACTAGTAAT------GTG---------AGCTTGAAGGAGTCTGATGCTTGGCCTGCGGAGCCTCAAGATGCTTATGGCTTGGAGAAGCTCATGACTGAAGAATTATGCAAGCACTATAATAAGGATTTTGGTATTGAGTGTCGGATCGGGCGGTTCCACAATATTTATGGACCCTTTGGAACTTGGAAAGGTGGTAGAGAGAAGGCCCCTGCTGCTTTTTGTCGAAAAACCCTCACTTCCACTGATAAGTTTGAGATGTGGGGAGATGGGCTACAAACGCGGTCTTTCACCTTCATTGATGAATGTGTAGAAGGTGTCCTAAGATTGACAAAGTCTGACTTCCGGGAGCCGGTAAATATTGGAAGTGATGAGATGGTTAGCATGAATGAGATGGCTGAGATAGTCCTAAGCTTTGAGAACCAG---AAGCTCCCCATCCATCATATCCCTGGCCCTGAGGGTGTTCGCGGACGAAACTCAGACAACACTCTGATTAAGGAGAAGCTTGGCTGGGCCCCTACCATGAAGTTGAAGGATGGATTGAGAATTACGTACTTTTGGATTAAGGAACAGTTAGCGAAAGAGAAGGCTCAAGGCATG---AATTTGTTA---GGTTATGGGTCATCAAAAGTAGTGGGAACCCAAGCACCTGTTCAACTGGGCTCTCTTAGAGCTGCTGATGGGAAAGAGTTGCTC---------------------------------------------------------------------

>PpGME-2

ATGGGG---AGTACC------GGT---GGACAT---GACTAC------------------GGTGCATACACCTAT---GAGAACCTCGAGAGGGAACCTTATTGGCCTTCAGAAAAGCTTCGAATTTCCATTACTGGGGCAGGTGGCTTTATTGCCTCACACATTGCTCGGAGATTGAAGAATGAGGGCCATTACATTATTGCTTCTGATTGGAAGAAGAACGAGCACATGACTGAAGACATGTTCTGTCATGAATTCCATCTCGTTGACCTTAGGGTTATGGATAATTGCTTGAAGGTTACCAAGAATGTTGACCATGTGTTCAACCTCGCTGCCGATATGGGTGGGATGGGCTTCATCCAGTCCAACCATTCTGTCATTTTTTACAATAATACTATGATTAGCTTCAACATGGTGGAAGCTGCTAGAATCAATGGAGTGAAGAGGTTTTTCTATGCTTCTAGTGCTTGCATTTACCCTGAGTTTAAGCAGCTGGAAACC---AAT------GTG---------AGCTTGAAGGAGTCTGATGCCTGGCCTGCAGAGCCTCAAGATGCCTATGGCTTAGAGAAGCTTGCAACTGAGGAGTTGTGCAAGCACTACACAAAGGACTTTGGAATTGAGTGTCGTATTGGAAGGTTCCATAACATTTATGGTCCTTTTGGAACCTGGAAAGGTGGGAGGGAGAAGGCACCTGCTGCTTTTTGCAGAAAGACTCTCACTTCCACTGATAAGTTTGAGATGTGGGGAGATGGACTTCAGACTCGATCCTTCACCTTCATCGATGAATGTGTAGAAGGTGTACTTCGGTTGACAAAGTCTGACTTCCGTGAGCCAGTGAATATTGGAAGTGACGAGATGGTCAGCATGAATGAGATGGCGGAGATAGTTCTTAGCTTTGAGGATAAG---AAGCTGCCTATCCAGCACATTCCTGGGCCAGAGGGTGTCCGTGGTCGTAACTCAGACAACACACTGATTAAAGAGAAACTTGGCTGGGCTCCCACCATGAGGTTGAAGGATGGGTTGAGAATTACATACTTCTGGATCAAGGAACAGATTGAGAAAGAGAAGGCACAAGGCACT---GACCTGTCG---AATTATGGGTCATCTAAGGTGGTGGGAACCCAAGCCCCAGTTCAACTTGGTTCGCTACGTGCTGCTGATGGCAAAGAA---------------------------------------------------------------------------

>PsGME-1

ATGGGA---AGCACTGGAGCTGAT---GGAGTG---GCCTAT------------------GGGGCATACACATAT---GAAGATCTGGAGAGAGAGCCATACTGGCCTAGTGAAAAAGTGATAATTTCTATCACTGGAGCTGGGGGTTTCATTGCCTCCCATATTGCCCGGAGATTGAAGTCTGAAGGGCATTACATCATTGCCTCTGATTGGAAGAAGAATGAGCACATGACTGAGGATATGTTCTGCAATGAATTCCATCTTGTGGATCTCAGAGTTATGGAAAATTGCTTGGCTGTTACCAAAGGGGTTGACCATGTTTTTAACTTGGCCGCTGACATGGGTGGTATGGGATTCATTCAATCTAATCATTCTGTTATCATGTACAACAACACAATGATCAGCTTCAACATGATTGAAGCTGCCAGAATCAATGGTGTTAAAAGGTTTTTCTATGCTTCTAGCGCATGCATTTATCCCGAATTCAAACAATTGGAGACC---AAC------GTG---------AGCTTGAAGGAGTCTGACGCTTGGCCAGCTGAGCCTCAAGATGCCTATGGCTTGGAAAAACTTGCCACAGAAGAATTGTGCAAGCACTACAACAAGGACTTTGGTATAGAATGCCGGATAGGGCGCTTCCACAACATCTATGGTCCTTTTGGCACATGGAAGGGTGGACGTGAGAAGGCACCTGCTGCCTTTTGCAGGAAGACAATCACATCCACCGATAGGTTTGAAATGTGGGGCGATGGCAAGCAAACACGATCCTTTACATTCATTGATGAATGTGTGGAAGGTGTCCTGAGATTGACTAAATCAGACTTTAGAGAGCCAGTGAATATTGGGAGTGATGAAATGGTTAGCATGAATGAGATGGCTGAAATGGTGCTGAGCTTTGAGAACAAA---AAGTTGCCAATACATCACATCCCTGGGCCAGAGGGTGTACGTGGTCGGAATTCTGATAATACATTGATCAAGGAAAAGCTAGGCTGGGCGCCAACCATGAGGCTGAAGGATGGCTTGAGGATTACTTATTTCTGGATTAAGGAACAAATTGAGAAAGAAAAGGTTCAGGGAATT---GATCTCTCC---ATCTATGGATCTTCTAAGGTGGTTGGCACTCAAGCCCCTGTTCAACTTGGTTCTCTCCGTGCTGCTGATGGAAAGGAA---------------------------------------------------------------------------

>PsGME-2

ATGGGA---AGCACTAGAGCTGAT---GGAGTG---GCCTAT------------------GGGGCATACACATAT---GAAGATCTGGAGAGAGAGCCATACTGGCCTAGTGAAAAAGTGATAATTTCTATCACTGGAGCTGGGGGTTTCATTGCCTCCCATATTGCCCGGAGATTGAAGTCTGAAGGGCATTACATCATTGCCTCTGATTGGAAGAAGAATGAGCACATGACTGAGGATATGTTCTGCAATGAATTCCATCTTGTGGATCTCAGAGTTATGGAAAATTGCTTGGCTGTTACCAAAGGGGTTGACCATGTTTTTAACTTGGCCGCTGACATGGGTGGTATGGGATTCATTCAATCTAATCATTCTGTTATCATGTACAACAACACAATGATCAGCTTCAACATGATTGAAGCTGCCAGAATCAATGGTGTTAAAAGGTTTTTCTATGCTTCTAGCGCATGCATTTATCCCGAATTCAAACAATTGGAGACC---AAC------GTG---------AGCTTGAAGGAGTCTGACGCTTGGCCAGCTGAGCCTCAAGATGCCTATGGCTTGGAAAAACTTGCCACAGAAGAATTGTGCAAGCACTACAACAAGGACTTTGGTATAGAATGCCGGATAGGACGCTTCCACAACATCTATGGTCCTTTTGGCACATGGAAGGGTGGACGTGAGAAGGCACCTGCTGCCTTTTGCAGGAAGACAATCACATCCACCGATAGGTTTGAAATGTGGGGCGATGGCAAGCAAACACGATCCTTTACATTCATTGATGAATGTGTGGAAGGTGTCCTGAGATTGACTAAATCAGACTTTAGAGAGCCAGTGAATATTGGGAGTGATGAAATGGTTAGCATGAATGAGATGGCTGAAATGGTGCTGAGCTTTGAGAACAAA---AAGTTGCCAATACATCACATCCCTGGGCCAGAGGGTGTACGTGGTCGGAATTCTGATAATACATTGATCAAGGAAAAGCTAGGCTGGGCGCCAACCATGAGGCTGAAGGATGGCTTGAGGATTACTTATTTCTGGATTAAGGAACAAATTGAGAAAGAAAAGGTTCAGGGAATT---GATCTCTCC---ATCTATGGATCTTCTAAGGTGGTTGGCACTCAAGCCCCTGTTCAACTTGGTTCTCTCCGTGCTGCTGATGGAAAGGAA---------------------------------------------------------------------------

>PsGME-3

ATGGGA---AGCATTGGAGCTGAT---GGAGTG---ACCTAT------------------GGGGAATACACCTAT---GCGAATCTGGACAGAGAGCTATACTGGCCAAGTGAAAAATTGAAAATTTCCATCACTGGAGCTGGTGGGTTCATTGCCTCCCATATTGCCCGGAGATTGAAATCTGAAGGGCACTACATTATTGCCTCTGATTGGAAGAAGAACGAGCACATGACCGAGGATATGTTCTGCAATGAATTCCATCTCGTGGATCTGAGAGTTATGGACAATTGCTTGGCTGTTACCAAAGGAGTTGACCATGTTTTCAACTTGGCCGCAGACATGGGTGGTATGGGATTCATTCAGTCTAATCACTCTGTTATCATGTACAACAACACCATGATCAGCTTCAACATGCTTGAAGCTGCCCGAATCAATGGTGTTAAAAGGTTTTTCTATGCTTCTAGTGCATGCATTTATCCCGAATTCAAACAATTGGAGACC---AAC------GTG---------AGCTTGAAGGAGTCTGATGCTTGGCCAGCTGAGCCTCAAGATGCCTATGGCTTGGAAAAGCTTGCCACAGAAGAATTGTGCAAACACTACACCAAGGACTTTGGTATAGAATGCAGGATAGGGCGTTTCCACAACATTTATGGTCCTTTTGGCACATGGAAGGGTGGACGTGAGAAAGCACCTGCTGCTTTTTGTAGGAAGACAATCACATCCACTGACAGGTTTGAAATGTGGGGCGATGGCGAGCAAACGCGATCCTTTACATTCATTGATGAATGTGTGGAGGGTGTCCTTAGGTTGACTAAATCAGACTTTAGAGAGCCAGTGAATATTGGCAGTGATGAAATGGTCAGCATGAACGAGATGGCTGAAATGGTGTCAAGCTTTGAGAACAAA---AAGTTGCCGATACATCACATCCCTGGGCCAGAGGGTGTACGTGGTCGGAATTCTGAAAATACATTGATTAAGGAAAAGCTAGGCTGGGCACCAACCATGAAGTTGAAGGATGGCTTGAGGATTACTTATTTCTGGATCAAGAAACAAATTGAGAAGGAAAAGGCTCAGGGAATT---GATCTCTCT---ATCTATGGGTCTTCCAAGGTGGTTGGCACTCAAGCCCCTGTTCAACTTGGTTCTCTTCGTGCTGCTGATGGAAAGGAA---------------------------------------------------------------------------

>PtGME-1

ATGGGA---AGTGCT------GAT---GGAAGC------TAT------------------GGTGCATACACCTAT---GAGGCCCTCGAGAGGGAGCCTTACTGGCCATCTGAAAATCTCAAAATTTCCATCACTGGAGCCGGTGGTTTTATTGCCTCCCACATTGCTCGCCGTTTGAAGTCTGAGGGTCATTATATTATTGCTTCTGACTGGAAGAAGAATGAGCACATGACAGAAGACATGTTCTGTCATGAATTCCATCTTGTTGATCTTAGGGTCATGGATAATTGCCTGAAGGTTACAAAAGGAGTTGACCATGTTTTCAACCTGGCTGCTGATATGGGCGGGATGGGCTTCATTCAGTCCAATCACTCTGTCATTATGTATAACAACACAATGATCAGCTTCAACATGCTTGAAGCTTCCAGGATCAATGGCGTTAAGAGGTTATTCTATGCCTCTAGTGCTTGTATTTACCCTGAATTTAAGCAGCTGGAGACT---AAT------GTG---------AGCCTCAAGGAATCTGATGCCTGGCCTGCAGAGCCTCAAGATGCTTATGGCTTGGAGAAGCTTGCAACGGAAGAGTTGTGCAAGCATTACACCAAAGACTTTGGAATTGAATGCCGCATTGGAAGGTTCCATAACATTTATGGTCCTTTTGGAACATGGAAAGGTGGCAGGGAGAAGGCACCCGCTGCTTTCTGCAGAAAGACTATGACTTCTATTGATAAATTTGAGATGTGGGGAGATGGACTTCAAACCCGATCCTTCACATTCATTGATGAATGTGTGGAAGGTGTGCTTAGATTGACAAAGTCAGACTTCCGTGAGCCAGTGAACATTGGAAGTGATGAGATGGTTAGCATGAATGAGATGGCTGAGATTGTTCTCAGCTTCGAGAACAAG---AATCTCCCTATTCATCACATTCCTGGCCCAGAAGGTGTTCGTGGACGTAACTCTGACAACACACTAATCAAAGAGAAGCTTGGTTGGGCTCCTACAATGAAGCTGAAGGATGGGCTGAGAATTACATACTTTTGGATCAAGGAACAGATTGAGAAAGAGAAGTCACAAGGAATG---GACTTGTCT---ATTTATGGTTCATCTAAAGTGGTGGGAACCCAAGCACCCGTTCAATTGGGCTCACTTCGTGCTGCTGATGGTAAAGAA---------------------------------------------------------------------------

>PtGME-2

ATGGGG---ACTGCT------GAC---GGAAGC------TAT------------------GGTTCTTACACCTAT---GAGGCCCTCGAGAGGGAGCCTTACTGGCCATCTGAAAAGCTCAGAATTTCCATCACTGGGGCAGGGGGTTTTATTGCCTCCCACATTGCTCGCCGTTTGAAGGCTGAGGGTCATTACATTATTGCTTCTGACTGGAAGAAGAATGAGCACATGACAGAAGACATGTTTTGTCATGAATTCCATCTTGTTGATCTGAGAGTCATGGATAATTGCTTGAAGGTTACAAAAGATGTAGACCATGTTTTCAACCTTGCTGCTGATATGGGCGGGATGGGCTTCATTCAGTCCAACCACTCTGTCATTATGTATAACAACACAATGATCAGCTTCAACATGCTTGAAGCCTCCAGGATCAATGGGGTTAAGAGGTTGTTTTATGCCTCTAGTGCTTGTATTTACCCTGAATTTAAGCAGCTGGAGACT---AAT------GTG---------AGCCTGAAGGAATCTGATGCCTGGCCTGCAGAGCCTCAAGATGCTTATGGATTGGAGAAGCTTGCAACGGAAGAGTTGTGCAAGCATTACACCAAAGACTTTGGAATTGAATGCCGTATTGGAAGATTCCATAACATTTATGGTCCTTTTGGAACATGGAAAGGTGGCAGGGAGAAGGCACCCGCTGCTTTCTGCAGAAAGGCTATCACTTCCATTGATAAATTCGAGATGTGGGGAGATGGACTTCAAACCCGATCTTTCACATTCATTGATGAGTGTGTGGAAGGTGTGCTTAGATTGACAAAGTCAGACTTCCGTGAGCCAGTGAACATTGGAAGTGATGAGATGGTTAGCATGAATGAGATGGCTGAGATTGTTCTCAGCTTTGAGAACAAG---AATCTCCCCATTCATCACATTCCTGGCCCAGAAGGTGTGCGTGGGCGTAACTCTGACAACACACTAATCAAAGAGAAGCTTGGTTGGGCTCCTACAATGAGGCTGAAGGATGGGCTGAGAATTACTTACTTTTGGATCAAGGAACAGATTGAGAAAGAGAAATCCAAAGGAATT---GACCTGTCT---ATTTATGGTTCATCGAAAGTGGTGGGAACTCAAGCACCTGTTCAATTGGGCTCGCTCCGCGCTGCTGATGGTAAAGAA---------------------------------------------------------------------------

>PtGME-3

ATGGGA---AGTGTT------GAT---GGAAGC------TAT------------------GGTGCATACACCTAT---GAGGCCCTCGAGAGGGAGCCTTACTGGCCATCTGAAAATCTCAAAATTTCCATCACTGGAGCAGGTGGTTTTATTGCCTCCCACATTGCTCGCCGTTTGAAGTCTGAGGGTCATTATATTATTGCTTCTGACTGGAAGAAGAATGAGCACATGACAGAAGACATGTTCTGTCATGAATTCCATCTTGTTGATCTTAGGGTCATGGATAATTGCCTGAAGGTTACAAAAGGAGTTGACCATGTTTTCAACCTGGCTGCTGATATGGGCGGGATGGGCTTCATTCAGTCCAACCACTCTGTCATTATGTATAACAACACAATGATCAGCTTCAACATGCTTGAAGCTTCCAGGATCAATGGCGTTAAGAGGTTATTCTATGCCTCTAGTGCTTGTATTTACCCTGAATTTAAGCAGCTGGAGACT---AAT------GTG---------AGCCTCAAGGAATCTGATGCCTGGCCTGCAGAGCCTCAAGATGCTTATGGCTTGGAGAAGCTTGCAACGGAAGAGTTGTGCAAGCATTACACCAAAGACTTTGGAATTGAATGCCGCATTGGAAGGTTCCATAACATTTATGGTCCTTTTGGAACATGGAAAGGTGGCAGGGAGAAGGCACCCGCTGCTTTCTGCAGAAAGACTATGACTTCTATTGATAAATTTGAGATGTGGGGAGATGGACTTCAAACCCGATCATTCACATTCATTGATGAATGTGTGGAAGGTGTGCTTAGATTGACAAAGTCAGACTTCCGCGAGCCAGTGAACATTGGAAGTGATGAGATGGTTAGCATGAATGAGATGGCTGAGATTGTTCTCAGCTTCGAGAACAAG---AATCTCCCTATTCATCACATTCCTGGCCCAGAAGGTGTGCGTGGACGTAACTCTGACAACACACTAATCAAAGAGAAGCTTGGTTGGGCTCCTACAATGAAGCTGAAGGATGGACTGAGATTTACATACTTTTGGATCAAGGAACAGATTGAGAAAGAGAAGTCACAAGGAATG---GACTTGTCT---ATTTATGGTTCATCTAAAGTGGTGGGAACCCAAGCGCCCGTTCAATTGGGCTCACTTCGTGCTGCTGATGGTAAAGAA---------------------------------------------------------------------------

>PvGME-1

ATGGGG---GGCAGCGAGAAGACA---GTTACC---GCTTAT------------------GGTGAGTACACCTAT---GCTGAGCTGGAGAGGGAGCCCTACTGGCCGAGTGAGAAGTTGAGGATTTCGATTACTGGGGCTGGTGGTTTCATTGGATCCCACATTGCTCGCCGTCTGAAGAGCGAGGGCCATTATATCATTGCTTCTGACTGGAAGAAGAATGAGCACATGACCGAGGACATGTTCTGCCATGAGTTCCACCTGGTTGATCTCAGAGTCATGGACAACTGTCTGAAGGTTACCCAAGGCGTCGACCATGTATTTAATCTTGCTGCTGATATGGGTGGCATGGGGTTCATCCAGTCAAACCACTCTGTCATCATGTACAACAACACCATGATCAGTTTCAACATGCTAGAGGCTGCACGTATCAATGGTGTGAAGAGGTTCTTCTACGCCTCCAGTGCATGCATTTACCCTGAATTCAAGCAGCTTGACACA---AAT------GTG---------AGCCTGAAGGAATCCGATGCCTGGCCTGCTGAGCCTCAAGATGCCTATGGCTTGGAGAAGCTTGCAACTGAGGAGTTGTGCAAGCACTACACCAAGGACTTTGGCATCGAGTGCCGCATTGGCCGTTTCCACAACATCTACGGTCCCTTCGGTACATGGAAAGGTGGTCGTGAGAAGGCACCTGCTGCCTTCTGCAGAAAGGCTCAGACATCCACCGAGAGGTTTGAGATGTGGGGCGATGGCCTCCAAACCCGATCCTTCACTTTCATCGACGAATGCGTCGAGGGTGTTCTGAGATTGACCAAGTCTGATTTCCGCGAGCCGGTGAACATTGGGAGCGACGAGATGGTGAGCATGAACGAGATGGCTGAGATCGTGCTGAGCTTTGAGGATAGG---AAGCTGCCCATCCACCACATCCCTGGTCCAGAGGGTGTCCGCGGGCGCAACTCTGACAACACCCTTATCAAGGAGAAGCTCGGGTGGGCTCCGACAATGAGGCTCAAGGACGGGCTTCGGTTCACCTACTTCTGGATCAAGGAGCAGATCGAGAAGGAGAAGACCCAGGGCATC---GACGTCGCG---GCGTACGGGTCCTCCAAGGTGGTGTCGACCCAGGCGCCCGTGCAGCTGGGCTCCCTCCGTGCCGCTGATGGCAAGGAGGGCCTC---------------------------------------------------------------------

>PvGME-2

ATGGGG---AGCAGCGAGAAGACT---GTTACT---GCTTAT------------------GGCGAGTACACCTAT---GCTGAGCTGGAGAGGGAGCCCTACTGGCCAAGTGAGAAGTTGAGGATTTCGATTACTGGGGCTGGTGGTTTCATTGGATCCCACATTGCTCGCCGTCTGAAGAGCGAGGGCCATTATATCATTGCCTCTGACTGGAAGAAGAATGAGCACATGACCGAGGACATGTTCTGCCATGAGTTCCACCTGGTTGATCTCAGGGTCATGGACAACTGTCTGAAGGTTACCCAAGGCGTCGATCATGTATTCAATCTTGCTGCTGATATGGGTGGCATGGGGTTCATCCAGTCAAACCACTCTGTCATCATGTACAACAACACCATGATCAGTTTCAACATGCTAGAGGCTGCACGTATCAATGGTGTGAAAAGGTTCTTCTACGCCTCCAGTGCATGCATTTACCCTGAATTCAAGCAGCTTGACACA---AAT------GTG---------AGCCTGAAGGAATCCGATGCCTGGCCTGCTGAGCCTCAAGACGCCTATGGCTTGGAGAAGCTTGCAACTGAGGAGTTGTGCAAGCACTACACCAAGGACTTTGGCATCGAGTGCCGCATTGGCCGTTTCCATAACATCTATGGTCCCTTCGGTACATGGAAAGGTGGTCGTGAGAAGGCACCTGCTGCCTTCTGCAGAAAAGCTCAGACATCAACCGAGAGGTTTGAGATGTGGGGCGATGGCCTCCAAACCCGATCCTTCACTTTCATCGACGAATGCGTCGAGGGTGTTCTAAGATTGACCAAGTCCGACTTCCGCGAGCCAGTGAACATTGGGAGTGACGAGATGGTGAGCATGAACGAGATGGCTGAGATCGTGCTGAGCTTTGAGGATAGG---AAGCTGCCCATCCACCACATCCCTGGTCCAGAGGGTGTCCGCGGGCGCAACTCTGACAACACCCTTATCAAGGAGAAGCTCGGGTGGGCTCCGACAATGAAGCTCAAGGACGGGCTTCGGTTCACCTACTTCTGGATCAAGGAGCAGATCGAGAAGGAGAAGACCCAGGGCATC---GACATCGCG---GCGTACGGGTCCTCTAAGGTGGTGTCCACCCAGGCGCCCGTGCAGCTGGGCTCCCTCCGCGCCGCCGACGGCAAGGAGGGCCTC---------------------------------------------------------------------

>PvGME-3

ATGGCG------------------------------CTCAAC------------------AAGGAGTACACGTAC---GCGGAGCTGGAGAAGGAGCCCTACTGGCCATTTGAGAAGCTGCGAATCTCCATCACGGGCGCCGGTGGGTTCATCGCGTCCCACATCGCGAGGCGCCTCAAGAGCGAGGGCCACTACATCATCGCCTCTGACTGGAAGAAGAATGAGCACATGACCGAGGACATGTTCTGCCATGAGTTCCATCTTGTCGACCTGAGGGTCATGGACAACTGCCTCAAGGTGACCACGGGGGTTGACCATGTGTTCAACCTTGCTGCTGATATGGGTGGGATGGGGTTCATCCAGTCCAACCACTCTGTGATCATGTACAACAACACCATGATCAGCTTTAACATGCTTGAGGCTGCTAGAATTAACGGCGTCAAGAGGTTCTTTTATGCATCAAGCGCCTGTATTTATCCTGAATTTAAGCAGTTGGAGACT---GTA------GTT---------AGCTTGAAGGAGTCAGATGCCTGGCCTGCAGAGCCTCAAGATGCCTATGGCTTGGAGAAACTTGCTACTGAGGAACTGTGCAAGCACTACACAAAGGATTTTGGCATTGAGTGCCGGATCGGTCGCTTTCACAACATATATGGTCCCTTTGGAACATGGAAAGGTGGAAGGGAGAAGGCACCTGCTGCTTTCTGCAGAAAGGCTTTAACCTCCACAGGGCGCTTTGAGATGTGGGGAGATGGTCTGCAAACCAGATCCTTCACATTTATTGATGAATGTGTTGAGGGTGTCCTTAGGTTAACAAAGTCTGATTTCCGTGAGCCTGTAAACATTGGAAGTGACGAAATGGTCAGTATGAATGAGATGGCTGAGATAGTCCTCAGCTTTGAGAACAAG---CAGCTGCCCATCCACCACATTCCTGGGCCGGAGGGTGTGCGTGGGCGCAACTCAGACAACACACTCATCAAGGAGAAGCTCGGCTGGGCTCCGACCATGAAGCTGAAGGATGGACTGAGGATAACATACTTCTGGATCAAGGAGCAGCTTGAGAAGGAGAAGGCTGAGGGCATG---GACCTATCG---GTCTACGGATCATCCAAGGTCGTGCAGACTCAGGCCCCTGTTCAGCTCGGTTCCCTCCGCGCTGCCGATGGCAAGGAG---------------------------------------------------------------------------

>PvGME-4

ATGGGG---AGCAGCGAGAAGACC---GTCAGC---GCTTAT------------------GGTGAGTACACCTAT---GCTGAGCTGGAGAGGGAGCCCTACTGGCCAAGTGAGAAGCTGAGGATTTCGATTACTGGGGCTGGTGGTTTCATTGGATCCCACATTGCTCGCCGTCTCAAGAGTGAGGGCCATTACATCATCGCCTCTGACTGGAAGAAGAATGAGCACATGACAGAGGACATGTTCTGCCATGAGTTCCACCTTGTTGACCTCAGGGTCATGGACAACTGTCTCAAGGTTACCCAAGGCGTCGACCATGTATTCAATCTTGCTGCTGATATGGGTGGCATGGGGTTCATCCAGTCAAACCACTCTGTCATCATGTACAACAACACCATGATCAGTTTCAACATGCTTGAGGCTGCACGCATCAATGGTGTGAAGAGGTTCTTCTATGCCTCGAGTGCATGCATTTACCCTGAATTCAAGCAGCTTGAAACA---AAT------GTG---------AGCTTGAAGGAATCTGATGCCTGGCCTGCTGAGCCTCAAGATGCCTATGGCTTGGAGAAGCTTGCAACCGAGGAGCTCTGCAAGCACTACACAAAGGACTTTGGCATCGAGTGCCGCATTGGGCGTTTCCACAACATCTATGGCCCCTTTGGAACATGGAAAGGTGGTCGTGAGAAGGCACCTGCTGCCTTCTGCAGAAAGGCTCAGACATCTACCGAGAGGTTTGAGATGTGGGGCGACGGACTCCAGACACGATCCTTCACTTTCATCGACGAGTGTGTCGAGGGTGTTCTGAGGTTGACCAAGTCTGACTTCTGCGAGCCGGTGAACATCGGGAGCGACGAGATGGTGAGCATGAACGAGATGGCCGAGATCGTCCTGAGCTTCGAGGATAGG---AAGCTGCCCATCCACCACATCCCTGGCCCCGAGGGGGTCCGCGGGCGCAACTCCGACAACACCCTCATCAAGGAGAAGCTTGGCTGGGCCCCGACCATGAAGCTCAAGGACGGGCTGAGGTTCACCTACTTCTGGATCAAGGAGCAGATCGAGAAGGAGAAGACGCAGGGGGTC---GACGTCGCG---GCGTACGGGTCCTCCAAGGTGGTGTCCACCCAGGCGCCCGTGCAGCTGGGCTCCCTCCGCGCCGCCGACGGCAAGGAGGGCCTC---------------------------------------------------------------------

>PvGME-5

ATGGGG---AGCAGCGAGAAGACC---GTCAGC---GCTTAT------------------GGTGAGTACACCTAT---GCTGAGCTGGAGAGGGAGCCCTACTGGCCGAGTGAGAAGCTGAGGATTTCGATTACTGGGGCTGGTGGTTTCATTGGATCCCACATTGCTCGCCGTCTCAAGAGCGAGGGCCATTACATCATCGCCTCTGACTGGAAGAAGAATGAGCACATGACAGAGGACATG---------------------------------GTCATGGACAACTGTCTCAAGGTTACCCAAGGCGTCGACCATGTATTCAATCTTGCTGCTGATATGGGTGGCATGGGGTTCATCCAGTCAAACCACTCTGTCATCATGTACAACAACACCATGATCAGTTTCAACATGCTTGAGGCTGCACGCATCAATGGTGTGAAGAGGTTCTTCTATGCCTCGAGTGCATGCATTTACCCTGAATTCAAGCAGCTTGACACA---AAT------GTG---------AGCTTGAAGGAATCTGATGCCTGGCCTGCTGAGCCTCAAGATGCCTATGGCTTGGAGAAGCTTGCAACTGAGGAGCTGTGCAAGCACTACACCAAGGACTTTGGCATCGAGTGCCGCATTGGGCGTTTCCACAACATCTATGGCCCATTTGGAACATGGAAAGGTGGTCGTGAGAAGGCACCGGCTGCCTTCTGCAGAAAGGCTCAGACATCTACCGAGAGGTTTGAGATGTGGGGTGATGGCCTCCAGACACGATCCTTCACTTTCATCGACGAGTGCGTCGAGGGTGTTCTGAGGCTGACCAAGTCAGACTTCCGCGAGCCGGTGAACATTGGGAGCGACGAGATGGTGAGCATGAACGAGATGGCCGAGATCGTCCTGAGCTTCGAGGATAGG---AAGCTGCCCATCCACCACATCCCTGGCCCGGAGGGGGTCCGCGGGCGCAACTCCAACAACACCCTCATCAAGGAGAAGCTTGGCTGGGCCCCGACGATGAAGCTCAAGGACGGGCTGAGGTTCACCTACTTCTGGATCAAGGAGCAGGTCGAGAAGGAGAAGAAGCAGGGGTTC---GACATCGCG---GCGTACGGATCCTCCAAGGTGGTGTCCACCCAGGCGCCCGTGCAGCTGGGCTCCCTCCGCGCCGCCGACGGCAAGGAGGGTCTC---------------------------------------------------------------------

>RcGME

ATGGGA---AGCAGT------GAA---GGAACC---AACTAT------------------GGGGCTTACACCTAT---GAGAACCTTGAGAGGGAACCTTACTGGCCATCTGAGAAGCTTCGAATTTCAATTACTGGTGCTGGTGGTTTTATTGCCTCCCACATTGCTCGCCGTTTGAAGAGTGAGGGCCATTACATTATTGCTTCCGACTGGAAGAAGAATGAGCACATGACAGAAGATATGTTCTGTCATGAATTCCATCTTGTGGATCTGAGGGTCATGGATAACTGCTTGAAGGTTACTAAGGGTGTAGACCATGTATTTAACCTTGCTGCTGATATGGGTGGGATGGGTTTCATCCAGTCCAACCACTCTGTCATTATGTACAACAACACAATGATCAGCTTCAACATGCTTGAGGCCTCTAGGATCAATGGAGTTAAGAGGCTCTTCTATGCCTCTAGTGCTTGTATTTACCCTGAATTTAAGCAACTGGACACT---AAT------GTT---------AGCTTGAAGGAGTCTGATGCTTGGCCTGCAGAGCCTCAAGATGCATACGGCTTGGAGAAACTTGCTACAGAGGAGTTGTGCAAGCACTACACCAAGGACTTTGGAATTGAATGCCGCATTGGAAGATTCCATAACATTTACGGTCCTTTTGGAACATGGAAAGGTGGCAGGGAGAAGGCACCTGCTGCTTTTTGCAGAAAGGCTCTGACTTCCACCGACAAGTTTGAGATGTGGGGAGATGGACTTCAGACCCGATCATTCACCTTTATTGATGAATGCGTGGAGGGTGTGCTTAGATTGACCAAGTCAGATTTCCGGGAGCCAGTGAACATTGGAAGTGATGAAATGGTTAGCATGAATGAGATGGCTGAGATTGTTCTTAGCTTTGAGGACAGG---AAGCTCCCCATCCATCACATTCCTGGCCCAGAAGGTGTCCGTGGGCGTAACTCTGACAACACCCTGATCAAAGAAAAGCTCGGTTGGGCTCCTACGATGAGGCTAAAGGATGGCCTGAGAATTACATACTTCTGGATCAAGGAACAGATAGAGAAAGAGAAGTCTCAAGGAGTT---GACTTGTCC---ATTTATGGATCATCTAAAGTGGTGGGAACTCAAGCACCAGTTCAATTGGGTTCACTTCGTGCTGCTGATGGCAAAGAG---------------------------------------------------------------------------

>SbGME-1

ATGGCA------------------------------CTCAAC------------------AAGGAGTACACCTAC---GCGGAGCTGGAGAAGGAGCCATACTGGCCGTTCGAGAAGCTGCGGATCTCCATCACTGGTGCTGGTGGGTTCATTGCCTCCCACATCGCGCGCCGCCTCAAGAGCGAGGGCCACTACATCATCGCCTCTGACTGGAAGAAGAACGAGCACATGTCCGAGGACATGTTCTGCCACGAGTTCCACCTCGTCGACCTAAGGGTGATGGACAACTGCCTCAAGGTCACCACGGGGGTTGACCATGTGTTCAACCTCGCGGCTGATATGGGTGGGATGGGGTTCATCCAGTCCAACCACTCTGTGATCATGTACAACAACACCATGATCAGCTTTAACATGCTTGAGGCTGCTAGAATTAACGGTGTCAAGAGGTTCTTTTATGCATCAAGTGCCTGCATCTACCCTGAATTTAAGCAGTTGGAAACT---GTA------GTT---------AGCTTAAAGGAGTCAGATGCTTGGCCTGCTGAGCCTCAAGATGCTTATGGCTTGGAAAAACTTGCTACTGAGGAACTGTGCAAGCACTATACAAAGGATTTTGGCATTGAGTGCCGGGTCGGTCGCTTTCACAACATATATGGTCCCTTTGGAACATGGAAGGGTGGAAGGGAGAAGGCGCCTGCTGCTTTCTGCAGAAAGGCTTTAACCTCCACTGGGCACTTTGAGATGTGGGGCGATGGCCTGCAAACCAGATCCTTCACATTTATTGATGAATGTGTTGAAGGTGTCCTTAGGTTAACAAAGTCTGACTTCCGTGAGCCTGTAAACATTGGAAGTGACGAAATGGTGAGCATGAACGAGATGGCAGAAATAGTCCTGAGCTTTGAGAACAAG---CAGCTGCCCATCCACCACATTCCTGGGCCGGAGGGTGTGCGTGGGCGCAACTCGGACAACACACTCATTAAGGAGAAGCTTGGCTGGGCTCCGACTATGAGGCTGAAGGATGGGCTGAGGATCACCTACTTCTGGATCAAGGAGCAGCTTGAGAAGGAGAAGGCTGAGGGCATG---GATCTGTCG---GTATACGGATCATCCAAGGTCGTGCAGACCCAGGCCCCGGTTCAGCTTGGCTCCCTTCGTGCCGCTGATGGCAAGGAG---------------------------------------------------------------------------

>SbGME-2

ATGGGG---AGCGGCGAGAAGACC---GTCACC---GCTTAT------------------GGCGAGTACACCTAT---GCTGAGCTGGAGAGGGAGCCCTACTGGCCAAGTGAGAAGCTGAGGATTTCGATTACTGGTGCTGGTGGTTTCATTGGATCCCACATTGCTCGCCGTCTGAAGAGCGAGGGCCATTACATCATTGCCTCTGACTGGAAGAAGAATGAGCACATGACCGAGGACATGTTCTGCCATGAGTTCCACCTTGTTGACCTCAGGGTCATGGACAACTGTCTGAAGGTCACCCAAGGTGTCGACCATGTCTTTAATCTTGCTGCTGATATGGGTGGCATGGGGTTCATCCAGTCCAATCACTCTGTGATCATGTACAACAACACCATGATCAGTTTCAACATGCTGGAGGCTGCACGTATCAATGGTGTGAAGAGGTTCTTCTACGCTTCAAGTGCATGCATTTACCCTGAATTCAAGCAGCTTGACACA---AAT------GTG---------AGCCTGAAGGAATCTGATGCCTGGCCTGCTGAGCCTCAAGACGCCTATGGCTTGGAGAAGCTTGCAACTGAGGAGCTGTGCAAGCACTACACCAAGGACTTTGGCATCGAGTGCCGCGTTGGCCGCTTCCACAACATTTACGGCCCTTTTGGTACATGGAAAGGTGGTCGTGAGAAGGCACCGGCCGCCTTCTGCAGAAAGGCTCAGACATCCACGGAGAGGTTTGAGATGTGGGGTGATGGTCTCCAGACCCGATCCTTCACTTTCATCGACGAGTGTGTTGAGGGTGTTCTGAGATTGACCAAGTCAGACTTCCGCGAGCCAGTGAACATCGGAAGCGATGAGATGGTGAGCATGAACGAGATGGCTGAGATCGTGCTGAGCTTTGAGGATAGG---AAGCTGCCCATCCACCACATCCCTGGTCCAGAGGGGGTCCGTGGGCGCAACTCTGACAACACCCTTATCAAGGAGAAGCTTGGCTGGGCCCCAACAATGAAGCTCAAGGATGGGCTGAGGTTCACCTACTTCTGGATCAAGGAGCAGATCGAGAAGGAGAAGACCCAGGGGGTT---GACATCGCG---GCGTATGGGTCGTCCAAGGTGGTGTCCACCCAGGCGCCCGTGCAGCTGGGCTCCCTCCGTGCCGCTGATGGCAAGGAGGGCCTC---------------------------------------------------------------------

>SfGME-1

ATGGCG---AGCAAT------GGAGTGAAGGAAGTAGTATAC------------------GGGGAGTACACTGCC---AGTAATCTGGAGCGGGAGCTATACTGGCCGCAGGAGAAGCTGCGGATTTCCATCACAGGAGCTGGCGGATTCATCGCCTCGCACATTGCCAGACGCTTGAAGACCGAGGGTCACTACATCGTGGCATCGGATTGGAAGAAGAATGAGCACATGAGTGAGGACATGTTCTGTGACGAGTTCCATTTGGTGGATCTCCGCGTCATGGACAACTGCTTGGCCGTCACCAAAGGCGTTCAGCATGTGTTCAACCTGGCGGCGGACATGGGCGGCATGGGCTTTATCCAGTCCAACCATTCCGTCATCATGTATAACAACACTATGATCAGCTTCAACATGCTGGAAGCAGCTCGCATCAACGGCGTCCACAGATTCTTTTATGCATCCAGTGCTTGCATTTACCCTGAATTCAAGCAACTGGAGACT---AAT------GTG---------AGCCTGAAGGAATCAGATGCCTGGCCTGCTGAGCCACAAGATGCTTATGGCCTGGAGAAACTGGCAACTGAGGAGCTTTGTAAACATTACACAAAGGATTTCAAAATGGAGTGTCGCATCGGAAGATTCCACAACATCTATGGCCCCTTCGGTACCTGGAAGGGCGGACGGGAGAAAGCTCCTGCTGCCTTTTGCAGGAAGGCTCTGACAGCAGTGGACAAGTTTGAGATGTGGGGTGATGGTCTGCAAACACGTTCCTTCACTTTTATTGATGAATGTGTGGAAGGTGTTTTGAGATTGACCAAATCAGACTTCCAGGAGCCACTGAATATTGGCAGTGATGAGATGGTAAGCATGAATGAGATGGCAGAGATAGTCCTGAGCTTTGAGAACAAA---AAACTGCCTATTGAACACATTCCTGGTCCTGAGGGTGTGCGTGGCCGCAACTCTGATAATACACTTATCAAAGAGAAGCTTGGATGGGCTCCAACTATGCGCCTGAAGGATGGACTACGCATCACATATTTGTGGATCAAGGAGCAGATTGAGAAAGAGAAGACTCAAGGTGTG---GACTTGGCTGGCAAGTATGCTTCATCAAAGGTGGTGGGCACTCAAGCTCCTGTTCAGCTTGGGTCCCTCAGAGCAGCTGATGGGAAAGAG---------------------------------------------------------------------------

>SfGME-2

ATGGTGATTTCTAAC------GGACTGAAGGAG------TAT------------------GGGGAGTATACGGCG---AGTAACTTGGATCGGGAACCTTACTGGCCGGAACAGAAGCTGCGGATTTCCATCACGGGAGCCGGAGGATTCATTGCGTCGCATATTGCCAGGCGTCTCAAGAGTGAGGGCCACTACATCGTGGCTTCTGACTGGAAGAAGAATGAGCACATGAGTGAGGACATGTTCTGTGACGAGTTCCATCTGGTGGATCTGCGCGTCATGGACAACTGCCTGGCCGTCACCAAGGGCGTACAGCATGTTTTCAACCTGGCGGCCGACATGGGCGGTATGGGCTTTATTCAGTCTAACCACTCTGTCATCATGTACAACAACACTATGATCAGCTTCAACATGCTGGAAGCAGCCCGCATCAATGGCATCCACAGATTTTTCTATGCATCCAGTGCTTGCATTTACCCTGAGTTCAAGCAGCTGGAAACT---AAT------GTG---------AGCCTGAAGGAATCAGATGCTTGGCCTGCTGAGCCACAAGATGCTTATGGCTTGGAGAAGCTGGCAACTGAGGAGCTTTGCAAACATTACACCAAGGATTTCAAAATGGAGTGTCGTATTGGAAGGTTCCACAACATCTATGGACCCTTTGGTACCTGGAAGGGGGGACGGGAGAAAGCTCCTGCTGCTTTTTGTAGGAAGGCTCTGACAGCAGTGGACAAATTTGAGATGTGGGGTGATGGCCTCCAAACACGCTCCTTCACATTCATTGATGAATGTGTAGAAGGTGTTTTGAGGTTGACCAAATCGGATTTCCAGGAGCCTTTGAATATTGGTAGTGATGAGATGGTAAGCATGAACGAGATGGCAGAGATAGTGTTGAGTTTTGAGAACAAA---AAACTGCCTATTGAGCATATACCCGGTCCTGAGGGTGTACGTGGCCGCAACTCTGACAATACTCTCATCAAAGAGAAGCTTGGATGGGCACCATCCATGCGCCTGAAGGATGGGCTGCGAATCACATATTTGTGGATCAAGGAGCAGATTGAGAAAGAGAAGACACAAGGTGTA---GACTTGGCTGGAAAGTACTCGTCATCAAAGGTGGTAGGCACTCAAGCTCCTGTCCAGCTTGGGTCCCTTAGAGCAGCTGATGGCAAAGAG---------------------------------------------------------------------------

>SiGME-1

ATGGGG---AGCAGCGAGAAGACC---GTTACC---GCTTAT------------------GGTGAGTACACCTAT---GCTGAGCTGGAGAGGGAGCCCTACTGGCCAAGTGAGAAGCTGAGGATTTCAATTACTGGGGCTGGTGGTTTCATTGGATCACACATTGCTCGCCGCCTGAAGAGTGAGGGCCATTACATCATCGCCTCTGACTGGAAGAAAAATGAGCATATGACTGAGGACATGTTCTGCCATGAGTTCCACCTTGTTGACCTCAGGGTCATGGACAACTGTCTGAAGGTTACCCAAGGCGTCGACCATGTATTCAATCTTGCTGCTGATATGGGTGGCATGGGGTTCATCCAGTCAAACCACTCTGTCATCATGTACAACAACACCATGATCAGTTTCAACATGCTTGAAGCTGCACGTATCAATGATGTGAAGAGGTTCTTCTACGCCTCGAGTGCATGCATTTACCCTGAATTCAAACAGCTTGACACA---AAT------GTG---------AGCTTGAAGGAATCTGATGCCTGGCCTGCTGAGCCTCAAGACGCCTATGGCTTGGAGAAGCTTGCAACTGAGGAGTTGTGCAAGCACTACACCAAGGACTTTGGCATTGAGTGCCGCGTTGGCCGTTTCCACAACATATATGGTCCCTTTGGAACATGGAAGGGTGGTCGTGAGAAGGCACCTGCTGCCTTCTGCAGAAAGGCTCAGACATCCACCGAGAGGTTTGAGATGTGGGGTGATGGCCTCCAGACCCGATCCTTCACATTCATTGACGAGTGTGTCGAGGGTGTTCTCAGATTGACTAAGTCAGACTTCCGCGAGCCGGTGAACATTGGAAGCGATGAGATGGTGAGCATGAATGAGATGGCTGAGATCGTCCTGAGCTTTGAGGATAGG---AAGCTGCCCATCCACCACATCCCTGGTCCAGAGGGGGTCCGTGGGCGCAACTCCGACAACACTCTCATCAAGGAAAAGCTTGGGTGGGCCCCGACAATGAAGCTCAAGGATGGGCTTCGGTTTACGTACTTCTGGATTAAGGAGCAGATCGAGAAGGAGAAGACCCAGGGCATC---GACATCGCA---GGATACGGGTCCTCCAAGGTGGTGTCGACCCAGGCGCCTGTGCAGCTGGGCTCCCTCCGTGCTGCCGACGGCAAGGAGGGCCTG---------------------------------------------------------------------

>SiGME-2

ATGGGG---AGCAGCGAGAAGACC---GTCAGC---ACTTAT------------------GGTGAGTACACCTAT---GCTGAGCTGGAGAGGGAGCCCTACTGGCCCAGTGAGAAGTTGAGGATTTCCATTACTGGGGCTGGTGGTTTCATTGGATCCCACATTGCTCGCCGTCTGAAAAGCGAGGGCCATTACATCATCGCCTCTGACTGGAAGAAGAATGAGCACATGACTGAGGATATGTTCTGCCATGAGTTCCACCTTGTTGACCTCAGGGTCATGGACAACTGTCTGAAGGTTACCCAAGGCGTCGACCATGTATTCAATCTTGCTGCTGATATGGGTGGCATGGGGTTCATCCAGTCAAACCACTCTGTCATCATGTACAACAACACCATGATCAGTTTCAACATGCTTGAGGCTGCACGCATCAATGGTGTGAAGAGGTTCTTCTATGCCTCGAGTGCGTGCATTTACCCAGAATTCAAGCAGCTTGAGACA---AAT------GTG---------AGCCTGAAGGAATCTGATGCCTGGCCTGCTGAGCCTCAAGATGCCTATGGCTTGGAGAAGCTTGCAACTGAGGAGCTCTGCAAGCACTACACCAAGGACTTTGGCATCGAGTGCCGTGTTGGCCGTTTCCACAACATCTATGGCCCCTTCGGAACATGGAAAGGTGGCCGTGAGAAGGCACCGGCTGCCTTCTGCAGAAAGGCTCAGACATCTACCGAGAGGTTTGAGATGTGGGGCGATGGCCTCCAGACACGGTCCTTCACCTTCATTGACGAGTGCGTTGAGGGTGTCCTGAGGCTGACCAAGTCAGACTTCCGTGAGCCGGTGAACATCGGGAGCGATGAGATGGTGAGCATGAACGAGATGGCCGAGATTGTCCTGAGCTTTGAGGATAGG---AAGCTGCCCATCCACCACATCCCTGGTCCCGAGGGGGTCCGTGGGCGCAACTCTGACAACACCCTTATCAAGGAGAAGCTAGGCTGGGCCCCGACAATGAAGCTCAAGGATGGGCTGAGGTTCACCTACTTCTGGATCAAGGAGCAGATCGAGAAGGAGAAGACGCAGGGTGTT---GACATCGCG---GCGTACGGGTCCTCCAAGGTGGTGTCGACGCAGGCGCCCGTGCAGCTGGGCTCCCTCCGCGCCGCCGACGGCAAGGAGGGCCTC---------------------------------------------------------------------

>SiGME-3

ATGGCG------------------------------CTCAAC------------------AAGGAGTACACGTAC---GCGGAGCTGGAGAAGGAGCCATACTGGCCGTTTGAGAAGCTGCGGATCTCCATCACGGGCGCTGGCGGGTTCATCGCCTCCCACATCGCTAGGCGCCTCATGAGCGAGGGCCACTACATCATCGCCTCTGACTGGAAGAAGAACGAGCACATGACCGAGGAAATGTTCTGCCATGAGTTCCACCTCATCGACCTGAGGGTCATGGACAACTGCCTCAAGGTGACCACGGGGGTTGACCATGTGTTTAACCTTGCTGCTGATATGGGAGGGATGGGGTTCATCCAGTCCAACCACTCTGTCATCATGTATAACAACACTATGATCAGCTTTAACATGCTCGAGGCTGCTAGAATCAATGGCGTCAAGAGGTTCTTTTATGCATCAAGCGCCTGTATCTACCCTGAATTTAAGCAGTTGGAGACT---GTA------GTT---------AGCTTGAAGGAGTCAGATGCCTGGCCTGCAGAGCCTCAAGATGCCTATGGCTTGGAGAAACTTGCTACTGAGGAACTGTGCAAGCACTACACAAAGGATTTTGGCATTGAGTGCCGGATCGGTCGCTTTCACAACATATATGGTCCCTTTGGGACATGGAAAGGTGGAAGGGAGAAGGCACCTGCTGCTTTCTGCAGAAAGGCTTTAACCTCCACTGGGCGCTTTGAGATGTGGGGCGATGGTCTGCAAACCAGATCCTTCACATTTATTGATGAATGTGTCGAGGGTGTCCTTAGGTTAACAAAGTCTGATTTCCGTGAGCCTGTAAACATTGGAAGTGATGAAATGGTCAGCATGAATGAGATGGCTGAGATAGTCCTCAGCTTTGAGAACAAG---CAGCTGCCCATCCACCACATTCCTGGACCGGAGGGTGTGCGAGGGCGGAATTCAGATAACACACTCATCAAGGAGAAGCTTGGCTGGGCTCCGACCATGAAGCTGAAGGATGGACTAAGGATCACATACTTCTGGATCAAGGAGCAGCTTGAGAAGGAGAAGGCTGAGGGCATG---GATCTGTCG---GTCTATGGTTCATCCAAGGTCGTGCAGACACAGGCCCCTGTTCAGCTCGGTTCCCTCCGCGCTGCCGATGGCAAGGAG---------------------------------------------------------------------------

>SmGME-1

ATGGAA------------------------------TCCTTT------------------GGAGCTTACACAGTTGGGGCAGAGCTGGAGCGTGAGGCCTACTGGCCAGAAGCCAAGCTCCGGATTTGCATCACCGGTGCTGGAGGATTCATAGCATCGCACATTGCACGGCGGCTCAAAGCCGAAGGCCACTATATAATTGCGTCCGACTGGAAGAAAAACGAACACATGAGCGAGGATATGTTCTGCCATGAGTTCCATCTCGTGGATCTGCGCGTCATGGACAATTGCATGGTGGTGACAAAGGGCGCGGATCACGTCTTCAATCTCGCGGCCGACATGGGCGGGATGGGTTTCATCCAGTCGAATCACTCAGTCATCCTTTTCAACAATACCATGATCAGCTTCAACATGCTCGAGGCCTCCCGAATCAATGGAGTCAAAAGATTCTTTTACGCGTCCAGCGCATGCATCTATCCGGAGTTCAAACAGCTAGAAACG---AAC------GTG---------AGTCTCAAAGAAGGCGATGCATGGCCGGCAGAGCCCCAAGATGCCTATGGGCTGGAGAAGCTGTGCACCGAAGAGCTGTGCAAGCACTACACCAAGGATTTTGGAATCGAGTGCCGGATTGGGCGCTTCCACAACATCTACGGGCCTTTTGGTACCTGGAAAGGCGGACGAGAGAAGGCTCCAGCCGCGTTTTGTCGCAAGGCCCTGACTTCCACCGACAAATTCGAGATGTGGGGCGATGGTATGCAGACGCGATCCTTCACGTTCATCGACGAGTGCGTGGAGGGCGTCTTGCGACTCACCAAGTCCGACTTCCGGGAGCCGCTCAACATCGGCAGCGACGAGATGGTGAGCATGAACGAGATGGCGGAGATGATCCTGGGCTTTGAAAACAAG---CAAATTCCAATCCAGCACATCCCCGGGCCGGAGGGAGTCCGCGGAAGGAACTCGGACAACAGTCTGATCAAGGAGAAGCTGGGCTGGGCTCCCTCGATGCGACTGAGAGATGGGCTGCGAATCACCTACATGTGGATCAAGGAGCAACTTGAAAGGGAGATGGCGGAGGGATCACACGACTTGGCGGCAGCTTACAGCTCCTCCAAGGTTGTGGGAACTCAAGCTCCAGTCCAGCTCGGCTCACTCAGGAAGGCCGATGGAAAGGAA---------------------------------------------------------------------------

>SmGME-2

ATGGAG------------------------------TGCTTT------------------GGAGCTTACACCGTAGGAGCCGAGCTAGAGCGCGAGGCGTATTGGCCCCAAGCCAAGCTCCGGATTTCCATCTCCGGCGCTGGAGGATTCATCGCCTCGCACATAGCGCGGCGGCTCAAGGCGGAGGGACACTACGTGATCGCGTCCGACTGGAAGAAGAACGAGCACATGAGCGAGGACATGTTTTGCCACGAATTCCATCTCGTGGATCTGCGCGTCATGGAGAATTGCTTGGCGGTGACAAGGGGCGTGGATCACGTCTTCAACCTCGCGGCGGACATGGGCGGAATGGGATTCATCCAGTCCAATCACTCTGTGATTCTCTTCAACAACACCATGATCAGTTTCAACATGCTCGAGGCCTCCCGGATCAATGGAGTCAAAAGATTTTTTTATGCGTCCAGTGCATGCATTTACCCCGAGTTTAAGCAGCTGGAGACT---AAC------GTG---------AGTTTGAAGGAGTCAGATGCATGGCCGGCAGAGCCCCAAGATGCCTATGGACTGGAGAAGCTATGCACTGAAGAGCTGTGCAAGCACTACACCAAGGATTTTGGGATCGAGTGCCGCATTGGACGATTCCACAACATCTATGGACCCTTTGGAACCTGGAAAGGTGGACGAGAGAAGGCTCCGGCTGCGTTTTGTCGAAAGGCTCTGACTTCCACCGATTCGTTTGAGATGTGGGGCGATGGAATGCAGACGCGATCCTTCACCTTCATCGACGAGTGCGTGGAGGGCGTCTTGCGACTCACAAAGTCCGATTTCCGGGAGCCGCTCAACATTGGAAGCGACGAGATGGTGAGCATGAACGAGATGGCAGAGATCATACTCGGATTTGAGAAGAAG---CAAATCCCGATCCGCCATATACCTGGGCCGGAGGGAGTTCGCGGGAGAAACTCGGATAACAGTTTGATCAAGGAGAAGCTCGGATGGGCTCCCTCGATGCGATTGAAAGATGGGCTGCGGATCACTTACTTGTGGATTAAGGAGCAGCTTGACAAGGAAATTGGCGAGGGATCTCAAGACTTACTTGCGGGTTACAGCTCCTCCAAGGTTGTGGGAACTCAAGCTCCAGTCCAGCTTGGTTCTCTCAGGAAGGCCGATGGAAAGGAG---------------------------------------------------------------------------

>SmGME-3

ATGGAA------------------------------TCCTTT------------------GGAGCTTATACAGTTGGGGCAGAGCTGGAGCGCAAAGCCTATTGGCCGGAATCTAAACTCCGGATTTGCATCACCGGTGCTGGAGGATTCATAGCATCGCACATTGCACGGCGGCTCAAGGCGGAGGGACACTACATAATCGCATCGGACTGGAAGAAGAACGAACACATGAGCGAGGATATGTTCTGCCATGAGTTCCATCTCGTGGATCTCCGCGTCATGGACAACTGCATGGTTGTGACGAGGGGCGCGGATCACGTCTTCAATCTCGCGGCCGACATGGGCGGGATGGGTTTCATCCAGTCGAATCACTCGGTCATCCTTTTCAACAATACCATGATCAGCTTCAACATGCTCGAGGCCTCCCGAATCAATGGAGTCAAAAGATTCTTTTACGCGTCGAGCGCATGCATTTATCCAGAGTTCAAACAGCTAGAAACG---AAC------GTG---------AGCCTCAAAGAAGGCGATGCATGGCCGGCAGAGCCCCAAGATGCCTATGGGCTGGAGAAGCTGTGCACCGAAGAGCTGTGCAAGCACTACACCAAGGACTTTGGAATCGAGTGCCGGATTGGGCGCTTCCACAACATCTACGGGCCTTTTGGTACCTGGAAAGGCGGACGAGAGAAGGCTCCAGCCGCGTTTTGTCGCAAGGCCTTGACTTCCACCGACAAATTCGAGATGTGGGGCGATGGTATGCAGACGCGATCCTTCACGTTCATCGACGAGTGCGTGGAGGGCGTCTTGCGACTCACCAAGTCCGACTTCCGGGAGCCGCTCAACATCGGCAGCGACGAGATGGTGAGCATGAACGAGATGGCGGAGATGATCCTGGGCTTTGAAAACAAG---CAAATTCCAATCCAGCACATCCCCGGGCCGGAGGGAGTCCGCGGAAGGAACTCGGACAACAGTCTGATCAAGGAGAAGCTGGGCTGGGCTCCCTCGATGCGACTGAGAGATGGGCTGCGAATCACCTACATGTGGATCAAGGAGCAGCTTGAGAAGGAGATGGCGGAGGGATCACACGACTTGGCGGCAGCTTATAGCTCCTCCAAGGTTGTGGGAACTCAAGCTCCAGTCCAACTCGGCTCACTCAGGAAGGCCGATGGAAAGGAG---------------------------------------------------------------------------

>SmGME-4

ATGGGG---------------AGC------------CACTAT------------------GGCGATTACACCGCAGGGGCAGAGCTGGAGCGCGAGACCTATTGGCCGAATGAAAAGCTTCGCATTTCCATCTCCGGCGCTGGAGGATTCATAGCATCACACATCGCGCGGCGGCTCAAGGCCGAGGGCCACTACGTCATCGCGTCGGATTGGAAGAAGAACGAGCATATGAGCGAGGAGATGTTTTGCCACGAATTCCATCTCGTGGATCTGCGAGTGATGGAGAATTGCTTGGCGGTGACCAAGGGTGTTGATCACGTCTTTAATTTGGCAGCCGACATGGGTGGGATGGGATTCATACAATCGAATCACTCGGTGATTCTTTTCAACAACACCATGATCAGTTTCAACATGCTGGAAGCAGCTCGGATCAATGGAGTCAAAAGGTTTTTCTATGCATCCAGCGCATGCATTTACCCAGAATTCAAGCAACTTGAAACG---AAC------GTG---------AGTCTCAAGGAATCGGATGCTTGGCCAGCAGAGCCTCAAGATGCTTATGGACTAGAGAAGCTCTGCAGTGAAGAGCTGTGCAAGCACTACACCAAGGATTTTGGAATCGAGTGCCGGATTGGAAGATTTCACAATATTTATGGGCCTTTTGGTACCTGGAAAGGTGGACGGGAGAAGGCTCCAGCGGCATTTTGTAGGAAAGCTCTAACTTCCACTGATAAGTTTGAGATGTGGGGTGATGGGAAGCAAACACGATCATTTACGTTCATCGATGAATGTGTGGAGGGTGTTTTGAGGCTGACAAAGTCGGACTTTCGTGAACCGCTCAATATCGGAAGTGATGAGATGGTGAGCATGAACGAGATGGCTGAGATTATCTTGGGTTTTGGAGACAAG---AAACTGCCAATCCACCATATCCCTGGGCCAGAAGGTGTTCGTGGTAGAAATTCGGACAACAATCTGATAAAGGAGAAGCTCGGTTGGGCCCCAACAATGCGACTCAAGGATGGACTCCGGATCACTTATATGTGGATCAAGGAGCAGCTAGACGAGGAGATCTCTGAGGGACAG---GATATGGCT---GCTTATAGCTCCTCCAAAGTTGTGGGAACTCAAGCTCCTGTCCAGCTTGGCTCGCTCAGGAAGGCCGATGGCAAGGAG---------------------------------------------------------------------------

>SmGME-5

ATGGAG------------------------------TCCTTT------------------GGCGCTTACACCGTAGGAGCCGAGCTAGAGCGCGAGGCGTATTGGCCGCAAGCCAAGCTCCGGATTTCCATCTCCGGCGCTGGAGGATTCATCGCCTCACACATAGCGCGGCGGCTCAAGGCGGAGGGACACTACGTGATCGCGTCCGACTGGAAGAAGAACGAGCACATGAGCGAGGACATGTTTTGCCACGAATTCCATCTCGTGGATCTGCGCGTCATGGAGAATTGCCTGGCGGTGACAAGGGGCGTGGATCACGTCTTCGATCTCGCGGCCGACATGGGCGGAATGGGATTCATCCAGTCCAATCACTCTGTGATTCTCTTCAACAACACCATGATCAGTTTCAACATGCTCGAGGCCTCCCGGATCAACGGAGTCAAAAGATTTTTTTATGCGTCCAGTGCATGCATTTACCCCGAGTTTAAGCAGCTGGAGACT---AAC------GTG---------AGTTTGAAGGAGTCAGATGCATGGCCGGCAGAGCCCCAAGATGCCTATGGACTGGAGAAGCTGTGCACTGAAGAGCTGTGCAAGCACTACACCAAGGATTTTGGGATCGAGTGCCGCATTGGACGATTCCACAACATCTATGGACCCTTTGGGACCTGGAAAGGTGGACGAGAGAAGGCTCCGGCTGCGTTTTGTCGAAAGGCCCTGACTTCCACCGATTCGTTTGAGATGTGGGGCGATGGAATGCAGACGCGATCGTTCACCTTCATCGACGAGTGCGTGGAGGGCGTCTTGCGACTCACAAAGTCCGATTTCCGGGAACCGCTCAACATTGGAAGCGACGAGATGGTGAGCATGAACGAGATGGCAGAGATCATACTAGGATTTGAGAACAAG---CAAATCCCGATCCGTCATATACCTGGGCCGGAGGGAGTCCGCGGGAGAAACTCGGAGAACAGTTTGATCAAGGAGAAGCTCGGATGGGCTCCCTCGATGCGATTGAAAGATGGGCTGCGTATCACTTACTTGTGGATTAAGGAGCAGCTTGACAAGGAGATTGGCGAGGGATCTCAAGACTTACTTACGGGTTACAGCTCCTCCAAGGTTGTGGGAACTCAAGCTCCAGTCCAACTTGGGTCTCTCAGGAAGGCCGATGGAAAGGAG---------------------------------------------------------------------------

>SmGME-6

ATGGAA------------------------------TCCTTT------------------GGAGCTTACACAGTTGGGGCAGAGCTGGAGCGTGAGGCCTACTGGCCAGAAGCCAAGCTCCGGATTTGCATCACCGGTGCTGGAGGATTCATAGCATCGCACATCGCACGGCGGCTCAAAGCCGAGGGCCACTATATAATTGCCTCCGATTGGAAGAAGAACGAACACATGAGCGAGGATATGTTCTGCCATGAGTTCCATCTCGTGGATCTGCGCGTCATGGACAACTGCATGGTTGTGACGAGGGGCGCGGATCACGTCTTCAATCTCGCGGCCGACATGGGCGGGATGGGTTTCATCCAGTCGAATCACTCGGTCATCCTTTTCAACAATACCATGATCAGCTTCAACATGCTCGAGGCCTCCCGAATCAATGGAGTCAAAAGATTCTTTTACGCGTCCAGCGCATGCATTTATCCAGAGTTCAAACAGCTAGAAACG---AAC------GTG---------AGTCTCAAGGAAGGCGATGCATGGCCGGCAGAGCCCCAAGATGCCTATGGGCTGGAGAAGCTGTGCACGGAAGAGCTGTGCAAGCACTACACCAAGGATTTTGGAATCGAGTGCCGGATTGGGCGCTTCCACAACATCTACGGGCCTTTTGGTACCTGGAAAGGCGGACGAGAGAAGGCTCCAGCCGCGTTTTGTCGCAAGGCCTTGACTTCCACCGACAAATTCGAGATGTGGGGCGATGGTATGCAGACGCGATCCTTCACGTTCATCGACGAGTGCGTGGAAGGCGTCTTGCGACTCACCAAGTCCGACTTCCGGGAGCCGCTCAACATCGGCAGCGACGAGATGGTGAGCATGAACGAGATGGCGGAGATGATCCTGGGCTTTGAAAACAAG---CAAATTCCAATCCAGCACATCCCCGGGCCGGAGGGAGTCCGCGGAAGGAACTCGGACAACAGTCTGATCAAGGAGAAGCTGGGCTGGGCTCCCTCGATGCGACTGAGAGATGGGCTGCGAATCACCTACATGTGGATCAAGGAGCAACTTGAAAAGGAGATGGCGGAGGGATCACACGACTTGGCGGCAGCTTACAGCTCCTCCAAGGTTGTGGGAACTCAAGCTCCAGTCCAGCTCGGCTCACTCAGGAAGGCCGATGGAAAGGAG---------------------------------------------------------------------------

>SmGME-7

ATGGAA------------------------------TCCTTT------------------GGAGCTTACACAGTTGGGGCGGAGCTGGAGCGTGAGGCCTACTGGCCAGCAGCCAAGCTCCGGATTTGCATCACCGGCGCTGGAGGATTTATTGCATCACACATTGCACGGCGACTCAAGGCGGAGGGACACTACATAATCGCATCGGACTGGAAGAAGAACGAACACATGAGTGAGGACATGTTCTGCCACGAGTTCCATCTCGTGGATCTGCGCGTCATGGACAATTGCATGGTGGTGACAAAGGGCGTGGATCATGTCTTCAATCTCGCGGCTGATATGGGCGGGATGGGTTTCATCCAGTCGAATCACTCGGTCATCCTTTTCAACAATACCATGATCAGCTTCAACATGCTCGAGGCCTCCCGAATCAATGGAGTCAAAAGATTCTTTTACGCGTCCAGCGCATGCATCTATCCGGAGTTCAAACAGCTAGAAACG---AAC------GTG---------AGCCTCAAAGAAGGCGATGCATGGCCGGCAGAGCCCCAAGATGCCTATGGGCTGGAGAAGCTGTGCACCGAAGAGCTGTGCAAGCACTACACCAAGGATTTTGGAATCGAGTGCCGGATTGGGCGCTTCCACAACATCTACGGGCCTTTTGGTACCTGGAAAGGCGGACGAGAGAAGGCTCCAGCCGCGTTTTGTCGCAAGGCCTTGACTTCCACCGACAAATTCGAGATGTGGGGCGATGGTATGCAGACGCGATCCTTCACGTTCATCGACGAGTGCGTGGAGGGAGTCTTGCGACTCACCAAATCCGACTTCCGGGAGCCGCTCAACATCGGCAGCGACGAGATGGTGAGCATGAACGAGATGGCGGAGATGATCCTGGGCTTTGAAAACAAG---CAAATTCCAATCCAGCACATCCCCGGGCCGGAGGGAGTCCGCGGAAGGAACTCGGACAACAGTCTGATCAAGGAGAAGCTGGGCTGGGCTCCCTCGATGCGACTGAGAGATGGGCTGCGAATCACCTACATGTGGATCAAGGAGCAGCTTGAGAAGGAGATGGCGGAGGGATCACACGACTTGGCGGCAGCTTACAGCTCCTCCAAGGTTGTGGGAACTCAAGCTCCAGTCCAGCTCGGCTCACTCAGGAAGGCCGACGGAAAGGAG---------------------------------------------------------------------------

>SmGME-8

ATGGAG------------------------------TGCTTT------------------GGAGCTTACACCGTAGGAGCCGAGCTAGAGCGCGAGGCGTATTGGCCCCAAGCCAAGCTCCGGATTTCCATCTCCGGCGCTGGAGGATTCATCGCCTCGCACATAGCGCGGCGGCTCAAGGCGGAGGGACACTACGTGATCGCGTCCGACTGGAAGAAGAACGAGCACATGAGCGAGGACATGTTTTGCCACGAATTCCATCTCGTGGATCTGCGCGTCATGGAGAATTGCTTGGCGGTGACAAGGGGCGTGGATCACGTCTTCAATCTCGCGGCGGACATGGGCGGAATGGGATTCATCCAGTCCAATCACTCTGTGATTCTCTTCAACAACACCATGATCAGTTTCAACATGCTCGAGGCGTCCCGGATCAATGGAGTCAAGAGATTTTTTTATGCGTCCAGTGCATGCATTTACCCCGAGTTTAAGCAGCTGGAGACT---AAC------GTG---------AGTTTGAAGGAGTCAGATGCATGGCCAGCAGAACCCCAAGATGCCTATGGACTGGAGAAGCTGTGCACTGAAGAGCTGTGCAAGCACTACACCAAGGATTTTGGGATCGAGTGCCGCATTGGACGATTCCACAACATCTATGGGCCCTTTGGGACCTGGAAAGGTGGACGAGAGAAGGCTCCGGCTGCGTTTTGTCGAAAGGCTCTGACCTCCACCGATTCGTTTGAGATGTGGGGCGATGGAATGCAGACGCGATCCTTCACCTTCATCGACGAGTGCGTGGAGGGCGTCTTGCGACTCACAAAGTCCGATTTCCGGGAGCCGCTCAACATTGGAAGCGACGAGATGGTGAGCATGAACGAGATGGCGGAGATCATACTCGGATTTGAGAAGAAG---CAAATCCCGATCCGCCATATACCTGGGCCGGAGGGAGTTCGCGGGAGAAACTCGGATAACAGTTTGATCAAGGAGAAACTCGGATGGGCTCCCTCGATGCGATTGAAAGATGGGCTGCGGATCACTTACTCGTGGATTAAGGAGCAGCTTGACAAGGAAATTGGCGAGGGATCTCAAGACTTACTTGCGGGTTACAGCTCCTCCAAGGTTGTGGGAACTCAAGCTCCAGTCCAGCTTGGTTCTCTCAGGAAGGCCGACGGAAAGGAG---------------------------------------------------------------------------

>SmGME-9

ATGGGG---------------AGC------------CACTAT------------------GGCGATTACACCGCAGGGGCAGAGCTGGAGCGCGAGGCCTATTGGCCGAATGAAAAGCTTCGCATTTCCATCTCCGGCGCTGGAGGATTCATCGCATCACACATTGCGCGGCGGCTCAAGGCCGAGGGCCACTACGTCATCGCGTCGGATTGGAAGAAGAACGAGCATATGAGCGAGGAGATGTTTTGCCACGAATTCCATCTCGTGGATCTGCGAGTGATGGAGAATTGCTTGGCGGTGACCAAGGGTGTTGATCACGTCTTTAATTTGGCAGCCGACATGGGTGGGATGGGATTCATACAATCGAATCACTCGGTGATTCTTTTCAACAACACCATGATCAGTTTCAACATGCTGGAAGCAGCTCGGATCAATGGAGTCAAAAGATTTTTCTATGCATCCAGCGCATGCATTTACCCAGAATTCAAGCAACTTGAAACG---AAC------GTG---------AGTCTCAAGGAATCGGATGCTTGGCCAGCAGAGCCTCAAGATGCTTATGGACTAGAGAAGCTCTGCAGTGAAGAGCTGTGCAAGCACTACACCAAGGATTTTGGAATCGAGTGCCGGATTGGAAGATTTCACAATATTTATGGGCCTTTTGGTACCTGGAAAGGTGGACGGGAGAAGGCTCCAGCGGCATTTTGTAGGAAAGCTCTAACTTCCACTGATAAGTTTGAGATGTGGGGTGATGGGAAGCAAACACGATCATTTACGTTCATCGATGAATGTGTGGAGGGTGTTTTGAGGCTGACAAAGTCGGACTTTCGTGAACCGCTCAATATCGGAAGTGATGAGATGGTGAGCATGAACGAGATGGCTGAGATTATCTTGGGTTTTGGAGACAAG---AAACTGCCAATCCACCATATCCCTGGGCCAGAAGGTGTTCGTGGTAGAAATTCGGACAACGATCTGATAAAGGAGAAGCTCGGTTGGGCCCCAACAATGCGACTCAAGGATGGACTCCGGATCACTTATATGTGGATCAAGGAGGAGCTAGACAAGGAGATCTCTGAGGGACAG---GATATGGCT---GCTTATAGCTCCTCCAAAGTTGTGGGAACTCAAGCTCCTGTCCAGCTTGGCTCGCTCAGGAAGGCCGATGGCAAGGAG---------------------------------------------------------------------------

>SpGME-1

ATGGGA---AGTGCT------GAT---GGAAGC------TAT------------------GGCGCATACACCTAT---GAGGCCCTCGAGAGGGAGCCATACTGGCCATCTGAAAAGCTCAGAATTTCCATTACTGGAGCTGGTGGTTTTATTGCCTCCCACATTGCTCGACGTTTGAAGGCTGAGGGTCATTATATTATTGCTTCTGACTGGAAGAAGAATGAGCACATGACTGAAGACATGTTCTGTAATGAATTCCATCTTGTTGATCTTAGGGTCATGGATAATTGCCTTAAGGTTACAAAAGGAGTAGACCATGTTTTCAACCTGGCTGCTGATATGGGTGGGATGGGCTTCATTCAGTCCAACCACTCTGTCATTATGTATAACAACACAATGATCAGCTTCAACATGCTTGAAGCTTCCAGGATCAACGGAGTTAAGAGGTTATTCTACGCCTCTAGTGCTTGTATTTACCCTGAATTTAAGCAGCTGGAGACT---AAT------GTG---------AGCCTGAAGGAAGCTGATGCCTGGCCTGCAGAGCCTCAAGATGCTTATGGCTTGGAGAAGCTTGCAACGGAAGAGCTGTGCAAGCATTACACCAAAGACTTTGGAATTGAATGCCGCATTGGAAGGTTCCATAACATTTATGGTCCTTTTGGAACATGGAAAGGTGGCAGGGAGAAGGCACCCGCTGCTTTCTGCAGAAAGGCTATCACTTCTATTGATAAATTTGAGATGTGGGGAGATGGACTTCAAACCCGATCCTTCACATTCATTGATGAATGTGTGGAAGGTGTGCTTAGATTGACAAAGTCAGACTTCCGTGAGCCATTGAACATTGGAAGTGATGAGATGGTTAGCATGAATGGGATGGCTGAGATTGTTCTCAGCTTTGACAACAAG---AATCTCCCTATTCATCACATTCCTGGCCCAGAAGGTGTGCGGGGACGTAACTCCGACAACACGCTCATCAAAGAGAAGCTTGGCTGGGCTCCTACAATGAAGCTGAAGGATGGGCTGAGAATCACATACTTTTGGATCAAGGAACAGATTGAGAAAGAGAAGACACAAGGAATT---GACTTGTCT---ATTTATGGTTCATCTAAAGTGGTGGGAACTCAAGCACCAGTTCAACTGGGCTCACTTCGTGCTGCTGATGGCAATGAA---------------------------------------------------------------------------

>SpGME-2

ATGGGG---AGTGCT------GAT---GGAAGC------TAT------------------GGTGCTTACACCTAT---GAGGCCCTCGAGAGGGAGCATTACTGGCCATCTGAAAAGCTCAGAATTTCCATCACTGGGGCAGGTGGTTTTATTGCCTCCCACATTGCTCGCCGTTTGAAGGCTGAGGGTCATTACATTATTGCTTCTGACTGGAAGAAGAATGAGCACATGACAGAAGACATGTTTTGTCATGAATTTCATCTTGTTGATCTGAGAGTCATGGATAATTGCTTGAAGGTTACAAAAGATGTAGACCATGTTTTCAACCTTGCTGCTGATATGGGCGGGATGGGCTTCATTCAGTCCAACCACTCTGTCATCATGTATAACAACACAATGATCAGCTTCAACATGCTTGAAGCCTCCAGGATCAATGGGGTTAAGAGATTGTTTTATGCCTCTAGTGCTTGTATTTACCCCGAATTTAAGCAGCTGGAGACA---AAT------GTG---------AGCCTGAAGGAATCTGATGCCTGGCCTGCAGAGCCTCAAGATGCTTATGGCCTGGAGAAGCTTGCGACGGAAGAGTTGTGCAAGCATTACACAAAAGACTTTGGAATTGAATGCCGTATCGGAAGATTCCATAACATTTATGGTCCCTTTGGAACATGGAAAGGTGGCAGGGAGAAGGCACCTGCTGCTTTCTGCAGAAAGGGGCTCACTTCCGTTGATAAATTTGAGATGTGGGGAGATGGACTTCAAACCCGATCTTTCACATTCATTGATGAGTGTGTGGAAGGTGTGCTTAGATTGACCAAGTCAGACTTCCGTGAGCCAGTGAACATTGGAAGTGATGAGATGGTTAGCATGAATGAGATGGCTGAAATTATTCTCAGCTTTGAGAACAAG---AATCTCCCCATTCATCACATTCCTGGCCCAGAAGGTGTACGCGGGCGTAACTCCGACAACACACTAATCAAGGAGAAGCTTGGTTGGGCACCTACAATGAGGCTGAAGGATGGGCTGAGAATTACTTACTTTTGGATTAAGGAACAGATCGAGAAAGAGAAGTCTCAAGGAATT---GACCTGTCT---ATCTATGGTTCATCGAAAGTGGTGGGAACTCAAGCACCTGTTCAGTTGGGCTCACTTCGTGCTGCTGATGGTAAAGAA---------------------------------------------------------------------------

>SpGME-3

ATGGGA---AGTGCT------GAT---GGAAGC------TAT------------------GGCGCATACACCTAT---GAGGCCCTCGAGAGGGAGCCATACTGGCCATCTGAAAAGCTCAGAATTTCCATTACTGGAGCTGGTGGTTTTATTGCCTCCCACATTGCTCGACGTTTGAAGGCTGAGGGTCATTATATTATTGCTTCTGACTGGAAGAAGAATGAGCACATGACTGAAGACATGTTCTGTAATGAATTTCATCTTGTTGATCTTAGGGTCATGGATAATTGCCTTAAGGTTACAAAAGGAGTAGACCATGTTTTCAACCTGGCTGCTGATATGGGTGGGATGGGCTTCATTCAGTCCAACCACTCTGTCATTATGTATAACAACACAATGATCAGCTTCAACATGCTTGAAGCTTCCAGGATCAACGGAGTTAAGAGGTTATTCTACGCCTCTAGTGCTTGTATTTACCCTGAATTTAAGCAGCTGGAGACT---AAT------GTG---------AGCCTGAAGGAAGCTGATGCCTGGCCTGCAGAGCCTCAAGATGCTTATGGCTTGGAGAAGCTTGCAACGGAAGAGCTGTGCAAGCATTACACCAAAGACTTTGGAATTGAATGCCGCATTGGAAGGTTCCATAACATTTATGGTCCTTTTGGAACATGGAAAGGTGGCAGGGAGAAGGCACCCGCTGCTTTCTGCAGAAAGGCTATCACTTCTATTGATAAATTTGAGATGTGGGGAGATGGACTTCAAACCCGATCCTTCACATTCATTGATGAATGTGTGGAAGGTGTGCTTAGATTGACAAAGTCAGACTTCCGTGAGCCATTGAACATTGGAAGTGATGAGATGGTTAGCATGAATGGGATGGCTGAGATTGTTCTCAGCTTTGACAACAAG---AATCTCCCTATTCATCACATTCCTGGCCCAGAAGGTGTGCGGGGACGTAACTCCGACAACACGCTCATCAAAGAGAAGCTTGGCTGGGCTCCTACAATGAAGCTGAAGGATGGGCTGAGAATCACATACTTTTGGATCAAGGAACAGATTGAGAAAGAGAAGACACAAGGAATT---GACTTGTCT---ATTTATGGTTCATCTAAAGTGGTGGGAACTCAAGCACCAGTTCAACTGGGCTCACTTCGTGCTGCTGATGGCAATGAA---------------------------------------------------------------------------

>SppGME

ATGGCC---AACGGG---------------------AGTTAT------------------GGGGAATACACCTAC---GAGAACCTGGAGAGGGAGCCCTACTGGCCCTCTGAGAAGCTCAGGATCTCCATTACGGGAGCTGGGGGGTTCATTGCCTCCCACATTGCCCGGCGCCTGAAGAGTGAGGGCCACTACATTATTGCATCCGACTGGAAGAAGAATGAGCACATGACAGAGGACATGTTCTGCCATGAGTTCCACCTCGTTGATCTCAGGGTCATGGAAAACTGCCTGAAAGTAACTAAAGATGTTGACCATGTGTTCAATCTCGCCGCTGACATGGGAGGAATGGGTTTTATTCAGTCCAACCATTCGGTGATCATGTACAACAACACCATGATCAGCTTCAACGTGCTCGAGGCTGCCAGGGTTAATGACGTCAAGAGGCTCTTTTATGCATCCAGCGCCTGCATCTACCCGGAGTTCAAGCAGCTGGAAACA---AAC------GTC---------AGCCTGAAGGAGTCCGACGCCTGGCCAGCTGAGCCCCAAGACGCCTACGGCCTGGAGAAGCTCGCCAGCGAAGAGCTCTGCAAGCACTACACCAAGGACTTCGGCATCGAGTGCCGGATTGGCCGCTTCCACAACATCTACGGCCCCTTCGGCACGTGGAAAGGCGGCCGGGAGAAGGCCCCAGCCGCCTTCTGCAGGAAGGCCATCACCTCCACCGACAAGTTCGAGATGTGGGGCGACGGCCTCCAGACCCGGTCCTTCACCTTCATCGACGAGTGCGTCGAGGGCGTGCTGAGGTTGACCAAGTCGGACTTCCGGGAGCCGGTCAACATCGGGAGCGACGAGATGGTCAGCATGAACGAGATGGCGGAGATCGTCCTCAGCTTCGAGGACCGG---ATGCTCCCCATCCACCACATCCCCGGCCCCGAGGGCGTCCGCGGCCGCAACTCTGACAACACCCTCATCAAGGAGAAGCTCGGGTGGGCCCCCACCATGAGGCTCAAGGACGGGCTGAGGATCACCTACTTCTGGATCAAGGAGCAGATCGAGAAGGAGAAGGCGCAGGGAATC---GACCTGTCG---GTCTACGGGTCGTCCAAGGTGGTGGGCACCCAGGCGCCAGTCCAGCTGGGCTCCCTCCGGGCCGCCGACGGGAAGGAA---------------------------------------------------------------------------

>SvGME-1

ATGGCG------------------------------CTCAAC------------------AAGGAGTACACGTAC---GCGGAGCTGGAGAAGGAGCCATACTGGCCGTTTGAGAAGCTGCGGATCTCCATCACGGGCGCTGGCGGGTTCATCGCCTCCCACATCGCTAGGCGCCTCATGAGCGAGGGCCACTACATCATCGCCTCTGACTGGAAGAAGAACGAGCACATGACCGAGGAAATGTTCTGCCATGAGTTCCACCTCATTGACCTGAGGGTCATGGACAACTGCCTCAAGGTGACCACGGGGGTTGACCATGTGTTTAACCTTGCTGCTGATATGGGAGGGATGGGGTTCATCCAGTCCAACCACTCTGTCATCATGTATAACAACACTATGATCAGCTTTAACATGCTCGAGGCTGCTAGAATCAATGGCGTCAAGAGGTTCTTTTATGCATCAAGCGCCTGTATCTACCCTGAATTTAAGCAGTTGGAGACT---GTA------GTT---------AGCTTGAAGGAGTCAGATGCCTGGCCTGCAGAGCCTCAAGATGCCTATGGCTTGGAGAAACTTGCTACTGAGGAACTGTGCAAGCACTACACAAAGGATTTTGGCATTGAGTGCCGGATCGGTCGCTTTCACAACATATATGGTCCCTTTGGGACATGGAAAGGTGGAAGGGAGAAGGCACCTGCTGCTTTCTGCAGAAAGGCTTTAACCTCCACTGGGCGCTTTGAGATGTGGGGCGATGGTCTGCAAACCAGATCCTTCACATTTATTGATGAATGTGTCGAGGGTGTCCTTAGGTTAACAAAGTCTGATTTCCGTGAGCCTGTAAACATTGGAAGTGATGAAATGGTCAGCATGAATGAGATGGCTGAGATAGTCCTCAGCTTTGAGAACAAG---CAGCTGCCCATCCACCACATTCCTGGACCGGAGGGTGTGCGAGGGCGGAATTCAGATAACACACTCATCAAGGAGAAGCTTGGCTGGGCTCCGACCATGAAGCTGAAGGATGGACTAAGGATCACATACTTCTGGATCAAGGAGCAGCTTGAGAAGGAGAAGGCTGAGGGCATG---GATCTGTCG---GTCTATGGTTCATCCAAGGTCGTGCAGACACAGGCCCCTGTTCAGCTCGGTTCCCTCCGCGCTGCCGATGGCAAGGAG---------------------------------------------------------------------------

>SvGME-2

ATGGGG---AGCAGCGAGAAGACC---GTTACC---GCTTAT------------------GGTGAGTACACCTAT---GCTGAGCTGGAGAGGGAGCCCTACTGGCCGAGTGAGAAGCTGAGGATTTCAATTACTGGGGCTGGTGGTTTCATTGGATCACACATTGCTCGCCGCCTGAAGAGTGAGGGCCATTACATCATCGCCTCTGACTGGAAGAAAAATGAGCATATGACTGAGGACATGTTCTGCCATGAGTTCCACCTTGTTGACCTCAGGGTCATGGACAACTGTCTGAAGGTTACCCAAGGCGTCGACCATGTATTCAATCTTGCTGCTGATATGGGTGGCATGGGGTTCATCCAGTCAAACCACTCTGTCATCATGTACAACAACACCATGATCAGTTTCAACATGCTTGAAGCTGCACGTATCAATGATGTGAAGAGGTTCTTCTACGCCTCGAGTGCATGCATTTACCCTGAATTCAAACAGCTTGACACA---AAT------GTG---------AGCTTGAAGGAATCTGATGCCTGGCCTGCTGAGCCTCAAGACGCCTATGGCTTGGAGAAGCTTGCAACTGAGGAGTTGTGCAAGCACTACACCAAGGACTTTGGCATTGAGTGCCGCGTTGGCCGTTTCCACAACATATATGGTCCCTTTGGAACATGGAAGGGTGGTCGTGAGAAGGCACCTGCTGCCTTCTGCAGAAAGGCTCAGACATCCACCGAGAGGTTTGAGATGTGGGGTGATGGCCTCCAGACCCGATCCTTCACATTCATTGACGAGTGTGTCGAGGGTGTTCTGAGATTGACTAAGTCAGACTTCCGCGAGCCGGTGAACATTGGAAGCGATGAGATGGTGAGCATGAATGAGATGGCTGAGATCGTCCTGAGCTTTGAGGATAGG---AAGCTGCCCATCCACCACATCCCTGGTCCAGAGGGGGTCCGTGGGCGCAACTCCGACAACACTCTCATCAAGGAAAAGCTTGGGTGGGCCCCGACAATGAAGCTCAAGGATGGGCTTCGGTTTACGTACTTCTGGATTAAGGAGCAGATCGAGAAGGAGAAGACCCAGGGCATC---GACATCGCA---GCATACGGGTCCTCCAAGGTGGTGTCGACCCAGGCGCCTGTGCAGCTGGGCTCCCTCCGTGCCGCCGACGGCAAGGAGGGCCTG---------------------------------------------------------------------

>SvGME-3

ATGGGG---AGCAGCGAGAAGACC---GTCAGC---ACTTAT------------------GGTGAGTACACCTAT---GCTGAGCTGGAGAGGGAGCCCTACTGGCCCAGTGAGAAGTTGAGGATTTCCATTACTGGGGCTGGTGGTTTCATTGGATCCCACATTGCTCGCCGTCTGAAAAGCGAGGGCCATTACATCATCGCCTCTGACTGGAAGAAGAATGAGCACATGACTGAGGATATGTTCTGCCATGAGTTCCACCTTGTTGACCTCAGGGTCATGGACAACTGTCTGAAGGTTACCCAAGGCGTCGACCATGTATTCAATCTTGCTGCTGATATGGGTGGCATGGGGTTCATCCAGTCAAACCACTCTGTCATCATGTACAACAACACCATGATCAGTTTCAACATGCTTGAGGCTGCACGCATCAATGGTGTGAAGAGGTTCTTCTATGCCTCGAGTGCGTGCATTTACCCAGAATTCAAGCAGCTTGAGACA---AAT------GTG---------AGCCTGAAGGAATCTGATGCCTGGCCTGCTGAGCCTCAAGATGCCTATGGCTTGGAGAAGCTTGCAACTGAGGAGCTCTGCAAGCACTACACCAAGGACTTTGGCATCGAGTGCCGTGTTGGCCGTTTCCACAACATCTATGGCCCCTTCGGAACATGGAAAGGTGGCCGTGAGAAGGCACCGGCTGCCTTCTGCAGAAAGGCTCAGACATCTACCGAGAGGTTTGAGATGTGGGGCGATGGCCTCCAGACACGGTCCTTCACCTTCATTGACGAGTGCGTTGAGGGTGTCCTGAGGCTGACCAAGTCAGACTTCCGCGAGCCGGTGAACATCGGGAGCGATGAGATGGTGAGCATGAACGAGATGGCCGAGATTGTCCTGAGCTTTGAGGATAGG---AAGCTGCCCATCCACCACATCCCTGGTCCCGAGGGGGTCCGTGGGCGCAACTCTGACAACACCCTTATCAAGGAGAAGCTAGGCTGGGCCCCGACAATGAAGCTCAAGGATGGGCTGAGGTTCACCTACTTCTGGATCAAGGAGCAGATCGAGAAGGAGAAGACGCAGGGTGTC---GACATCGCG---GCGTACGGGTCCTCCAAGGTGGTGTCGACGCAGGCGCCCGTGCAGCTGGGCTCCCTCCGCGCCGCCGACGGCAAGGAGGGCCTC---------------------------------------------------------------------

>TcGME

ATGGGA---AGTGCT------GAT---GGGACC---AACTAT------------------GGTGCTTTCACCTAT---GAGGCCCTGGAGAGGGAGCCTTACTGGCCATCTGAGAAACTCCGAATTTCAATTACTGGGGCAGGTGGGTTCATTGCATCCCACATTGCTCGACGTCTGAAGAGTGAAGGCCATTACATCATTGCTTCTGACTGGAAGAAGAATGAGCACATGACTGAAGATATGTTTTGTCATGAATTCCATCTCGCTGATCTTCGGGTCATGGACAATTGCTTGAAAGTTACCAATGGAGTGGATCATGTTTTCAACCTTGCTGCCGATATGGGTGGGATGGGTTTTATTCAGTCCAATCACTCTGTCATTATGTACAACAACACAATGATCAGTTTCAACATGCTTGAGGCTGCTAGGATCAACGGAGTTAAGAGGTTTTTCTATGCCTCCAGTGCTTGTATCTACCCTGAATTTAAGCAGTTGGAGACT---AAT------GTG---------AGCTTGAAAGAATCCGATGCCTGGCCTGCTGAGCCTCAAGATGCTTATGGCTTGGAGAAGCTTGCGACAGAGGAGTTGTGCAAGCACTACACTAAAGACTTTGGAATTGAATGTCGCATTGGACGGTTCCACAACATTTATGGCCCTTTTGGAACATGGAAAGGTGGAAGGGAGAAGGCTCCAGCTGCCTTTTGCAGAAAAGCTATTACATCCACCGATAAGTTTGAGATGTGGGGAGATGGTCTTCAGACCCGATCTTTCACCTTCATTGATGAATGTGTAGAAGGTGTACTTAGATTGACGAAGTCTGATTTCCGTGAGCCTGTGAACATTGGAAGCGATGAGATGGTTAGCATGAATGAGATGGCTGAGATTGTTCTTAGCTTTGAGGATAAA---AAGCTTCCAATTCATCATATCCCTGGTCCAGAGGGTGTCCGTGGTCGTAATTCAGACAATACATTGATCAAAGAAAAACTTGGGTGGGCTCCCACGATGAGGTTGAAGGATGGCCTGAGAATTACATACTTCTGGATCAAGGAACAAATTGAGAAAGAGAAGTCTCAAGGCATT---GACCTGACT---ATTTATGGGTCATCTAAGGTGGTGGGAACTCAAGCACCAGTTCAGTTGGGCTCACTTCGTGCTGCAGACGGCAAAGAA---------------------------------------------------------------------------

>TpGME-1

ATGGGA---AGTACT------GGG---AAAACT---AACTAT------------------GGAGAATACACCTAT---GAGAATCTTGAGAGAGAGCCTTATTGGCCATCAGAAAAACTTAAAATTTCAATCACTGGTGCCGGGGGTTTTATTGCGTCTCACTTAGCGCGTCGCCTTAAGACCGAGGGACATTACATTATTGCTTCTGATTGGAAGAAGAATGAGCACATGACTGAGGATATGTTCTGTGATGAATTCCATCTTGTTGATCTTAGGGTCATGGATAACTGTTTAACAGTTACGAAAGGGGTTGACCATGTTTTCAATCTTGCCGCTGATATGGGTGGAATGGGTTTTATTCAGTCCAATCATTCTGTTATTATGTATAATAACACTATGATTAGTTTCAATATGATTGAGGCTGCTAGGATTAATGGCATTAAGAGGTTCTTTTATGCCTCGAGTGCTTGTATCTACCCTGAATTTAAACAGTTGGAAACTACTAAT------GTG---------AGCTTGAAGGAGTCTGATGCATGGCCTGCTGAGCCACAAGATGCGTACGGGCTAGAGAAGCTTGCAACAGAGGAGATATGCAAGCACTATAACAAAGATTTTGGAATTGAGTGCCGCATTGGGAGGTTCCATAACATATATGGTCCTTTCGGGACATGGAAAGGTGGAAGGGAAAAGGCTCCTGCTGCTTTTTGTCGTAAAGCAATCACATCCACAGACAAATTCGAGATGTGGGGAGATGGTTTACAAACACGGTCATTCACCTTCATTGATGAGTGTGTTGAAGGTGTGCTTAGACTGACTAAATCCGACTTCCGTGAGCCGGTAAATATTGGAAGTGATGAAATGGTCAGCATGAATGAAATGGCTGAGATTGTTCTTGGTTTTGACAACAAG---AAGACTCCTATCCATCACATTCCAGGTCCAGAAGGTGTTCGTGGTCGTAACTCAGACAATACACTTATAAAAGAGAAACTTGGCTGGGCTCCAACAATGAAGTTGAAGGATGGTTTGAGGATTACATACATCTGGATTAAGGAGCAGCTTGAAAAGGAGAATGCTCAAGGTATT---GATACATCA---GGATATGGATCATCAAAAGTGGTGCAAACCCAAGCTCCAGTTCAATTAGGCTCACTTCGGGCCGCAGACGGCAAAGAGAGTGGT---------------------------------------------------------------------

>TpGME-2

ATGGGA---AGTTCT------GGA---ACAAAC---GATTAT------------------GGTTCATACACTTAC---CAGAACCTTGAAAGAGAGCCTTATTGGCCATCAGAAAAGCTAAGAATTTCAATAACTGGTGCTGGTGGTTTTATTGCCTCACACATTGCTCGCCGTCTTAAGACCGAAGGGCATTACATTATTGCTTCTGATTGGAAGAAAAATGAACACATGACTGAAGACATGTTCTGTCATGAGTTTCATCTTGTTGATCTTAGGGTCATGGATAACTGTCTCAAAGTTACCGAGAATGTGGATCATGTTTTCAATCTTGCTGCTGATATGGGTGGTATGGGTTTTATCCAATCCAACCACTCTGTCATTATGTACAACAACACTATGATTAGTTTCAACATGATTGAGGCTGCTAGGATTAATGGTGTTAAGAGGTTTTTTTATGCCTCTAGTGCTTGTATCTACCCTGAATTTAAACAGCTGGAAACG---AAT------GTG---------AGCTTGAAGGAGGCTGATGCATGGCCTGCTGAGCCACAAGATGCATATGGGCTGGAGAAGCTTGCAACAGAAGAGTTATGCAAGCATTATAACAAAGATTTTGGAATTGAGTGCCGCATTGGGCGGTTCCATAACATTTATGGCCCTTATGGTACATGGAAAGGTGGAAGGGAGAAGGCTCCTGCTGCTTTTTGTCGAAAGACACTTACTTCCACGGACAAATTTGAGATGTGGGGAGATGGATTGCAGACACGATCCTTCACCTTCATTGACGAGTGTGTTGAAGGCGTGCTGAGATTGACTAAATCAGACTTCCGAGAGCCAGTGAATATTGGAAGTGACGAAATGGTTAGCATGAATGAGATGGCTGAGATTGTTCTTAGCTTTGAGGACAAA---AGCATACCCATACAGCACATTCCTGGTCCAGAGGGTGTCCGTGGCCGCAATTCAGACAATACACTTATCAAAGAGAAACTTGGCTGGGCTCCAACAATGAAGTTGAAGGATGGGCTGAGAATTACATACTTCTGGATTAAAGAGCAGCTTGAGAAAGAGAAGGCTGGAGGTGTT---GATGTAACA---TCCTATGGATCATCCAAAGTGGTGTCGACTCAAGCCCCTGTTCAACTTGGTTCACTTCGGGCAGCAGATGGCAATGAA---------------------------------------------------------------------------

>VcGME

ATGTCG---GCCGAT------ACGGTGCATGAG---CAGTAT------------------GCCTCTGTGTCCAAGCTTGCGAAATATCCATTTGAGCCGTATTGGCCTGACAAGAAGCTGAAGATTTGTGTTACGGGCGCCGGGGGCTTCATTGCTAGCCATTTGGCTAAGCGTCTTAAGTCAGAAGGGCACTACATTGTTGCTTGCGACTGGAAGCGGAACGAGCACTTCGCGGAGGAGGAGTTCTGCCACGAGTTTCATCTGGTGGACCTCCGGTTGTTCGAGAACTGCAAGAAGGTAGCCGAGGGATGCGAGCACGTCTTCAACCTTGCTGCGGACATGGGTGGCATGGGTTTCATCCAGTCCAACCATTCCGTCATTTTGTACAACAATACTATGGTGTCGTTCAACATGATGGAGGCTGCACGGGTCTGTGGCGTGAAGAGATTCTTCTATGCCTCGTCCGCCTGCATCTACCCGGAGTTCAAGCAGCTTGACACC---CAG------GTGGAGGGCGGTGGCCTCAAGGAGGCGGATGCATGGCCCGCGCAGCCTCAAGATGCATACGGTCTTGAAAAGCTTGTAAGCGAGGAGCTGGGCAAGCATTACGGCAAGGACTTCGGAATTGATGTCCGCCTCGCGCGCTTCCACAACATCTACGGCCCGCATGGCACGTGGAAGGGTGGCCGTGAGAAGGCTCCCGCTGCCTTCTGTCGCAAGGTTCTCACGTCCACCACGGAGATTGAGATGTGGGGTGACGGCAAGCAAACTCGCAGCTTCACCTTCATCGATGACTGCGTGGAGGGCATCTTGCGGATTACCAAGTCCGACTTTACGGAGCCCCTGAATTTGGGATCTACCGAAATGGTGTCCATGAACGAAATGATGGAAATGGCCATGTCATTTGAAGACAAG---AAGCTGCCGATAAAGCACATCCCAGGTCCTGAGGGTGTGCGTGGCCGCAACTCGGACAACAAGCTTATCTTGGAGAAGCTTGGCTGGGAGCCCACAGTGTCGCTCCGGGACGGCCTCAAGATGACGTACTTTTGGATCAAGTCCCAAATCGAGAAGGAGGCTGAGAGCGGCGTG---GACGCCAGC---AAGTACAGCCATAGCACAATTGTGCAGACCAGCGCGCCGGTGGAGCTCGGTTCGCTTCGCAAGGCTGATGGACAGGAAGGATTC---------------------------------------------------------------------

>ZmGME-1

ATGGGG---AGCAGCGAGAAGACC---GTTACC---GCTTAC------------------GGCGAGTACACCTAC---GCTGAGCTGGAGAGGGAGCCCTACTGGCCGAGTGAGAAGCTGAGGATTTCGATTACTGGGGCTGGTGGTTTCATTGGATCCCACATTGCTCGCCGTCTGAAGAACGAGGGCCATTACATCATTGCCTCTGACTGGAAGAAGAACGAGCACATGACCGAGGACATGTTCTGCCATGAGTTCCACCTCGTTGATCTCAGGGTCATGGACAACTGTCTCAAGGTCACCCATGGTGTCGACCATGTCTTCAATCTTGCTGCAGATATGGGTGGCATGGGGTTCATCCAGTCAAATCACTCTGTGATCATGTACAACAACACCATGATCAGTTTTAACATGCTGGAGGCTGCACGTATCAATGGTGTGAAGAGGTTCTTCTATGCCTCGAGTGCATGCATTTACCCTGAGTTCAAGCAGCTTGACACA---AAT------GTG---------AGCTTGAAGGAATCTGATGCCTGGCCTGCTGAGCCTCAAGATGCCTATGGCTTGGAGAAGCTTGCAACCGAGGAGCTGTGCAAGCACTACACCAAGGACTTTGGCATCGAGTGCCGCGTCGGCCGCTTCCACAACATCTACGGCCCTTTCGGAACATGGAAAGGTGGCCGCGAGAAGGCACCGGCTGCCTTCTGCAGAAAGGCTCAGACATCCACGGAGAGGTTTGAGATGTGGGGCGATGGTCTCCAGACCAGGTCCTTCACCTTCATCGACGAGTGCGTGGAGGGTGTTCTGAGATTGACCAAGTCAGACTTCCGCGAGCCAGTGAACATCGGAAGCGATGAGATGGTGAGCATGAACGAGATGGCTGAGATCGTGCTGGGCTTTGAGGATAGG---AAGCTGCCCATCCACCACATCCCTGGCCCGGAAGGGGTCCGCGGGCGCAACTCTGACAACACCCTTATCAAGGAGAAGCTTGGCTGGGCCCCGACGATGAAGCTCAAGGATGGGCTGCGGTTCACCTACTTCTGGATCAAAGAGCAGATCGAGAAGGAGAAGACGCAGGGGGTC---GACATCGCG---GCGTACGGGTCGTCCAAGGTGGTGTCCACCCAGGCGCCCGTGCAGCTGGGCTCCCTCCGCGCCGCCGACGGCAAGGAGGGCCTC---------------------------------------------------------------------

>ZmGME-2

ATGGCG------------------------------CTCAAC------------------AAGGAGTACACGTAC---GCGGAGCTGGAGAAGGAGCCGTACTGGCCGTTCGAGAAGCTGCGGGTCTCCATCACCGGGGCCGGCGGGTTCATCGCCTCCCACATCGCGCGCCGCCTCAAGGGCGAGGGCCACTACGTCGTCGCCTCCGACTGGAAGAGGAACGAGCACATGCCCGAGGACATGTTCTGCCACGAGTTCCACCTCGTGGACCTGAGGGTCATGGACAACTGCCTCAAGGTCACCACGGGGGTTGACCACGTGTTCAACCTCGCGGCTGACATGGGTGGGATGGGGTTCATCCAGTCCAACCACTCCGTCATCATGTACAACAACACCATGATCAGCTTTAACATGCTTGAGGCTGCTAGAATTAACGGCGTCAAGAGGTTCTTCTATGCATCAAGTGCCTGCATCTACCCTGAGTTCAAGCAGCTGGAAACT---GTA------GTT---------AGCTTGAAGGAGTCCGATGCTTGGCCTGCCGAGCCTCAAGATGCCTATGGCCTGGAAAAACTCGCGACTGAGGAGCTGTGCAAGCACTATACAAAGGATTTTGGCATCGAGTGCCGGATTGGTCGCTTTCACAACATATATGGTCCCTTCGGCACATGGAAAGGTGGAAGGGAGAAGGCGCCTGCTGCTTTCTGCAGGAAGGCTCTGACCTCCACAGGGCGCTTTGAGATGTGGGGTGATGGCCTGCAAACCAGATCCTTCACATTTATTGATGAATGTGTTGAAGGTGTCCTTAGGTTAACAAAGTCCGATTTCCGTGAGCCTGTAAACATTGGAAGCGACGAAATGGTGAGCATGAACGAGATGGCGGAAATGGTCCTGAGCTTTGAGAACAAG---CAGCTGCCCATCCACCACATCCCCGGGCCGGAGGGCGTGCGTGGGCGCAACTCAGACAACACGCTCATCAAGGAGAAGCTTGGCTGGGCTCCGACTATGAGGCTGAAGGACGGGCTGAGGATCACGTACTCCTGGATCAAGGAGCAGCTTGAGAAGGAGAAGGCCGAGGGCATG---GATCTGTCG---GTCTACGGATCATCCAAGGTCGTGCAGACACAGGCCCCGGTTCAGCTCGGCTCCCTCCGCGCCGCTGATGGCAAGGAG---------------------------------------------------------------------------
