# Supplementary material for: Molecular Evolution of GDP-D-Mannose Epimerase (GME), a Key Gene in Plant Ascorbic Acid Biosynthesis
Source: Front Plant Sci. 2018 Sep 4;9:1293. doi: 10.3389/fpls.2018.01293 (PMC6132023; doi:10.3389/fpls.2018.01293)
Supplement: Supplementary file 7 [file Table_7.DOCX]

**Supplemental Data 2. Alignment of plant GME protein sequences used in this study**

>AcGME

MG-ST--DAAT-KY------GEYTY-EQLERELYWPSEKLRISITGAGGFIASHIARRLKTEGHYIIASDWKKNEHMPEDMFCHEFHLADLRVMDNCMKVTSGVDHVFNLAADMGGMGFIQSNHSVIMYNNTMISFNMLEAARINGVKRFFYASSACIYPEFKQLETSN--V---SLKEADAWPAEPQDAYGLEKLATEELCKHYTKDFGIECRIGRFHNIYGPFGTWKGGREKAPAAFCRKALTAIDKFEMWGDGLQTRSFTFIDECVEGVLRLTKSDFREPVNIGSDEMVSMNEMAEIVLGFEDK-KLPIQHIPGPEGVRGRNSDNTLIKEKLGWAPTMRLKDGLRITYFWIKEQIEKEKTRGI-DLS-VYGSSKVVGTQAPVQLGSLRAADGKE-------------------------

>AdGME

MG-ST--S-ES-NY------GSYTY-ENLEREPYWPEAKLRISITGAGGFIASHIARRLKGEGHYIIASDWKKNEHMTEDMFCHEFHLVDLRVMDNCLKVTTGVDHVFNLAADMGGMGFIQSNHSVIMYNNTMISFNMLEAARVNGVKRFFYASSACIYPEFKQLDT-N--V---SLKESDAWPAEPQDAYGLEKLATEELCKHYTKDFGIECRIGRFHNIYGPFGTWKGGREKAPAAFCRKTLTSTDRFEMWGDGLQTRSFTFIDECVEGVLRLTKSDFREPVNIGSDEMVSMNEMAEIVLSFENK-KLPIHHIPGPEGVRGRNSDNTLIKEKLGWAPTMKLKDGLRFTYFWIKEQLEKEKAQGI-DLS-TYGSSKVVGTQAPVQLGSLRAADGKE-------------------------

>AeGME

MG-ST--S-ES-NY------GSYTY-ENPEREPYWPEAKLRISITGAGGFIASHIARRLKGEGHYIIASDWKKNEHMTEDMFCHEFHLVDLRVMDNCLKVTTGVDHVFNLAADMGGMGFIQSNHSVIMYNNTMISFNMLEAARVNGVKRFFYASSACIYPEFKQLDT-N--V---SLKESDAWPAEPQDAYGLEKLATEELCKHYTKDFGIECRIGRFHNIYGPFGTWKGGREKAPAAFCRKTLTSTDRFEMWGDGLQTRSFTFIDECVEGVLRLTKSDFREPVNIGSDEMVSMNEMAEIVLSFENK-KLPIHHIPGPEGVRGRNSDNPLIKEKLGWAPTMKLKDGLRFTYFWIKEQLEKEKAQGI-DLS-TYGSSKVVGTQAPVQLGSLRAADGKE-------------------------

>AhGME

MG-TT--N-GS-DY------GAYTY-KELEREPYWPSEKLKISITGAGGFIASHIARRLKHEGHYVIASDWKKNEHMTEDMFCDEFHLVDLRVMENCLKVTKGVDHVFNLAADMGGMGFIQSNHSVIMYNNTMISFNMIEAARINGIKRFFYASSACIYPEFKQLETTN--V---SLKESDAWPAEPQDAYGLEKLATEELCKHYNKDFGIECRIGRFHNIYGPFGTWKGGREKAPAAFCRKALTSTDRFEMWGDGLQTRSFTFIDECVEGVLRLTKSDFREPVNIGSDEMVSMNEMAEMVLSFEEK-KLPIHHIPGPEGVRGRNSDNNLIKEKLGWAPNMRLKEGLRITYFWIKEQIEKEKAKGS-DVS-LYGSSKVVGTQAPVQLGSLRAADGKE-------------------------

>AlGME

MG-TT--N-GS-DY------GAYTY-KELEREPYWPSEKLKISITGAGGFIASHIARRLKHEGHYVIASDWKKNEHMTEDMFCDEFHLVDLRVMENCLKVTKGVDHVFNLAADMGGMGFIQSNHSVIMYNNTMISFNMIEAARINGIKRFFYASSACIYPEFKQLETTN--V---SLKESDAWPAEPQDAYGLEKLATEELCKHYNKDFGIECRIGRFHNIYGPFGTWKGGREKAPAAFCRKALTSTDRFEMWGDGLQTRSFTFIDECVEGVLRLTKSDFREPVNIGSDEMVSMNEMAEMVLSFEEK-KLPIHHIPGPEGVRGRNSDNNLIKEKLGWAPNMRLKEGLRITYFWIKEQIEKEKAKGS-DVS-LYGSSKVVGTQAPVQLGSLRAADGKE-------------------------

>AmhGME

MG-SN--D-GF-DY------GAYTY-DKLEREPYWPSEKLRISITGAGGFIGSHIARRLKSEGHYVIASDWKKNEHMTEDMFCNEFHLVDLRVMDNCLTVTKDVDHVFNLAADMGGMGFIQSNHSVIMYNNTMISFNMLEAARINGVKRFFYASSACIYPEFKQLETAN--V---SLKEADAWPAEPQDAYGLEKLATEELCKHYTKDFGIECRIGRFHNIYGPFGTWKGGREKAPAAFCRKAITSTDKFEMWGDGLQTRSFTFIDECVEGVLRLTKSDFREPVNIGSDEMVSMNEMAEIVLSFEDR-KLPIHHIPGPEGVRGRNSDNTLIKEKLGWAPTMRLKDGLRITYFWIKEQIEKEKSQGV-DLS-VYGSSKVVTTQAPVQLGSLRAADGKE-------------------------

>AmtGME

MG-SAGKE-AT-SY------GEYTY-ANLERESYWPSEKLRISITGAGGFIASHIARRLKSEGHYIIASDWKKNEHMPEDMFCHEFHLVDLRVMDNCLKVTTGVDHVFNLAADMGGMGFIQSNHSVIMYNNTMISFNMLEAARINGVKRFFYASSACIYPEFKQLET-N--V---SLKESDAWPAEPQDAYGLEKLATEELCKHYTKDFGIECRVGRFHNIYGPFGTWKGGREKAPAAFCRKAITSTDKFEMWGDGLQTRSFTFIDECVEGVLRLTKSDFREPVNIGSDEMVSMNEMAEIVLSFEDK-KLPIHHIPGPEGVRGRNSDNTLIKEKLGWAPTMKLKDGLRFTYFWIKDQIEKEKAQGI-DLS-VYGSSKVVGTQAPVQLGSLRAADGKE-------------------------

>AncGME-1

MG-GAENN-GT-NY------GEYTY-AELEREPYWPSEKLRISITGAGGFIASHIARRLKSEGHYIIASDWKKNEHMDEDMFCHEFHLADLRVMDNCLKVTDGVDHVFNLAADMGGMGFIQSNHSVIMYNNTMISFNMLEAARINGVKRLFYASSACIYPEFKQLET-N--V---SLKESDAWPAEPQDAYGLEKLATEELCKHYNKDFGIECRVGRFHNIYGPFGTWKGGREKAPAAFCRKALTSTDRFEMWGDGLQTRSFTFIDECVEGVLRLTKSDFREPVNIGSDEMVSMNEMAEIVLSFENK-QLPIHHIPGPEGVRGRNSDNTLIKEKLGWAPTMKLRDGLRFTYFWIKEQIEKEKGQGL-DIS-VYGSSKVVQTQAPVQLGSLRAADGKE-------------------------

>AncGME-2

MG-STGNE-GT-IY------GEYTY-AELEREPYWPTEKLRISVTGAGGFIGSHIARRLKSEGHYIIASDWKKNEHMTEDMFCHEFHLVDLRVMDNCLKVTTGVDHVFNLAADMGGMGFIQSNHSVIMYNNTMISFNMLEAARINSVKRFFYASSACIYPEFKQLDT-N--V---SLKESDAWPAEPQDAYGLEKLATEELCKHYTKDFGIECRIGRFHNIYGPFGTWKGGREKAPAAFCRKTLTSTDRFEMWGDGLQTRSFTFIDECVEGVLRLTKSDFREPVNIGSDEMVSMNEMAEIVLSFEDK-KLPIHHIPGPEGVRGRNSDNTLIKEKLGWAPSMRLRDGLRITYFWIKEQLEKEKVAGV-DLS-LYGSSKVVQTQAPVQLGSLRAADGNE-------------------------

>ArGME

MG-ST--S-ES-NY------GSYTY-ENLEREPYWPEAKLRISITGAGGFIASHIARRLKGEGHYIIASDWKKNEHMTEDMFCHEFHLVDLRVMDNCLKVTTGVDHVFNLAADMGGMGFIQSNHSVIMYNNTMISFNMLEAARVNGVKRFFYASSACIYPEFKQLDT-N--V---SLKESDAWPAEPQDAYGLEKLATEELCKHYTKDFGIECRIGRFHNIYGPFGTWKGGREKAPAAFCRKTLTSTDRFEMWGDGLQTRSFTFIDECVEGVLRLTKSDFREPVNIGSDEMVSMNEMAEIVLSFENK-KLPIHHIPGPEGVRGRNSDNTLIKEKLGWAPTMKLKNGLRFTYFWIKEQLEKEKAQGI-DLS-TYGSSKVVGTQAPVQLGSLRAADGKE-------------------------

>AtGME

MG-TT--N-GT-DY------GAYTY-KELEREQYWPSENLKISITGAGGFIASHIARRLKHEGHYVIASDWKKNEHMTEDMFCDEFHLVDLRVMENCLKVTEGVDHVFNLAADMGGMGFIQSNHSVIMYNNTMISFNMIEAARINGIKRFFYASSACIYPEFKQLETTN--V---SLKESDAWPAEPQDAYGLEKLATEELCKHYNKDFGIECRIGRFHNIYGPFGTWKGGREKAPAAFCRKAQTSTDRFEMWGDGLQTRSFTFIDECVEGVLRLTKSDFREPVNIGSDEMVSMNEMAEMVLSFEEK-KLPIHHIPGPEGVRGRNSDNNLIKEKLGWAPNMRLKEGLRITYFWIKEQIEKEKAKGS-DVS-LYGSSKVVGTQAPVQLGSLRAADGKE-------------------------

>BdGME-1

MG-STDKT-GT-PY------GEYTY-AELERELYWPSEKLRISITGAGGFIGSHIARRLKSEGHYIIASDWKKNEHMTEDMFCHEFHLVDLRVMDNCLKVTNGVDHVFNLAADMGGMGFIQSNHSVIMYNNTMISFNILEAGRINGVKRFFYASSACIYPEFKQLET-N--V---SLKEADAWPAEPQDAYGLEKLATEELCKHYTKDFAIECRVGRFHNIYGPFGTWKGGREKAPAAFCRKAQTSTERFEMWGDGLQTRSFTFIDECVEGVLRLTKSDFREPVNIGSDEMVSMNEMAEIVLGFEDK-KLPIHHIPGPEGVRGRNSDNTLIKEKLGWAPTMRLKDGLRFTYFWIKEQIEKERTEGM-DVA-RYGSSKVVSTQAPVQLGSLRAADGKE-------------------------

>BdGME-2

MA----------LN------KEYTY-ADLEKEPYWPFEKLRISITGAGGFIASHIARRLKGEGHYIIASDWKKNEHMEEDMFCHEFHLADLRVMDNCLKVTTGVDHVFNLAADMGGMGFIQSNHSVIMYNNTMISFNMLEAARINGIKRFFYASSACIYPEFKQLET-V--V---SLKEADAWPAEPQDAYGLEKLATEELCKHYTKDFGIECRVGRFHNIYGPYGTWKGGREKAPAAFCRKAQTSTDRFEMWGDGLQTRSFTFIDECVEGVLRLTKSDFREPVNIGSDEMVSMNEMAEIVLGFENK-QLPIHHIPGPEGVRGRNSDNTLIKEKLGWAPTMRLKDGLRITYFWIKEQLEKERAEGG-DVS-AYGSSKVCTTQAPVQLGSLRAADGKE-------------------------

>BrGME-1

MAAAT--N-GSTDY------GAYTY-KELERELYWPSEKLRISITGAGGFIASHIARRLKHEGHYVIASDWKKNEHMTEDMFCDEFHLVDLRVMENCLKVTDKVDHVFNLAADMGGMGFIQSNHSVIMYNNTMISFNMIEAARINGIKRFFYASSACIYPEFKQLETTN--V---SLKESDAWPAEPQDAYGLEKLATEELCKHYNKDFGIECRIGRFHNIYGPFGTWKGGREKAPAAFCRKALTSTDRFEMWGDGLQTRSFTFIDECVEGVLRLTKSDFREPVNIGSDEMVSMNEMAEMVLSFEEK-KLPIHHIPGPEGVRGRNSDNNLIKEKLGWAPTMRLKEGLRITYFWIKEQIEKEKAKGS-DVT-LYGSSKVVGTQAPVQLGSLRAADGKE-------------------------

>BrGME-2

MASTT--N-GSTDY------GAYTY-KDLSRELYWPSHKLKISITGAGGFIASHIARRLKHEGHYVIASDWKKNEHMTEDMFCDEFHLVDLRVMENCLKVTDGVDHVFNLAADMGGMGFIQSNHSVIMYNNTMISFNMIEAARINGIKRFFYASSACIYPEFKQLETSN--V---SLKESDAWPAEPQDAYGLEKLATEELCKHYNKDFGIECRIGRFHNIYGPFGTWKGGREKAPAAFCRKALTSTDRFEMWGDGLQTRSFTFIDECVEGVLRLTKSDFREPVNIGSDEMVSMNEMAEMVLSFEEK-KLRIHHIPGPEGVRGRNSDNKLIKEKLGWAPTMRLKEGLRITYFWIKEQIEKKKAKGS-DVS-LYGSSKVVGTQAPVQLGSLRAADGKE-------------------------

>BrsGME-1

MG-STDKT-GT-PY------GEYTY-AELERELYWPSEKLRISITGAGGFIGSHIARRLKSEGHYIIASDWKKNEHMTEDMFCHEFHLVDLRVMDNCLKVTSGVDHVFNLAADMGGMGFIQSNHSVIMYNNTMISFNMLEAGRINGVKRFFYASSACIYPEFKQLET-N--V---SLKEADAWPAEPQDAYGLEKLATEELCKHYTKDFAIECRVGRFHNIYGPFGTWKGGREKAPAAFCRKAQTSTERFEMWGDGLQTRSFTFIDECVEGVLRLTKSDFREPVNIGSDEMVSMNEMAEIVLSFEDK-KLPIHHIPGPEGVRGRNSDNTLIKEKLGWAPTMKLKDGLRFTYFWIKEQIEKERTEGM-DVA-RYGSSKVVSTQAPVQLGSLRAADGKE-------------------------

>BrsGME-2

MA----------LN------KEYTY-ADLEKEPYWPFEKLRISITGAGGFIASHIARRLKGEGHYIIASDWKKNEHMDEDMFCHEFHLADLRVMDNCLKVTTGVDHVFNLAADMGGMGFIQSNHSVIMYNNTMISFNMLEAARINGIKRFFYASSACIYPEFKQLET-V--V---SLKEADAWPAEPQDAYGLEKLATEELCKHYTKDFGIECRVGRFHNIYGPYGTWKGGREKAPAAFCRKAQTSTDRFEMWGDGLQTRSFTFIDECVEGVLRLTKSDFREPVNIGSDEMVSMNEMAEIVLGFENK-QLPIHHIPGPEGVRGRNSDNTLIKEKLGWAPTMRLKDGLRITYFWIKEQLEKERAEGG-DVS-AYGSSKVCTTQAPVQLGSLRAADGKE-------------------------

>BsGME

MG-TT--N-GS-DY------GAYTY-KELEREPYWPSEKLKISITGAGGFIASHIARRLKHEGHYVIASDWKKNEHMTEDMFCDEFHLVDLRVMENCLKVTKGVDHVFNLAADMGGMGYIQSNHSVIMYNNTMISFNMIEAARINGIKRFFYASSACIYPEFKQLETTN--V---SLKESDAWPAEPQDAYGLEKLATEELCKHYNKDFGIECRIGRFHNIYGPFGTWKGGREKAPAAFCRKALTSTDRFEMWGDGLQTRSFTFIDECVEGVLRLTKSDFREPVNIGSDEMVSMNEMAEMVLSFEVK-KLPIHHIPGPEGVRGRNSDNNLIKEKLGWAPNMRLKEGLRITYFWIKEQIEKEKEKGS-DVS-LYGSSKVVGTQAPVQLGSLRAADGKE-------------------------

>CcGME

MG-ST--E-GT--Y------GAYTY-EELEREPYWPSEKLRISVTGAGGFIASHIARRLKSEGHYIIASDWKKNEHMTEDMFCHEFHLVDLRVMDNCLKVTKGVDHVFNLAADMGGMGFIQSNHSVIMYNNTMISFNMLEASRISGVKRFFYASSACIYPEFKQLET-N--V---SLKESDAWPAEPQDAYGLEKLASEELCKHYTKDFGIECRVGRFHNIYGPFGTWKGGREKAPAAFCRKALTSTDKFEMWGDGLQTRSFTFIDECVEGVLRLTKSDFREPVNIGSDEMVSMNEMAEIVLSFEDK-KLPIHHIPGPEGVRGRNSDNTLIKEKLGWAPSMKLKDGLRITYFWIKEQIEKEKTQGI-DLS-VYGSSKVVGTQAPVQLGSLRAADGKE-------------------------

>CgGME

MG-TT--N-GT-DY------GAYTY-KELEREPYWPSEKLKISITGAGGFIASHIARRLKHEGHYVIASDWKKNEHMTEDMFCDEFHLVDLRVMENCLKVTKGVDHVFNLAADMGGMGFIQSNHSVIMYNNTMISFNMIEAARINGIKRFFYASSACIYPEFKQLETTN--V---SLKESDAWPAEPQDAYGLEKLATEELCKHYNKDFGIECRIGRFHNIYGPFGTWKGGREKAPAAFCRKALTSTDKFEMWGDGLQTRSFTFIDECVEGVLRLTKSDFREPVNIGSDEMVSMNEMAEMVLSFEEK-KLPIQHIPGPEGVRGRNSDNNLIKEKLGWAPNMRLKEGLRITYFWIKEQIEKEKAKGS-DVT-LYGSSKVVGTQAPVQLGSLRAADGKE-------------------------

>ChrGME

MA-TA--AVHE-DY------ASVSKLAKYPFEPYWPSAKLRICVTGAGGFIASHLAKRLKSEGHYIVACDWKRNEHFAEEEFCHEFHLVDLRVYDNCKKVCEGCEHVFNLAADMGGMGFIQSNHSVIMYNNTMVSFNMMEAARVTGIKRFFYASSACIYPEYKQLDV-E--VEGGGLKEDDAWPAQPQDAYGLEKLATEELCKHYNKDFGIECRIARFHNIYGPHGTWKGGREKAPAAFCRKVLTSTSEIEMWGDGKQTRSFTFIDDCVEGILRITKSDFRDPLNLGSTEMVSMNGMMELAMSFDDK-KLPIKHIPGPEGVRGRNSDNKLILEKLGWEPTVTLADGLKRTYEWIKGQLDAEKEKGV-DAT-KYSHSTIVQTSAPIELGSLRKADGEEGFE----------------------

>CosGME

MA-TN--TLNS-EY------YESSKLAKFPFEPYWPEQKLKICVTGAGGFIASHLAKRLKSEGHYLVCADWKRNSFMPEEAFCDEFHLVDLRVYDNCKKVVKGCDHVFNLAADMGGMGFIQSNHSVIMYNNTMISFNMMEVARIEGIKRFFYASSACIYPENRQLET-E--IEGGGLKEDTAWPAQPQDAYGLEKLASEELAMHYDKDFGIECRIARFHNIYGPYGTWKGGREKAPAAFCRKVLTSPKDIEMWGDGLQTRSFTFIDDCVEGILRITKSDYKEPLNLGSSEMVSMNEMMETIKGFEAK-DLPIRHIPGPEGVRGRNSDNALILEKIGWEPTIKLADGLRVTYTWIKSQLEEEA--GT-DAS-VYASSTIVQTSAPKELGTLRQADGDEGFASKAAAKVAKLANGVANGLKTSA

>CpGME

MG-SN--D-GT-DY------GAYTY-KELEREPYWPSEKLRISITGAGGFIASHIARRLKSEGHYIIASDWKKNEHMTEDMFCHEFHLVDLRVMDNCLKVTKGVDHVFNLAADMGGMGFIQSNHSVIMYNNTMISFNMLEASRINGVKRFFYASSACIYPEFKQLET-N--V---SLKESDAWPAEPQDAYGLEKLATEELCKHYTKDFGIECRIGRFHNIYGPFGTWKGGREKAPAAFCRKAITSTDKFEMWGDGIQTRSFTFIDECVEGVLRLTKSDFREPVNIGSDEMVSMNEMAEIVLSFENK-KLPIQHIPGPEGVRGRNSDNTLIKEKLGWAPTMKLKDGLRITYFWIKEQIEKEKSQGI-DLS-IYGSSKVVGTQAPVQLGSLRAADGKE-------------------------

>CrGME

MG-TT--N-GT-DY------GAYTY-KELEREPYWPSEKLKISITGAGGFIASHIARRLKHEGHYVIASDWKKNEHMTEDMFCDEFHLVDLRVMENCLKVTKGVDHVFNLAADMGGMGFIQSNHSVIMYNNTMISFNMIEAARINGIKRFFYASSACIYPEFKQLETTN--V---SLKESDAWPAEPQDAYGLEKLATEELCKHYNKDFGIECRIGRFHNIYGPFGTWKGGREKAPAAFCRKALTSTDKFEMWGDGLQTRSFTFIDECVEGVLRLTKSDFREPVNIGSDEMVSMNEMAEMVLSFEEK-KLPIQHIPGPEGVRGRNSDNNLIKEKLGWAPNMRLKEGLRITYFWIKEQIEKEKAKGS-DVT-LYGSSKVVGTQAPVQLGSLRAADGKE-------------------------

>CsGME

MG-ST--E-GT--Y------GAYTY-EELEREPYWPSEKLRISVTGAGGFIASHIARRLKSEGHYIIASDWKKNEHMTEDMFCHEFHLVDLRVMDNCLKVTKGVDHVFNLAADMGGMGFIQSNHSVIMYNNTMISFNMLEASRISGVKRFFYASSACIYPEFKQLET-N--V---SLKESDAWPAEPQDAYGLEKLASEELCKHYTKDFGIECRVGRFHNIYGPFGTWKGGREKAPAAFCRKALTSTDKFEMWGDGLQTRSFTFIDECVEGVLRLTKSDFREPVNIGSDEMVSMNEMAEIVLSFEDK-KLPIHHIPGPEGVRGRNSDNTLIKEKLGWAPSMKLKDGLRITYFWIKEQIEKEKTQGI-DLS-VYGSSKVVGTQAPVQLGSLRAADGKE-------------------------

>CusGME

MG-SA--G-ET-TY------GSYTY-QELEREAYWPSEKLRISITGAGGFIASHIARRLKSEGHYIIASDWKKNEHMTEDMFCHEFHLVDLRVMDNCMKVTENVDHVFNLAADMGGMGFIQSNHSVIMYNNTMISFNMLEAARINGVKRFFYASSACIYPEFKQLET-N--V---SLKESDAWPAEPQDAYGLEKLATEELCKHYTKDFGIECRIGRFHNIYGPFGTWKGGREKAPAAFCRKALTSVDKFEMWGDGLQTRSFTFIDECVEGVLRLTKSDFREPVNIGSDEMVSMNEMAEIVLSFDDK-KLPIHHIPGPEGVRGRNSDNTLIKEKLGWAPTMKLKDGLRITYMWIKEQIEKEKSKGI-DLT-VYGSSKVVGTQAPVQLGSLRAADGKE-------------------------

>DcGME-1

MG-ST--E-QN-LY------GAYTY-ESLEREPYWPAENLRISITGAGGFIASHIARRLKSEGHYIIASDWKKNEHMPEDMFCHEFHLADLRVMDNCLKVTQNVDHVFNLAADMGGMGFIQSNHSVIMYNNTMISFNMLEAGRINGVKRLFYASSACIYPEFKQLET-N--V---SLKESDAWPAEPQDAYGLEKLATEELCKHYTKDFGIECRIGRFHNIYGPFGTWKGGREKAPAAFCRKALTSTDKFEMWGDGLQTRSFTFIDECVEGVLRLTKSDFREPVNIGSDEMVSMNEMAEIVLSFEDR-KLPIQHIPGPEGVRGRNSDNTLIKEKLGWAPTMKLKDGLRITYFWIKEQIEKEKAKGG-DMS-VYGSSKVVGTQAPVQLGSLRAADGKE-------------------------

>DcGME-2

MG-ST--E-QN-LY------IAYTY-ESLEREPYWPSEKLRISITGAGGFIASHIARRLKSEGHYIIASDWKRNEHMPEEMFCHEFHLVDLRVMDNCLKVSENVDHVFNLAADMGGMGFIQSNHSVIMYNNTMISFNMLEAARINGIKRFFYASSACIYPEFKQLET-N--V---SLKEADAWPAEPQDAYGLEKLATEELCKHYTKDFGIECRIGRFHNIYGPFGTWKGGREKAPAAFCRKALTSTDKFEMWGDGLQTRSFTFIDECVEGVLRLTKSDFREPVNIGSDEMVSMNEMAEIILSFEDK-KLPIQHIPGPEGVRGRNSDNTLIKEKLDWAPTMKLKDGLNITYFWIKEQIEKEKAKGG-DLS-VYGSSKVVGTQAPVQLGSLRAADGKE-------------------------

>EgGME-1

MG-SI--D-GT-DY------GAFTY-ENLEREPYWPSEKLRISITGAGGFIASHIARRLKSEGHYIIASDWKKNEHMTEDMFCHEFHLVDLRVMDNCLKVTKGVDHVFNLAADMGGMGFIQSNHSVIMYNNTMISFNMLEAARINGVKRFFYASSACIYPEFKQLET-N--V---SLKESDAWPAEPQDAYGLEKLATEELCKHYTKDFGIECRVGRFHNIYGPFGTWKGGREKAPAAFCRKTITSTDKFEMWGDGLQTRSFTFIDECVEGVLRLTKSDFREPVNIGSDEMVSMNEMAEIVLSFENK-KLPIHHIPGPEGVRGRNSDNTLIKEKLGWAPTMKLRDGLRITYFWIKEQIEKEKAQGM-DLS-IYGSSKVVGTQAPVQLGSLRAADGKE-------------------------

>EgGME-2

MG-SI--D-RS-SY------GAYTY-ESLEREPYWPSQKLRISITGAGGFIASHIARRLKSEGHYIIASDWKKNEHMTEDMFCNEFHLVDLRVMDNCLKVTQGVDHVFNLAADMGGMGFIQSNHSVIMYNNTMISFNMLEASRINSVKRFFYASSACIYPEFKQLET-N--V---SLKESDAWPAEPQDAYGLEKLATEELCKHYTKDFAIECRIGRFHNIYGPFGTWKGGREKAPAAFCRKALTSTDKFEMWGDGLQTRSFTFIDECVEGVLRLTKSDFREPVNIGSDEMVSMNEMAEMVLSFEDK-KLPIHHIPGPEGVRGRNSENTLIKEKLGWAPTMRLKDGLRITYFWIKEQIEKEKAQGT-DLS-IYGSSKVVGTQAPVQLGSLRAADGKE-------------------------

>EsGME

MT-TT--N-GA-DY------GAYTY-KELERELYWPSEKLRISITGAGGFIASHIARRLKHEGHYVIASDWKKNEHMTEDMFCNEFHLVDLRVMENCLKVTNGVDHVFNLAADMGGMGFIQSNHSVIMYNNTMISFNMIEAARINGIKRFFYASSACIYPEFKQLETTN--V---SLKESDAWPAEPQDAYGLEKLATEELCKHYNKDFGIECRIGRFHNIYGPFGTWKGGREKAPAAFCRKALTSTDRFEMWGDGLQTRSFTFIDECVEGVLRLTKSDFREPVNIGSDEMVSMNEMAEMVLSFEEK-NLPIHHIPGPEGVRGRNSDNNLIKEKLGWAPTMRLKEGLRITYFWIKEQIEKEKAKGS-DVT-LYGSSKVVGTQAPVQLGSLRAADGKE-------------------------

>FvGME

MG-SA--G-ES-GY------GAYTY-EALEREPYWPSEKLRISITGAGGFIASHIARRLKNEGHYIIASDWKKNEHMTEDMFCDEFHLVDLRVMDNCLKVTKDVDHVFNLAADMGGMGFIQSNHSVIMYNNTMISFNMLEAARITGVKRFFYASSACIYPEFKQLET-N--V---SLKEADAWPAEPQDAYGLEKLATEELCKHYTKDFGIECRIGRFHNIYGPFGTWKGGREKAPAAFCRKALTSTDKFEMWGDGLQTRSFTFIDECVEGVLRLTKSDFREPVNIGSDEMVSMNEMAEIVLSFENK-KLPIQHIPGPEGVRGRNSDNTLIKEKLGWAPTMRLKDGLRFTYFWIKEQIEKEKAQGT-DLS-VYGSSKVVGTQAPVQLGSLRAADGKE-------------------------

>GmGME-1

MG-SS--G-TT-DY------GAYTY-QNLEREPYWPSEKLRISITGAGGFIASHIARRLKTEGHYIIASDWKKNEHMTEDMFCHEFHLVDLRVMDNCLTVTKGVDHVFNLAADMGGMGFIQSNHSVIMYNNTMISFNMIEAARINGVKRFFYASSACIYPEFKQLET-N--V---SLKESDAWPAEPQDAYGLEKLATEELCKHYNKDFGIECRIGRFHNIYGPYGTWKGGREKAPAAFCRKTLTSKDRFEMWGDGLQTRSFTFIDECVEGVLRLTKSDFREPVNIGSDEMVSMNEMAEIVLSFEDK-NIPIYHIPGPEGVRGRNSDNTLIKEKLGWAPTMKLKDGLRITYFWIKEQLEKEKAEGV-DLS-GYGSSKVVQTQAPVQLGSLRAADGKE-------------------------

>GmGME-2

MG-SS--G-TT-DY------GAYTY-QNLEREPYWPSEKLRISITGAGGFIASHIARRLKTEGHYIIASDWKKNEHMTEDMFCHEFHLVDLRVMDNCLTVTKGVDHVFNLAADMGGMGFIQSNHSVIMYNNTMISFNMIEAARINGVKRFFYASSACIYPEFKQLET-N--V---SLKESDAWPAEPQDAYGLEKLATEELCKHYNKDFGIECRIGRFHNIYGPYGTWKGGREKAPAAFCRKTLTSKDRFEMWGDGLQTRSFTFIDECVEGVLRLTKSDFREPVNIGSDEMVSMNEMAEIVLSFEDK-NIPIYHIPGPEGVRGRNSDNTLIKEKLGWAPTMKLKDGLRITYFWIKEQLEKEKAEGV-DLS-GYGSSKVVQTQAPVQLGSLRAADGKE-------------------------

>GmGME-3

MG-SA--G-GT-DY------GAYTY-ENLEREPYWPSEKLKISITGAGGFIASHIARRLKTEGHYIIASDWKKNEHMTEDMFCDEFHLVDLRVMNNCLKVTEGVDHVFNLAADMGGMGFIQSNHSVIMYNNTMISFNMIEAARINGIKRFFYASSACIYPEFKQLET-N--V---SLKESDAWPAEPQDAYGLEKLATEELCKHYNKDFGIECRIGRFHNIYGPFGTWKGGREKAPAAFCRKVITSSDRFEMWGDGLQTRSFTFIDECVEGVLRLTKSDFREPVNIGSDEMVSMNEMAEIILGFENK-NIPIHHIPGPEGVRGRNSDNTLIKEKLGWAPTMRLKDGLRITYFWIKEQIEKEKAQGI-DIS-VYGSSKVVQTQAPVQLGSLRAADGKE-------------------------

>GmGME-4

MG-SA--G-RT-DY------GAYTY-ENLEREPYWPSEKLKISITGAGGFIASHIARRLKTEGHYVIASDWKKNEHMTENMFCDEFHLVDLRVMDNCLKVTKGVDHVFNLAADMGGMGFIQSNHSVIMYNNTMISFNMIEAARINGIKRFFYASSACIYPEFKQLET-N--V---SLKESDAWPAEPQDAYGLEKLATEELCKHYNKDFGIECRIGRFHNIYGPFGTWKGGREKAPAAFCRKVITSTDRFEMWGDGLQTRSFTFIDECVEGVLRLTKSDFREPVNIGSDEMVSMNEMAEIILGFENK-NIPIHHIPGPEGVRGRNSDNTLIKEKLGWAPTMRLKDGLRITYFWIKEQIEKEKAQGI-DIS-VYGSSKVVQTQAPVQLGSLRAADGKE-------------------------

>GmGME-5

MG-IS--G-TT-DY------GSFTY-QNLEREPYWPSEKLRISITGAGGFIASHIARRLKTEGHYIIASDWKKNEHMTEGMFCHEFHLVDLRVMDNCLTVTKGVDHVFNLAADMGGMGFIQSNHSVIMYNNTMISFNMIEAARINGVKRFFYASSACIYPEFKQLET-N--V---SLKESDAWPAEPQDAYGLEKLATEELCKHYNKDFGIECRIGRFHNIYGPYGTWKGGREKAPAAFCRKTLTSKDRFEMWGDGLQTRSFTFIDECVEGVLRLTKSDFREPVNIGSDEMVSMNEMAEIVLSFEDK-NIPIYHIPGPEGVRGRNSDNTLIKEKLGWAPTMKLKDGLRITYFWIKEQLEKEKAEGV-DLS-GYGSSKVVQTQAPVQLGSLRAADGKE-------------------------

>GrGME-1

MG-ST--D-GT-SY------GAYTY-DALEREPYWPSEKLRISITGAGGFIASHIARRLKSEGHYIIASDWKKNEHMTEDMFCHEFHLVDLRVMENCLKVTNGVDHVFNLAADMGGMGFIQSNHSVIMYNNTMISFNMLEAARISGVKRFFYASSACIYPEFKQLET-N--V---SLKESDAWPAEPQDAYGLEKLATEELCKHYTKDFGIECRIGRFHNIYGPFGTWKGGREKAPAAFCRKAITSIDKFEMWGDGLQTRSFTFIDECVEGVLRLTKSDFREPVNIGSDEMVSMNEMAEIVLSFEDK-KLPIHHIPGPEGVRGRNSDNTLIKEKLGWAPTMRLKDGLRITYFWIKEQIEKEKVQGI-DLS-VYGSSKVVGTQAPVQLGSLRAADGKE-------------------------

>GrGME-2

MG-ST--D-GT-SY------GAYTY-DALEREPYWPSQKLRISITGAGGFIASHIARRLKSEGHYIIASDWKKNEHMTEDMFCNEFHLVDLRVMENCLKVSKGVDHVFNLAADMGGMGFIQSNHSVIMYNNTMISFNMLEAARISGVKRFFYASSACIYPEFKQLDT-N--V---SLKESDAWPAEPQDAYGLEKLATEELCKHYTKDFGIECRIGRFHNIYGPFGTWKGGREKAPAAFCRKAITSIDKFEMWGDGLQTRSFTFIDECVEGVLRLTKSDFREPVNIGSDEMVSMNEMAEIVLSFEDK-NLPIHHIPGPEGVRGRNSDNTLIKEKLGWAPTMRLKDGLRITYFWIKEQIEKEKAQGI-ELS-VYGSSKVVGTQAPVQLGSLRAADGKE-------------------------

>KfGME

MG-TT--D-GG-KY------GAFTY-ENLEREPYWPSEKLRISITGAGGFIASHIARRLKSEGHYIIASDWKKNEHMTEDMFCHEFHLADLRVMENCLKVTSGVDHVFNLAADMGGMGFIQSNHSVIMYNNTMISFNMMEAARINGVKRFFYASSACIYPEFKQLET-N--V---SLKESDAWPAEPQDAYGLEKLATEELCKHYNKDFGIECRIGRFHNIYGPFGTWKGGREKAPAAFCRKALTATDKFEMWGDGLQTRSFTFIDECVEGVLRLTKSDFREPVNIGSDEMVSMNEMAEIVLSFEDR-KLPIEHIPGPEGVRGRNSDNTLIKEKLGWAPSMKLKDGLRITYFWIKEQIEKEKAEGK-DLS-VYGSSKVVGTQAPVQLGSLRAADGKE-------------------------

>KlGME-1

MG-TT--D-GG-KY------GAFTY-ENLEREPYWPSEKLRISITGAGGFIASHIARRLKSEGHYIIASDWKKNEHMTEDMFCHEFHLADLRVMENCLKVTSGVDHVFNLAADMGGMGFIQSNHSVIMYNNTMISFNMMEAARINGVKRFFYASSACIYPEFKQLET-N--V---SLKESDAWPAEPQDAYGLEKLATEELCKHYNKDFGIECRIGRFHNIYGPFGTWKGGREKAPAAFCRKALTATDKFEMWGDGLQTRSFTFIDECVEGVLRLTKSDFREPVNIGSDEMVSMNEMAEIVLSFEDR-KLPIEHIPGPEGVRGRNSDNTLIKEKLGWAPSMKLKDGLRITYFWIKEQIEKEKAEGK-DLS-VYGSSKVVGTQAPVQLGSLRAADGKE-------------------------

>KlGME-2

MG-TT--D-GG-KY------GAFTY-ENLEREPYWPSEKLRISITGAGGFIASHIARRLKSEGHYIIASDWKKNEHMTEDMFCHEFHLADLRVMENCLKVTSGVDHVFNLAADMGGMGFIQSNHSVIMYNNTMISFNMMEAARINGVKRFFYASSACIYPEFKQLET-N--V---SLKESDAWPAEPQDAYGLEKLATEELCKHYNKDFGIECRIGRFHNIYGPFGTWKGGREKAPAAFCRKALTATDKFEMWGDGLQTRSFTFIDECVEGVLRLTKSDFREPVNIGSDEMVSMNEMAEIVLSFEDR-KLPIEHIPGPEGVRGRNSDNTLIKEKLGWAPSMKLKDGLRITYFWIKEQIEKEKAEGK-DLS-VYGSSKVVGTQAPVQLGSLRAADGKE-------------------------

>LuGME-1

MG-SN--D-GS-NY------GAYTY-EELEREAYWPSEKLRISITGAGGFIASHIARRLKSEGHYIIASDWKKNEHMPEDMFCHEFHLVDLRVMDNCLKVTQAVDHVFNLAADMGGMGFIQSNHSVIMYNNTMISFNMLEASRINGVKRFFYASSACIYPEFKQLET-N--V---SLKEADAWPAEPQDAYGLEKLATEELCKHYTKDFGIECRVGRFHNIYGPFGTWKGGREKAPAAFCRKALTSADKFEMWGDGLQTRSFTFIDECVEGVLRLTKSDFREPVNIGSDEMVSMNEMAEIVLSFEER-KLPIQHIPGPEGVRGRNSDNTLIKEKLGWAPTMMLKDGLKITYFWIKEQIEKEKAKGV-DLA-VYGSSKVVGTQAPVQLGSLRAADGKE-------------------------

>LuGME-2

MG-SN--D-EA-SY------GSYTY-EELEREPYWPSEKLRISITGAGGFIASHIARRLKSEGHYIIASDWKKNEHMTEDMFCHEFHLVDLRVMDNCLKVTNGVDHVFNLAADMGGMGFIQSNHSVIMYNNTMISFNMLEASRINGVKRFFYASSACIYPEFKQLET-N--V---SLKEADAWPAEPQDAYGLEKLATEELCKHYTKDFGIECRVGRFHNIYGPFGTWKGGREKAPAAFCRKALTSADKFEMWGDGLQTRSFTFIDECVEGVLRLTKSDFREPVNIGSDEMVSMNEMAEIVLSFEDR-KLPIHHIPGPEGVRGRNSDNTLIKEKLGWAPTMKLKDGLRFTYFWIKEQIEKEKAKGV-DLA-VYGSSKVVGTQAPVQLGSLRAADGKE-------------------------

>LuGME-3

MG-SN--D-EA-SY------GSYTY-EELQRELYWPSEKLRISITGAGGFIASHIARRLKSEGHYIIASDWKKNEHMTEDMFCHEFHLVDLRVMDNCLKVTNGVDHVFNLAADMGGMGFIQSNHSVIMYNNTMISFNMLEASRINGVKRFFYASSACIYPEFKQLET-N--V---SLKEADAWPAEPQDAYGLEKLATEELCKHYTKDFGIECRVGRFHNIYGPFGTWKGGREKAPAAFCRKALTSAEKFEMWGDGLQTRSFTFIDECVEGVLRLTKSDFREPVNIGSDEMVSMNEMAEIVLSFEDR-KHPIHHIPGPEGVRGRNSDNTLIKEKLGWAPTMKLKDGLRFTYFWIKEQIEKEKAKGV-DLA-VYGSSKVVGTQAPVQLGSLRAADGKE-------------------------

>LuGME-4

MG-SN--D-GS-SY------GAYTY-EELEREAYWPSEKLRISITGAGGFIASHIARRLKSEGHYIIASDWKKNEHMPEDMFCHEFHLVDLRVMDNCLKVTQAVDHVFNLAADMGGMGFIQSNHSVIMYNNTMISFNMLEASRINGVKRFFYASSACIYPEFKQLET-N--V---SLKEADAWPAEPQDAYGLEKLATEELCKHYTKDFGIECRVGRFHNIYGPFGTWKGGREKAPAAFCRKALTSADKFEMWGDGLQTRSFTFIDECVEGVLRLTKSDFREPVNIGSDEMVSMNEMAEIVLSFEDR-KLPIQHIPGPEGVRGRNSDNTLIKEKLGWAPTMRLKDGLRITYFWIKEQIEKEKAKGV-DLA-VYGSSKVVGTQAPVQLGSLRAADGKE-------------------------

>MeGME-1

MG-ST--D-GT-SY------GAFTY-ENLEREPYWSSEKLRISITGAGGFIASHIARRLKSEGHYIIASDWKKNEHMTEDMFCHEFHLVDLRVMDNCLKVTKDVDHVFNLAADMGGMGFIQSNHSVIMYNNTMISFNMLEAARINGVKRFFYASSACIYPEFKQLDT-N--V---SLKESDAWPAEPQDAYGLEKLATEELCKHYTKDFGIECRIGRFHNIYGPFGTWKGGREKAPAAFCRKAITSTDKFEMWGDGLQTRSFTFIDECVEGVLRLTKSDFREPVNIGSDEMVSMNEMAEIVLSFENK-KLPIHHIPGPEGVRGRNSDNTLIKEKLGWAPTMKLKDGLRITYFWIKEQIEKEKAQGI-DLS-IYGSSKVVGTQAPVQLGSLRAADGKE-------------------------

>MeGME-2

MG-ST--E-GT-NY------GAFTY-ENLEREPYWPSEKLRISITGAGGFIASHIARRLKSEGHYIIASDWKKNEHMTEDMFCHEFHLVDLRVMDNCLKVTKDVDHVFNLAADMGGMGFIQSNHSVIMYNNTMISFNMLEAARISGVKRFFYASSACIYPEFKQLDT-N--V---SLKESDAWPAEPQDAYGLEKLATEELCKHYTKDFGIECRIGRFHNIYGPFGTWKGGREKAPAAFCRKAITSIDKFEMWGDGLQTRSFTFIDECVEGVLRLTKSDFREPVNIGSDEMVSMNEMADIVLSFENK-KLPIHHIPGPEGVRGRNSDNTLIKEKLGWAPTMKLKDGLRITYFWIKEQIEKEKAQGI-DLS-IYGSSKVVGTQAPVQLGSLRAADGKE-------------------------

>MgGME-1

MG-ST----GENLY------KAYTY-ENLETEPYWPSEKLRISITGAGGFIASHIARRLKSEGHYIIASDWKKNEHMPEDMFCHEFHLVDLRVMDNCLKVTEGVDHVFNLAADMGGMGFIQSNHSVIMYNNTMISFNMIEAGRINGVKRFFYASSACIYPEFKQLET-N--V---SLKESDAWPAEPQDAYGLEKLATEELCKHYNKDFGIECRIGRFHNIYGPFGTWKGGREKAPAAFCRKTLTSTDKFEMWGDGLQTRSFTFIDECVEGVLRLTKSDFREPVNIGSDEEVSMNGMAEIISSFEDK-KLPIHHIPGPEGVRGRNSDNTLIKEKLGWAPSMKLKDGLRITYFWIKEQLEKEKTLGV-DLS-NYGSSKVVGTQAPVQLGSLRAADGKE-------------------------

>MgGME-2

MA-SS--D-ET-NY------GSYTY-ETLEREPYWPSEKLRISITGAGGFIASHIARRLKSEGHYIIASDWKKNEHMTEDMFCNEFHLVDLRVMDNCLKVTDGVDHVFNLAADMGGMGFIQSNHSVIMYNNTMISFNMIEAARINGVKRFFYASSACIYPEFKQLET-N--V---SLKESDAWPAEPQDAYGLEKLATEELCKHYNKDFGIECRIGRFHNIYGPFGTWKGGREKAPAAFCRKSLTSTDKFEMWGDGLQTRSFTFIDECVEGVLRLTKSDFREPVNIGSDEMVSMNEMAEIVLGFENK-KLPIYHIPGPEGVRGRNSDNTLIKEKLGWAPSMKLKDGLRITYFWIKEQLEKEKAHGT-DLS-AYGSSKVVGTQAPVQLGSLRAADGKE-------------------------

>MipGME

MA-AA-------GYDNLQLRGKYGT------EKYWPEKKLKICVTGAGGFIASHLAKRLKEEGHHIVGCDWKRNEHMPEEMFCDEFILADLRLFENCQKVLKGCDHCFNLAADMGGMGFIQSNHSVIFYNNVMISFNVMEACRVEGVTRVFYASSACIYPEGAQLTT-EARLSA-GLKEADAWPAQPQDAYGLEKLASEEVYKHYQSDFGIQTRIARFHNIYGPFGTWKGGREKAPAAFCRKAATATTEVEMWGDGLQTRSFTYIDDCVEGIVRLTKSDFCEPVNLGSDEM-------ALALGFAGKPDMPIKHIPGPEGVRGRNSNNDLIKEKLGYAPSVPLAEGLKVTFEWINEKIEEEVKGGA-NAEEAFSKSTICGTMAPTELGALRAADGQEGLKAK--------------------

>MpGME

MA-SNGVN-GT-TN------GLYEA-TNLDKELYWPEKKLRISVTGAGGFIASHIARRLKSEGHYVIASDWKRNEHMTEDMFCDEFHLVDLRVMENCLKVTQDVEHVFNLAADMGGMGFIQSNHSVIMYNNTMISFNMLEAARINGVKRFFYASSACIYPEYKQLDV-DC-V---SLKEADAWPAEPQDAYGLEKLATEELCKHYTKDFGMQCRIGRFHNIYGPYGTWKGGREKAPAAFCRKAITSTEKFEMWGDGKQTRSFTFIDECVEGVLRLTKSEYSEPVNIGSDEMVSMNEMAEIVLSFEGK-DLPIEHIPGPEGVRGRNSDNTLIKEKLGWSPTMRLKDGLRITYYWILEQVEKEKAKGV-TMD-TYATSKVVGTQAPVALGSLRAADGKE-------------------------

>MsrGME-1

MS-AA-------GY---ELRGVYGT------EPYWPEKKLKICVTGAGGFIASHLAKRLKEEGHYVVGCDWKRNEHMPEEMFCDEFILADLRLFENCQKVLKGCDHCFNLAADMGGMGFIQSNHSVIFYNNVMISFNVMEACRVEGVTRVFYASSACIYPEGAQLTT-E--LSA-GLKESDAWPAQPQDAYGLEKLASEEVYKHYQSDFGIQTRIARFHNIYGPFGTWKGGREKAPAAFCRKAATATTEVEMWGDGKQTRSFTYIDDCVEGIIRLTKSDFAEPVNLGSDEMVSMNEMQALALGFAGKQDMPIKHIPGPEGVRGRNSNNDLIKEKLGYAPSVKLADGLKVTYEWIEGKIKEEVAAGA-NAEEAFSKSTICGTMAPTELGALRAADGQENLA-K--------------------

>MsrGME-2

MS-AA-------GF---KLRGIYGT------EPYWPKKKLKICVTGAGGFIASHLAQRLKEEGHFVVGCDWKRNEHMPEEMFCDEFILADLRLFENCQNVLKGCDHCFNLAADMGGMGFIQSNHSVIFYNNIMISFNMMEACRVEGITRVFYASSACIYPEGAQLTT-D--LSA-GLKEADAWPAQPQDAYGLEKLASEEVYKHYQSDFGIQTRIARFHNIYGPFGTWKGGREKAPAAFCRKAATATTEVEMWGDGKQTRSFTYIDDCIEGILRLTKSDFAEPVNLGSDEMVSMNEMQALALGFAGKPNMPVKHIPGPEGVRGRNSNNDLIMEKLGYAPSVKLADGLKVTYEWIEAKIKEEVADGA-DAEAAFSKSTICGTMAPTELGALRAADGAENLK----------------------

>MtGME-1

MG-SS--GINN-DY------GAFTY-QNLEREPYWPTEKLRISITGAGGFIASHIARRLKTEGHYIIASDWKKNEHMTEDMFCHEFHLVDLRVMDNCLKVTKDVDHVFNLAADMGGMGFIQSNHSVIMYNNTMISFNMIEAARINGVKRFFYASSACIYPEFKQLET-N--V---SLKEADAWPAEPQDAYGLEKLATEELCKHYNKDFGIECRIGRFHNIYGPFGTWKGGREKAPAAFCRKTLTSTDKFEMWGDGLQTRSFTFIDECVEGVLRLTKSDFREPVNIGSDEMVSMNEMAEIVLSFENK-SIPIQHIPGPEGVRGRNSDNTLIKEKLGWAPTMKLKDGLRITYFWIKEQLEKEKAGGV-DVT-SYGSSKVVSTQAPVQLGSLRAADGNE-------------------------

>MtGME-2

MG-ST--E-KT-NY------GEYTY-ENLEREPYWPSEKLKISITGAGGFIASHLARRLKKEGHYIIASDWKKNEHMTEDMFCDEFHLVDLRVMDNCLTVTKGVDHVFNLAADMGGMGFIQSNHSVIMYNNTMISFNMIEAARINGIKRFFYASSACIYPEFKQLETTN--V---SLKESDAWPAEPQDAYGLEKLATEEICKHYNKDFGIECRIGRFHNIYGPFGTWKGGREKAPAAFCRKAITSTDKFEMWGDGLQTRSFTFIDECVEGVLRLTKSDFREPVNIGSDEMVSMNEMAEIVLGFEDK-KTPIHHIPGPEGVRGRNSDNTLIKEKLGWAPTMKLKDGLRITYVWIKEQLEKEKAQGL-DTS-GYGSSKVVSTQAPVQLGSLRAADGKEGSS----------------------

>OlGME

MA-AA-------GY---ELRGIYGT------EEYWPEKKLKICVTGAGGFIGSHLAKRLKEEGHHVVACDWKRNEHMEEAMFCDEFILADLRLYENCKKVLEGCDHCFNLAADMGGMGFIQSNHSVIFYNNVMISFNMMEAMRVQGVTRCFYASSACIYPEGTQLST-E--MQD-GLKEASAWPAQPQDAYGLEKLASEEVYKHYQQDFGIQTRIGRFHNIYGPYGTWKGGREKAPAAFCRKAATAESEVEMWGDGKQTRSFTYIDDCVEGILRLTKSDFAEPVNIGSDEMISMNDMQAMTLKFAGK-DLPIKHIPGPEGVRGRNSNNELIKEKLGWAPSVKLADGLKVTFEWISSKIAEEKAKGV-DTAAAFGKSTICGTQAPTELGQLRAADGDEKL-----------------------

>OsGME-1

MG-SSEKN-GT-AY------GEYTY-AELEREQYWPSEKLRISITGAGGFIGSHIARRLKSEGHYIIASDWKKNEHMTEDMFCHEFHLVDLRVMDNCLKVTNGVDHVFNLAADMGGMGFIQSNHSVIMYNNTMISFNMLEAARINGVKRFFYASSACIYPEFKQLET-N--V---SLKESDAWPAEPQDAYGLEKLATEELCKHYTKDFGIECRVGRFHNIYGPFGTWKGGREKAPAAFCRKAQTSTDRFEMWGDGLQTRSFTFIDECVEGVLRLTKSDFREPVNIGSDEMVSMNEMAEIILSFEDR-ELPIHHIPGPEGVRGRNSDNTLIKEKLGWAPTMKLKDGLRFTYFWIKEQIEKEKTQGV-DIA-GYGSSKVVSTQAPVQLGSLRAADGKE-------------------------

>OsGME-2

MA----------LN------EEYTY-VELEKEPYWPFEKLRISITGAGGFIASHIARRLKSEGHYIIASDWKKNEHMTEDMFCHEFHLVDLRVMDNCLKVTTGVDHVFNLAADMGGMGFIQSNHSVIMYNNTMISFNMLEAARINGVKRFFYASSACIYPEFKQLDT-V--V---SLKESDAWPAEPQDAYGLEKLATEELCKHYTKDFGIECRVGRFHNIYGPFGTWKGGREKAPAAFCRKALTSTDRFEMWGDGLQTRSFTFIDECVEGVLRLTKSDFREPVNIGSDEMVSMNEMAEIVLSFENK-QLPIHHIPGPEGVRGRNSDNTLIKEKLGWAPTMRLKDGLRITYFWIKEQLEKEKAEGV-DLS-AYGSSKVVQTQAPVQLGSLRAADGKE-------------------------

>OtGME

MG-SSEKT-VS-AY------GEYTY-AELEREPYWPSEKLRISITGAGGFIGSHIARRLKSEGHYIIASDWKKNEHMTEDMFCHEFHLVDLRVMDNCLKVTNGVDHVFNLAADMGGMGFIQSNHSVIMYNNTMISFNMLEAARINGVKRFFYASSACIYPEFKQLDT-N--V---SLKESDAWPAEPQDAYGLEKLATEELCKHYTKDFGIECRVGRFHNIYGPFGTWKGGREKAPAAFCRKAQTSTDRFEMWGDGLQTRSFTFIDECVEGVLRLTKSDFREPVNIGSDEMVSMNEMAEIVLSFEDR-KLPIHHIPGPEGVRGRNSDNTLIKEKLGWAPTMKLKDGLRFTYFWIKEQIEKEKTQGV-DIA-AYGSSKVVSTQAPVQLGSLRAADGKEGL-----------------------

>PhGME-1

MG-SSEKT-VT-AY------GEYTY-AELEREPYWPSEKLRISITGAGGFIGSHIARRLKSEGHYIIASDWKKNEHMTEDMFCHEFHLVDLRVMDNCLKVTQGVDHVFNLAADMGGMGFIQSNHSVIMYNNTMISFNMLEAARINGVKRFFYASSACIYPEFKQLDT-N--V---SLKESDAWPAEPQDAYGLEKLATEELCKHYTKDFGIECRIGRFHNIYGPFGTWKGGREKAPAAFCRKAQTSTERFEMWGDGLQTRSFTFIDECVEGVLRLTKSDFREPVNIGSDEMVSMNEMAEIVLSFEDR-KLPIHHIPGPEGVRGRNSDNTLIKEKLGWAPTMRLKDGLRFTYFWIKEQIEKEKTQGI-DVA-AYGSSKVVSTQAPVQLGSLRAADGKEGL-----------------------

>PhGME-2

MG-SSEKT-VT-AY------GEYTY-AELEREPYWPSEKLRISITGAGGFIGSHIARRLKSEGHYIIASDWKKNEHMTEDMFCHEFHLVDLRVMDNCLKVTQGVDHVFNLAADMGGMGFIQSNHSVIMYNNTMISFNMLEAARINGVKRFFYASSACIYPEFKQLDT-N--V---SLKESDAWPAEPQDAYGLEKLATEELCKHYTKDFGIECRVGRFHNIYGPFGTWKGGREKAPAAFCRKAQTSTERFEMWGDGLQTRSFTFIDECVEGVLRLTKSDFREPVNIGSDEMVSMNEMAEIVLSFEDR-KLPVHHIPGPEGVRGRNSDNTLIKEKLGWAPTMKLKDGLRFTYFWIKEQIEKEKTQGV-DVA-AYGSSKVVSTQAPVQLGSLRAADGKEGL-----------------------

>PhGME-3

MA----------LN------KEYTY-AELEKEPYWPFEKLRISITGAGGFIASHIARRLKSEGHYIIASDWKKNEHMTEEMFCHEFHLVDLRVMDNCLKVTTGVDHVFNLAADMGGMGFIQSNHSVIMYNNTMISFNMLEAARINGVKRFFYASSACIYPEFKQLET-V--V---SLKESDAWPAEPQDAYGLEKLATEELCKHYTKDFGIECRIGRFHNIYGPFGTWKGGREKAPAAFCRKALTSTGRFEMWGDGLQTRSFTFIDECVEGVLRLTKSDFREPVNIGSDEMVSMNEMAEIVLSFENK-QLPIHHIPGPEGVRGRNSDNTLIKEKLGWAPTMKLKDGLRITYFWIKEQLEKEKAEGM-DLS-VYGSSKVVQTQAPVQLGSLRAADGKE-------------------------

>PhpGME-1

MA-SN--G----SF------GDYTA-TNLDREEYWPSQKLRISITGAGGFIASHIARRLKSEGHYIIASDWKKNEHMSEDMFCDEFHLVDLRVMDNCMKVTKGAHHVFNLAADMGGMGFIQSNHAVIMYNNTMISFNMLEAARINGVTRFFYASSACIYPEFKQLET-D--VS--SLKESDAWPALPQDAYGLEKLATEELCKHYTKDFGMECRIGRFHNIYGPYGTWKGGREKAPAAFCRKALTATEHFEMWGDGKQTRSFTFIDECVEGVLRLTKSDFQEPVNIGSDEMVSMNEMAEIVLSFDNK-QLPIKHIPGPEGVRGRNSDNTLIKEKLGWAPSMRLRDGLAITYKWIKEQIEKEKESGA-DLASKYGSSKVVGTQAPVQLGSLRAADGKE-------------------------

>PhpGME-2

MA-SY--G----RF------GDYTA-TNLDREAYRPAEKLRISITGAGGFIASHIARRLKSEGHYIIASDWKKNEHMSEDMFCDEFHLVDLRVMDNCLKVTKGANHVFNLAADMGGMGFIQSNHAVIMYNNTMISFNMLEASRINGVSRFFYASSACIYPEFKQLET-D--VS--SLKESDAWPALPQDAYGLEKLATEELCRHYTKDFGMECRIGRFHNIYGPYGTWKGGREKAPAAFCRKALTATEYFEMWGDGKQTRSFTFIDECVEGVLRLTKSDFQEPVNIGSDEMLSMNEMAEIVLSFDNK-KLPIKHIPGPEGVRGRNSDNTLIKEKLGWAPSMRLRDGLAITYKWIKEQIEKEKESGA-DLASKYGSSKVVGTQAPVQLGSLRAADGKE-------------------------

>PhpGME-3

MAESN----GT-SF------GNYTA-TNLDRELYWPNQKLRISITGAGGFIASHIARRLKSEGHYIIASDWKKNEHMSEDAFCDEFHLVDLRVMDNCLKVTQGAHHVFNLAADMGGMGFIQSNHAVIMYNNTMISFNMLEAARINGVSRFFYASSACIYPEFKQLET-D--VS--SLKESDAWPALPQDAYGLEKLATEELCKHYTKDFGMECRIGRFHNIYGPYGTWKGGREKAPAAFCRKALTATEYFEMWGDGKQTRSFTFIDECVEGVLRLTKSDFQEPVNIGSDEMVSMNEMAEIVLSFDNK-KLPIKHIPGPEGVRGRNSDNTLIKEKLGWAPSMRLMDGLAITYKWIKEQIDKEKELGT-ELASKYGTSMVVGTQAPVQLGSLRAADGKE-------------------------

>PhvGME-1

MG-SS--G-AN-DY------GAYTY-QNLEREPYWPSEKLRISITGAGGFIASHIARRLKTEGHYIIASDWKKNEHMTEDMFCHEFHLVDLRVMDNCLAVTKGVDHVFNLAADMGGMGFIQSNHSVIMYNNTMISFNMIEAARINGVKRFFYASSACIYPEFKQLET-N--V---SLKEADAWPAEPQDAYGLEKLATEELCKHYNKDFGIECRIGRFHNIYGPYGTWKGGREKAPAAFCRKTLTSKDQFEMWGDGLQTRSFTFIDECVEGVLRLTKSDFREPVNIGSDEMVSMNEMAEIVLSFEDK-TIPIYHIPGPEGVRGRNSDNTLIKEKLGWAPTMKLKDGLRITYFWIKEQLEKEKAAGV-DLS-VYGSSKVVQTQAPVQLGSLRAADGKE-------------------------

>PhvGME-2

MG-SA--G-KT-DY------GEYTY-ENLEREPYWPSEKLKISITGAGGFIASHIARRLKREGHYIIASDWKKNEHMTEDMFCDEFHLVDLRVMDNCLKVTKGVDHVFNLAADMGGMGFIQSNHSVIMYNNTMISFNMIEAARINGIKRFFYASSACIYPEFKQLET-N--V---SLKESDAWPAEPQDAYGLEKLATEELCKHYNKDFGIECRIGRFHNIYGPFGTWKGGREKAPAAFCRKVITSTDRFEMWGDGLQTRSFTFIDECVEGVLRLTKSDFREPVNIGSDEMVSMNEMAQIILGFESK-NIPIHHIPGPEGVRGRNSENTLIKEKLGWAPTMKLKDGLRITYFWIKEQIEKEKTQGI-DIS-VYGSSKVVQTQAPVQLGSLRAADGKE-------------------------

>PpGME-1

MG-TT--G-GS-KY------GEYTY-ENLEREQYWPSEKLRVSITGAGGFIASHIARRLKSEGHYIIASDWKKNEHMTEDMFCNEFHLVDLRVMDNCLKVTSGVDHVFNLAADMGGMGFIQSNHSVIMYNNTMISFNMLEAARINGAKRLFYASSACIYPEFKQLDTSN--V---SLKESDAWPAEPQDAYGLEKLMTEELCKHYNKDFGIECRIGRFHNIYGPFGTWKGGREKAPAAFCRKTLTSTDKFEMWGDGLQTRSFTFIDECVEGVLRLTKSDFREPVNIGSDEMVSMNEMAEIVLSFENQ-KLPIHHIPGPEGVRGRNSDNTLIKEKLGWAPTMKLKDGLRITYFWIKEQLAKEKAQGM-NLL-GYGSSKVVGTQAPVQLGSLRAADGKELL-----------------------

>PpGME-2

MG-ST--G-GH-DY------GAYTY-ENLEREPYWPSEKLRISITGAGGFIASHIARRLKNEGHYIIASDWKKNEHMTEDMFCHEFHLVDLRVMDNCLKVTKNVDHVFNLAADMGGMGFIQSNHSVIFYNNTMISFNMVEAARINGVKRFFYASSACIYPEFKQLET-N--V---SLKESDAWPAEPQDAYGLEKLATEELCKHYTKDFGIECRIGRFHNIYGPFGTWKGGREKAPAAFCRKTLTSTDKFEMWGDGLQTRSFTFIDECVEGVLRLTKSDFREPVNIGSDEMVSMNEMAEIVLSFEDK-KLPIQHIPGPEGVRGRNSDNTLIKEKLGWAPTMRLKDGLRITYFWIKEQIEKEKAQGT-DLS-NYGSSKVVGTQAPVQLGSLRAADGKE-------------------------

>PsGME-1

MG-STGAD-GV-AY------GAYTY-EDLEREPYWPSEKVIISITGAGGFIASHIARRLKSEGHYIIASDWKKNEHMTEDMFCNEFHLVDLRVMENCLAVTKGVDHVFNLAADMGGMGFIQSNHSVIMYNNTMISFNMIEAARINGVKRFFYASSACIYPEFKQLET-N--V---SLKESDAWPAEPQDAYGLEKLATEELCKHYNKDFGIECRIGRFHNIYGPFGTWKGGREKAPAAFCRKTITSTDRFEMWGDGKQTRSFTFIDECVEGVLRLTKSDFREPVNIGSDEMVSMNEMAEMVLSFENK-KLPIHHIPGPEGVRGRNSDNTLIKEKLGWAPTMRLKDGLRITYFWIKEQIEKEKVQGI-DLS-IYGSSKVVGTQAPVQLGSLRAADGKE-------------------------

>PsGME-2

MG-STRAD-GV-AY------GAYTY-EDLEREPYWPSEKVIISITGAGGFIASHIARRLKSEGHYIIASDWKKNEHMTEDMFCNEFHLVDLRVMENCLAVTKGVDHVFNLAADMGGMGFIQSNHSVIMYNNTMISFNMIEAARINGVKRFFYASSACIYPEFKQLET-N--V---SLKESDAWPAEPQDAYGLEKLATEELCKHYNKDFGIECRIGRFHNIYGPFGTWKGGREKAPAAFCRKTITSTDRFEMWGDGKQTRSFTFIDECVEGVLRLTKSDFREPVNIGSDEMVSMNEMAEMVLSFENK-KLPIHHIPGPEGVRGRNSDNTLIKEKLGWAPTMRLKDGLRITYFWIKEQIEKEKVQGI-DLS-IYGSSKVVGTQAPVQLGSLRAADGKE-------------------------

>PsGME-3

MG-SIGAD-GV-TY------GEYTY-ANLDRELYWPSEKLKISITGAGGFIASHIARRLKSEGHYIIASDWKKNEHMTEDMFCNEFHLVDLRVMDNCLAVTKGVDHVFNLAADMGGMGFIQSNHSVIMYNNTMISFNMLEAARINGVKRFFYASSACIYPEFKQLET-N--V---SLKESDAWPAEPQDAYGLEKLATEELCKHYTKDFGIECRIGRFHNIYGPFGTWKGGREKAPAAFCRKTITSTDRFEMWGDGEQTRSFTFIDECVEGVLRLTKSDFREPVNIGSDEMVSMNEMAEMVSSFENK-KLPIHHIPGPEGVRGRNSENTLIKEKLGWAPTMKLKDGLRITYFWIKKQIEKEKAQGI-DLS-IYGSSKVVGTQAPVQLGSLRAADGKE-------------------------

>PtGME-1

MG-SA--D-GS--Y------GAYTY-EALEREPYWPSENLKISITGAGGFIASHIARRLKSEGHYIIASDWKKNEHMTEDMFCHEFHLVDLRVMDNCLKVTKGVDHVFNLAADMGGMGFIQSNHSVIMYNNTMISFNMLEASRINGVKRLFYASSACIYPEFKQLET-N--V---SLKESDAWPAEPQDAYGLEKLATEELCKHYTKDFGIECRIGRFHNIYGPFGTWKGGREKAPAAFCRKTMTSIDKFEMWGDGLQTRSFTFIDECVEGVLRLTKSDFREPVNIGSDEMVSMNEMAEIVLSFENK-NLPIHHIPGPEGVRGRNSDNTLIKEKLGWAPTMKLKDGLRITYFWIKEQIEKEKSQGM-DLS-IYGSSKVVGTQAPVQLGSLRAADGKE-------------------------

>PtGME-2

MG-TA--D-GS--Y------GSYTY-EALEREPYWPSEKLRISITGAGGFIASHIARRLKAEGHYIIASDWKKNEHMTEDMFCHEFHLVDLRVMDNCLKVTKDVDHVFNLAADMGGMGFIQSNHSVIMYNNTMISFNMLEASRINGVKRLFYASSACIYPEFKQLET-N--V---SLKESDAWPAEPQDAYGLEKLATEELCKHYTKDFGIECRIGRFHNIYGPFGTWKGGREKAPAAFCRKAITSIDKFEMWGDGLQTRSFTFIDECVEGVLRLTKSDFREPVNIGSDEMVSMNEMAEIVLSFENK-NLPIHHIPGPEGVRGRNSDNTLIKEKLGWAPTMRLKDGLRITYFWIKEQIEKEKSKGI-DLS-IYGSSKVVGTQAPVQLGSLRAADGKE-------------------------

>PtGME-3

MG-SV--D-GS--Y------GAYTY-EALEREPYWPSENLKISITGAGGFIASHIARRLKSEGHYIIASDWKKNEHMTEDMFCHEFHLVDLRVMDNCLKVTKGVDHVFNLAADMGGMGFIQSNHSVIMYNNTMISFNMLEASRINGVKRLFYASSACIYPEFKQLET-N--V---SLKESDAWPAEPQDAYGLEKLATEELCKHYTKDFGIECRIGRFHNIYGPFGTWKGGREKAPAAFCRKTMTSIDKFEMWGDGLQTRSFTFIDECVEGVLRLTKSDFREPVNIGSDEMVSMNEMAEIVLSFENK-NLPIHHIPGPEGVRGRNSDNTLIKEKLGWAPTMKLKDGLRFTYFWIKEQIEKEKSQGM-DLS-IYGSSKVVGTQAPVQLGSLRAADGKE-------------------------

>PvGME-1

MG-GSEKT-VT-AY------GEYTY-AELEREPYWPSEKLRISITGAGGFIGSHIARRLKSEGHYIIASDWKKNEHMTEDMFCHEFHLVDLRVMDNCLKVTQGVDHVFNLAADMGGMGFIQSNHSVIMYNNTMISFNMLEAARINGVKRFFYASSACIYPEFKQLDT-N--V---SLKESDAWPAEPQDAYGLEKLATEELCKHYTKDFGIECRIGRFHNIYGPFGTWKGGREKAPAAFCRKAQTSTERFEMWGDGLQTRSFTFIDECVEGVLRLTKSDFREPVNIGSDEMVSMNEMAEIVLSFEDR-KLPIHHIPGPEGVRGRNSDNTLIKEKLGWAPTMRLKDGLRFTYFWIKEQIEKEKTQGI-DVA-AYGSSKVVSTQAPVQLGSLRAADGKEGL-----------------------

>PvGME-2

MG-SSEKT-VT-AY------GEYTY-AELEREPYWPSEKLRISITGAGGFIGSHIARRLKSEGHYIIASDWKKNEHMTEDMFCHEFHLVDLRVMDNCLKVTQGVDHVFNLAADMGGMGFIQSNHSVIMYNNTMISFNMLEAARINGVKRFFYASSACIYPEFKQLDT-N--V---SLKESDAWPAEPQDAYGLEKLATEELCKHYTKDFGIECRIGRFHNIYGPFGTWKGGREKAPAAFCRKAQTSTERFEMWGDGLQTRSFTFIDECVEGVLRLTKSDFREPVNIGSDEMVSMNEMAEIVLSFEDR-KLPIHHIPGPEGVRGRNSDNTLIKEKLGWAPTMKLKDGLRFTYFWIKEQIEKEKTQGI-DIA-AYGSSKVVSTQAPVQLGSLRAADGKEGL-----------------------

>PvGME-3

MA----------LN------KEYTY-AELEKEPYWPFEKLRISITGAGGFIASHIARRLKSEGHYIIASDWKKNEHMTEDMFCHEFHLVDLRVMDNCLKVTTGVDHVFNLAADMGGMGFIQSNHSVIMYNNTMISFNMLEAARINGVKRFFYASSACIYPEFKQLET-V--V---SLKESDAWPAEPQDAYGLEKLATEELCKHYTKDFGIECRIGRFHNIYGPFGTWKGGREKAPAAFCRKALTSTGRFEMWGDGLQTRSFTFIDECVEGVLRLTKSDFREPVNIGSDEMVSMNEMAEIVLSFENK-QLPIHHIPGPEGVRGRNSDNTLIKEKLGWAPTMKLKDGLRITYFWIKEQLEKEKAEGM-DLS-VYGSSKVVQTQAPVQLGSLRAADGKE-------------------------

>PvGME-4

MG-SSEKT-VS-AY------GEYTY-AELEREPYWPSEKLRISITGAGGFIGSHIARRLKSEGHYIIASDWKKNEHMTEDMFCHEFHLVDLRVMDNCLKVTQGVDHVFNLAADMGGMGFIQSNHSVIMYNNTMISFNMLEAARINGVKRFFYASSACIYPEFKQLET-N--V---SLKESDAWPAEPQDAYGLEKLATEELCKHYTKDFGIECRIGRFHNIYGPFGTWKGGREKAPAAFCRKAQTSTERFEMWGDGLQTRSFTFIDECVEGVLRLTKSDFCEPVNIGSDEMVSMNEMAEIVLSFEDR-KLPIHHIPGPEGVRGRNSDNTLIKEKLGWAPTMKLKDGLRFTYFWIKEQIEKEKTQGV-DVA-AYGSSKVVSTQAPVQLGSLRAADGKEGL-----------------------

>PvGME-5

MG-SSEKT-VS-AY------GEYTY-AELEREPYWPSEKLRISITGAGGFIGSHIARRLKSEGHYIIASDWKKNEHMTEDM-----------VMDNCLKVTQGVDHVFNLAADMGGMGFIQSNHSVIMYNNTMISFNMLEAARINGVKRFFYASSACIYPEFKQLDT-N--V---SLKESDAWPAEPQDAYGLEKLATEELCKHYTKDFGIECRIGRFHNIYGPFGTWKGGREKAPAAFCRKAQTSTERFEMWGDGLQTRSFTFIDECVEGVLRLTKSDFREPVNIGSDEMVSMNEMAEIVLSFEDR-KLPIHHIPGPEGVRGRNSNNTLIKEKLGWAPTMKLKDGLRFTYFWIKEQVEKEKKQGF-DIA-AYGSSKVVSTQAPVQLGSLRAADGKEGL-----------------------

>RcGME

MG-SS--E-GT-NY------GAYTY-ENLEREPYWPSEKLRISITGAGGFIASHIARRLKSEGHYIIASDWKKNEHMTEDMFCHEFHLVDLRVMDNCLKVTKGVDHVFNLAADMGGMGFIQSNHSVIMYNNTMISFNMLEASRINGVKRLFYASSACIYPEFKQLDT-N--V---SLKESDAWPAEPQDAYGLEKLATEELCKHYTKDFGIECRIGRFHNIYGPFGTWKGGREKAPAAFCRKALTSTDKFEMWGDGLQTRSFTFIDECVEGVLRLTKSDFREPVNIGSDEMVSMNEMAEIVLSFEDR-KLPIHHIPGPEGVRGRNSDNTLIKEKLGWAPTMRLKDGLRITYFWIKEQIEKEKSQGV-DLS-IYGSSKVVGTQAPVQLGSLRAADGKE-------------------------

>SbGME-1

MA----------LN------KEYTY-AELEKEPYWPFEKLRISITGAGGFIASHIARRLKSEGHYIIASDWKKNEHMSEDMFCHEFHLVDLRVMDNCLKVTTGVDHVFNLAADMGGMGFIQSNHSVIMYNNTMISFNMLEAARINGVKRFFYASSACIYPEFKQLET-V--V---SLKESDAWPAEPQDAYGLEKLATEELCKHYTKDFGIECRVGRFHNIYGPFGTWKGGREKAPAAFCRKALTSTGHFEMWGDGLQTRSFTFIDECVEGVLRLTKSDFREPVNIGSDEMVSMNEMAEIVLSFENK-QLPIHHIPGPEGVRGRNSDNTLIKEKLGWAPTMRLKDGLRITYFWIKEQLEKEKAEGM-DLS-VYGSSKVVQTQAPVQLGSLRAADGKE-------------------------

>SbGME-2

MG-SGEKT-VT-AY------GEYTY-AELEREPYWPSEKLRISITGAGGFIGSHIARRLKSEGHYIIASDWKKNEHMTEDMFCHEFHLVDLRVMDNCLKVTQGVDHVFNLAADMGGMGFIQSNHSVIMYNNTMISFNMLEAARINGVKRFFYASSACIYPEFKQLDT-N--V---SLKESDAWPAEPQDAYGLEKLATEELCKHYTKDFGIECRVGRFHNIYGPFGTWKGGREKAPAAFCRKAQTSTERFEMWGDGLQTRSFTFIDECVEGVLRLTKSDFREPVNIGSDEMVSMNEMAEIVLSFEDR-KLPIHHIPGPEGVRGRNSDNTLIKEKLGWAPTMKLKDGLRFTYFWIKEQIEKEKTQGV-DIA-AYGSSKVVSTQAPVQLGSLRAADGKEGL-----------------------

>SfGME-1

MA-SN--GVKEVVY------GEYTA-SNLERELYWPQEKLRISITGAGGFIASHIARRLKTEGHYIVASDWKKNEHMSEDMFCDEFHLVDLRVMDNCLAVTKGVQHVFNLAADMGGMGFIQSNHSVIMYNNTMISFNMLEAARINGVHRFFYASSACIYPEFKQLET-N--V---SLKESDAWPAEPQDAYGLEKLATEELCKHYTKDFKMECRIGRFHNIYGPFGTWKGGREKAPAAFCRKALTAVDKFEMWGDGLQTRSFTFIDECVEGVLRLTKSDFQEPLNIGSDEMVSMNEMAEIVLSFENK-KLPIEHIPGPEGVRGRNSDNTLIKEKLGWAPTMRLKDGLRITYLWIKEQIEKEKTQGV-DLAGKYASSKVVGTQAPVQLGSLRAADGKE-------------------------

>SfGME-2

MVISN--GLKE--Y------GEYTA-SNLDREPYWPEQKLRISITGAGGFIASHIARRLKSEGHYIVASDWKKNEHMSEDMFCDEFHLVDLRVMDNCLAVTKGVQHVFNLAADMGGMGFIQSNHSVIMYNNTMISFNMLEAARINGIHRFFYASSACIYPEFKQLET-N--V---SLKESDAWPAEPQDAYGLEKLATEELCKHYTKDFKMECRIGRFHNIYGPFGTWKGGREKAPAAFCRKALTAVDKFEMWGDGLQTRSFTFIDECVEGVLRLTKSDFQEPLNIGSDEMVSMNEMAEIVLSFENK-KLPIEHIPGPEGVRGRNSDNTLIKEKLGWAPSMRLKDGLRITYLWIKEQIEKEKTQGV-DLAGKYSSSKVVGTQAPVQLGSLRAADGKE-------------------------

>SiGME-1

MG-SSEKT-VT-AY------GEYTY-AELEREPYWPSEKLRISITGAGGFIGSHIARRLKSEGHYIIASDWKKNEHMTEDMFCHEFHLVDLRVMDNCLKVTQGVDHVFNLAADMGGMGFIQSNHSVIMYNNTMISFNMLEAARINDVKRFFYASSACIYPEFKQLDT-N--V---SLKESDAWPAEPQDAYGLEKLATEELCKHYTKDFGIECRVGRFHNIYGPFGTWKGGREKAPAAFCRKAQTSTERFEMWGDGLQTRSFTFIDECVEGVLRLTKSDFREPVNIGSDEMVSMNEMAEIVLSFEDR-KLPIHHIPGPEGVRGRNSDNTLIKEKLGWAPTMKLKDGLRFTYFWIKEQIEKEKTQGI-DIA-GYGSSKVVSTQAPVQLGSLRAADGKEGL-----------------------

>SiGME-2

MG-SSEKT-VS-TY------GEYTY-AELEREPYWPSEKLRISITGAGGFIGSHIARRLKSEGHYIIASDWKKNEHMTEDMFCHEFHLVDLRVMDNCLKVTQGVDHVFNLAADMGGMGFIQSNHSVIMYNNTMISFNMLEAARINGVKRFFYASSACIYPEFKQLET-N--V---SLKESDAWPAEPQDAYGLEKLATEELCKHYTKDFGIECRVGRFHNIYGPFGTWKGGREKAPAAFCRKAQTSTERFEMWGDGLQTRSFTFIDECVEGVLRLTKSDFREPVNIGSDEMVSMNEMAEIVLSFEDR-KLPIHHIPGPEGVRGRNSDNTLIKEKLGWAPTMKLKDGLRFTYFWIKEQIEKEKTQGV-DIA-AYGSSKVVSTQAPVQLGSLRAADGKEGL-----------------------

>SiGME-3

MA----------LN------KEYTY-AELEKEPYWPFEKLRISITGAGGFIASHIARRLMSEGHYIIASDWKKNEHMTEEMFCHEFHLIDLRVMDNCLKVTTGVDHVFNLAADMGGMGFIQSNHSVIMYNNTMISFNMLEAARINGVKRFFYASSACIYPEFKQLET-V--V---SLKESDAWPAEPQDAYGLEKLATEELCKHYTKDFGIECRIGRFHNIYGPFGTWKGGREKAPAAFCRKALTSTGRFEMWGDGLQTRSFTFIDECVEGVLRLTKSDFREPVNIGSDEMVSMNEMAEIVLSFENK-QLPIHHIPGPEGVRGRNSDNTLIKEKLGWAPTMKLKDGLRITYFWIKEQLEKEKAEGM-DLS-VYGSSKVVQTQAPVQLGSLRAADGKE-------------------------

>SmGME-1

ME----------SF------GAYTVGAELEREAYWPEAKLRICITGAGGFIASHIARRLKAEGHYIIASDWKKNEHMSEDMFCHEFHLVDLRVMDNCMVVTKGADHVFNLAADMGGMGFIQSNHSVILFNNTMISFNMLEASRINGVKRFFYASSACIYPEFKQLET-N--V---SLKEGDAWPAEPQDAYGLEKLCTEELCKHYTKDFGIECRIGRFHNIYGPFGTWKGGREKAPAAFCRKALTSTDKFEMWGDGMQTRSFTFIDECVEGVLRLTKSDFREPLNIGSDEMVSMNEMAEMILGFENK-QIPIQHIPGPEGVRGRNSDNSLIKEKLGWAPSMRLRDGLRITYMWIKEQLEREMAEGSHDLAAAYSSSKVVGTQAPVQLGSLRKADGKE-------------------------

>SmGME-2

ME----------CF------GAYTVGAELEREAYWPQAKLRISISGAGGFIASHIARRLKAEGHYVIASDWKKNEHMSEDMFCHEFHLVDLRVMENCLAVTRGVDHVFNLAADMGGMGFIQSNHSVILFNNTMISFNMLEASRINGVKRFFYASSACIYPEFKQLET-N--V---SLKESDAWPAEPQDAYGLEKLCTEELCKHYTKDFGIECRIGRFHNIYGPFGTWKGGREKAPAAFCRKALTSTDSFEMWGDGMQTRSFTFIDECVEGVLRLTKSDFREPLNIGSDEMVSMNEMAEIILGFEKK-QIPIRHIPGPEGVRGRNSDNSLIKEKLGWAPSMRLKDGLRITYLWIKEQLDKEIGEGSQDLLAGYSSSKVVGTQAPVQLGSLRKADGKE-------------------------

>SmGME-3

ME----------SF------GAYTVGAELERKAYWPESKLRICITGAGGFIASHIARRLKAEGHYIIASDWKKNEHMSEDMFCHEFHLVDLRVMDNCMVVTRGADHVFNLAADMGGMGFIQSNHSVILFNNTMISFNMLEASRINGVKRFFYASSACIYPEFKQLET-N--V---SLKEGDAWPAEPQDAYGLEKLCTEELCKHYTKDFGIECRIGRFHNIYGPFGTWKGGREKAPAAFCRKALTSTDKFEMWGDGMQTRSFTFIDECVEGVLRLTKSDFREPLNIGSDEMVSMNEMAEMILGFENK-QIPIQHIPGPEGVRGRNSDNSLIKEKLGWAPSMRLRDGLRITYMWIKEQLEKEMAEGSHDLAAAYSSSKVVGTQAPVQLGSLRKADGKE-------------------------

>SmGME-4

MG-----S----HY------GDYTAGAELERETYWPNEKLRISISGAGGFIASHIARRLKAEGHYVIASDWKKNEHMSEEMFCHEFHLVDLRVMENCLAVTKGVDHVFNLAADMGGMGFIQSNHSVILFNNTMISFNMLEAARINGVKRFFYASSACIYPEFKQLET-N--V---SLKESDAWPAEPQDAYGLEKLCSEELCKHYTKDFGIECRIGRFHNIYGPFGTWKGGREKAPAAFCRKALTSTDKFEMWGDGKQTRSFTFIDECVEGVLRLTKSDFREPLNIGSDEMVSMNEMAEIILGFGDK-KLPIHHIPGPEGVRGRNSDNNLIKEKLGWAPTMRLKDGLRITYMWIKEQLDEEISEGQ-DMA-AYSSSKVVGTQAPVQLGSLRKADGKE-------------------------

>SmGME-5

ME----------SF------GAYTVGAELEREAYWPQAKLRISISGAGGFIASHIARRLKAEGHYVIASDWKKNEHMSEDMFCHEFHLVDLRVMENCLAVTRGVDHVFDLAADMGGMGFIQSNHSVILFNNTMISFNMLEASRINGVKRFFYASSACIYPEFKQLET-N--V---SLKESDAWPAEPQDAYGLEKLCTEELCKHYTKDFGIECRIGRFHNIYGPFGTWKGGREKAPAAFCRKALTSTDSFEMWGDGMQTRSFTFIDECVEGVLRLTKSDFREPLNIGSDEMVSMNEMAEIILGFENK-QIPIRHIPGPEGVRGRNSENSLIKEKLGWAPSMRLKDGLRITYLWIKEQLDKEIGEGSQDLLTGYSSSKVVGTQAPVQLGSLRKADGKE-------------------------

>SmGME-6

ME----------SF------GAYTVGAELEREAYWPEAKLRICITGAGGFIASHIARRLKAEGHYIIASDWKKNEHMSEDMFCHEFHLVDLRVMDNCMVVTRGADHVFNLAADMGGMGFIQSNHSVILFNNTMISFNMLEASRINGVKRFFYASSACIYPEFKQLET-N--V---SLKEGDAWPAEPQDAYGLEKLCTEELCKHYTKDFGIECRIGRFHNIYGPFGTWKGGREKAPAAFCRKALTSTDKFEMWGDGMQTRSFTFIDECVEGVLRLTKSDFREPLNIGSDEMVSMNEMAEMILGFENK-QIPIQHIPGPEGVRGRNSDNSLIKEKLGWAPSMRLRDGLRITYMWIKEQLEKEMAEGSHDLAAAYSSSKVVGTQAPVQLGSLRKADGKE-------------------------

>SmGME-7

ME----------SF------GAYTVGAELEREAYWPAAKLRICITGAGGFIASHIARRLKAEGHYIIASDWKKNEHMSEDMFCHEFHLVDLRVMDNCMVVTKGVDHVFNLAADMGGMGFIQSNHSVILFNNTMISFNMLEASRINGVKRFFYASSACIYPEFKQLET-N--V---SLKEGDAWPAEPQDAYGLEKLCTEELCKHYTKDFGIECRIGRFHNIYGPFGTWKGGREKAPAAFCRKALTSTDKFEMWGDGMQTRSFTFIDECVEGVLRLTKSDFREPLNIGSDEMVSMNEMAEMILGFENK-QIPIQHIPGPEGVRGRNSDNSLIKEKLGWAPSMRLRDGLRITYMWIKEQLEKEMAEGSHDLAAAYSSSKVVGTQAPVQLGSLRKADGKE-------------------------

>SmGME-8

ME----------CF------GAYTVGAELEREAYWPQAKLRISISGAGGFIASHIARRLKAEGHYVIASDWKKNEHMSEDMFCHEFHLVDLRVMENCLAVTRGVDHVFNLAADMGGMGFIQSNHSVILFNNTMISFNMLEASRINGVKRFFYASSACIYPEFKQLET-N--V---SLKESDAWPAEPQDAYGLEKLCTEELCKHYTKDFGIECRIGRFHNIYGPFGTWKGGREKAPAAFCRKALTSTDSFEMWGDGMQTRSFTFIDECVEGVLRLTKSDFREPLNIGSDEMVSMNEMAEIILGFEKK-QIPIRHIPGPEGVRGRNSDNSLIKEKLGWAPSMRLKDGLRITYSWIKEQLDKEIGEGSQDLLAGYSSSKVVGTQAPVQLGSLRKADGKE-------------------------

>SmGME-9

MG-----S----HY------GDYTAGAELEREAYWPNEKLRISISGAGGFIASHIARRLKAEGHYVIASDWKKNEHMSEEMFCHEFHLVDLRVMENCLAVTKGVDHVFNLAADMGGMGFIQSNHSVILFNNTMISFNMLEAARINGVKRFFYASSACIYPEFKQLET-N--V---SLKESDAWPAEPQDAYGLEKLCSEELCKHYTKDFGIECRIGRFHNIYGPFGTWKGGREKAPAAFCRKALTSTDKFEMWGDGKQTRSFTFIDECVEGVLRLTKSDFREPLNIGSDEMVSMNEMAEIILGFGDK-KLPIHHIPGPEGVRGRNSDNDLIKEKLGWAPTMRLKDGLRITYMWIKEELDKEISEGQ-DMA-AYSSSKVVGTQAPVQLGSLRKADGKE-------------------------

>SpGME-1

MG-SA--D-GS--Y------GAYTY-EALEREPYWPSEKLRISITGAGGFIASHIARRLKAEGHYIIASDWKKNEHMTEDMFCNEFHLVDLRVMDNCLKVTKGVDHVFNLAADMGGMGFIQSNHSVIMYNNTMISFNMLEASRINGVKRLFYASSACIYPEFKQLET-N--V---SLKEADAWPAEPQDAYGLEKLATEELCKHYTKDFGIECRIGRFHNIYGPFGTWKGGREKAPAAFCRKAITSIDKFEMWGDGLQTRSFTFIDECVEGVLRLTKSDFREPLNIGSDEMVSMNGMAEIVLSFDNK-NLPIHHIPGPEGVRGRNSDNTLIKEKLGWAPTMKLKDGLRITYFWIKEQIEKEKTQGI-DLS-IYGSSKVVGTQAPVQLGSLRAADGNE-------------------------

>SpGME-2

MG-SA--D-GS--Y------GAYTY-EALEREHYWPSEKLRISITGAGGFIASHIARRLKAEGHYIIASDWKKNEHMTEDMFCHEFHLVDLRVMDNCLKVTKDVDHVFNLAADMGGMGFIQSNHSVIMYNNTMISFNMLEASRINGVKRLFYASSACIYPEFKQLET-N--V---SLKESDAWPAEPQDAYGLEKLATEELCKHYTKDFGIECRIGRFHNIYGPFGTWKGGREKAPAAFCRKGLTSVDKFEMWGDGLQTRSFTFIDECVEGVLRLTKSDFREPVNIGSDEMVSMNEMAEIILSFENK-NLPIHHIPGPEGVRGRNSDNTLIKEKLGWAPTMRLKDGLRITYFWIKEQIEKEKSQGI-DLS-IYGSSKVVGTQAPVQLGSLRAADGKE-------------------------

>SpGME-3

MG-SA--D-GS--Y------GAYTY-EALEREPYWPSEKLRISITGAGGFIASHIARRLKAEGHYIIASDWKKNEHMTEDMFCNEFHLVDLRVMDNCLKVTKGVDHVFNLAADMGGMGFIQSNHSVIMYNNTMISFNMLEASRINGVKRLFYASSACIYPEFKQLET-N--V---SLKEADAWPAEPQDAYGLEKLATEELCKHYTKDFGIECRIGRFHNIYGPFGTWKGGREKAPAAFCRKAITSIDKFEMWGDGLQTRSFTFIDECVEGVLRLTKSDFREPLNIGSDEMVSMNGMAEIVLSFDNK-NLPIHHIPGPEGVRGRNSDNTLIKEKLGWAPTMKLKDGLRITYFWIKEQIEKEKTQGI-DLS-IYGSSKVVGTQAPVQLGSLRAADGNE-------------------------

>SppGME

MA-NG-------SY------GEYTY-ENLEREPYWPSEKLRISITGAGGFIASHIARRLKSEGHYIIASDWKKNEHMTEDMFCHEFHLVDLRVMENCLKVTKDVDHVFNLAADMGGMGFIQSNHSVIMYNNTMISFNVLEAARVNDVKRLFYASSACIYPEFKQLET-N--V---SLKESDAWPAEPQDAYGLEKLASEELCKHYTKDFGIECRIGRFHNIYGPFGTWKGGREKAPAAFCRKAITSTDKFEMWGDGLQTRSFTFIDECVEGVLRLTKSDFREPVNIGSDEMVSMNEMAEIVLSFEDR-MLPIHHIPGPEGVRGRNSDNTLIKEKLGWAPTMRLKDGLRITYFWIKEQIEKEKAQGI-DLS-VYGSSKVVGTQAPVQLGSLRAADGKE-------------------------

>SvGME-1

MA----------LN------KEYTY-AELEKEPYWPFEKLRISITGAGGFIASHIARRLMSEGHYIIASDWKKNEHMTEEMFCHEFHLIDLRVMDNCLKVTTGVDHVFNLAADMGGMGFIQSNHSVIMYNNTMISFNMLEAARINGVKRFFYASSACIYPEFKQLET-V--V---SLKESDAWPAEPQDAYGLEKLATEELCKHYTKDFGIECRIGRFHNIYGPFGTWKGGREKAPAAFCRKALTSTGRFEMWGDGLQTRSFTFIDECVEGVLRLTKSDFREPVNIGSDEMVSMNEMAEIVLSFENK-QLPIHHIPGPEGVRGRNSDNTLIKEKLGWAPTMKLKDGLRITYFWIKEQLEKEKAEGM-DLS-VYGSSKVVQTQAPVQLGSLRAADGKE-------------------------

>SvGME-2

MG-SSEKT-VT-AY------GEYTY-AELEREPYWPSEKLRISITGAGGFIGSHIARRLKSEGHYIIASDWKKNEHMTEDMFCHEFHLVDLRVMDNCLKVTQGVDHVFNLAADMGGMGFIQSNHSVIMYNNTMISFNMLEAARINDVKRFFYASSACIYPEFKQLDT-N--V---SLKESDAWPAEPQDAYGLEKLATEELCKHYTKDFGIECRVGRFHNIYGPFGTWKGGREKAPAAFCRKAQTSTERFEMWGDGLQTRSFTFIDECVEGVLRLTKSDFREPVNIGSDEMVSMNEMAEIVLSFEDR-KLPIHHIPGPEGVRGRNSDNTLIKEKLGWAPTMKLKDGLRFTYFWIKEQIEKEKTQGI-DIA-AYGSSKVVSTQAPVQLGSLRAADGKEGL-----------------------

>SvGME-3

MG-SSEKT-VS-TY------GEYTY-AELEREPYWPSEKLRISITGAGGFIGSHIARRLKSEGHYIIASDWKKNEHMTEDMFCHEFHLVDLRVMDNCLKVTQGVDHVFNLAADMGGMGFIQSNHSVIMYNNTMISFNMLEAARINGVKRFFYASSACIYPEFKQLET-N--V---SLKESDAWPAEPQDAYGLEKLATEELCKHYTKDFGIECRVGRFHNIYGPFGTWKGGREKAPAAFCRKAQTSTERFEMWGDGLQTRSFTFIDECVEGVLRLTKSDFREPVNIGSDEMVSMNEMAEIVLSFEDR-KLPIHHIPGPEGVRGRNSDNTLIKEKLGWAPTMKLKDGLRFTYFWIKEQIEKEKTQGV-DIA-AYGSSKVVSTQAPVQLGSLRAADGKEGL-----------------------

>TcGME

MG-SA--D-GT-NY------GAFTY-EALEREPYWPSEKLRISITGAGGFIASHIARRLKSEGHYIIASDWKKNEHMTEDMFCHEFHLADLRVMDNCLKVTNGVDHVFNLAADMGGMGFIQSNHSVIMYNNTMISFNMLEAARINGVKRFFYASSACIYPEFKQLET-N--V---SLKESDAWPAEPQDAYGLEKLATEELCKHYTKDFGIECRIGRFHNIYGPFGTWKGGREKAPAAFCRKAITSTDKFEMWGDGLQTRSFTFIDECVEGVLRLTKSDFREPVNIGSDEMVSMNEMAEIVLSFEDK-KLPIHHIPGPEGVRGRNSDNTLIKEKLGWAPTMRLKDGLRITYFWIKEQIEKEKSQGI-DLT-IYGSSKVVGTQAPVQLGSLRAADGKE-------------------------

>TpGME-1

MG-ST--G-KT-NY------GEYTY-ENLEREPYWPSEKLKISITGAGGFIASHLARRLKTEGHYIIASDWKKNEHMTEDMFCDEFHLVDLRVMDNCLTVTKGVDHVFNLAADMGGMGFIQSNHSVIMYNNTMISFNMIEAARINGIKRFFYASSACIYPEFKQLETTN--V---SLKESDAWPAEPQDAYGLEKLATEEICKHYNKDFGIECRIGRFHNIYGPFGTWKGGREKAPAAFCRKAITSTDKFEMWGDGLQTRSFTFIDECVEGVLRLTKSDFREPVNIGSDEMVSMNEMAEIVLGFDNK-KTPIHHIPGPEGVRGRNSDNTLIKEKLGWAPTMKLKDGLRITYIWIKEQLEKENAQGI-DTS-GYGSSKVVQTQAPVQLGSLRAADGKESG-----------------------

>TpGME-2

MG-SS--G-TN-DY------GSYTY-QNLEREPYWPSEKLRISITGAGGFIASHIARRLKTEGHYIIASDWKKNEHMTEDMFCHEFHLVDLRVMDNCLKVTENVDHVFNLAADMGGMGFIQSNHSVIMYNNTMISFNMIEAARINGVKRFFYASSACIYPEFKQLET-N--V---SLKEADAWPAEPQDAYGLEKLATEELCKHYNKDFGIECRIGRFHNIYGPYGTWKGGREKAPAAFCRKTLTSTDKFEMWGDGLQTRSFTFIDECVEGVLRLTKSDFREPVNIGSDEMVSMNEMAEIVLSFEDK-SIPIQHIPGPEGVRGRNSDNTLIKEKLGWAPTMKLKDGLRITYFWIKEQLEKEKAGGV-DVT-SYGSSKVVSTQAPVQLGSLRAADGNE-------------------------

>VcGME

MS-AD--TVHE-QY------ASVSKLAKYPFEPYWPDKKLKICVTGAGGFIASHLAKRLKSEGHYIVACDWKRNEHFAEEEFCHEFHLVDLRLFENCKKVAEGCEHVFNLAADMGGMGFIQSNHSVILYNNTMVSFNMMEAARVCGVKRFFYASSACIYPEFKQLDT-Q--VEGGGLKEADAWPAQPQDAYGLEKLVSEELGKHYGKDFGIDVRLARFHNIYGPHGTWKGGREKAPAAFCRKVLTSTTEIEMWGDGKQTRSFTFIDDCVEGILRITKSDFTEPLNLGSTEMVSMNEMMEMAMSFEDK-KLPIKHIPGPEGVRGRNSDNKLILEKLGWEPTVSLRDGLKMTYFWIKSQIEKEAESGV-DAS-KYSHSTIVQTSAPVELGSLRKADGQEGF-----------------------

>ZmGME-1

MG-SSEKT-VT-AY------GEYTY-AELEREPYWPSEKLRISITGAGGFIGSHIARRLKNEGHYIIASDWKKNEHMTEDMFCHEFHLVDLRVMDNCLKVTHGVDHVFNLAADMGGMGFIQSNHSVIMYNNTMISFNMLEAARINGVKRFFYASSACIYPEFKQLDT-N--V---SLKESDAWPAEPQDAYGLEKLATEELCKHYTKDFGIECRVGRFHNIYGPFGTWKGGREKAPAAFCRKAQTSTERFEMWGDGLQTRSFTFIDECVEGVLRLTKSDFREPVNIGSDEMVSMNEMAEIVLGFEDR-KLPIHHIPGPEGVRGRNSDNTLIKEKLGWAPTMKLKDGLRFTYFWIKEQIEKEKTQGV-DIA-AYGSSKVVSTQAPVQLGSLRAADGKEGL-----------------------

>ZmGME-2

MA----------LN------KEYTY-AELEKEPYWPFEKLRVSITGAGGFIASHIARRLKGEGHYVVASDWKRNEHMPEDMFCHEFHLVDLRVMDNCLKVTTGVDHVFNLAADMGGMGFIQSNHSVIMYNNTMISFNMLEAARINGVKRFFYASSACIYPEFKQLET-V--V---SLKESDAWPAEPQDAYGLEKLATEELCKHYTKDFGIECRIGRFHNIYGPFGTWKGGREKAPAAFCRKALTSTGRFEMWGDGLQTRSFTFIDECVEGVLRLTKSDFREPVNIGSDEMVSMNEMAEMVLSFENK-QLPIHHIPGPEGVRGRNSDNTLIKEKLGWAPTMRLKDGLRITYSWIKEQLEKEKAEGM-DLS-VYGSSKVVQTQAPVQLGSLRAADGKE-------------------------
